# Imidazole-Derived Alkyl and Aryl Ethers: Synthesis, Characterization, In Vitro Anticancer and Antioxidant Activities, Carbonic Anhydrase I–II Inhibition Properties, and In Silico Studies

**DOI:** 10.1021/acsomega.4c00028

**Published:** 2024-05-03

**Authors:** Mays Faris, Hayrani Eren Bostancı, İbrahim Özcan, Mustafa Öztürk, Ümit Muhammed Koçyiğit, Taner Erdoğan, Hakan Tahtaci

**Affiliations:** † Department of Chemistry, Faculty of Science, Karabuk University, 78050 Karabuk, Türkiye; ‡ Department of Biochemistry Sivas, Faculty of Pharmacy, Sivas Cumhuriyet University, 58010 Sivas, Türkiye; § Sivas Vocational School of Technical Sciences, Sivas Cumhuriyet University, 58010 Sivas, Türkiye; ∥ Kocaeli Vocational School, Department of Chemistry and Chemical Processing Technologies, Kocaeli University, 41140 Kocaeli, Türkiye

## Abstract

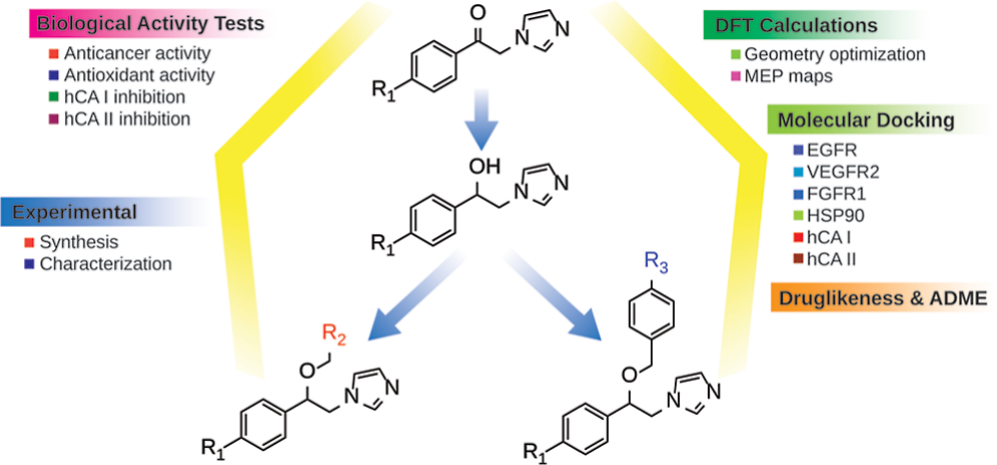

Imidazole derivatives display extensive applications in pharmaceutical chemistry and have been investigated as bioactive compounds for medicinal chemistry. In this study, besides the starting materials (**3a**–**c** and **4a**–**c**), synthesis, characterization, and biological activity studies were conducted on a total of 18 compounds, nine of which are known and the other nine are original. The compounds investigated in the study are a series of alkyl (**7**–**15**) and aryl (**16**–**24**) ether derivatives bearing substituted phenyl and imidazole rings, which were characterized using various methods including ^1^H NMR, ^13^C NMR, FT-IR analysis, elemental analysis, and mass spectroscopy. Computer-aided drug design studies have been carried out to predict the biological activities of compounds. Besides DFT calculations, the binding affinities of the compounds to EGFR, VEGFR2, FGFR1, HSP90, hCA I, and hCA II were investigated. Additionally, drug-likeness and ADME analyses were performed on the compounds. Anticancer, antioxidant, and enzyme inhibition activity tests were performed in biological activity studies on the synthesized compounds. Among the synthesized compounds, compounds **17** and **19**–**24** generally exhibited inhibition profiles against the widespread cytosolic hCA I isozyme with IC_50_ values ranging from 4.13 to 15.67 nM and cytosolic hCA II isozyme with IC_50_ values ranging from 5.65 to 14.84 nM. L929 (mouse fibroblast cell line) was used as the control healthy cell line, and MCF7 (breast cancer), C6 (rat glioblastoma), and HT-29 (colon cancer) cells were used in cell culture studies as cancer cell lines. Before the study on cancer cells, all compounds were examined on healthy cells, and their cytotoxicity was determined. As a result of these data, studies continued with six compounds determined to be nontoxic. On cancerous cells, it was determined that compounds **3a, 3b, 4a, 4b, 4c**, and **7** had cytotoxic effects on both colon cancer and brain tumors. It was found that compound **3b** had a more toxic effect than cisplatin on the glioma cell line with an IC_50_ value of 10.721 ± 0.38 μM, and compound **3a** had a more toxic effect on the colon cancer cell line with an IC_50_ value of 20.88 ± 1.02 μM. However, it was determined that the same compounds did not have a statistically significant effect on breast cancer. Flow cytometry studies also showed that when the IC_50_ dose of compound **3b** was applied to the C6 cell line, the cells tended to early and late apoptosis. Additionally, it has been shown by flow cytometry that the cell cycle stops in the G0/G1 phase. A similar effect was observed in the colon cancer cell line with compound **3a**. Compound **3b** caused early and late apoptosis of the colon cancer cell line with the applied IC_50_ dose and stopped the cell cycle in the G0/G1 phase. Finally, the FRAP method studied all synthesized compounds’ antioxidant effects. According to the measured antioxidant power results, it was determined that no compound had a more effective reducing power than vitamin E.

## 1 Introduction

**Figure 1:**
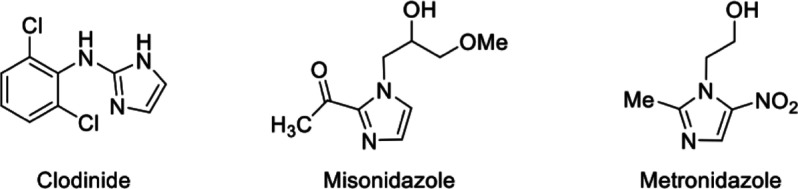
Some examples of drugs containing imidazole group.

The discovery of novel and effective medicinal compounds is of great importance due to many undesirable effects of current drugs. Therefore, various heterocyclic ring systems have been studied for the development of novel compounds with clinical effects.^1−6^ Imidazole analogues have attracted the attention of many researchers due to their diverse biological and pharmaceutical properties, such as antiparasitic, analgesic, anticonvulsant, anti-inflammatory, anticancer, antimalarial, antitubercular, antiviral, and antimicrobial activities.^7−21^ Therefore, including the electron-enriched imidazole ring in structures has become necessary in the pharmaceutical industries’ design, formulation, and development of new and effective drugs.^6^ The structures of some commercially used compounds containing imidazole groups, such as clonidine (α2-adrenergic agonist), misonidazole (photoreceptor used in radiation therapy), and metronidazole (antibiotic), are given in Figure 1.

Despite the advances in research and treatment, cancer remains a major global health problem, affecting millions of lives each year. The development of anticancer drugs is of paramount importance. The development of novel medicines holds the promise of increasing therapeutic efficacy while minimizing side effects, thereby improving patient outcomes and quality of life. On the other hand, the continuous evolution of cancer cells and the emergence of drug resistance require continuous research efforts to develop innovative therapeutic strategies that can overcome these challenges.

Free radicals are chemical species produced as a byproduct of normal metabolism or due to environmental factors. Antioxidants are molecules that have the ability to prevent or repair cellular damage caused by free radicals. They can therefore contribute to the prevention of diseases caused by free radicals (such as diseases caused by the weakening of the immune system, heart diseases, cancer, Alzheimer’s disease, Parkinson’s disease, etc.). Thus, it was aimed to reveal the antioxidant activities of the compounds synthesized in this study, thus contributing to the future studies on developing new antioxidant agents.

Carbon dioxide and bicarbonate interconversion is a biochemical process of great importance in all living things.^22^ Carbonic anhydrase (CA) enzyme is a metalloenzyme that catalyzes this transformation, contains zinc (Zn^2+^) ion in its active site, and functions in many tissues.^24^ Inhibitors of CA isoenzymes are used as therapeutic targets for important diseases (cancer, epilepsy, osteoporosis, hypertension, and glaucoma) due to the widespread distribution of isoenzymes in tissues and their involvement in many important physiological/pathological events.^24,25^

Among the 12 hCA isoforms in the human body, hCA I and hCA II, in particular, play a role in epilepsy treatment as well as other physiologically vital processes such as respiration, pH balance, electrolyte secretion, pathophysiology, and progression of vision loss in diabetes and glaucoma patients.^23^

Computer-aided drug design (CADD) is a pivotal domain in drug discovery and development. Utilizing computer simulations, modeling, and computational chemistry techniques, CADD expedites the process by conducting numerous steps virtually before any laboratory work commences. This approach accelerates timelines and diminishes expenses significantly. Computers adeptly simulate drug interactions with target molecules on a molecular scale, aiding in the comprehension, optimization, and identification of optimal molecular structures during the drug design phase. Therefore, CADD has increasingly become a growing important field and of greater interest. CADD has become increasingly important, especially in recent years, and has become a method that researchers use to understand how potential drugs interact with target molecules. In recent years, there have been several studies in the literature aimed at developing both anticancer drugs^26−29^ and HCA inhibitors^30−33^ using the CADD approach. On the other hand, drug-likeness and ADME analyses are also critical computational tools for drug discovery and development studies. By analyzing the ADME properties of potential drugs and their structural similarities to previously discovered drugs, an assessment can be made of the drug potential of the compounds under investigation. Methods commonly used in drug-likeness analysis include Lipinski’s Rule of Five,^34^ Ghose Filter,^35^ Veber’s Rule,^36^ Egan’s Rule,^37^ and Muegge’s Rule.^38^

For all these reasons, the current study aimed to investigate the possible enzyme inhibition, anticancer, and antioxidant activities of known compounds, as well as newly synthesized compounds, by carrying out experimental and theoretical calculations, with the aim of contributing to subsequent studies. For this purpose, besides the known compounds (**3a**–**c**, **4a**–**c**, **9**, **10**, **12**, **16**, **17**, **19**–**21**, and **23**),^39−46^ the syntheses of nine new compounds (**7, 8, 11**, **13**–**15**, **18**, **22,** and **24**) were accomplished. Compounds were characterized using methods such as ^1^H NMR, ^13^C NMR, FT-IR analysis, elemental analysis, and mass spectroscopy. Additionally, CADD studies were conducted to predict the biological activities of the compounds. Alongside DFT calculations, the binding affinities of the compounds to EGFR, VEGFR2, FGFR1, HSP90, hCA I, and hCA II were investigated. Additionally, drug-likeness and ADME analyses were performed on the compounds. Furthermore, the compounds’ anticancer effects with MTT and flow cytometry methods and antioxidant effects with the FRAP method (ferric reducing antioxidant power assay) were investigated, and hCA I (CA I), and hCA II (CA II) inhibition properties were experimentally examined.

## 2 Experimental Section

### 2.1 General Methods

**Scheme 1:**
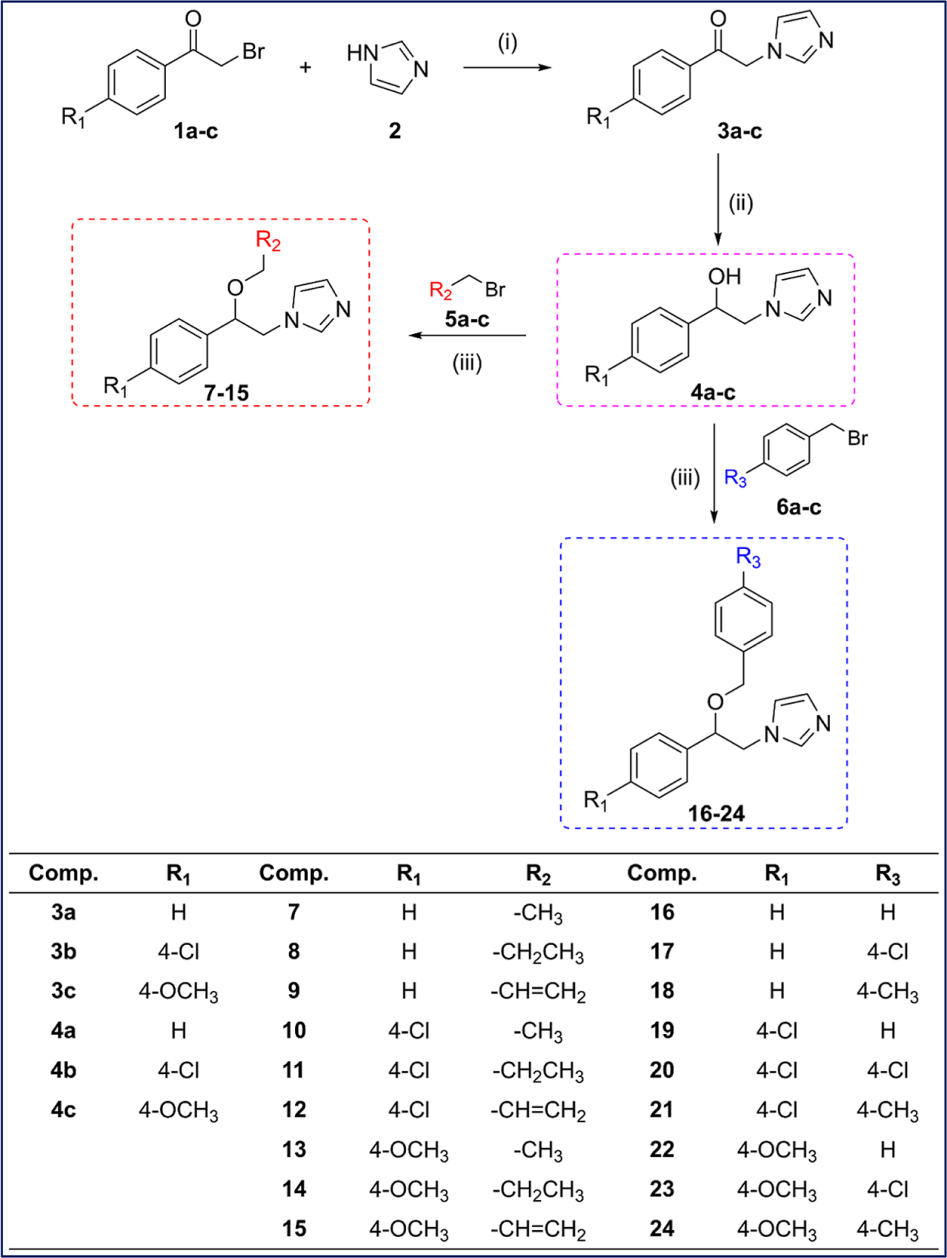
Synthesis of Alkyl (**7–15**) and Aryl (**16–24**) Ether Derivatives. Reagents and Conditions: (i) Acetone, Triethylamine 0–5 °C, 30 min, and r.t. 5 h; (ii) Absolute Ethyl Alcohol, NaBH_4_, 0–5 °C, 30 min, and r.t. 3 h; (iii) DMF, NaH, r.t. 6 h

The FT-IR spectra of the compounds were measured in ATR and a PerkinElmer Spectrum 100 FT-IR spectrometer. ^1^H NMR and ^13^C NMR spectra of the compounds were recorded on an Agilent Annual Refill (400 MHz) spectrometer at 400 and 100 MHz, respectively, in CDCl_3_ and DMSO with tetramethylsilane (TMS) as the internal reference. Chemical shift values are provided in ppm (parts per million), and the designation of signals was as follows: s, singlet; bs, broad singlet; d, doublet; dd, doublet of doublet; t, triplet; q, quartet; and m, multiplet. The mass spectra of the compounds were determined by the ESI(+) method, and a Thermo TSQ Quantum Access device was used. Elemental analysis was performed on a LECO 932 CHNS (Leco-932, St. Joseph, MI, USA) instrument, and the result was within ±0.4% of the theoretical values. The melting points were recorded on a Thermo Scientific IA9000 instrument. Thin layer chromatography (TLC) was carried out with silica gel 60 F_254_ aluminum TLC plates, and spots were observed in UV light of 254 nm. Column chromatography for the purification of the compounds was carried out with silica gel 70–230 mesh ASTM, and chloroform or chloroform-methanol was used as the solvent system. All compounds were synthesized via the synthetic routes shown in Scheme 1.

### 2.2 Synthesis of Ketone Derivatives (**3a–c**)

In a 250 mL two-neck round-bottom flask, acetophenone derivatives (**1a**–**c**) (0.02512 mol) were dissolved in acetone. Imidazole (**2**) (2.7363 g; 0.04019 mol) was added to this solution. The solution was then cooled to 0 °C with a salt-ice bath. At 0 °C, triethylamine (3.482 mL; 0.02512 mol) was added dropwise to this mixture (about 30 min). The reaction mixture was then stirred at room temperature for 5 h. The progress of the reaction was monitored by TLC. After completion of the reaction, the mixture was filtered to remove triethylamine hydrobromide salt precipitates; the precipitate was washed with acetone; and the total filtrate was evaporated in a rotary evaporator. The crude residue was suspended in brine and extracted with chloroform. The organic phase was dried with anhydrous sodium sulfate, filtered, and evaporated. The crude solid was crystallized from benzene–petroleum ether–2-propanol. The pure substance was dried in a vacuum oven with phosphorus pentoxide.

#### 2.2.1 2-(1*H*-Imidazol-1-yl)-1-phenylethanone (**3a**)

Yellowish solid, yield: 2.39 g (51%), mp 118–120 °C (benzene/petroleum ether/2-propanol). FT-IR (ATR, cm^–1^): 3129 (Ar–CH), 2933 (Aliph. CH), 1692 (C=O), 1596 (C=N). ^1^H NMR (400 MHz, CDCl_3_): δ (ppm) 5.35 (s, 2H), 6.87 (s, 1H), 7.04 (s, 1H), 7.42–7.48 (m, 3H), 7.59 (t, *J* = 7.2 Hz, 1H), 7.90 (d, *J* = 7.2 Hz, 2H). ^13^C NMR (100 MHz, CDCl_3_, δ ppm): 52.49, 120.37, 120.92, 129.05, 129.32, 134.10, 134.32, 138.14, 191.86. Anal. (% calcd/found) for C_11_H_10_N_2_O (MW: 186.21) C, 70.95/70.71; H, 5.41/5.35; N, 15.04/14.96. MS (ESI-*m*/*z*): 186.90 (M^+^, 100).

#### 2.2.2 1-(4-Chlorophenyl)-2-(1*H*-imidazol-1-yl)ethanone (**3b**)

White solid, yield: 3.43 g (62%), mp 161–163 °C (benzene/petroleum ether/2-propanol). FT-IR (ATR, cm^–1^): 3131 (Ar–CH), 2933 (Aliph. CH), 1689 (C=O), 1589 (C=N). ^1^H NMR (400 MHz, CDCl_3_): δ (ppm) 5.35 (s, 2H), 6.89 (s, 1H), 7.08 (s, 1H), 7.46 (d, *J* = 8.8 Hz, 3H), 7.87 (d, *J* = 6.8 Hz, 2H). ^13^C NMR (100 MHz, CDCl_3_, δ ppm): 52.40, 120.27, 129.34, 129.48, 129.58, 132.42, 138.09, 140.91, 190.66. Anal. (% calcd/found) for C_11_H_9_ClN_2_O (MW: 220.65) C, 59.88/59.76; H, 4.11/4.02; N, 12.70/12.79. MS (ESI-*m*/*z*): 220.80 (M^+^, 100).

#### 2.2.3 2-(1*H*-Imidazol-1-yl)-1-(4-methoxyphenyl)ethanone (**3c**)

Yellowish solid, yield: 3.15 g (58%), mp 137–138 °C (benzene/petroleum ether/2-propanol). FT-IR (ATR, cm^–1^): 3120 (Ar–CH), 2963 (Aliph. CH), 1680 (C=O), 1604 (C=N). ^1^H NMR (400 MHz, CDCl_3_): δ (ppm) 3.82 (s, 3H), 5.28 (s, 2H), 6.87 (s, 1H), 6.91 (d, *J* = 8.4 Hz, 2H), 7.03 (s, 1H), 7.42 (s, 1H), 7.87 (d, *J* = 8.8 Hz, 2H). ^13^C NMR (100 MHz, CDCl_3_, δ ppm): 52.09, 55.59, 114.22, 120.37, 127.07, 129.28, 130.29, 138.15, 164.34, 190.18. Anal. (% calcd/found) for C_12_H_12_N_2_O_2_ (MW: 216.24) C, 66.65/66.54, H: 5.59/5.67, N, 12.96/12.86. MS (ESI-*m*/*z*): 216.81 (M^+^, 100).

### 2.3 Synthesis of Alcohol Derivatives (**4a–c**)

In a 250 mL two-necked flask, ketone (0.0107 mol) was dissolved in absolute ethanol, and the solution was cooled to 0–5 °C in an ice bath. Then, NaBH_4_ (0.0214 mol) was dissolved in absolute ethanol and added dropwise to this mixture with a dropping funnel. The reaction mixture was stirred for about 30 min at 0–5 °C. Then, the mixture was stirred for another 3 h at room temperature. The solvent was evaporated with a rotary evaporator when the reaction was complete. The crude residue was suspended in brine and extracted with chloroform. The organic layer was dried over anhydrous sodium sulfate, filtered, and then evaporated to dryness. The solid was recrystallized from the benzene/petroleum ether (4:1). The pure matter was dried with phosphorus pentoxide in a vacuum oven.

#### 2.3.1 2-(1*H*-Imidazol-1-yl)-1-phenylethanol (**4a**)

White solid, yield: 1.51 g (75%), mp 149–151 °C (benzene/petroleum ether, 4:1). FT-IR (ATR, cm^–1^): 3119 (OH), 3055 (Ar–CH), 2894 (Aliph. CH), 1595 (C=N). ^1^H NMR (400 MHz, DMSO-*d*_6_): δ (ppm) 4.00–4.14 (m, 2H), 4.80 (s, 1H), 5.61 (bs, 1H, OH), 6.83 (s, 1H), 7.11 (s, 1H), 7.24–7.32 (m, 5H), 7.51 (s, 1H). ^13^C NMR (100 MHz, DMSO-*d*_6_, δ ppm): 54.04, 72.49, 120.54, 126.45, 127.77, 127.94, 128.51, 138.10, 143.05. Anal. (% calcd/found) for C_11_H_12_N_2_O (MW: 188.23) C, 70.19/70.03; H, 6.43/6.33; N, 14.88/14.74. MS (ESI-*m*/*z*): 188.87 (M^+^, 60), 190.83 (M + 2, 100).

#### 2.3.2 1-(4-Chlorophenyl)-2-(1*H*-imidazol-1-yl)ethanol (**4b**)

White solid, yield: 1.91 g (80%), mp 179–180 °C (benzene/petroleum ether, 4:1). FT-IR (ATR, cm^–1^): 3115 (OH), 3061 (Ar–CH), 2853 (Aliph. CH), 1594 (C=N). ^1^H NMR (400 MHz, DMSO-*d*_6_): δ (ppm) 4.00–4.13 (m, 2H), 4.83 (s, 1H), 5.82 (bs, 1H, OH), 6.81 (s, 1H), 7.08 (s, 1H), 7.34 (d, *J* = 5.2 Hz, 4H). 7.47 (s, 1H). ^13^C NMR (DMSO-*d*_6_, δ ppm): 53.76, 71.72, 120.53, 128.07, 128.32, 128.45, 132.24, 138.12, 142.02. Anal. (% calcd/found) for C_11_H_11_ClN_2_O (MW: 222.67) C, 59.33/58.14; H, 4.98/5.02; N, 12.58/12.47. MS (ESI-*m*/*z*): 222.83 (M^+^, 100).

#### 2.3.3 2-(1*H*-Imidazol-1-yl)-1-(4-methoxyphenyl)ethenol (**4c**)

White solid, yield: 1.98 g (85%), mp 104–105 °C (benzene/petroleum ether, 4:1). FT-IR (ATR, cm^–1^): 3123 (OH), 3062 (Ar–CH), 2969 (Aliph. CH), 1609 (C=N). ^1^H NMR (400 MHz, DMSO-*d*_6_): δ (ppm) 3.72 (s, 3H), 3.97–4.10 (m, 2H), 4.75 (d, *J* = 2.4 Hz, 1H), 5.68 (bs, 1H, OH), 6.81 (s, 1H), 6.87 (d, *J* = 8.4 Hz, 2H), 7.08 (s, 1H). 7.24 (d, *J* = 8.0 Hz, 2H), 7.46 (s, 1H). ^13^C NMR (DMSO-*d*_6_, δ ppm): 54.07, 55.46, 72.11, 113.89, 120.46, 127.63, 128.13, 135.07, 138.12, 158.97. Anal. (% calcd/found) for C_12_H_14_N_2_O_2_ (MW: 218.25) C, 66.04/66.17; H, 6.47/6.33; N, 12.84/12.74. MS (ESI-*m*/*z*): 218.84 (M^+^, 100).

### 2.4 General Procedure for the Synthesis of Alkyl (**7–15**) and Aryl (**16–24**) Ether Derivatives

In a 100 mL round-bottom flask, alcohol derivatives **(4a–c)** (2.12 mmol) were dissolved in DMF (about 6 mL). NaH (60% mineral oil dispersion, 3.19 mmol) was added in small fractions to this solution. The alkyl **(5a–c)** or aryl **(6a–c)** halides (2.12 mmol) were dissolved in DMF (about 4 mL) and added dropwise to this solution. The reaction mixture was stirred at room temperature for 6 h. TLC monitored the progress of the reaction at appropriate time intervals. After the completion of the reaction, the excess sodium hydride was decomposed with methyl alcohol, and then the solvent was evaporated under reduced pressure using a rotary evaporator. The crude residue was suspended in brine and extracted with dichloromethane or chloroform. The organic phase was dried over anhydrous sodium sulfate, filtered, and then evaporated with a rotary evaporator. The crude residue was purified by column chromatography on a silica-gel column using chloroform or chloroform–methanol mixture as the eluent to obtain target compounds. The pure matter was dried with phosphorus pentoxide in a vacuum oven. The physical properties and spectral data of the target compounds are listed below.

#### 2.4.1 1-(2-Ethoxy-2-phenylethyl)-1*H*-imidazole (**7**)

Yellowish oil, yield: 0.28 g (61%), *R*_f_ = 0.59 (CHCl_3_–CH_3_OH, 10:1). FT-IR (ATR, cm^–1^): 3111 (Ar–CH), 2974 (Aliph. CH), 1601 (C=N), 1099 (ROR). ^1^H NMR (400 MHz, CDCl_3_): δ (ppm) 1.12 (t, *J* = 7.0 Hz, 3H), 3.21–3.28 (m, 1H), 3.35–3.43 (m, 1H), 4.00–4.12 (m, 2H), 4.40–4.43 (m, 1H), 6.88 (s, 1H), 6.97 (s, 1H), 7.20 (d, *J* = 6.8 Hz, 2H), 7.26–7.34 (m, 3H), 7.39 (s, 1H). ^13^C NMR (100 MHz, CDCl_3_, δ ppm): 15.11, 53.41, 64.69, 81.34, 119.83, 126.41, 128.39, 128.70, 137.76, 138.78. Anal. (% calcd/found) for C_13_H_16_N_2_O (MW: 216.28) C, 72.19/72.05; H, 7.46/7.33; N, 12.95/12.88. MS (ESI-*m*/*z*): 217.13 (M+1, 100).

#### 2.4.2 1-(2-Phenyl-2-propoxyethyl)-1*H*-imidazole (**8**)

Yellowish oil, yield: 0.33 g (68%), *R*_f_ = 0.64 (CHCl_3_–CH_3_OH, 10:1). FT-IR (ATR, cm^–1^): 3110 (Ar–CH), 2962 (Aliph. CH), 1597 (C=N), 1099 (ROR). ^1^H NMR (400 MHz, CDCl_3_): δ (ppm) 0.85 (t, *J* = 7.4 Hz, 3H), 1.48–1.57 (m, 2H), 3.12–3.18 (m, 1H), 3.27–3.32 (m, 1H), 4.01–4.12 (m, 2H), 4.40–4.43 (m, 1H), 6.89 (s, 1H), 6.98 (s, 1H), 7.21 (d, *J* = 6.4 Hz, 2H), 7.26–7.36 (m, 3H), 7.40 (s, 1H). ^13^C NMR (100 MHz, CDCl_3_, δ ppm): 10.58, 22.91, 53.46, 70.95, 81.49, 119.85, 126.44, 128.38, 128.70, 137.78, 138.81. Anal. (% calcd/found) for C_14_H_18_N_2_O (MW: 230.31) C, 73.01/73.16; H, 7.88/7.71; N, 12.16/12.10. MS (ESI-*m*/*z*): 231.06 (M+1, 100).

#### 2.4.3 1-(2-(Allyloxy)-2-phenylethyl)-1*H*-imidazole (**9**)

Yellowish oil, yield: 0.36 g (75%), *R*_f_ = 0.52 (CHCl_3_–CH_3_OH, 10:1). FT-IR (ATR, cm^–1^): 3110 (Ar–CH), 2978 (Aliph. CH), 1604 (C=N), 1092 (ROR). ^1^H NMR (400 MHz, CDCl_3_): δ (ppm) 3.68–3.74 (m, 1H), 3.91–3.95 (m, 1H), 4.04–4.17 (m, 2H), 4.51 (q, *J* = 3.8 Hz, 1H), 5.11–5.16 (m, 2H), 5.71–5.81 (m, 1H), 6.89 (s, 1H), 6.99 (s, 1H), 7.21 (d, *J* = 7.6 Hz, 2H), 7.30–7.36 (m, 3H), 7.39 (s, 1H). ^13^C NMR (100 MHz, CDCl_3_, δ ppm): 53.32, 69.74, 80.53, 117.16, 119.85, 126.56, 128.56, 128.79, 128.85, 134.01, 137.79, 138.26. Anal. (% calcd/found) for C_14_H_16_N_2_O (MW: 228.29) C, 73.66/73.54; H, 7.06/7.00; N, 12.27/12.38. MS (ESI-*m*/*z*): 229.03 (M + 1, 100).

#### 2.4.4 1-(2-(4-Chlorophenyl)-2-ethoxyethyl)-1*H*-imidazole (**10**)

Yellowish oil, yield: 0.27 g (51%), *R*_f_ = 0.58 (CHCl_3_–CH_3_OH, 10:1). FT-IR (ATR, cm^–1^): 3111 (Ar–CH), 2974 (Aliph. CH), 1596 (C=N), 1087 (ROR). ^1^H NMR (400 MHz, CDCl_3_): δ (ppm) 1.12 (t, *J* = 6.8 Hz, 3H), 3.21–3.29 (m, 1H), 3.33–3.40 (m, 1H), 3.99–4.10 (m, 2H), 4.40 (q, *J* = 4.6 Hz, 1H), 6.86 (s, 1H), 6.97 (s, 1H), 7.13 (d, *J* = 8.4 Hz, 2H), 7.30 (d, *J* = 8.4 Hz, 2H), 7.37 (s, 1H). ^13^C NMR (100 MHz, CDCl_3_, δ ppm): 15.10, 53.20, 64.85, 80.64, 119.81, 127.77, 128.85, 128.93, 134.17, 137.30, 137.74. Anal. (% calcd/found) for C_13_H_15_ClN_2_O (MW: 250.72) C, 62.28/62.12; H, 6.03/5.95; N, 11.17/11.10. MS (ESI-*m*/*z*): 250.94 (M^+^, 100).

#### 2.4.5 1-(2-(4-Chlorophenyl)-2-propoxyethyl)-1*H*-imidazole (**11**)

Yellowish oil, yield: 0.36 g (64%), *R*_f_ = 0.55 (CHCl_3_–CH_3_OH, 10:1). FT-IR (ATR, cm^–1^): 3111 (Ar–CH), 2962 (Aliph. CH), 1596 (C=N), 1087 (ROR). ^1^H NMR (400 MHz, CDCl_3_): δ (ppm) 0.84 (t, *J* = 7.6 Hz, 3H), 1.47–1.56 (m, 2H), 3.12–3.18 (m, 1H), 3.23–3.29 (m, 1H), 3.99–4.10 (m, 2H), 4.39 (q, *J* = 4.8 Hz, 1H), 6.86 (s, 1H), 6.97 (s, 1H), 7.13 (d, *J* = 8.0 Hz, 2H), 7.30 (d, *J* = 8.4 Hz, 2H), 7.37 (s, 1H). ^13^C NMR (100 MHz, CDCl_3_, δ ppm): 10.56, 22.88, 53.22, 71.09, 80.78, 119.84, 127.81, 128.88, 128.92, 134.16, 137.31, 137.77. Anal. (% calcd/found) for C_14_H_17_ClN_2_O (MW: 264.75) C, 63.51/63.33; H, 6.47/6.41; N, 10.58/10.49. MS (ESI-*m*/*z*): 265.08 (M + 1, 100).

#### 2.4.6 1-(2-(Allyloxy)-2-(4-chlorophenyl)ethyl)-1*H*-imidazole (**12**)

Yellowish oil, yield: 0.47 g (85%), *R*_f_ = 0.46 (CHCl_3_–CH_3_OH, 10:1). FT-IR (ATR, cm^–1^): 3111 (Ar–CH), 2980 (Aliph. CH), 1597 (C=N), 1075 (ROR). ^1^H NMR (400 MHz, CDCl_3_): δ (ppm) 3.67–3.73 (m, 1H), 3.88–3.92 (m, 1H), 4.02–4.13 (m, 2H), 4.49 (q, *J* = 4.6 Hz, 1H), 5.13 (t, *J* = 8.2 Hz, 2H), 5.70–5.79 (m, 1H), 6.85 (s, 1H), 6.98 (s, 1H), 7.13 (d, *J* = 8.0 Hz, 2H), 7.30 (d, *J* = 8.4 Hz, 2H), 7.36 (s, 1H). ^13^C NMR (100 MHz, CDCl_3_, δ ppm): 53.08, 69.84, 79.75, 117.41, 119.84, 127.91, 128.96, 129.01, 133.76, 134.35, 136.76, 137.77. Anal. (% calcd/found) for C_14_H_15_ClN_2_O (MW: 262.73) C, 64.00/63.86; H, 5.75/5.65; N, 10.66/10.58. MS (ESI-*m*/*z*): 262.98 (M^+^, 100).

#### 2.4.7 1-(2-Ethoxy-2-(4-methoxyphenyl)ethyl)-1*H*-imidazole (**13**)

Yellowish oil, yield: 0.31 g (59%), *R*_f_ = 0.40 (CHCl_3_–CH_3_OH, 10:1). FT-IR (ATR, cm^–1^): 3111 (Ar–CH), 2973 (Aliph. CH), 1610 (C=N), 1093 (ROR). ^1^H NMR (400 MHz, CDCl_3_): δ (ppm) 1.12 (t, *J* = 7.0 Hz, 3H), 3.21–3.27 (m, 1H), 3.36–3.40 (m, 1H), 3.79 (s, 3H), 3.98–4.12 (m, 2H), 4.38 (q, *J* = 4.4 Hz, 1H), 6.87 (d, *J* = 8.8 Hz, 3H), 6.98 (s, 1H), 7.12 (d, *J* = 8.4 Hz, 2H), 7.40 (s, 1H). ^13^C NMR (100 MHz, CDCl_3_, δ ppm): 15.13, 53.47, 55.25, 64.44, 80.87, 114.08, 119.84, 127.65, 128.70, 130.73, 137.79, 159.63. Anal. (% calcd/found) for C_14_H_18_N_2_O_2_ (MW: 246.30) C, 68.27/68.12; H, 7.37/7.33; N, 11.37/11.28. MS (ESI-*m*/*z*): 247.02 (M + 1, 100).

#### 2.4.8 1-(2-(4-Methoxyphenyl)-2-propoxyethyl)-1*H*-imidazole (**14**)

Yellowish oil, yield: 0.36 g (65%), *R*_f_ = 0.50 (CHCl_3_–CH_3_OH, 10:1). FT-IR (ATR, cm^–1^): 3111 (Ar–CH), 2960 (Aliph. CH), 1610 (C=N), 1093 (ROR). ^1^H NMR (400 MHz, CDCl_3_): δ (ppm) 0.82 (t, *J* = 7.4 Hz, 3H), 1.47–1.52 (m, 2H), 3.09–3.14 (m, 1H), 3.23–3.28 (m, 1H), 3.77 (s, 3H), 3.97–4.10 (m, 2H), 4.35 (q, *J* = 4.4 Hz, 1H), 6.86 (t, *J* = 6.4 Hz, 3H), 6.96 (s, 1H), 7.11 (d, *J* = 8.4 Hz, 2H), 7.37 (d, *J* = 8.4 Hz, 1H). ^13^C NMR (100 MHz, CDCl_3_, δ ppm): 10.58, 22.89, 53.47, 55.22, 70.68, 80.96, 114.05, 119.87, 127.67, 128.62, 130.72, 137.77, 159.59. Anal. (% calcd/found) for C_15_H_20_N_2_O_2_ (MW: 260.33) C, 69.20/69.33; H, 7.74/7.61; N, 10.76/10.69. MS (ESI-*m*/*z*): 261.16 (M + 1, 100).

#### 2.4.9 1-(2-(Allyloxy)-2-(4-methoxyphenyl)ethyl)-1*H*-imidazole (**15**)

Yellowish oil, yield: 0.47 g (85%), *R*_f_ = 0.53 (CHCl_3_–CH_3_OH, 10:1). FT-IR (ATR, cm^–1^): 3111 (Ar–CH), 2935 (Aliph. CH), 1611 (C=N), 1075 (ROR). ^1^H NMR (400 MHz, CDCl_3_): δ (ppm) 3.66–3.70 (m, 1H), 3.77 (s, 3H), 3.86–3.90 (m, 1H), 3.99–4.13 (m, 2H), 4.44 (q, *J* = 4.4 Hz, 1H), 5.08–5.13 (m, 2H), 5.71–5.75 (m, 1H), 6.85 (d, *J* = 9.2 Hz, 3H), 6.96 (s, 1H), 7.10 (d, *J* = 8.4 Hz, 2H), 7.36 (s, 1H). ^13^C NMR (100 MHz, CDCl_3_, δ ppm): 53.31, 55.23, 69.45, 80.01, 114.13, 117.04, 119.87, 127.81, 128.72, 130.12, 134.11, 137.77, 159.71. Anal. (% calcd/found) for C_15_H_18_N_2_O_2_ (MW: 258.32) C, 69.74/69.56; H, 7.02/6.89; N, 10.84/10.78. MS (ESI-*m*/*z*): 259.06 (M + 1, 100).

#### 2.4.10 1-(2-(Benzyloxy)-2-phenylethyl)-1*H*-imidazole (**16**)

Yellowish oil, yield: 0.31 g (52%), *R*_f_ = 0.62 (CHCl_3_–CH_3_OH, 10:1). FT-IR (ATR, cm^–1^): 3110 (Ar–CH), 2941 (Aliph. CH), 1609 (C=N), 1095 (ROR). ^1^H NMR (400 MHz, CDCl_3_): δ (ppm) 4.05–4.10 (m, 1H), 4.14–4.24 (m, 2H), 4.47–4.55 (m, 2H), 6.88 (s, 1H), 7.02 (s, 1H), 7.15 (d, *J* = 6.4 Hz, 2H), 7.28–7.32 (m, 5H), 7.35–7.42 (m, 4H). ^13^C NMR (100 MHz, CDCl_3_, δ ppm): 53.35, 70.72, 80.46, 119.90, 126.71, 127.65, 127.77, 128.46, 128.69, 128.84, 128.90, 137.48, 137.84, 138.12. Anal. (% calcd/found) for C_18_H_18_N_2_O (MW: 278.35) C, 77.67/77.57; H, 6.52/6.34; N, 10.06/10.12. MS (ESI-*m*/*z*): 279.01 (M + 1, 100).

#### 2.4.11 1-(2-((4-Chlorobenzyl)oxy)-2-phenylethyl)-1*H*-imidazole (**17**)

Yellowish oil, yield: 0.58 g (88%), *R*_f_ = 0.61 (CHCl_3_–CH_3_OH, 10:1). FT-IR (ATR, cm^–1^): 3108 (Ar–CH), 2963 (Aliph. CH), 1598 (C=N), 1087 (ROR). ^1^H NMR (400 MHz, CDCl_3_): δ (ppm) 4.04–4.09 (m, 1H), 4.12–4.18 (m, 2H), 4.40–4.51 (m, 2H), 6.87 (s, 1H), 7.02 (t, *J* = 7.8 Hz, 3H), 7.24 (d, *J* = 8.0 Hz, 4H), 7.34–7.41 (m, 4H). ^13^C NMR (100 MHz, CDCl_3_, δ ppm) 53.25, 69.88, 80.58, 119.83, 126.67, 128.58, 128.79, 128.91, 128.94, 133.47, 135.98, 137.83. Anal. (% calcd/found) for C_18_H_17_ClN_2_O (MW: 312.79) C, 69.12/69.01; H, 5.48/5.35; N, 8.96/8.84. MS (ESI-*m*/*z*): 313.10 (M + 1, 100).

#### 2.4.12 1-(2-((4-Methylbenzyl)oxy)-2-phenylethyl)-1*H*-imidazole (**18**)

Yellowish oil, yield: 0.34 g (55%), *R*_f_ = 0.55 (CHCl_3_–CH_3_OH, 10:1). FT-IR (ATR, cm^–1^): 3109 (Ar–CH), 2921 (Aliph. CH), 1608 (C=N), 1092 (ROR). ^1^H NMR (400 MHz, CDCl_3_): δ (ppm) 2.33 (s, 3H), 4.03–4.08 (m, 1H), 4.12–4.19 (m, 2H), 4.33–4.54 (m, 2H), 6.88 (s, 1H), 7.03 (t, *J* = 7.4 Hz, 3H), 7.12 (d, *J* = 7.6 Hz, 2H), 7.27 (d, *J* = 6.8 Hz, 2H), 7.34–7.41 (m, 4H). ^13^C NMR (100 MHz, CDCl_3_, δ ppm): 21.17, 53.34, 70.58, 80.25, 119.93, 126.73, 127.80, 128.63, 128.79, 128.88, 129.14, 134.43, 137.49, 137.83, 138.23. Anal. (% calcd/found) for C_19_H_20_N_2_O (MW: 292.37) C, 78.05/78.00; H, 6.90/6.73; N, 9.58/9.45. MS (ESI-*m*/*z*): 293.01 (M + 1, 100).

#### 2.4.13 1-(2-(Benzyloxy)-2-(4-chlorophenyl)ethyl)-1*H*-imidazole (**19**)

Yellowish oil, yield: 0.59 g (89%), *R*_f_ = 0.58 (CHCl_3_–CH_3_OH, 10:1). FT-IR (ATR, cm^–1^): 3110 (Ar–CH), 2931 (Aliph. CH), 1597 (C=N), 1087 (ROR). ^1^H NMR (400 MHz, CDCl_3_): δ (ppm) 4.03–4.15 (m, 2H), 4.20 (d, *J* = 11.2 Hz, 1H), 4.43–4.52 (m, 2H), 6.85 (s, 1H), 6.99 (s, 1H), 7.12 (d, *J* = 6.8 Hz, 2H), 7.17 (d, *J* = 8.8 Hz, 2H), 7.27–7.34 (m, 5H), 7.38 (s, 1H). ^13^C NMR (100 MHz, CDCl_3_, δ ppm): 53.09, 70.83, 79.65, 119.87, 127.64, 127.88, 128.07, 128.49, 128.96, 129.10, 134.44, 136.62, 137.15, 137.80. Anal. (% calcd/found) for C_18_H_17_ClN_2_O (MW: 312.79) C, 69.12/69.02; H, 5.48/5.34; N, 8.96/8.78. MS (ESI-*m*/*z*): 313.10 (M + 1, 100).

#### 2.4.14 1-(2-((4-Chlorobenzyl)oxy)-2-(4-chlorophenyl)ethyl)-1*H*-imidazole (**20**)

Yellowish oil, yield: 0.65 g (89%), *R*_f_ = 0.62 (CHCl_3_–CH_3_OH, 10:1). FT-IR (ATR, cm^–1^): 3110 (Ar–CH), 2931 (Aliph. CH), 1597 (C=N), 1085 (ROR). ^1^H NMR (400 MHz, CDCl_3_): δ (ppm) 4.03–4.17 (m, 3H), 4.38–4.49 (m, 2H), 6.84 (s, 1H), 7.01 (t, *J* = 7.4 Hz, 3H), 7.16 (d, *J* = 8.4 Hz, 2H), 7.24 (d, *J* = 8.4 Hz, 2H), 7.33 (d, *J* = 8.4 Hz, 2H), 7.38 (s, 1H). ^13^C NMR (100 MHz, CDCl_3_, δ ppm): 53.03, 70.00, 79.79, 119.80, 128.03, 128.64, 128.91, 129.07, 129.17, 133.62, 134.59, 135.65, 136.35, 137.80. Anal. (% calcd/found) for C_18_H_16_Cl_2_N_2_O (MW: 347.24) C, 62.26/62.12; H, 4.64/4.51; N, 8.07/7.98. MS (ESI-*m*/*z*): 347.05 (M^+^, 100).

#### 2.4.15 1-(2-(4-Chlorophenyl)-2-((4-methylbenzyl)oxy)ethyl)-1*H*-imidazole (**21**)

Yellowish oil, yield: 0.47 g (68%), *R*_f_ = 0.61 (CHCl_3_–CH_3_OH, 10:1). FT-IR (ATR, cm^–1^): 3110 (Ar–CH), 2923 (Aliph. CH), 1597 (C=N), 1086 (ROR). ^1^H NMR (400 MHz, CDCl_3_): δ (ppm) 2.32 (s, 3H), 4.02–4.18 (m, 3H), 4.40–4.51 (m, 2H), 6.85 (s, 1H), 7.01 (t, *J* = 6.0 Hz, 3H), 7.11 (d, *J* = 7.6 Hz, 2H), 7.17 (d, *J* = 8.0 Hz, 2H), 7.33 (d, *J* = 8.4 Hz, 2H), 7.37 (s, 1H). ^13^C NMR (100 MHz, CDCl_3_, δ ppm): 21.15, 53.11, 70.70, 79.45, 119.89, 127.79, 128.08, 128.94, 129.08, 129.18, 134.08, 134.40, 136.73, 137.66, 137.80. Anal. (% calcd/found) for C_19_H_19_ClN_2_O (MW: 326.82) C, 69.83/69.71; H, 5.86/5.73; N, 8.57/8.48. MS (ESI-*m*/*z*): 327.10 (M + 1, 100).

#### 2.4.16 1-(2-(Benzyloxy)-2-(4-methoxyphenyl)ethyl)-1*H*-imidazole (**22**)

Yellowish oil, yield: 0.53 g (81%), *R*_f_ = 0.63 (CHCl_3_–CH_3_OH, 10:1). FT-IR (ATR, cm^–1^): 3110 (Ar–CH), 2934 (Aliph. CH), 1610 (C=N), 1092 (ROR). ^1^H NMR (400 MHz, CDCl_3_): δ (ppm) 3.80 (s, 3H), 4.01–4.06 (m, 1H), 4.11–4.21 (m, 2H), 4.45 (d, *J* = 11.6 Hz, 2H), 6.89 (t, *J* = 8.4 Hz, 3H), 7.00 (s, 1H), 7.14 (q, *J* = 7.8 Hz, 4H), 7.29 (t, *J* = 7.2 Hz, 3H), 7.39 (s, 1H). ^13^C NMR (100 MHz, CDCl_3_, δ ppm): 53.33, 55.27, 70.42, 79.94, 114.24, 119.91, 127.63, 127.71, 127.99, 128.42, 128.80, 129.98, 137.59, 137.83, 159.83. Anal. (% calcd/found) for C_19_H_20_N_2_O_2_ (MW: 308.37) C, 74.00/74.12; H, 6.54/6.43; N, 9.08/9.00. MS (ESI-*m*/*z*): 309.11 (M + 1, 100).

#### 2.4.17 1-(2-((4-Chlorobenzyl)oxy)-2-(4-methoxyphenyl)ethyl)-1*H*-imidazole (**23**)

White solid, yield: 0.51 g (71%), *R*_f_ = 0.45 (CHCl_3_–CH_3_OH, 10:1), mp 104–105 °C (CHCl_3_). FT-IR (ATR, cm^–1^): 3120 (Ar–CH), 2940 (Aliph. CH), 1608 (C=N), 1090 (ROR). ^1^H NMR (400 MHz, CDCl_3_): δ (ppm): 3.82 (s, 3H), 4.03–4.07 (m, 1H), 4.12–4.17 (m, 2H), 4.38–4.45 (m, 2H), 6.86–6.92 (m, 3H), 7.00–7.05 (m, 3H), 7.16 (t, *J* = 6.4 Hz, 2H), 7.25 (t, *J* = 6.6 Hz, 2H), 7.40 (d, *J* = 4.4 Hz, 1H). ^13^C NMR (100 MHz, CDCl_3_, δ ppm): 53.29, 55.30, 69.62, 80.08, 114.30, 119.83, 127.96, 128.57, 128.92, 129.70, 133.44, 136.08, 137.84, 159.91. Anal. (% calcd/found) for C_19_H_19_ClN_2_O_2_ (MW: 342.82) C, 66.57/66.42; H, 5.59/5.46; N, 8.17/8.08. MS (ESI-*m*/*z*): 343.06 (M + 1, 100).

#### 2.4.18 1-(2-(4-Methoxyphenyl)-2-((4-methylbenzyl)oxy)ethyl)-1*H*-imidazole (**24**)

Yellowish oil, yield: 0.51 g (75%), *R*_f_ = 0.50 (CHCl_3_–CH_3_OH, 10:1). FT-IR (ATR, cm^–1^): 3110 (Ar–CH), 2933 (Aliph. CH), 1611 (C=N), 1089 (ROR). ^1^H NMR (400 MHz, CDCl_3_): δ (ppm) 2.31 (s, 3H), 3.80 (s, 3H), 4.00–4.04 (m, 1H), 4.10–4.16 (m, 2H), 4.39–4.47 (m, 2H), 6.88 (t, *J* = 8.0 Hz, 3H), 7.01 (t, *J* = 7.8 Hz, 3H), 7.10 (d, *J* = 7.2 Hz, 2H), 7.16 (d, *J* = 8.4 Hz, 2H), 7.38 (s, 1H). ^13^C NMR (100 MHz, CDCl_3_, δ ppm): 21.15, 53.35, 55.26, 70.28, 79.73, 114.21, 119.93, 127.78, 127.99, 128.73, 129.10, 130.08, 134.50, 137.44, 137.81, 159.78. Anal. (% calcd/found) for C_20_H_22_N_2_O_2_ (MW: 322.40) C, 74.51/74.41; H, 6.88/6.73; N, 8.69/8.54. MS (ESI-*m*/*z*): 323.18 (M + 1, 100).

### 2.5 Anticancer Activity

#### 2.5.1 Cell Culture

The steps for cell culture were also mentioned in the previous study.^47^ Human breast adenocarcinoma cell line (MCF7), rat glial tumor cell line (C6), healthy mouse fibroblast cell line (L929), and colon cancer (HT29) cell line were purchased from ATCC (American type culture collection) and studied. Cells were mixed with 89% DMEM (Dulbecco’s modified Eagle’s medium; Gibco, Thermo Fisher Scientific), 10% FBS (fetal bovine serum; Sigma-Aldrich), and 1% penicillin (Sigma-Aldrich) solutions. The cells in which the medium was added were allowed to grow by incubating at 37 °C in an environment containing 95% humidity and 5% CO_2_.

#### 2.5.2 Cell Viability Assay

The cytotoxic effects of all compounds by MTT analysis on L929, HT29, MCF7, and C6 cell lines were investigated. 96-well plates were used for seeding cells. Approximately 1 × 10^4^ cells were seeded in each well. Cells were allowed to adhere for 24 h, and then the compounds were applied at different concentrations. All compounds were run in three repetitions. Wells with a maximum of 100 μM compounds were incubated for 24 h. Three wells without compounds treated with only DMSO (with total volume of % 0.5) were used as negative controls, and cisplatin was used as a positive control. After the incubation, the wells were treated with MTT solution to determine metabolically active cells and incubated at 37 °C for 3 h. After the MTT interaction, the FRAP wells were emptied, and DMSO solution was placed in them. The formazan crystals formed were dissolved with this solution, and the number of viable cells in each well was determined by color change. The absorbance values were read at 540 nm with the help of a microplate, and the values found were represented as mean ± standard deviation (±SD).

#### 2.5.3 Annexin V Binding Assay

Approximately 5 × 10^5^ seeds of each cancer cell were seeded into six-well plates and allowed to adhere overnight. The next day, compounds (**3a** and **3b**) found to be most effective for C6 and HT-29 cancer cells were incubated at IC_50_ doses for another 24 h. Cells harvested after trypsinization were suspended in PBS containing at least 1% FBS. The manufacturer’s instructions were then followed, and the Annexin V and dead cell reagent were mixed with the cells. Afterward, the percentage of dead, viable, early and late apoptotic cells was determined using the muse cell analyzer (Millipore) device.

#### 2.5.4 Cell Cycle Assay

DNA content (cell cycle) analyses were performed with the MUSE flow cytometry device. When the IC_50_ results obtained were evaluated, compounds **3a** and **3b** caused selective toxicity in the HT29 and C6 cancer cell lines compared to L929, respectively. Therefore, the main pathways of compounds **3a** and **3b** in cells were determined with the MUSE (cell cycle) kit.

### 2.6 Antioxidant Activity

The FRAP method was used to determine the antioxidant power of compounds. This method, which determines the reducing power of compounds, was developed by Benzie and Strain.^48^ Fe(III)–TPTZ complex is formed as a result of the reaction of Fe(III) with tripyridyltriazine (TPTZ), and this complex is reduced to the Fe(II)–TPTZ complex with the antioxidant in the environment. The color of this complex is dark blue and gives the maximum absorbance at 593 nm.^48^ Incubation is performed for up to 30 min to complete the reaction, and the correct absorbance values were read. The FRAP values of the samples were calculated as “μmol trolox equiv/g sample” with the help of the standard curve obtained using methanol–trolox standard solutions (0; 0.2; 0.4; 0.6; 0.8; and 1.0 μmol/mL) which were used as the positive control.^49^

### 2.7 Investigation of the Synthesized Compounds on CA Isoenzymes Using the Esterase Activity Method

The method used to spectrophotometrically detect the antiepileptic, antiglaucoma, and antidiuretic effects of the obtained compounds in vitro is the esterase activity method. This method relies on the esterase activity of CA for the hydrolysis of *p*-nitrophenyl acetate as a substrate, which is a part of the reaction mechanism involving *p*-nitrophenol or *p*-nitrophenolate. Both *p*-nitrophenol and *p*-nitrophenolate exhibit the same absorbance at 348 nm.^50,51^

### 2.8 Computational Studies

#### 2.8.1 DFT Calculations

DFT calculations were performed using the B3LYP (Becke’s three-parameter exchange functional combined with the Lee, Yang, and Parr correlation functional) method, 6-31+G(d,p) basis set, and IEFPCM solvation model. Gaussian 09 Rev. D01^52^ and GaussView 5^53^ software packages were used in the calculations. Discovery Studio Visualizer^54^ was used to visualize the results.

#### 2.8.2 Molecular Docking Studies

In molecular docking studies, AutoDock Tools^55^ and AutoDock Vina^56^ were used. Discovery Studio Visualizer was used in the visualization of the results. The structures of EGFR, VEGFR2, FGFR1, HSP90, hCA I, and hCA II were obtained from RCSB Protein Data Bank (PDB ID: 2J6M, 4AG8, 4V04, 1YET, 1CZM and 3HS4).^57−63^ Three-dimensional structures of the investigated compounds were obtained from DFT calculations. Prior to molecular docking, water and bound ligands were removed, hydrogens were added, and nonpolar hydrogens were merged and Gasteiger charges were added. Molecular docking was carried out in a 24 × 24 × 24 Å^3^ grid box covering the active site of the receptor.

#### 2.8.3 Drug-Likeness and ADME Analyses

Drug-likeness and ADME analyses were carried out with the assistance of SwissADME web server.^64^ The structures were submitted to the web server as a SMILES string. In this part of the study, physicochemical properties, lipophilicity, water solubility, pharmacokinetics, drug-likeness, and properties related to medicinal chemistry were estimated.

## 3 Results and Discussion

### 3.1 Chemistry

In this study, three ketones (**3a**–**c**), three alcohols (**4a**–**c**), nine alkyl ether derivatives (**7**–**15**), and nine aryl ether derivatives (**16**–**24**) containing substituted phenyl and imidazole rings were synthesized via the synthetic routes shown in Scheme 1, and the structures of these compounds were characterized using ^1^H NMR, ^13^C NMR, FT-IR, elemental analysis, and mass spectroscopy techniques.

In the first step, ketone derivatives (**3a**–**c**) were synthesized with varying yields between 51 and 62% by the reaction of imidazole (**2**) with various phenacyl bromide derivatives (**1a**–**c**) in the presence of triethylamine, as described in the literature.^65,66^ In this reaction, the proton of imidazole is removed by triethylamine in an acetone medium. Subsequently, a nucleophilic center, with a partially positive carbon atom, undergoes an SN_2_-type substitution reaction by attack from behind.

The most significant evidence for the formation of these compounds (**3a**–**c**) in the FT-IR spectra is the observation of carbonyl group (C=O) absorption bands in the range of 1692–1680 cm^–1^. Additionally, the presence of aromatic –CH stretching vibrations in the range of 3131–3120 cm^–1^, aliphatic –CH stretching vibrations in the range of 2963–2933 cm^–1^, and azomethine group (–C=N) stretching bands in the range of 1604–1589 cm^–1^ further supports the structures completely.

In the ^1^H NMR spectra of these compounds, peaks corresponding to a methylene (−CH_2_) group, observed as a singlet, were observed in the range of 5.35–5.28 ppm, which correspond to two protons. Additionally, peaks attributed to the phenyl and imidazole groups in these compounds were observed in the range of 7.90–6.87 ppm. These values, consistent with literature data, confirm their structures.^67^ In the ^13^C NMR spectra of the synthesized compounds (**3a**–**c**), characteristic peaks for the carbonyl carbon (C=O) were observed in the range of 191.86–190.18 ppm, and peaks for aromatic groups were observed in the range of 140.91–114.22 ppm, supporting the proposed structures.^68^ Finally, the molecular ion peaks observed at 186.90, 220.80, and 216.81 in the mass spectrum have completely confirmed the structures of compounds **3a**, **3b**, and **3c**, respectively.

In the second step of the synthesis, compounds (**4a**–**c**) were obtained as secondary alcohol derivatives through the reduction of ketone derivatives (**3a**–**c**) obtained in the first step with sodium borohydride (NaBH_4_) in absolute ethanol, yielding efficiency between 75 and 85%.

In the FT-IR spectra of these compounds (**4a**–**c**), the most significant evidence for the formation of secondary alcohol derivatives is the disappearance of the carbonyl group (C=O) absorption bands in the range of 1692–1680 cm^–1^ and the appearance of broad –OH stretching bands in the range of 3123–3115 cm^–1^. This condition in the FT-IR spectra demonstrates the reduction of the carbonyl group to a secondary alcohol.

There are two important indicators in the ^1^H NMR spectra of these compounds that suggest the presence of secondary alcohols. The first one is the broad singlet observed in the range of 5.82–5.61 ppm, which corresponds to one proton and is associated with the –OH protons. The other one is the peaks observed in the range of 4.83–4.75 ppm, which correspond to one proton belonging to the –CH group that is bonded to the –OH group. The disappearance of the peaks observed in the 4.83–4.75 ppm range upon proton–deuterium exchange indicates that these peaks are associated with the –OH group.

In the ^13^C NMR spectra of these compounds (**4a**–**c**), characteristic peaks belonging to the carbonyl carbon (C=O) in the range of 191.86–190.18 ppm disappeared, and, instead, new peaks associated with the carbon bonded to the –OH group in the range of 72.49–71.72 ppm have been observed. This change observed in the ^13^C NMR spectra indicates a specific case where the carbonyl group has been reduced to a secondary alcohol.^69,70^ Additionally, in the mass spectra, molecular ion peaks at 190.83, 222.83, and 218.84 for compounds **4a**, **4b**, and **4c**, respectively, have fully confirmed their structures.

In the third and final stage of the synthesis, the target compounds, alkyl (**7**–**15**) and aryl ether derivatives (**16**–**24**), were obtained with yields ranging from 51 to 89% through the reactions of these alcohol compounds (**4a**–**c**) in the presence of sodium hydride (NaH) in DMF with various alkyl (**5a**–**c**) and aryl (**6a**–**c**) halides.

The disappearance of the broad –OH stretching bands in the FT-IR spectra of target compounds (**7**–**15**, **16**–**24**) in the range of 3123–3115 cm^–1^ and the observation of specific ROR stretching bands belonging to ether derivatives in the range of 1099–1075 cm^–1^ are important evidence of the formation of these compounds.

The most significant evidence indicating the formation of the suggested compounds in the ^1^H NMR spectra of these compounds is the disappearance of the –OH proton peaks observed as a broad singlet in the range of 4.83–4.75 ppm and the increase in peak intensity in the aliphatic region (1–5 ppm range) for compounds **7**–**15** and in the aromatic region (6.5–8.00 ppm) for compounds **16**–**24**. These data are in excellent agreement with similar studies in the literature.^65−67,71^

The carbon peaks observed in the aliphatic region (60–10 ppm range) for compounds **7**–**15** and in the aromatic region (140–115 ppm range) for compounds **16**–**24** in the ^13^C NMR spectra of the target compounds match the carbon counts of the proposed structures. Finally, the molecular ion peaks of the target compounds were observed as expected in the mass spectra, confirming the structures using all the spectroscopic methods used.

The ^1^H NMR, ^13^C NMR, FT-IR, elemental analysis, and mass spectroscopy data for all compounds synthesized in this study are available in the Experimental Section. All of the spectra are provided in the Supporting Information section.

### 3.2 Biological Activity Studies

#### 3.2.1 Anticancer Activity

**Figure 2:**
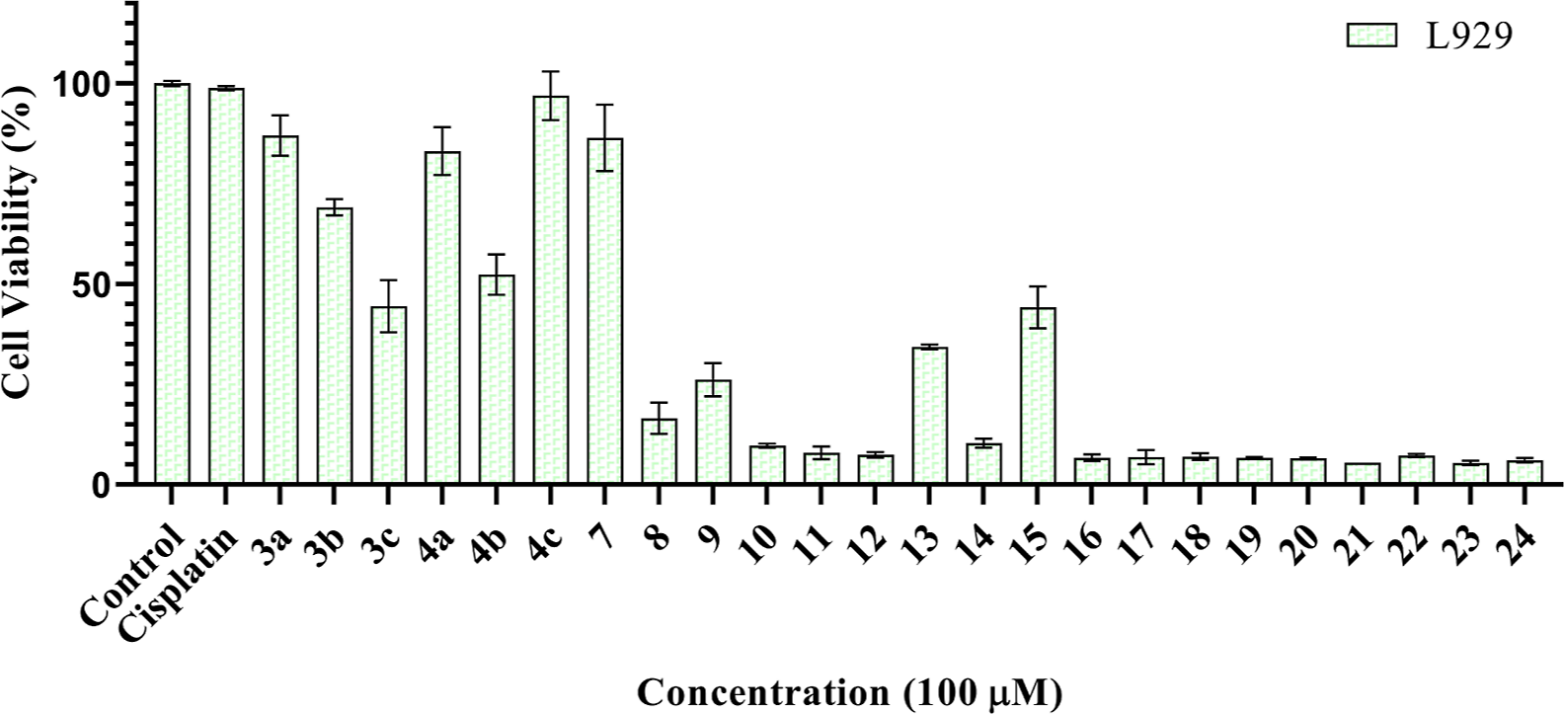
After 24 h of incubation, cell viability of all compounds on the L929 mouse fibroblast cell line at 100 μM.

The compounds were first applied to L929 mouse fibroblast cells at a dose of 100 μM in triplicate for 24 h, and cell viability was measured via the MTT method. With the results obtained, it was determined which compounds were toxic and which were nontoxic. It was observed that the compounds **3a**, **3b**, **4a**, **4b**, **4c**, and **7** were not toxic in the L929 mouse fibroblast healthy cell line, and anticancer studies continued to be carried out on these compounds. The cell viability graph as a result of the study performed on a healthy cell line is shown in Figure 2.

**Figure 3:**
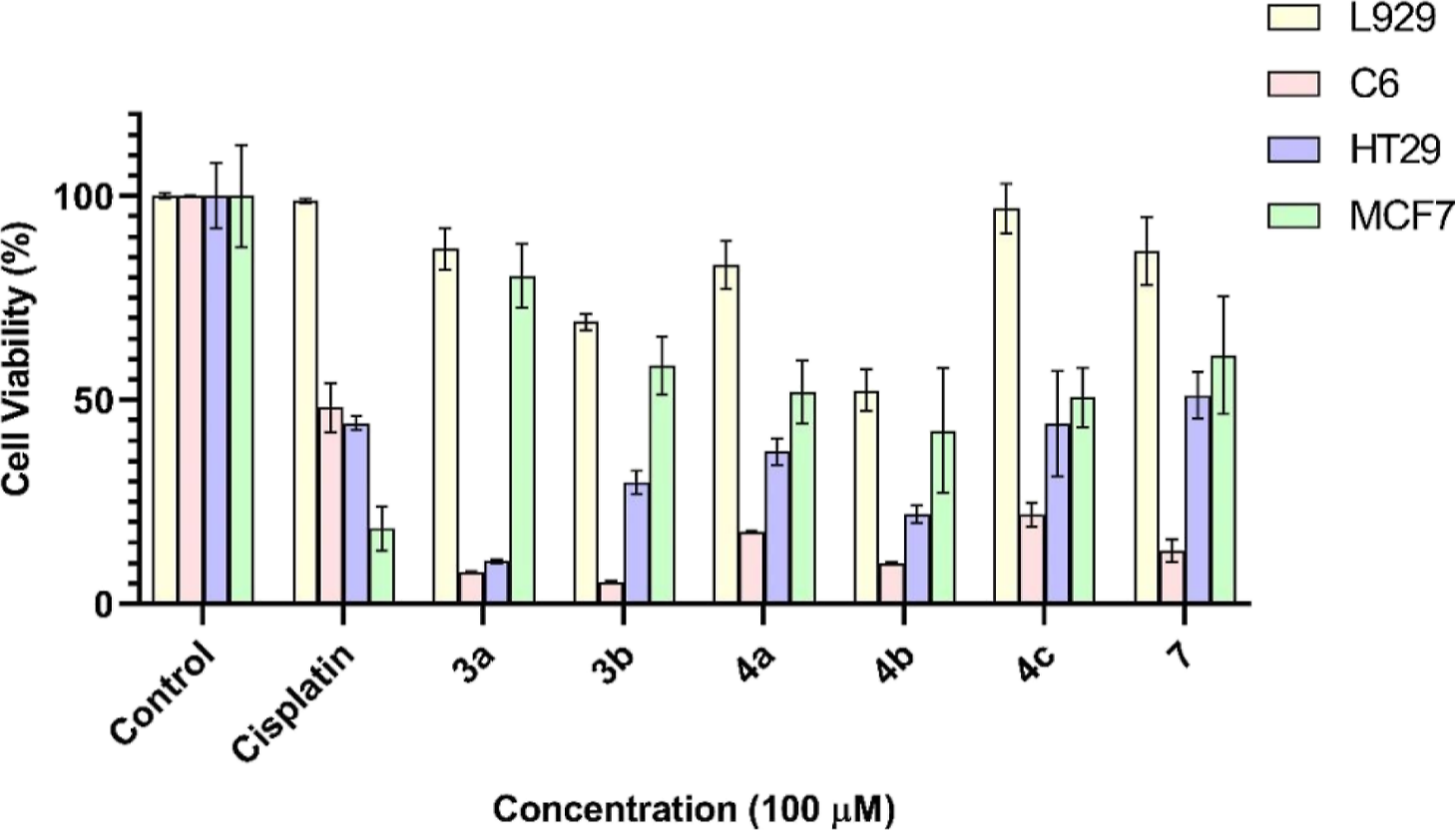
After 24 h of incubation, cell viability of compounds **3a**, **3b**, **4a**, **4b**, **4c**, and **7** on cancer cell lines at 100 μM.

As a result of studies conducted on MCF7, C6, and HT29 cell lines, it has been observed that some structures provide more effective results than the drug cisplatin, which is currently used in cancer treatment. All six compounds studied were more effective than cisplatin on the C6 cell line. Compound **3b** was found to be the most effective with an IC_50_ value of 10.721 ± 0.38 μM. The IC_50_ value of cisplatin for the C6 cell line was calculated as 88.24 ± 8.12 μM. In colon cancer, compounds **3a**, **3b**, **4a**, and **4b** were determined to be more toxic than cisplatin. Compound **3a** was found to be the most effective compound on colon cancer with an IC_50_ value of 20.88 ± 1.02 μM. The IC_50_ value of cisplatin on colon cancer was calculated as 68.23 ± 3.4 μM. IC_50_ values of the six selected compounds are shown in Table 1. In cytotoxic studies on breast cancer, no compound was found to be more toxic than cisplatin. Cell viabilities determined as a result of the maximum dose (100 μM) of the six compounds determined on cancer cells are shown in Figure 3. The IC_50_ values of cisplatin found for the mentioned cell lines correlate with the results obtained in previously published articles.^47,72^

**Table 1:** After 24 h of Incubation, IC_50_ (μM) Values of Compounds **3a**, **3b**, **4a**, **4b**, **4c**, and **7** on all Cell Lines

comp.	L929	C6	HT29	MCF7
cisplatin	277.70 ± 1.09	88.24 ± 12.13	68.23 ± 3.39	26.81 ± 10.95
**3a**	174.05 ± 10.11	15.76 ± 0.08	20.88 ± 1.02	160.90 ± 15.51
**3b**	138.26 ± 4.06	10.72 ± 0.38	59.63 ± 5.73	116.81 ± 14.45
**4a**	166.26 ± 11.91	35.22 ± 0.55	74.62 ± 6.67	103.83 ± 15.35
**4b**	104.77 ± 10.10	20.09 ± 0.32	44.12 ± 4.27	84.91 ± 21.54
**4c**	193.89 ± 12.19	43.78 ± 5.83	88.40 ± 15.7	101.33 ± 14.54
**7**	172.92 ± 16.56	26.08 ± 5.70	102.33 ± 11.52	121.81 ± 28.82

#### 3.2.2 Flow Cytometry

**Figure 4:**
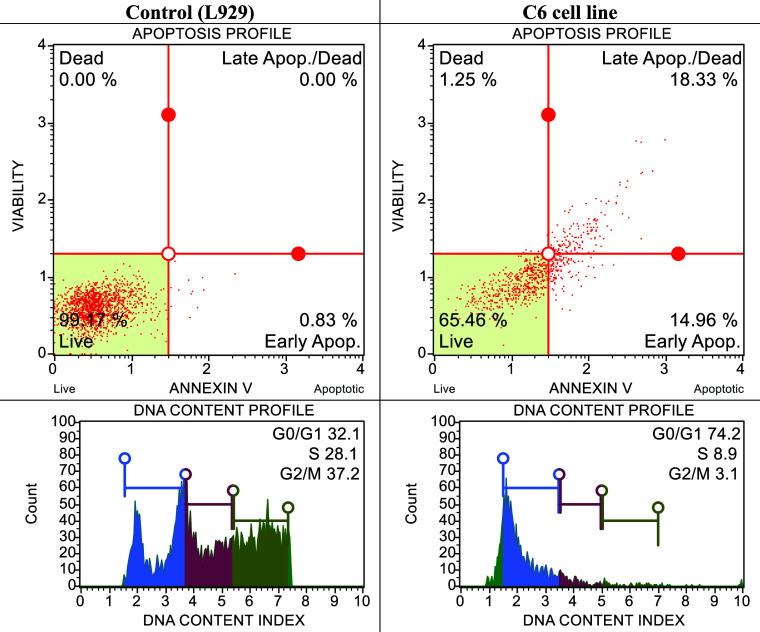
After 24 h of incubation, apoptotic and cell cycle consequences of compound **3b** on healthy mouse fibroblast (L929) and cancer cells (C6) with IC_50_ dose (10.72 ± 0.38 μM).

**Figure 5:**
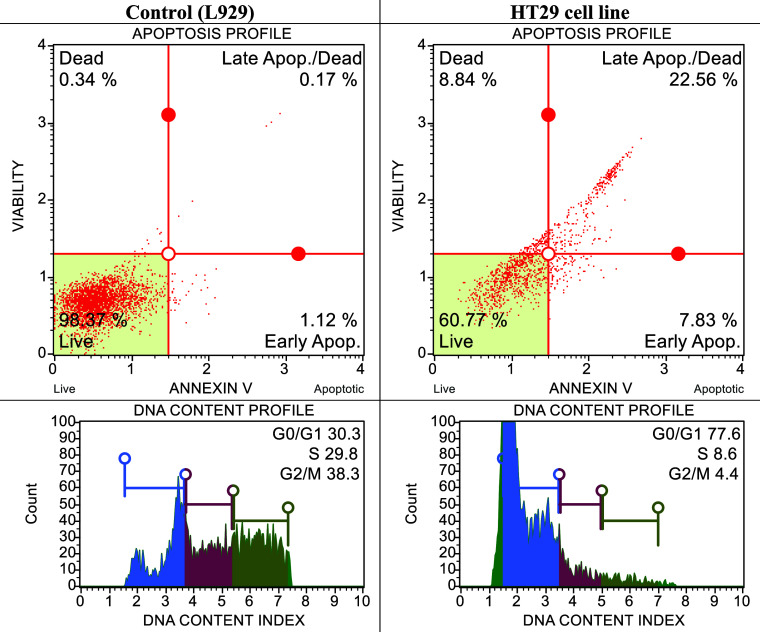
After 24 h of incubation, apoptotic and cell cycle consequences of compound **3a** on healthy mouse fibroblast (L929) and cancer cells (HT29) with IC_50_ dose (20.88 ± 1.02 μM).

With the flow cytometry studies described previously,^73^ the two most active treatments on cancer cells (**3a** and **3b**) were listed, how they were directed to apoptosis with Annexin V dye and where the cell cycle was stopped with the necessary kit. The results obtained are shown in Figures 4 and 5.

When the IC_50_ dose of compound **3b** for the glioblastoma cell line was applied to healthy and cancerous cells, the viability percentage in the healthy cell remained at 99.17%, while the viability percentage in the cancerous cell was found to be 65.46%. Additionally, it was determined that 18.33% progressed to late apoptosis and 14.96% progressed to early apoptosis. However, in cell cycle studies, it was determined that cells stopped in the G0/G1 phase after drug application. When the IC_50_ dose of compound **3a** for the colon cancer cell line was applied to healthy and cancerous cells, the viability percentage in the healthy cell remained at 98.37%, while the viability percentage in the cancerous cell was found to be 60.77%. It was also determined that 22.56% progressed to late apoptosis and 7.83% progressed to early apoptosis. However, in cell cycle studies, it was determined that the cells stopped in the G0/G1 phase after drug administration.

#### 3.2.3 Antioxidant Activity

Like some reducing agents, antioxidants also cause the Fe^3+^ ferricyanide complex to be reduced to Fe^2+^. In this method, the color of the test solution changes from yellow to green, depending on the reducing power of the sample tested. This green color gives maximum absorbance at 700 nm, and increasing absorbance indicates increasing reduction strength. According to this method, trolox was used as the standard antioxidant compound, and measurements were made in accordance with the procedure determined by Benzie and Strain.^48^ The results found are shown in Table 2. No compound has an antioxidant power as active as vitamin E. The synthesized imidazole derivates are not likely to be free-radical scavengers based on their structures. It is more likely that their antioxidant or pro-oxidative action, if any, is indirect, for example, through the inhibition of relevant proteins.

**Table 2:** FRAP (μmol Trolox equiv/g) Values of All Compounds

compounds	μmol trolox equiv/g	compounds	μmol trolox equiv/g
**3a**	0.18 ± 0.07	**13**	0.15 ± 0.06
**3b**	0.20 ± 0.04	**14**	0.28 ± 0.01
**3c**	0.21 ± 0.03	**15**	0.31 ± 0.03
**4a**	0.31 ± 0.01	**16**	0.41 ± 0.03
**4b**	0.25 ± 0.02	**17**	0.35 ± 0.07
**4c**	0.29 ± 0.05	**18**	0.30 ± 0.04
**7**	0.10 ± 0.08	**19**	0.27 ± 0.01
**8**	0.19 ± 0.02	**20**	0.31 ± 0.06
**9**	0.12 ± 0.01	**212**	0.25 ± 0.01
**10**	0.20 ± 0.03	**22**	0.40 ± 0.05
**11**	0.31 ± 0.07	**23**	0.45 ± 0.04
**12**	0.28 ± 0.04	**24**	0.46 ± 0.06
		vitamin E	1.00 ± 0.04

#### 3.2.4 CA I/II Inhibition Assay

The effects of newly synthesized imidazole compounds against CA I and II isoenzymes were examined spectrophotometrically. Accordingly, while the compounds (**3a**–**c** and **4a**–**c**) did not show a significant effect on the activity, it was observed that the compounds **(7–18)** increased the enzyme activities (Table S3 and Figure S113). The remaining molecules were found to reduce the enzyme activity and have inhibitory potential.

**Figure 6:**
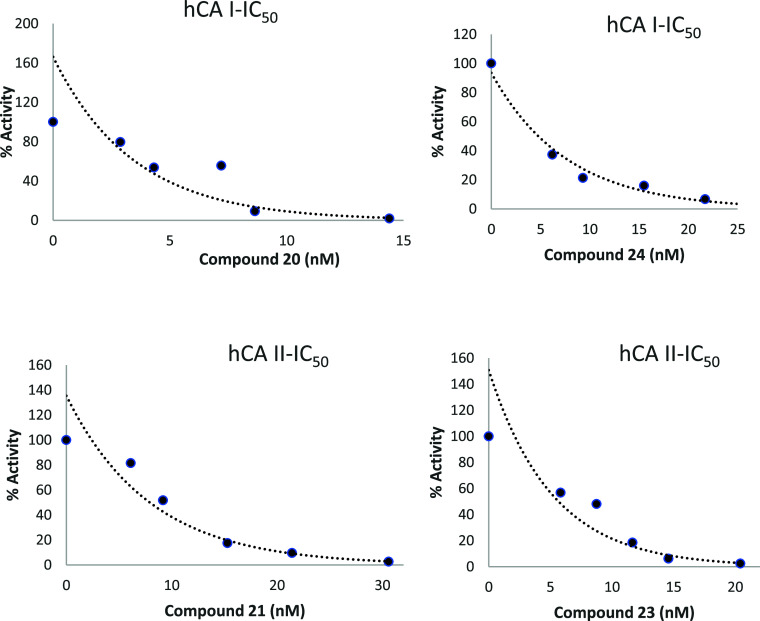
% Activity–inhibitor concentration graphs of the best inhibiting compounds.

The synthesized compounds (**17, 19–24**) exhibited generally inhibition profiles against the widespread cytosolic hCA I isozyme with IC_50_ values ranging from 4.13 to 15.67 nM and cytosolic hCA II isozyme with IC_50_ values ranging from 5.65 to 14.84 nM. Percent activity–inhibitor concentration graphs of the best inhibiting molecules are given in Figure 6. The inhibition potentials of these molecules are better than that of the standard acetazolamide (AAZ) substance.

The most active compound **20** showed IC_50_ values of 4.13 nM on hCA I (Figure 6). For hCA I, IC_50_ values of acetazolamide (AAZ) as the positive control and the imidazole derivatives (**17, 19–24**) were studied in this study in the following order: compound **20** (4.13 nM, *r*^2^: 0.875) < compound **24** (4.77 nM, *r*^2^: 0.979) < compound **23** (7.494 nM, *r*^2^: 0.942) < compound **21** (9.83 nM, *r*^2^: 0.897) < compound **17** (15.67 nM, *r*^2^: 0.852) < compound **19** (15.67 nM, *r*^2^: 0.820) < AAZ (15.58 nM, *r*^2^: 0.990).

The most active compound **23** showed IC_50_ values of 5.65 nM on hCA II (Figure 6). For hCA II, IC_50_ values of acetazolamide (AAZ) as the positive control and the imidazole derivatives (**17, 19–24**) were studied in this study in the following order: compound **23** (5.65 nM, *r*^2^: 0.932) < compound **21** (7.90 nM, *r*^2^: 0.977) < AAZ (8.37 nM, *r*^2^: 0.980) < compound **24** (9.66 nM, *r*^2^: 0.883) < compound **20** (10.91 nM, *r*^2^: 0.950) < compound **19** (11.74 nM, *r*^2^: 0.894) < compound **17** (14.84 nM, *r*^2^: 0.894) (Table 3).

**Table 3:** IC_50_ Values of Molecules That Inhibit hCA I and II Isoenzymes

compounds	IC_50_ (nM)			
	hCA I	*r*^2^	hCA II	*r*^2^
**17**	13.22	0.852	14.84	0.894
**19**	15.67	0.820	11.74	0.894
**20**	4.13	0.875	10.91	0.950
**21**	9.83	0.897	7.90	0.977
**23**	7.49	0.942	5.65	0.932
**24**	4.77	0.979	9.66	0.883
AAZ	15.58	0.990	8.37	0.980

When the compounds are examined structurally, it is seen that compounds **7**–**15**, which increase the activities of enzymes, contain ethyl (compounds **7**, **10**, and **13**), propyl (compounds **8**, **11**, and **14**), and allyl groups (compounds **9**, **12,** and **15**). Additionally, the presence of aryl groups (compounds **17**, **19**–**21**, **23**, and **24**) in the synthesized compounds with the inhibitory potential is noteworthy (Table 3).

### 3.3 Computational Studies

#### 3.3.1 DFT Calculations

In DFT calculations, geometry-optimized structures of the known compounds and novel compounds are obtained with the use of the B3LYP method, 6-31+G(d,p) basis set, and IEFPCM solvation model (water selected as the solvent). The optimized geometries of the novel compounds and the known compounds are given in Figures S100 and S101. Frequency analyses were also conducted in this section to confirm that the optimized geometric structures correspond to a minimum. The absence of imaginary frequencies in the analyses indicates that each structure corresponds to a minimum.

In this part of the study, molecular electrostatic potential maps of the compounds have also been calculated to determine the electron-rich and electron-deficient regions of the compounds under investigation. The calculated molecular electrostatic potential maps for the new compounds and known compounds are presented in Figures S102 and S103.

The results of molecular electrostatic potential map calculations show that negative charge was dominantly located on the electronegative oxygen and nitrogen atoms, and these negative centers act as hydrogen-bond acceptors in the ligand–receptor interactions in molecular docking studies.

#### 3.3.2 Molecular Docking Studies

Anticancer activity can occur through many different pathways/mechanisms. Inhibition of structures such as EGFR, VEGFR2, FGFR1, and HSP90 stand out with some of these methods. There are many studies in the literature investigating the activities of these targets by modulating/inhibiting them to determine the anticancer activity.^29,74−77^ In addition to anticancer activity, the compounds were examined for their potential to inhibit hCA I and hCA II enzymes. The investigation aimed to determine whether these compounds can interact with these targets and, if so, to explore the extent and manner of such interactions. The binding scores obtained from molecular docking calculations belonging to hCA I and hCA II are given in Table 4 while the results obtained for EGFR, VEGFR2, FGFR1, and HSP90 are given in Supporting Information (Table S1).

**Table 4:** Binding Scores Obtained from Molecular Docking Calculations

AutoDock Vina binding scores (kcal/mol)			AutoDock Vina binding scores (kcal/mol)		
compounds	hCA I	hCA II	compounds	hCA I	hCA II
**3a**	–5.80	–6.20	**14**	–5.70	–6.10
**3b**	–6.20	–6.00	**15**	–5.70	–6.10
**3c**	–6.10	–5.90	**16**	–7.10	–7.40
**4a**	–6.10	–6.50	**17**	–6.90	–7.60
**4b**	–6.40	–6.20	**18**	–6.90	–7.80
**4c**	–6.30	–6.00	**19**	–7.40	–7.40
**7**	–5.80	–6.40	**20**	–6.90	–7.40
**8**	–5.60	–6.40	**21**	–6.90	–7.30
**9**	–5.60	–6.60	**22**	–6.50	–7.00
**10**	–5.70	–5.90	**23**	–6.60	–7.10
**11**	–5.90	–6.00	**24**	–6.60	–7.30
**12**	–5.90	–6.00	acetazolamide	–5.50	–5.90
**13**	–5.70	–5.70			

**Figure 7:**
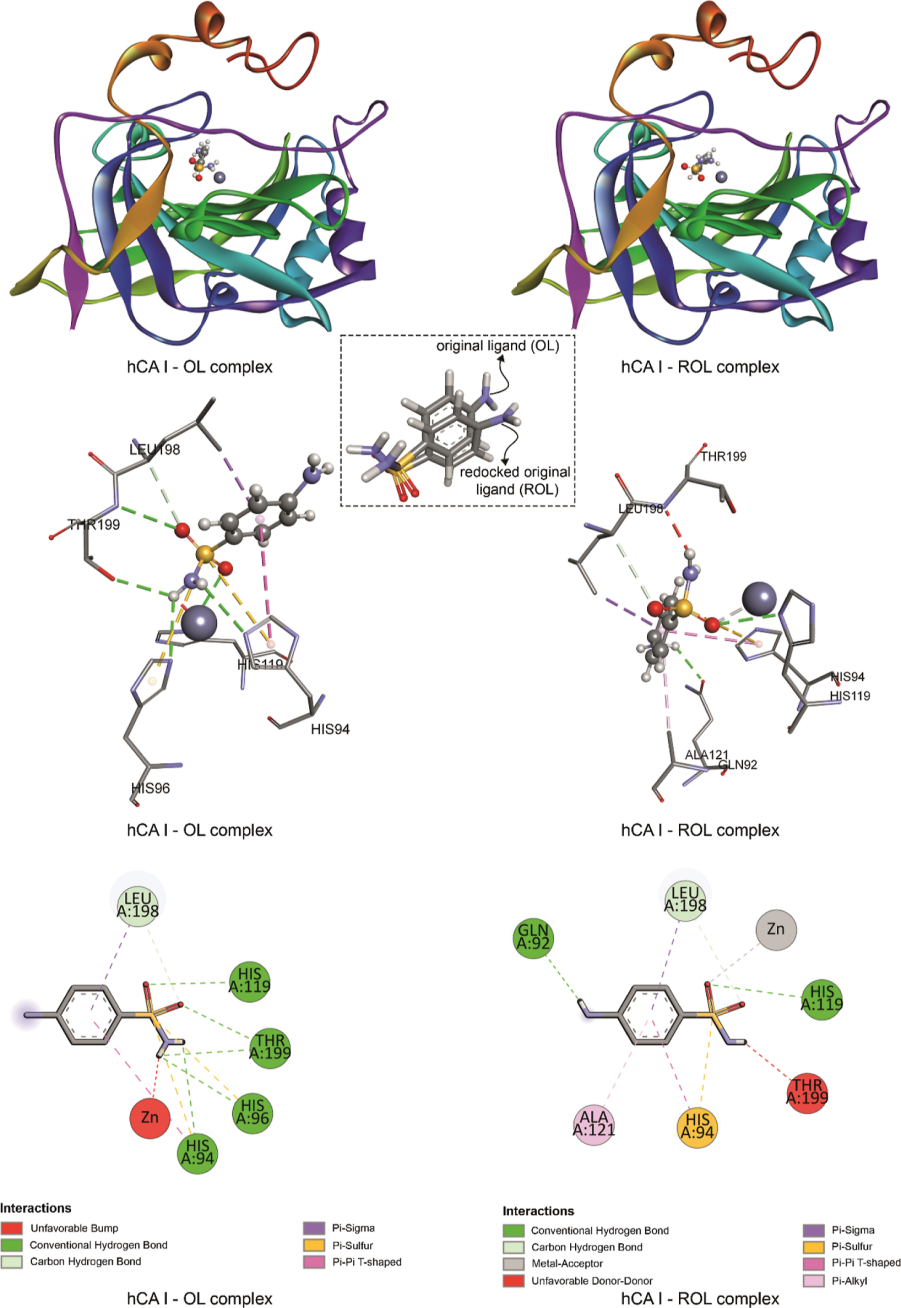
Binding poses and 2D and 3D representations of the interactions obtained for the hCA I—OL complex and hCA I—ROL complex (OL stands for the original ligand, which is the ligand molecule in the crystal structure; ROL stands for redocked original ligand which is the binding pose of the original ligand molecule obtained after redocking).

**Figure 8:**
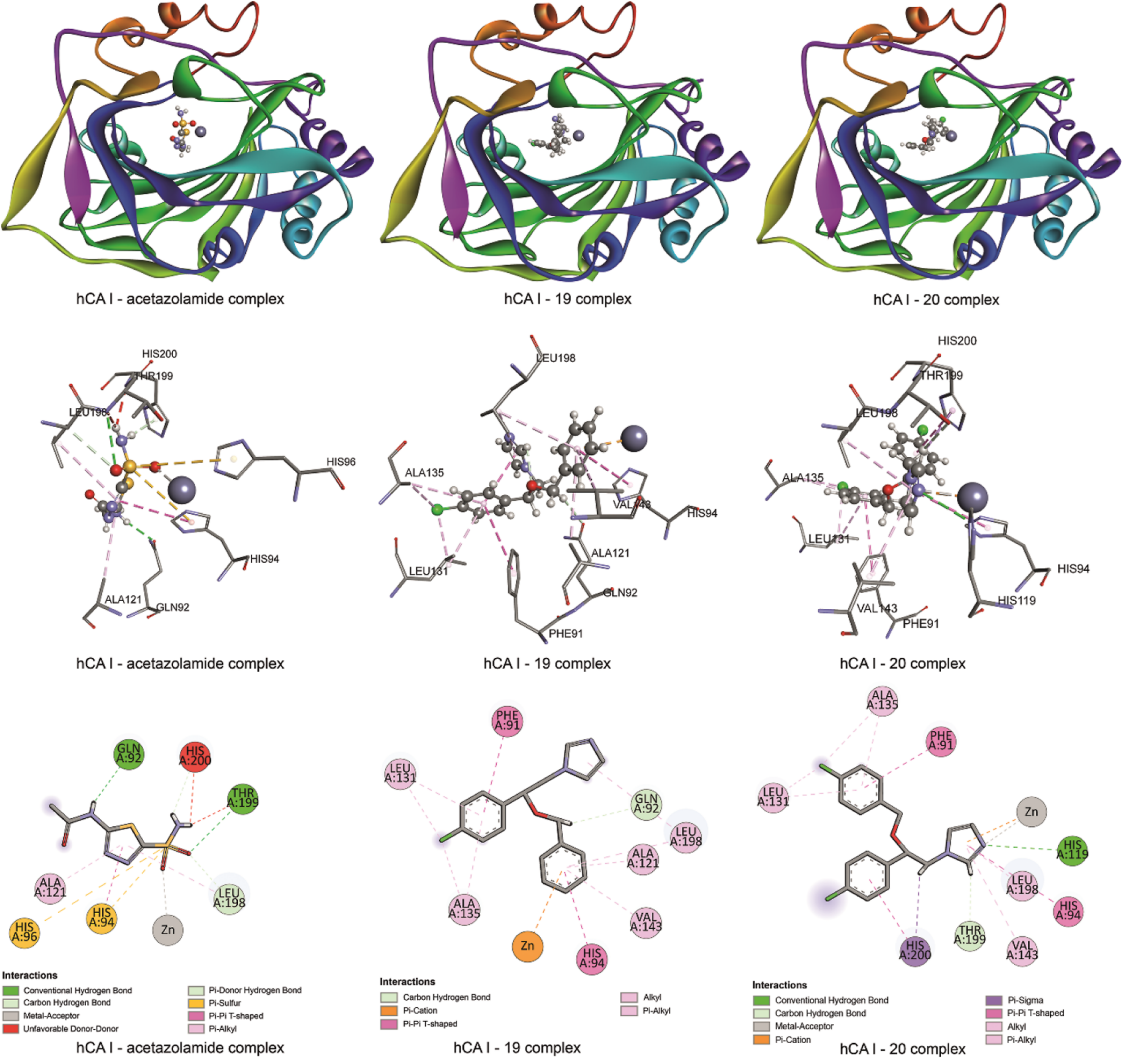
Binding poses and 2D and 3D representations of the interactions between hCA I and compounds **19** and **20**.

**Figure 9:**
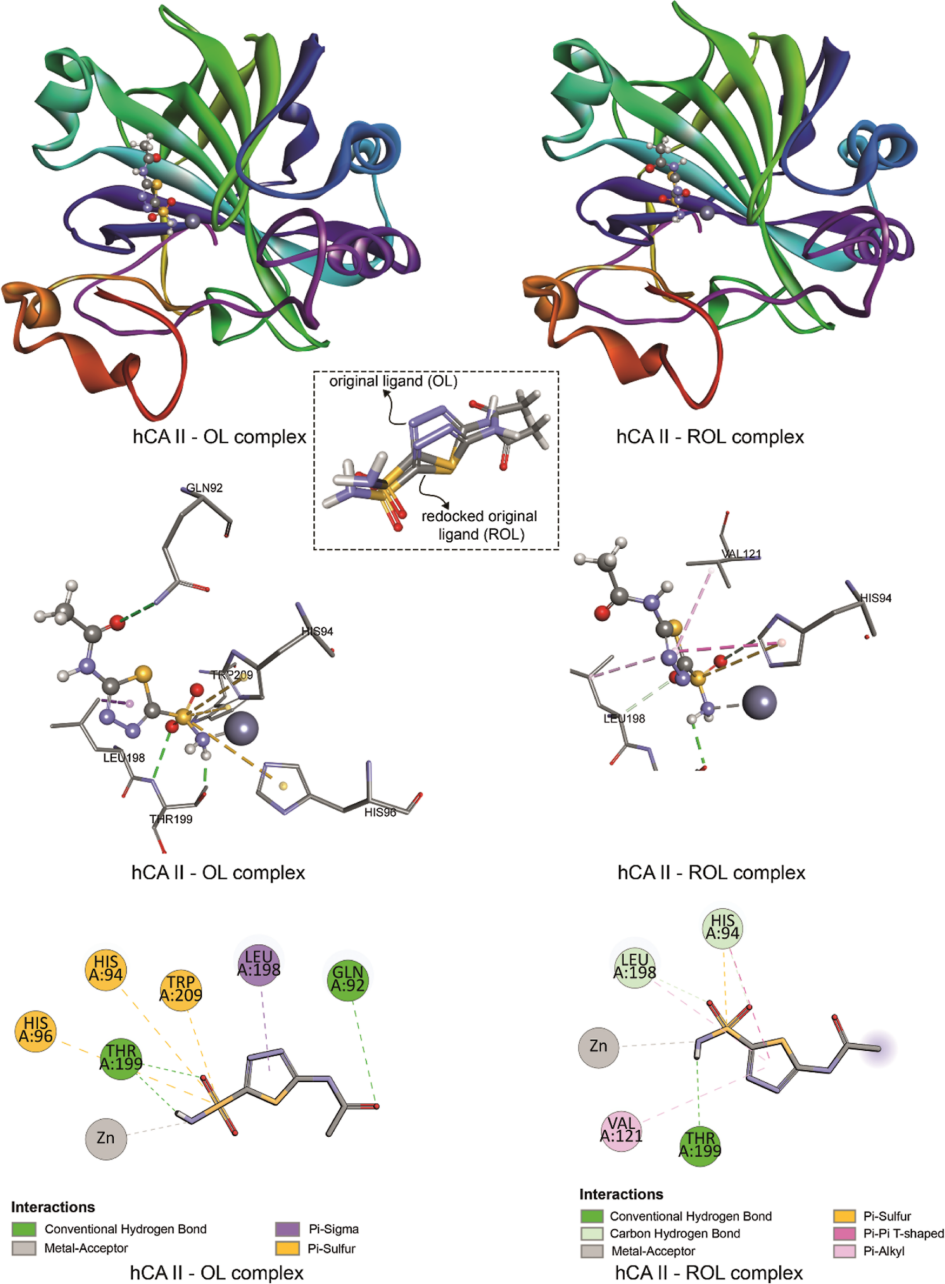
Binding poses and 2D and 3D representations of the interactions obtained for hCA II—OL complex and hCA II—ROL complex (OL: original ligand, which is the acetazolomide in the crystal structure; ROL: redocked original ligand which is the binding pose of acetazolamide obtained after redocking).

**Figure 10:**
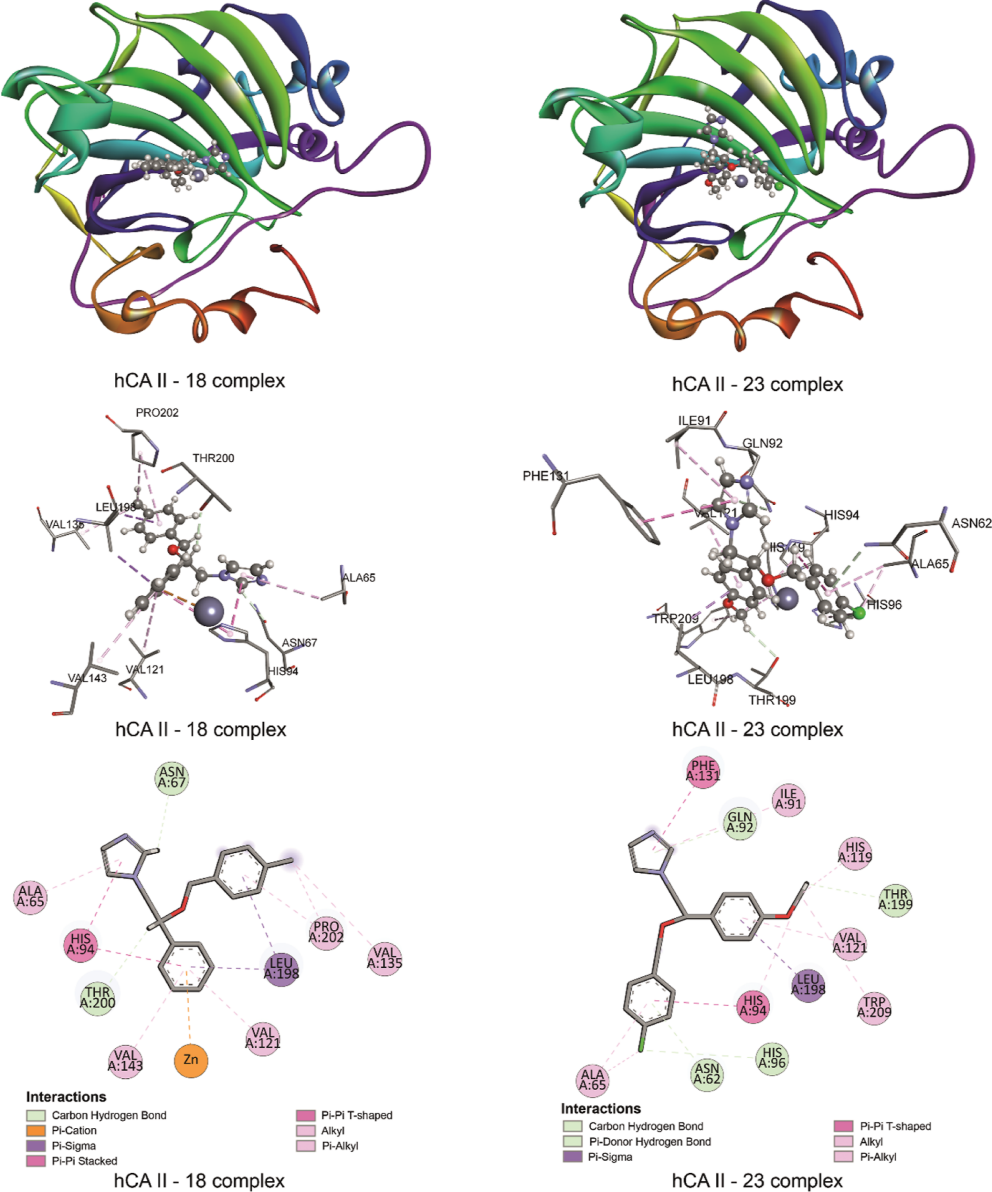
Binding poses and 2D and 3D representations of the interactions obtained for the hCA II—18 complex and hCA II—23 complex.

The molecular docking results indicate that the binding affinities of aryl ether derivatives (**16**–**24**) to hCA II are higher than that of the reference drug acetazolamide. Similar results were also obtained for hCA I. In addition to aryl ether derivatives (**16**–**24**), **3a**–**b** and **4a**–**c** have higher binding affinities than the reference drug acetazolamide. Ligand–receptor interactions belonging to hCA I—alkyl ethers, hCA I—aryl ethers, hCA II—alkyl ethers, and hCA II—aryl ethers are given in Figures S109–S112, respectively. Results showed that generally aryl ethers have a higher binding affinity to hCA I and hCA II than the corresponding alkyl ethers probably due to the presence of an extra aromatic ring in the structure, thus leading to additional interactions. However, unfortunately, none of the compounds exhibited a higher binding affinity to EGFR, VEGFR2, FGFR1, and HSP90 compared to the reference drugs. The results of the molecular docking calculations of Gefitinib and Geldanamycin were obtained from previous studies in which molecular docking calculation had been performed with the use of same parameters and procedures.^78,79^ The binding poses and ligand-receptor interactions of the highest scoring ligand-receptor complexes for hCA I and hCA II inhibition are shown in Figures 7–10. Additionally, inhibition of EGFR, VEGFR2, FGFR1, and HSP90 is provided in the Supporting Information (Figures S104–S108).

In Figure 7, binding pose and ligand–receptor interactions belonging to the complex consisting of hCA I and its original ligand existing in the crystal structure are given. Figure 7 also includes the binding pose obtained by redocking the original ligand and ligand–receptor interactions. The docking protocol was validated by redocking the original ligand in the crystal structure of hCA I obtained from RCSB PDB. The docking pose obtained after redocking shows a difference with an rmsd of 1.15 Å compared to its original position in the crystal structure. It was observed that in the original crystal structure of the hCA I—OL complex, metal–acceptor interaction could not be obtained; instead of this, an unfavorable bump was observed. In the hCA I—ROL complex, no unfavorable bump was observed, but an unfavorable donor–donor interaction was observed between the redocked pose of the original ligand and hCA I.

The molecular docking results indicate that compound **19** which has the highest binding affinity (−7.4 kcal/mol) to hCA I interacts with PHE91, GLN92, HIS94, ALA121, LEU131, ALA135, VAL143, and LEU198 residues of hCA I in addition to the zinc cation. It was observed that in addition to hydrogen bonding, π–cation, π–π T-shaped, alkyl, and π–alkyl interactions were formed between hCA I and compound **19**. The results also indicate that the interaction of the reference drug acetazolamide, whose binding score is −5.5 kcal/mol, with hCA I, GLN92, HIS94, HIS96, ALA121, LEU198, and THR199 residues in addition to zinc cation takes a role via hydrogen bonding, π–sulfur, π–π T-shaped, π–alkyl, and metal–acceptor interactions. Additionally, an unfavorable donor–donor interaction was also observed between HIS200 and the –NH_2_ group of acetazolamide. Figure 8 also shows the binding pose and ligand–receptor interactions belonging to compound **20** which has the highest inhibitory effect according to experimental data. Although molecular docking results show that the main difference between compound **19** and compound **20** is the different orientation in the binding site, and therefore, the phenyl group in compound **20** cannot interact with residues HIS94, VAL143, ALA121, and LEU198, including the zinc ion, experimental results show that compound **20** is the most effective compound against hCA I. The reason for this observed difference may be due to the orientation that compound **20** has under experimental conditions, similar to the molecular docking results of compound **19**, or it may be that compound **19** fails to stably maintain the binding pose suggested by molecular docking calculations under experimental conditions, resulting in a less inhibitory effect on hCA I than compound **20**.

Figure 9 shows the interactions between hCA II and the original ligand in the crystal structure of hCA II obtained from RCSB PDB and the interactions in the complex obtained by redocking of this ligand. The docking pose obtained after redocking shows a difference with an rmsd of 1.38 Å compared to its original position in the crystal structure, validating the docking procedure used.

The molecular docking results belonging to the hCA II–compound **18** interaction which has the highest binding affinity (−7.8 kcal/mol) to hCA II indicate that compound **18** interacts with ALA65, ASN67, HIS94, VAL121, VAL135, VAL143, LEU198, THR200, and PRO202 residues and the zinc ion of hCA II via hydrogen bonds, π–σ, π–π T-shaped, π–π-stacked, alkyl, π–cation, and π–alkyl interactions. The compounds investigated do not have an electron-donating amino group as in the acetazolamide molecule. Therefore, unlike the acetazolamide molecule, compound **18** does not show a metal–acceptor interaction with zinc, but a π–cation interaction is observed between the aromatic ring of compound **18** and Zn^2+^. The results obtained for the reference drug acetazolamide whose binding score is −5.9 kcal/mol show that in the hCA II–acetazolamide complex, HIS94, VAL121, LEU198, and THR199 residues interact with the ligand, and besides hydrogen bonds, π–alkyl, π–sulfur, π–π T-shaped, and metal–acceptor interactions take a role in the stabilization of the ligand–receptor complex. Although the results of the molecular docking calculations show that the most negative binding energy belongs to compound **18**, unfortunately, the experimental results show that the predicted inhibitory activity for this compound cannot be observed. In Figure 10, the binding pose of compound **23,** which is the most active compound against hCA II according to the experimental results, and the interactions between compound **23** and hCA II are given. According to the molecular docking calculations, no interactions occurred between compound **23** and the zinc cation in the hCA II—**23** complex. As a result, the binding affinity of compound **23** remains relatively low, and the highest binding affinity was not achieved for compound **23**, which showed the highest inhibitory property according to experimental results. It is essential for the compound to interact with the zinc cation to observe its inhibitory properties. However, none of the nine binding poses obtained from molecular docking for compound **23** showed any interaction with the zinc cation, unlike compound **18**.

The compounds under investigation have generally demonstrated a higher binding affinity to hCA I and hCA II compared to the reference drugs. This observation highlights the potential of these compounds as promising candidates for targeting CA enzymes in various therapeutic applications. The significance of hCA I and hCA II inhibition lies in their critical roles in physiological processes such as pH regulation, bicarbonate transport, and ion balance. Therefore, compounds showing a higher affinity for inhibiting these enzymes could hold the potential for developing therapies targeting conditions like glaucoma, epilepsy, and cancer. Understanding their inhibition profiles aids in designing more effective treatments with minimized side effects and improved patient outcomes. Thus, further investigation of their pharmacological properties and potential therapeutic benefits may be of significant benefit to drug development efforts.

On the other hand, the molecular docking results show that none of the investigated compounds have a higher binding affinity to EGFR, VEGFR2, FGFR1, and HSP90 than the corresponding reference drugs, Gefitinib, Axitinib, Ponatinib, and Geldanamycin, respectively. This observation is further supported by experimental results.

#### 3.3.3 Drug-Likeness and ADME Analyses

In this part of the study, drug-likeness and ADME analyses of the novel compounds were carried out, and TPSA^80^ (topological polar surface area), lipophilicity,^34,81−86^ water solubility,^82,86,88^ pharmacokinetics,^89^ drug-likeness^35−38,90,91^ properties related to medicinal chemistry^92−94^ of the investigated compounds were estimated. The results are given in Table S2. Generally, it was observed that the investigated compounds are drug-like compounds, have log *P*_o/w_ (consensus) in the range of 1.97–3.30 and log *K*_p_ (skin permeation) ranging from −6.62 to −5.54 cm/s.^87^

Drug-likeness and ADME analyses show that none of the investigated novel compounds violate the rules required by Lipinski, Ghose, Veber, Egan, and Muegge filters. It is also observed that all the investigated novel compounds have high gastrointestinal absorption, and all are blood–brain barrier-permeant. A substance or medication acknowledged and transported by *P*-glycoprotein is termed a *P*-glycoprotein substrate. Being identified as such signifies that *P*-glycoprotein can actively expel the substance from cells, diminishing its internal concentration. Cytochrome P450 enzymes like CYP1A2, CYP2C19, CYP2C9, CYP2D6, and CYP3A4 are involved in the metabolism of various compounds, including pharmaceuticals. Consequently, inhibiting these enzymes may elevate drug levels in the bloodstream, enhancing therapeutic outcomes while potentially escalating side effects and toxicity. One of the compounds (**22**) has been estimated to be *P*-glycoprotein substrate; two of the compounds (**11** and **24**) have been estimated to be cytochrome P450 1A2 inhibitors; all the compounds except **7** have been estimated to be cytochrome P450 2C19 inhibitor; three of the compounds (**18**, **22,** and **24**) have been estimated to be cytochrome P450 2C9 and cytochrome P450 3A4 inhibitors; and finally, three of the compounds (**7**, **13,** and **15**) have been estimated to be noncytochrome P450 2D6 inhibitors. PAINS stands for “pan-assay interference compounds,” which are chemical compounds that frequently exhibit false-positive results in a wide range of biological assays. These compounds may bind to various targets nonspecifically, leading to misleading conclusions in drug discovery and development efforts. Therefore, PAINS analyses may help filtering out such problematic compounds early in the drug discovery process to focus on more promising candidates. On the other hand, Brenk analysis evaluates various molecular properties, including size, shape, polarity, and the presence of functional groups associated with the known liabilities or undesirable properties in drugs. This could include features such as high molecular weight, excessive lipophilicity, or the presence of reactive groups associated with toxicity or metabolic instability. It is observed that all the novel compounds have no alert in PAINS; only one of them has an alert in Brenk analysis because of bearing an isolated alkene group. The molecular weights of compounds **7**, **8** and **13** over 250 g/mol. Due to the leadlikenesses of these compounds and XLOGP higher than 3.5, the leadlikenesses of 18 was predicted to be No.

## 4 Conclusions

In this study, we have synthesized alkyl (**7**–**15**) and aryl (**16**–**24**) ether derivatives containing substituted phenyl and imidazole rings as target compounds. Anticancer, antioxidant, and enzyme inhibition activity tests were applied to the synthesized compounds. As a result of activity studies, compounds **3a**, **3b**, **4a**, **4b**, **4c**, and **7** were determined to be alternative cancer drug precursor molecules. As a result of cytotoxicity studies carried out with these molecules on healthy cell lines, it was determined that they left at least 50% viability at their maximum dose (100 μM). Significant activities have been observed in studies conducted on cancer cell lines of these molecules. While the healthy cell line and glioma used in anticancer studies are mouse cells, the colon cancer and breast cancer cells used are human cell lines. In this respect, future studies and similar studies with only human cell lines or only animal cell lines will improve this study. However, using the same method, targeted transport of compounds **20** and **24**, which show effective inhibition of CA 1, and compounds **21** and **23**, which show effective inhibition of CA II, with nanoparticles may enable the development of effective treatment methods, and for future studies, this may be a topic worth exploring. Also, the antioxidant powers of the compounds synthesized using the FRAP method were tried to be determined. According to these results, the precursor molecules do not show an antioxidant effect and do not perform radical scavenging at the cellular level. Compounds may exert different modes of action on antioxidant activity. Additionally, in future studies, the “total oxidant status” values of these molecules can be investigated to examine whether they cause cells to undergo apoptosis in this way.

According to the results obtained from molecular docking studies, all compounds investigated in the current study exhibited higher binding affinities to hCA I and hCA II compared to the reference drug, acetazolamide. CA IX and XII are overexpressed in many cancers, meaning there is a much higher concentration compared to normal cells.^95^ This overexpression is linked to tumor growth, spread, and worse patient outcomes.^96^ Therefore, they are considered potential targets for cancer drugs. The role of carbonic anhydrases 1 and 2 is less clear. Although some studies suggest that these values may be elevated in cancers such as lung cancer, their functions are not directly linked to tumor survival like CA IX and XII.^97^ Inhibition studies for CA IX and XII can be carried out in future studies, and this study can be improved. The analysis of the molecular docking results shows that the inhibition of the activity of the FGFR1, VEGFR2, EGFR, and HSP90 proteins is probably not the reason for their observed selective cytotoxic activity on glioblastoma and colorectal cancer.

## Supporting Information Available

^1^H NMR, ^13^C NMR, FTIR, and mass spectra of all compounds, geometry-optimized structures of the compounds, molecular electrostatic potential maps of the compounds, binding poses and ligand–receptor interactions, binding scores obtained from molecular docking calculations, drug-likeness and ADME analyses, effect of synthesized compounds on hCA I and II isoenzyme activity, and % activity–inhibitor concentration graphs of all the compounds (PDF)
