# Supplementary material for: Imidazole-Derived Alkyl and Aryl Ethers: Synthesis, Characterization, In Vitro Anticancer and Antioxidant Activities, Carbonic Anhydrase I–II Inhibition Properties, and In Silico Studies
Source: ACS Omega. 2024 May 3;9(19):20937–56. doi: 10.1021/acsomega.4c00028 (PMC11097166; doi:10.1021/acsomega.4c00028)
Supplement: Supplementary file 1 — ao4c00028_si_001.pdf [file ao4c00028_si_001.pdf]

Supporting Information

Imidazole-derived alkyl and aryl ethers: Synthesis, characterization, *in vitro* anticancer and antioxidant activities, carbonic anhydrase I-II inhibition properties and *in silico* studies

Mays Faris[a], Hayrani Eren Bostanci[b]*, İbrahim Ozcan[a], Mustafa Öztürk[c], Umıt M. Kocyigit[b], Taner Erdogan[d], Hakan Tahtaci[a]*

[a]Karabuk University, Department of Chemistry, Faculty of Science, 78050, Karabuk, Türkiye.

[b]Sivas Cumhuriyet University, Faculty of Pharmacy, Department of Biochemistry Sivas,Türkiye

[c]Sivas Cumhuriyet University, Sivas Vocational School of Technical Sciences, 58010, Sivas, Türkiye.

[d]Kocaeli University, Kocaeli Vocational School, Department of Chemistry and Chemical Processing Technologies, 41140, Kocaeli, Türkiye.

Table of Content:

1. ^{1}H NMR, ^{13}C NMR, FTIR and Mass Spectra of all Compounds

2. Geometry-Optimized Structures of the Novel and Known Compounds

3. Molecular Electrostatic Potential Maps of the Novel and Known Compounds

4. Binding Poses and Ligand-Receptor Interactions

5. Binding Scores

6. Drug-likeness and ADME Analyses

7. Effect of Synthesized Compounds on hCA I and II Isoenzymes Activity

8. % Activity - inhibitor concentration graphs of all the compounds

1. ^{1}H NMR, ^{13}C NMR, FTIR and Mass Spectra of all Compounds

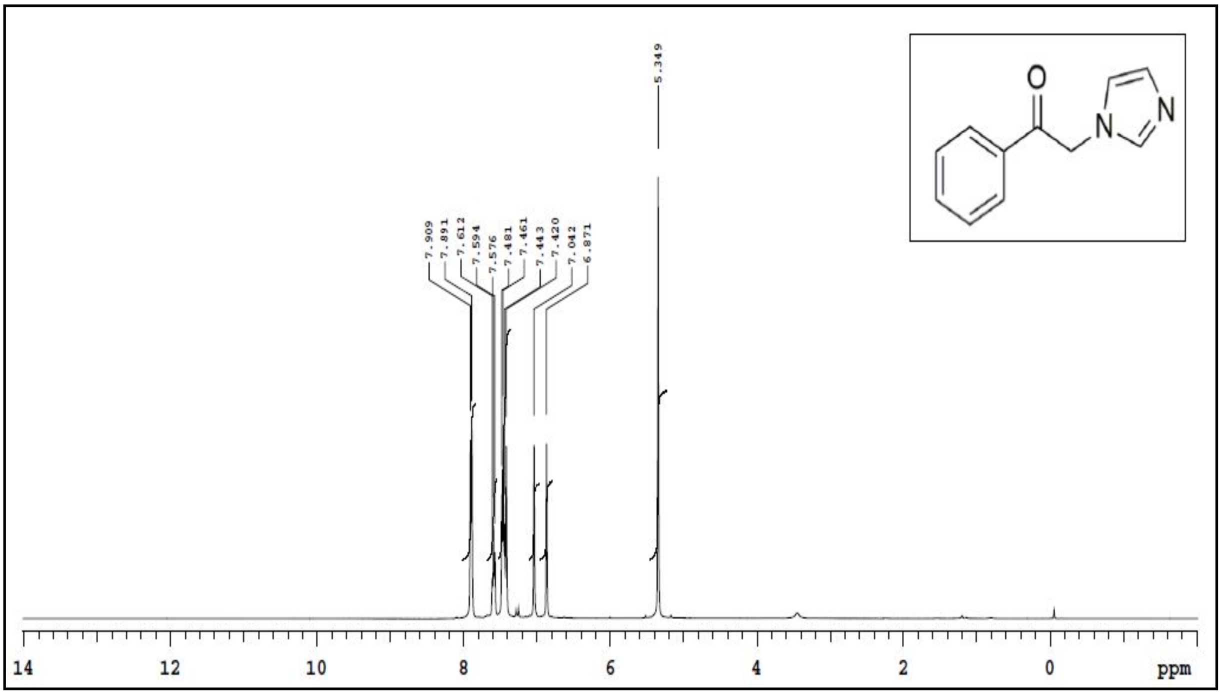

Figure S1. ^{1}H NMR Spectrum ($CDCl_3$) **(3a).**

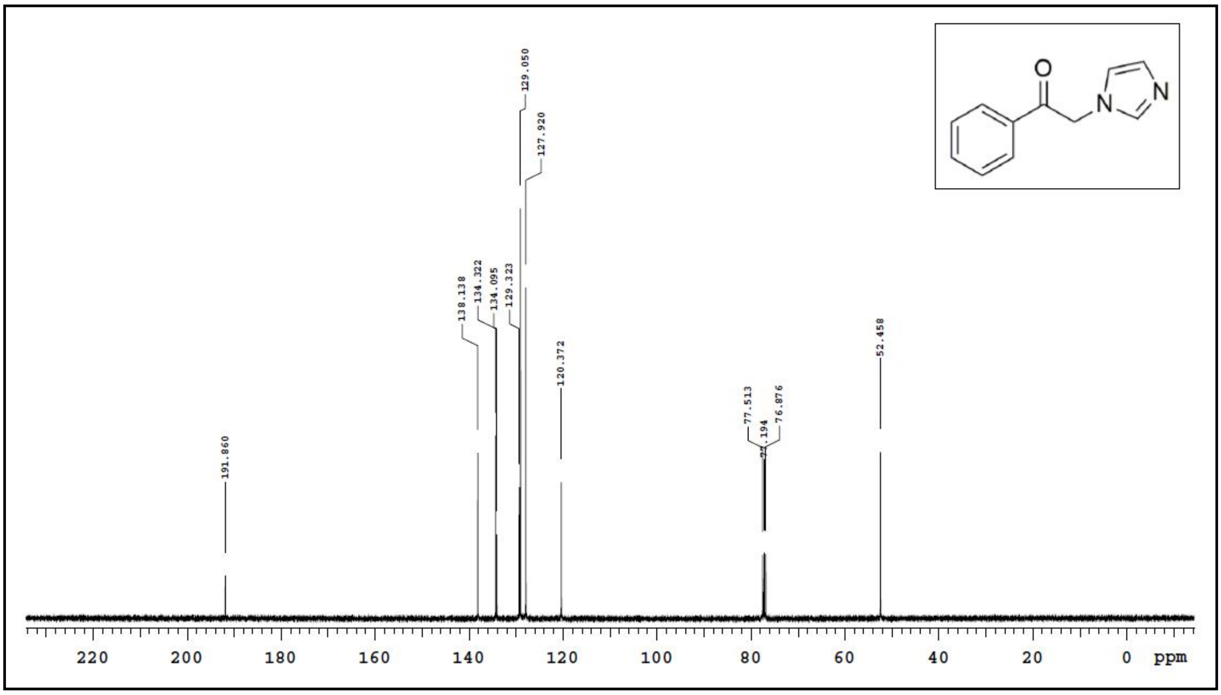

Figure S2. ^{13}C NMR Spectrum ($CDCl_3$) **(3a).**

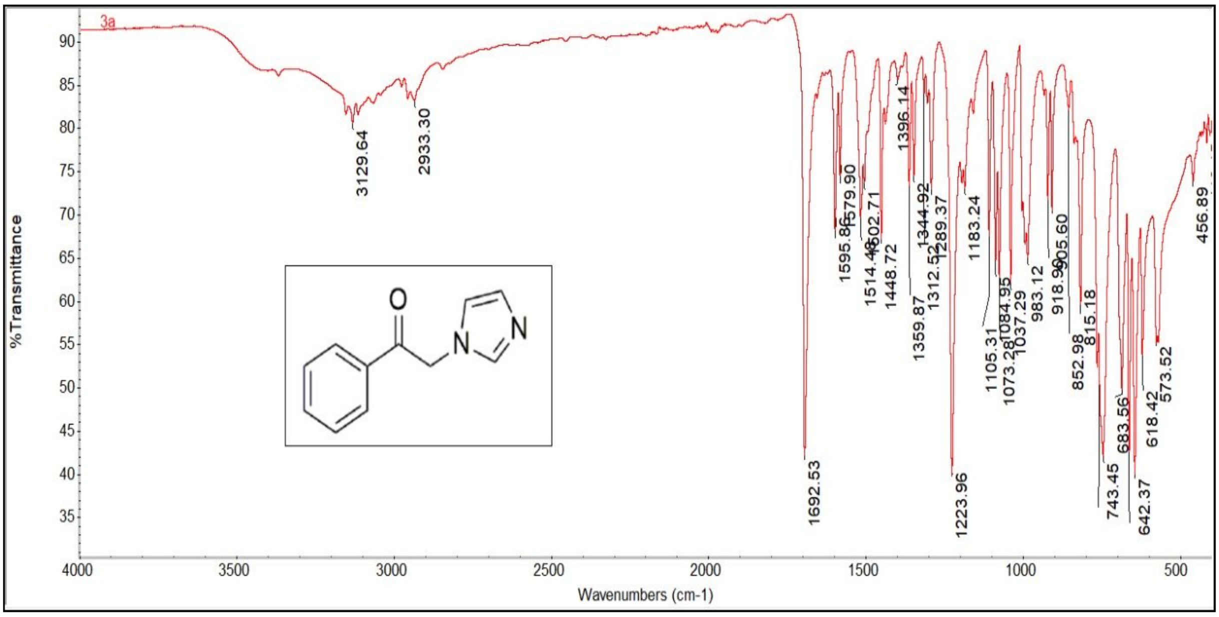

Figure S3. FT-IR Spectrum **(3a).**

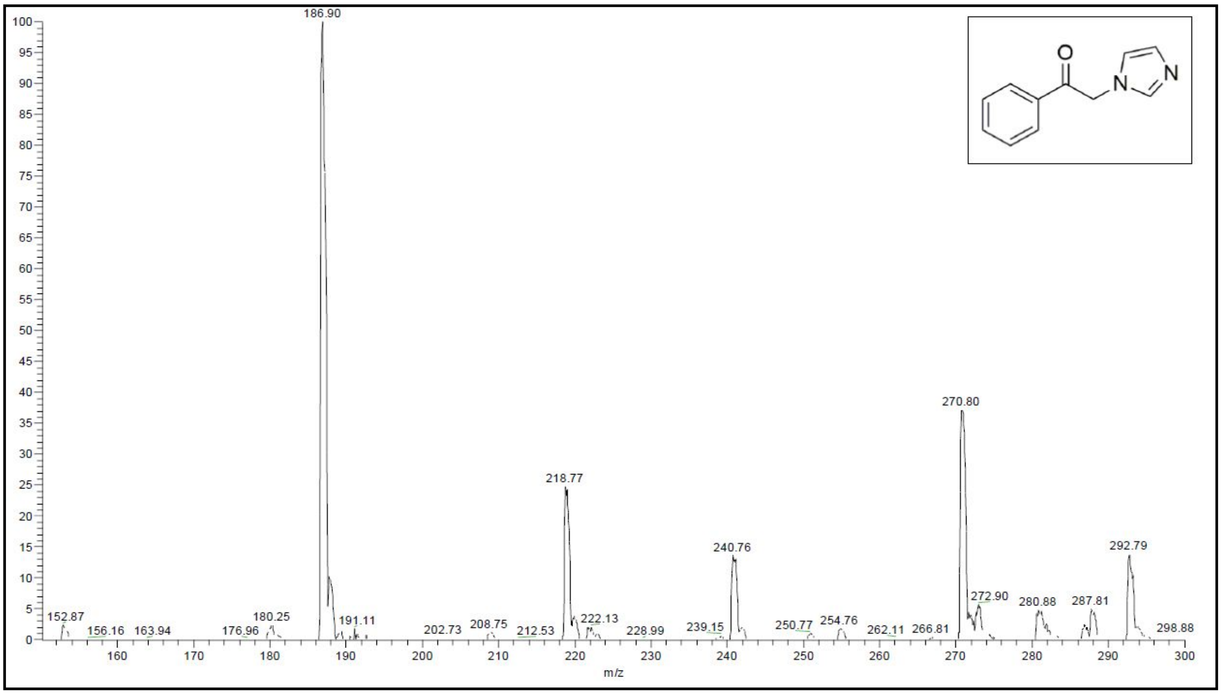

Figure S4. Mass Spectrum **(3a).**

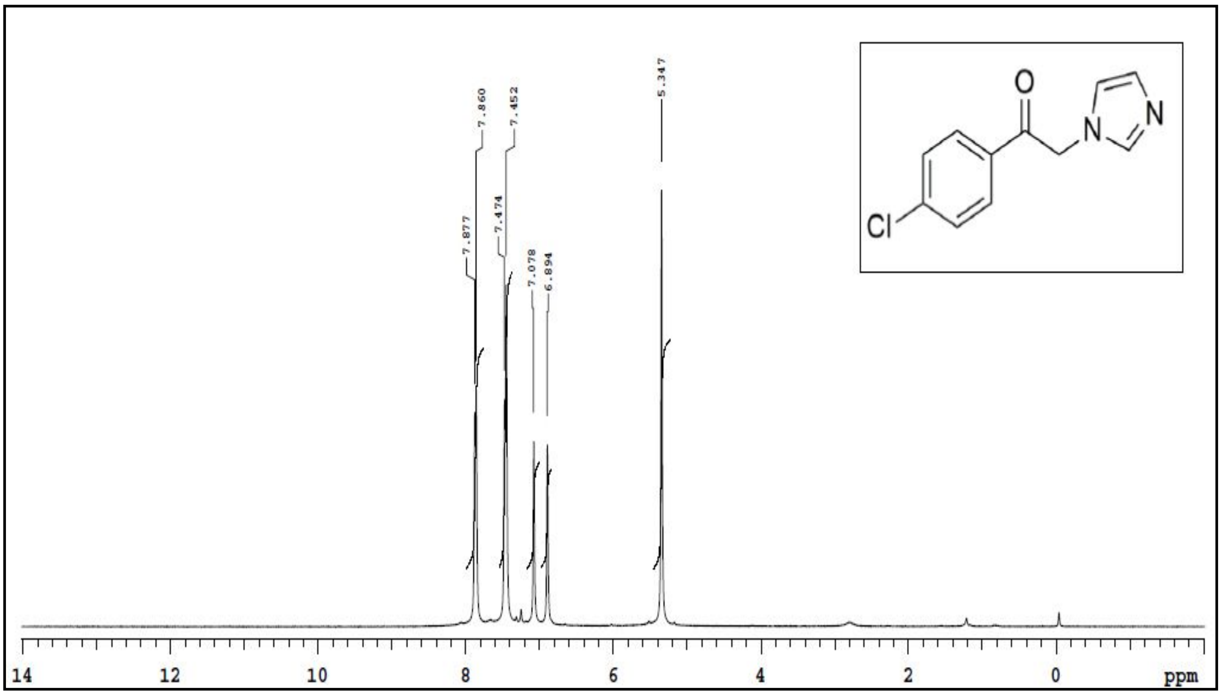

Figure S5. ^{1}H NMR Spectrum ($CDCl_3$) **(3b).**

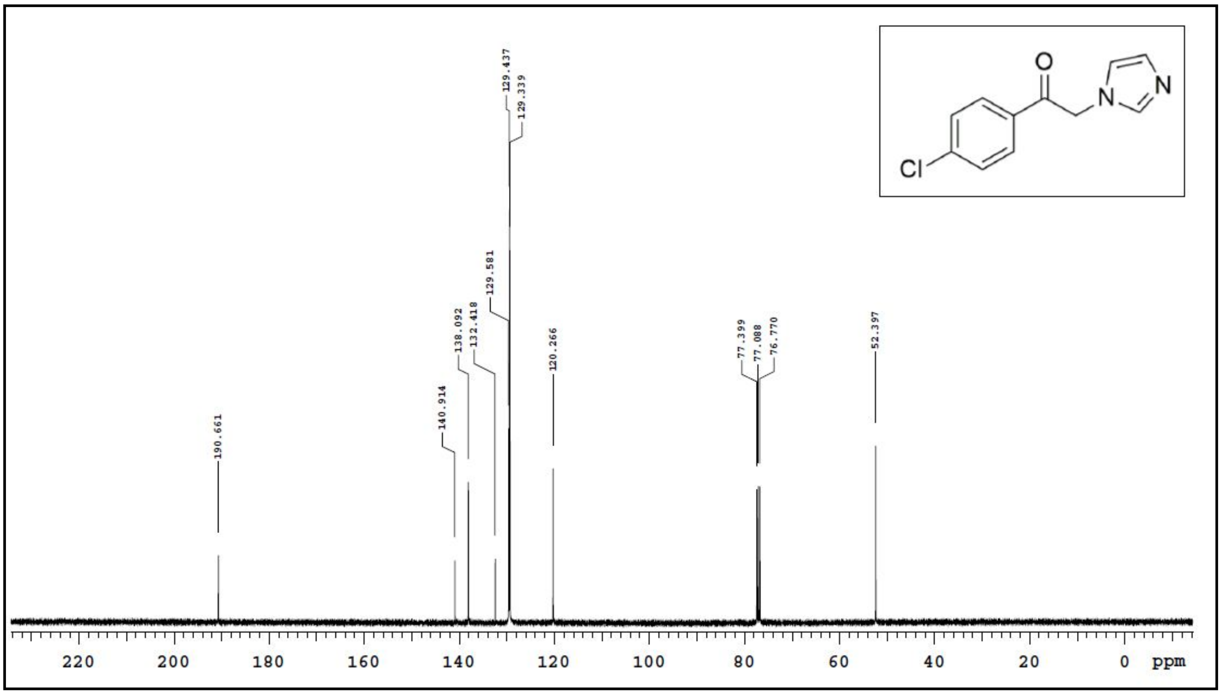

Figure S6. ^{13}C NMR Spectrum ($CDCl_3$) **(3b).**

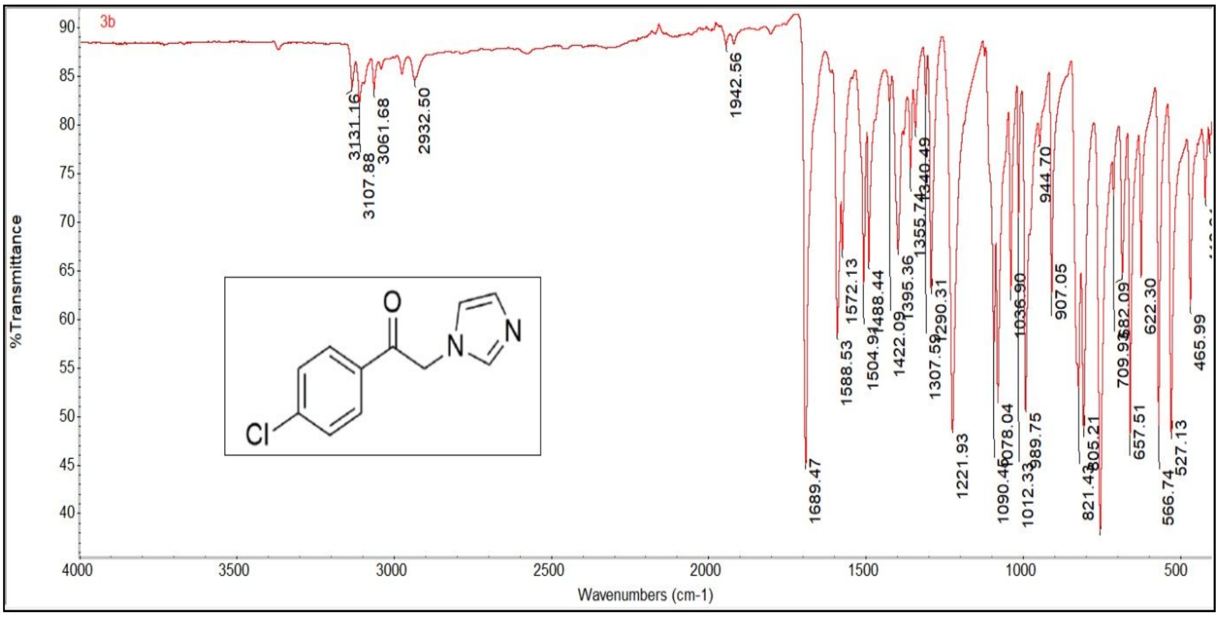

Figure S7. FT-IR Spectrum **(3b).**

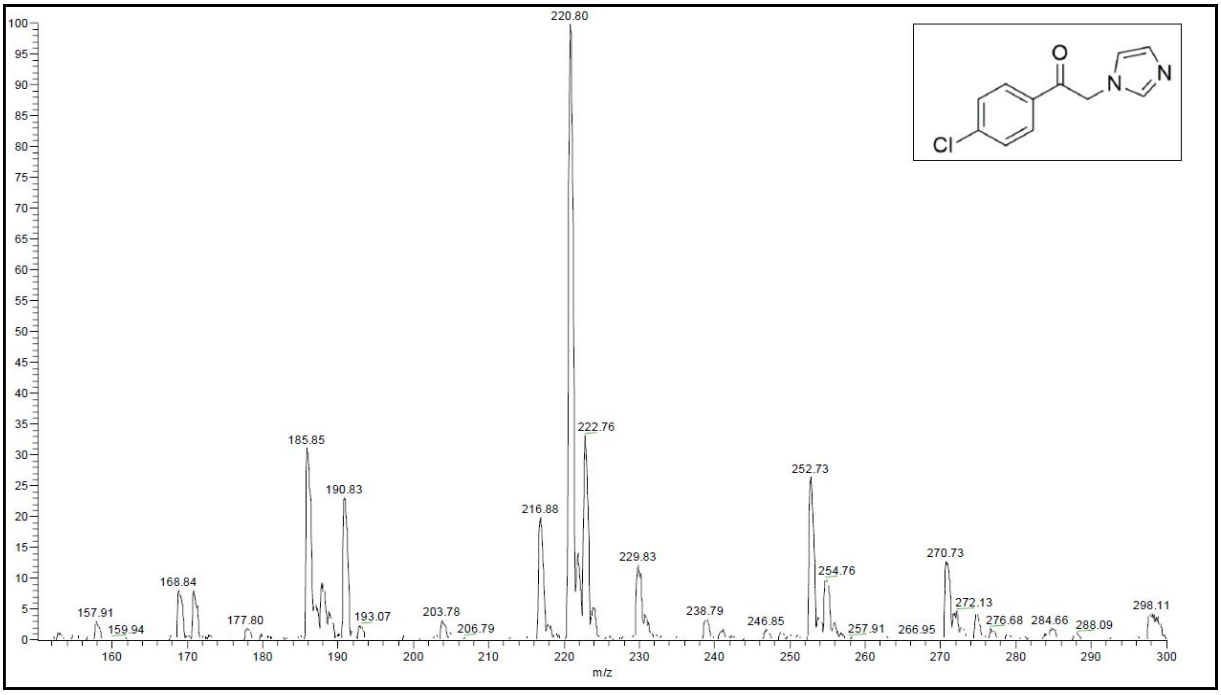

Figure S8. Mass Spectrum **(3b).**

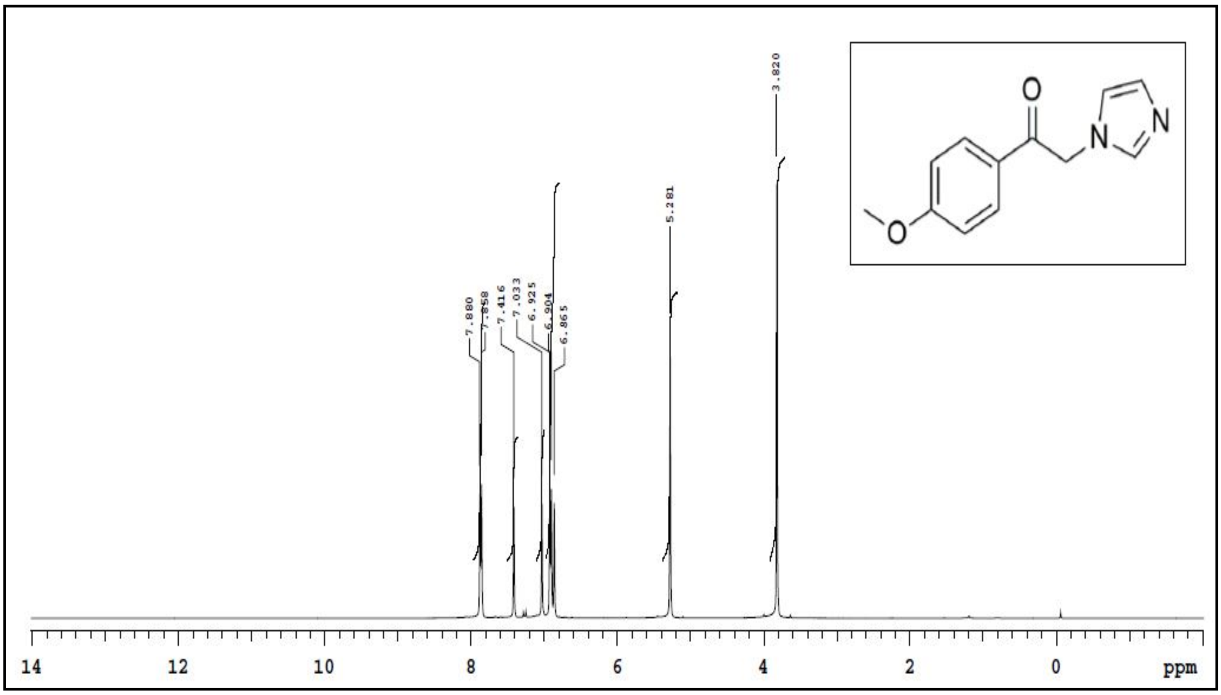

Figure S9. ^{1}H NMR Spectrum ($CDCl_3$) **(3c).**

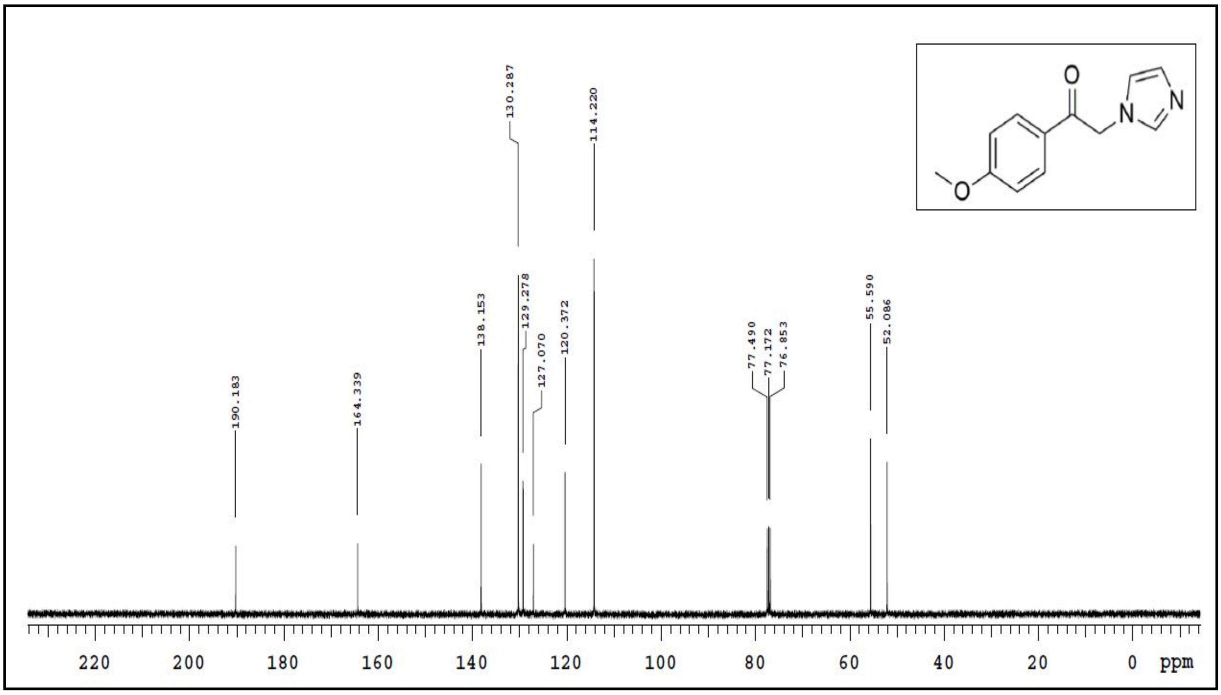

Figure S10. ^{13}C NMR Spectrum ($CDCl_3$) **(3c).**

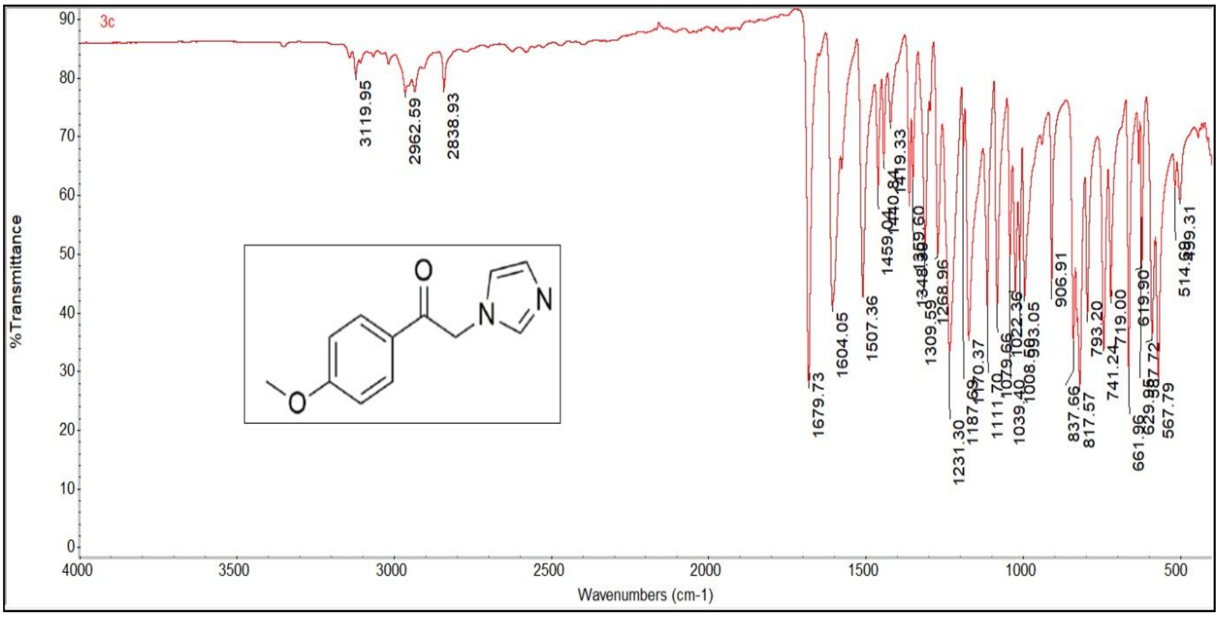

Figure S11. FT-IR Spectrum **(3c).**

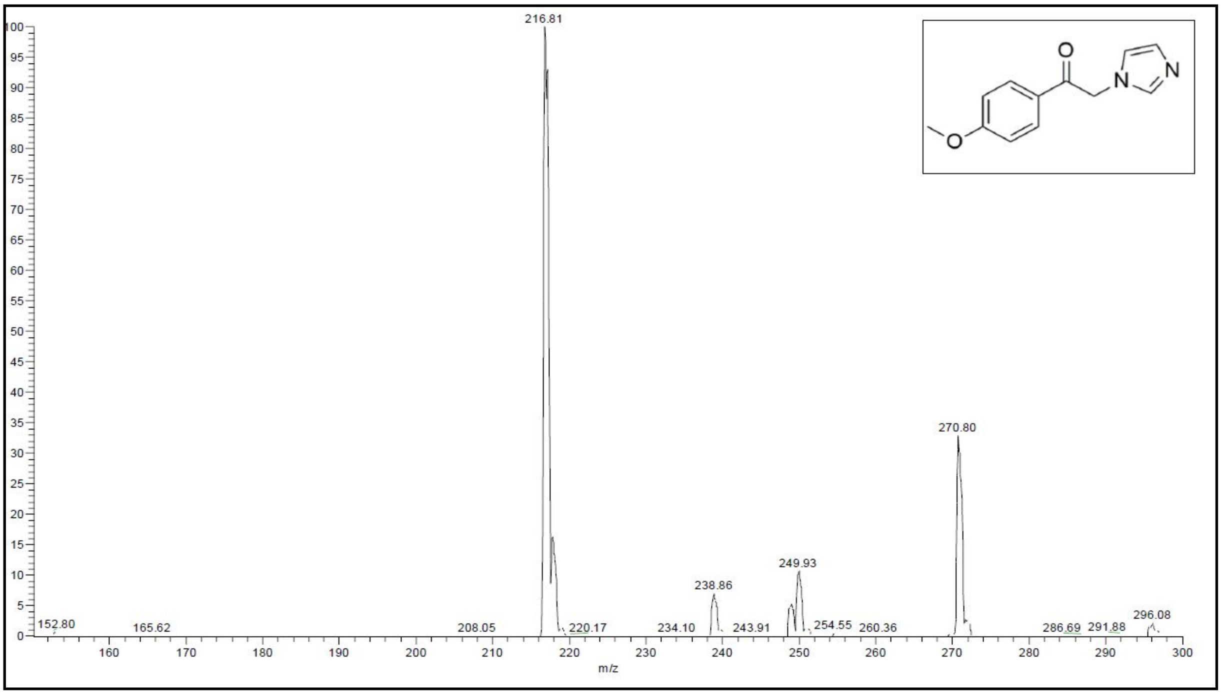

Figure S12. Mass Spectrum **(3c).**

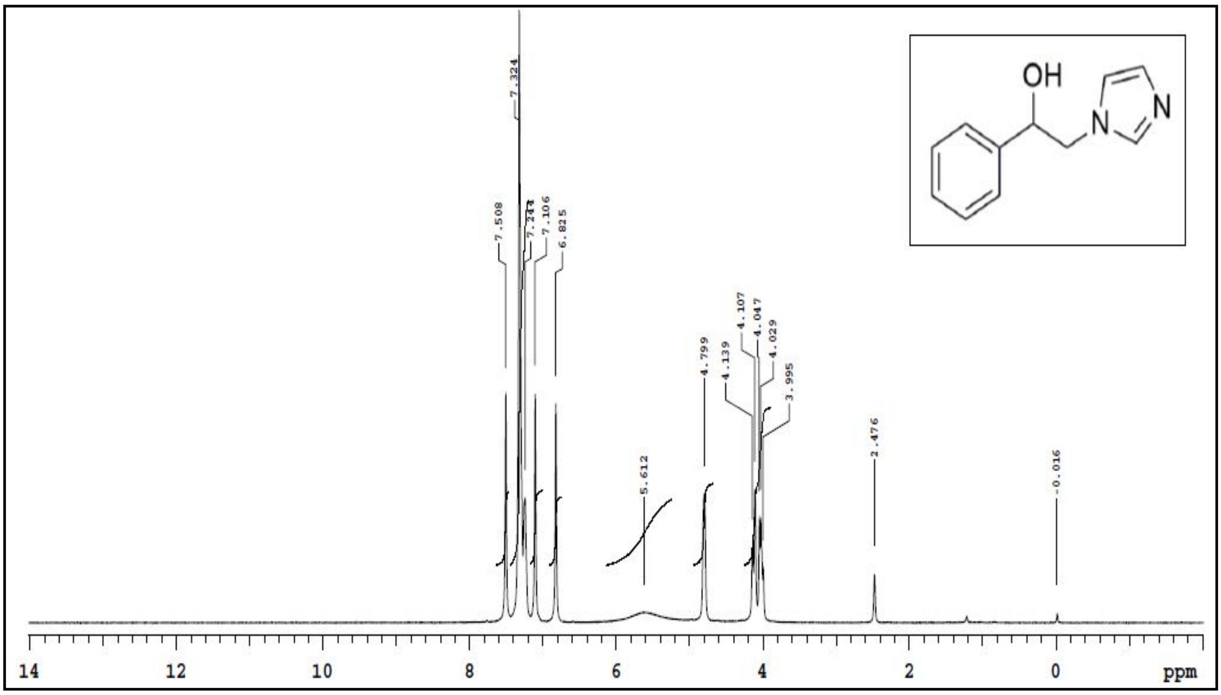

Figure S13. ^{1}H NMR Spectrum (DMSO-d_6) **(4a).**

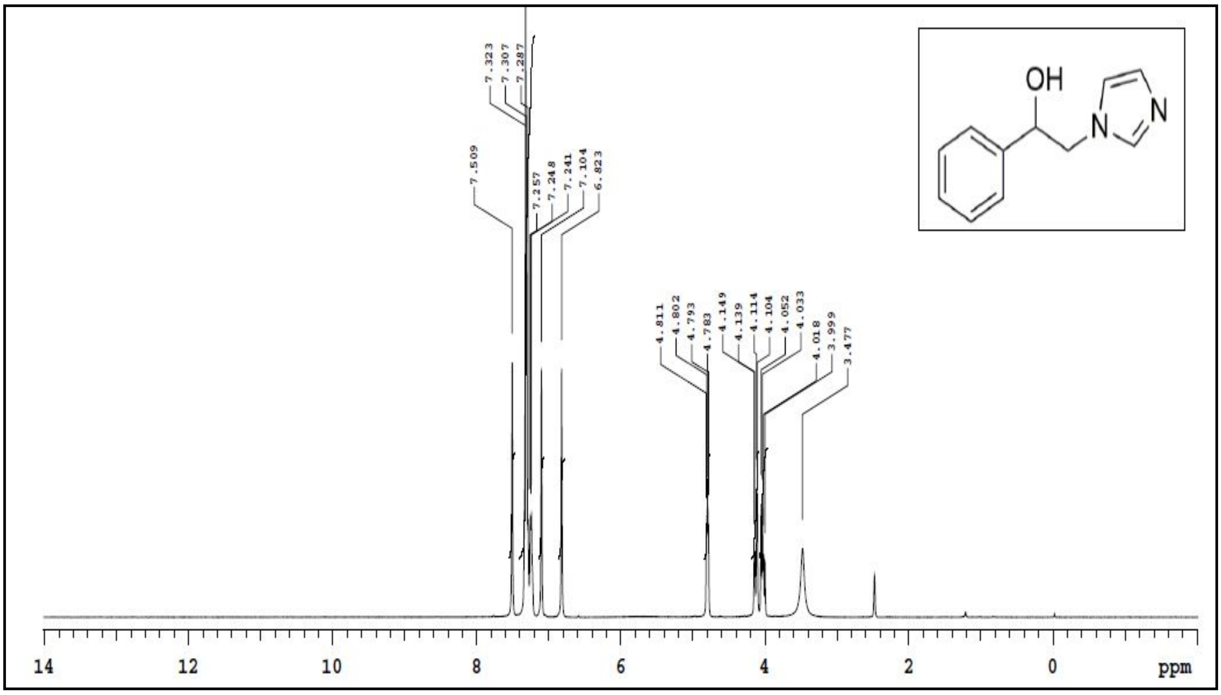

Figure S14. ^{1}H NMR Exchange Spectrum (DMSO-d_6) **(4a).**

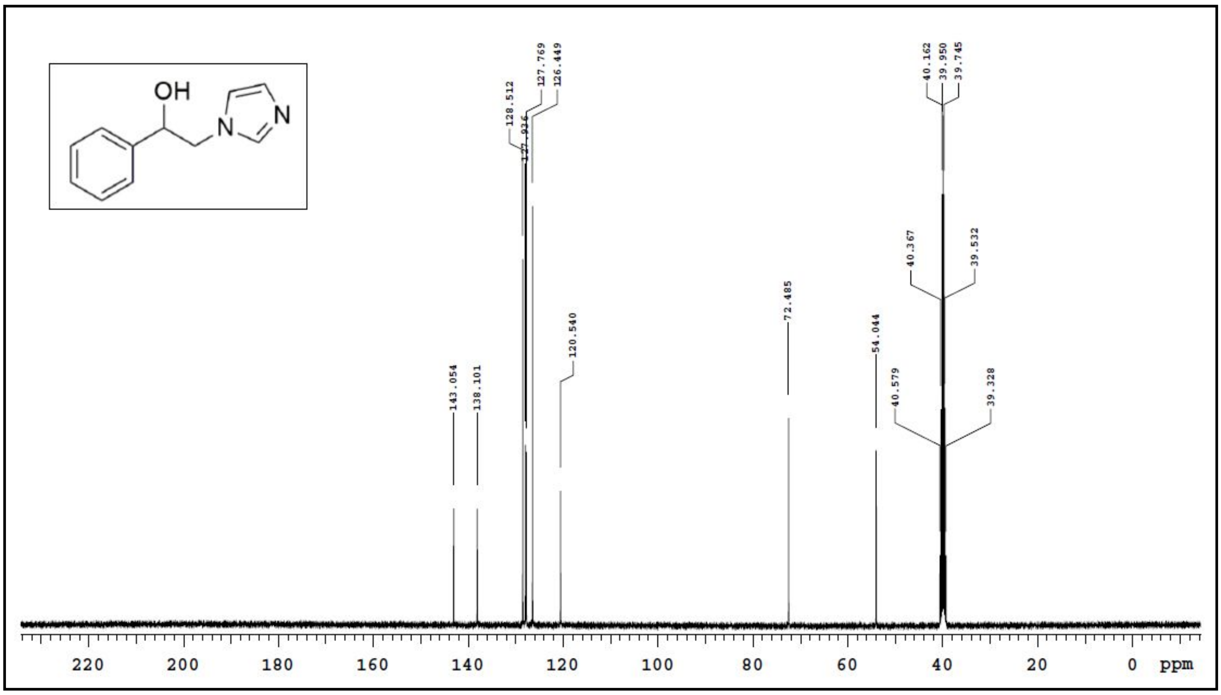

Figure S15. ^{13}C NMR Spectrum (DMSO-d_6) **(4a).**

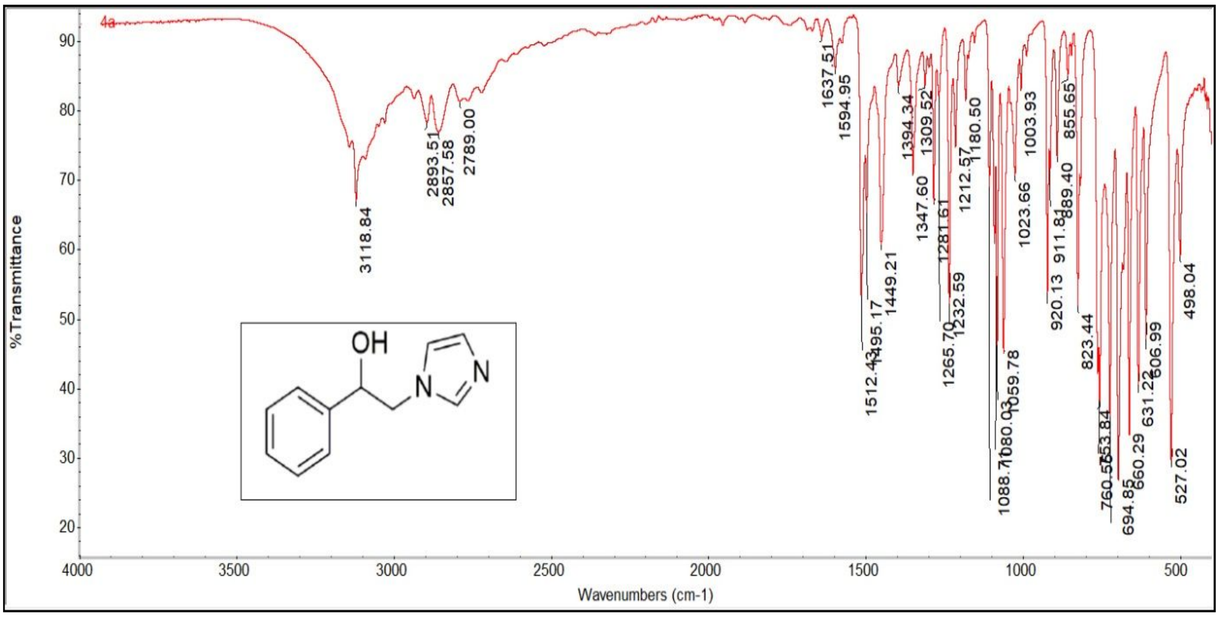

Figure S16. FT-IR Spectrum **(4a).**

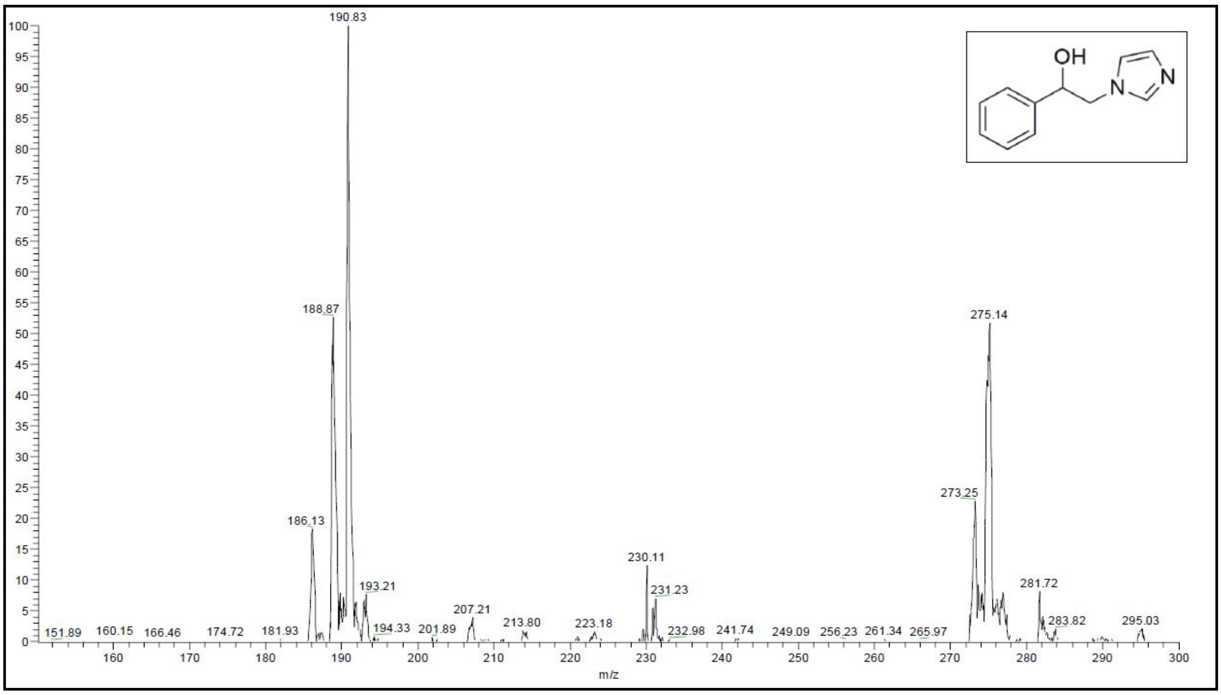

Figure S17. Mass Spectrum **(4a).**

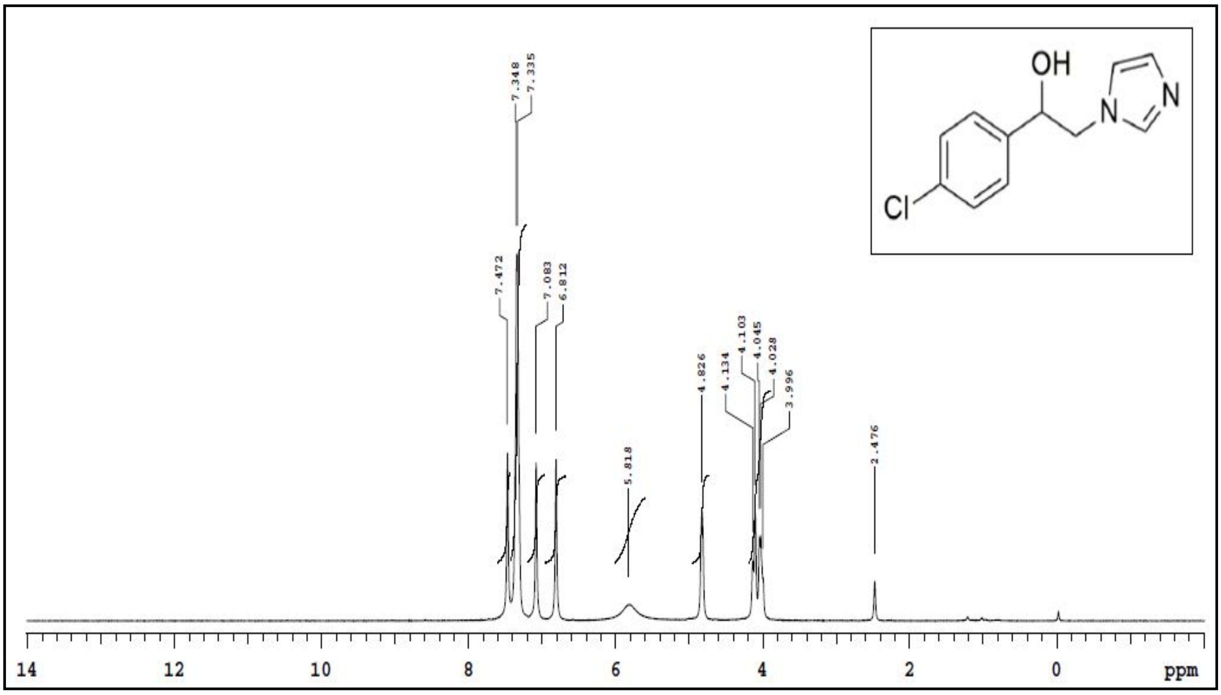

Figure S18. ^{1}H NMR Spectrum (DMSO-d$_6$) **(4b).**

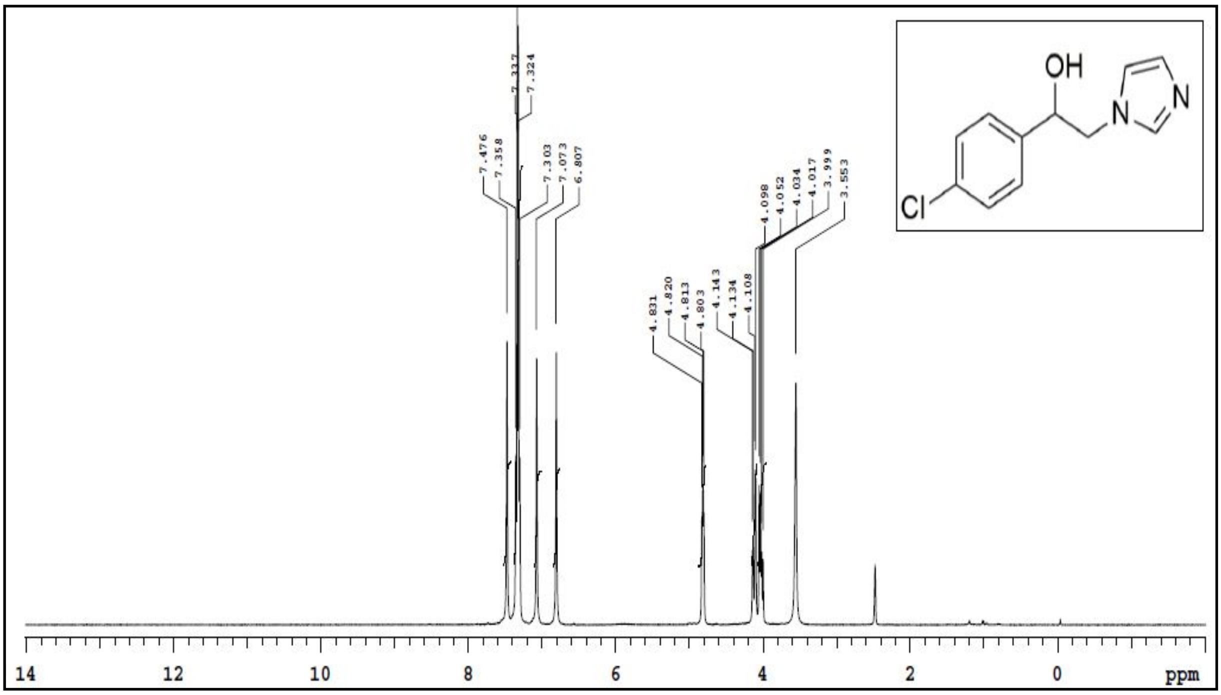

Figure S19. ^{1}H NMR Exchange Spectrum (DMSO-d_6) **(4b).**

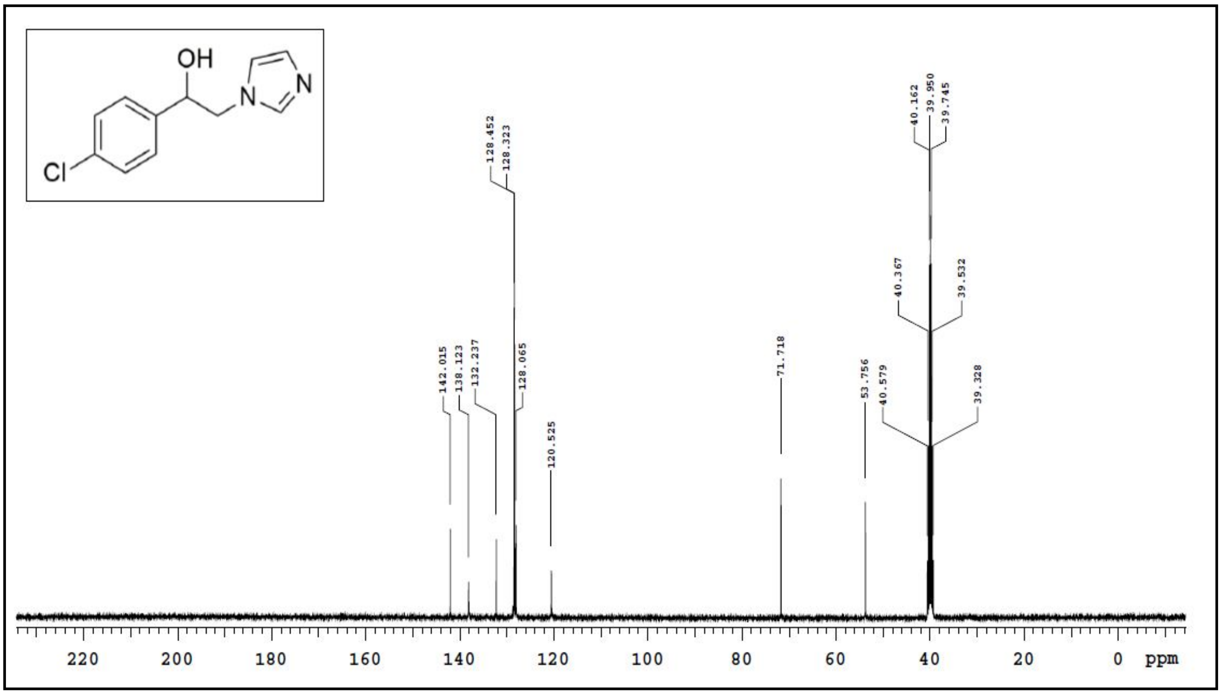

Figure S20. ^{13}C NMR Spectrum (DMSO-d_6) **(4b).**

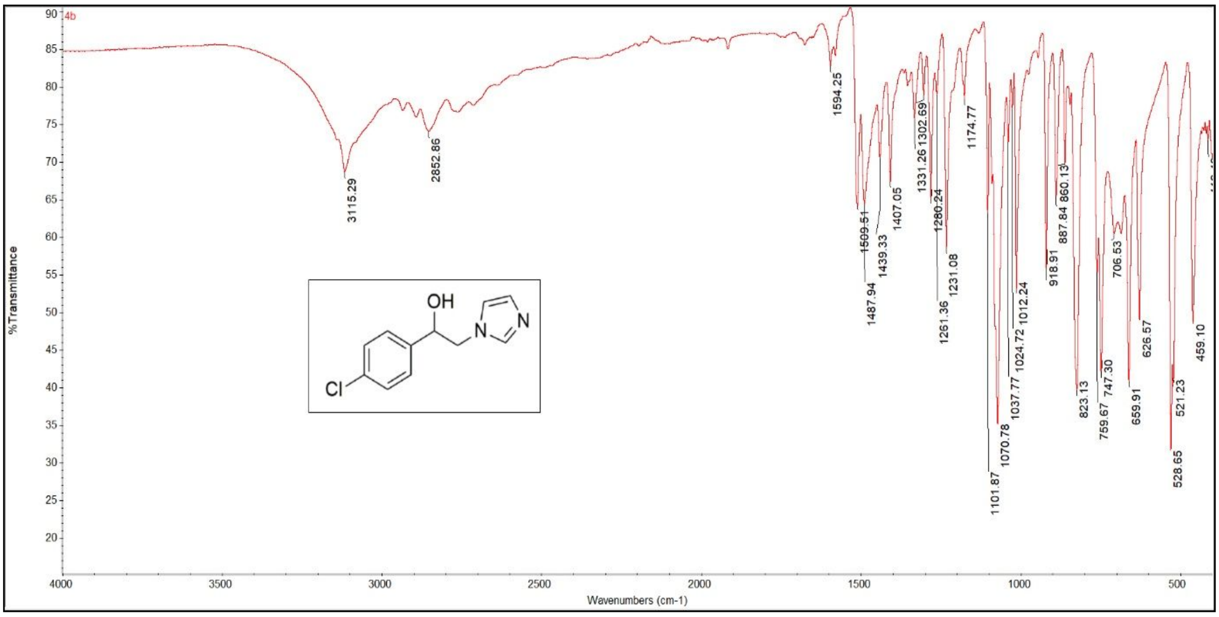

Figure S21. FT-IR Spectrum **(4b).**

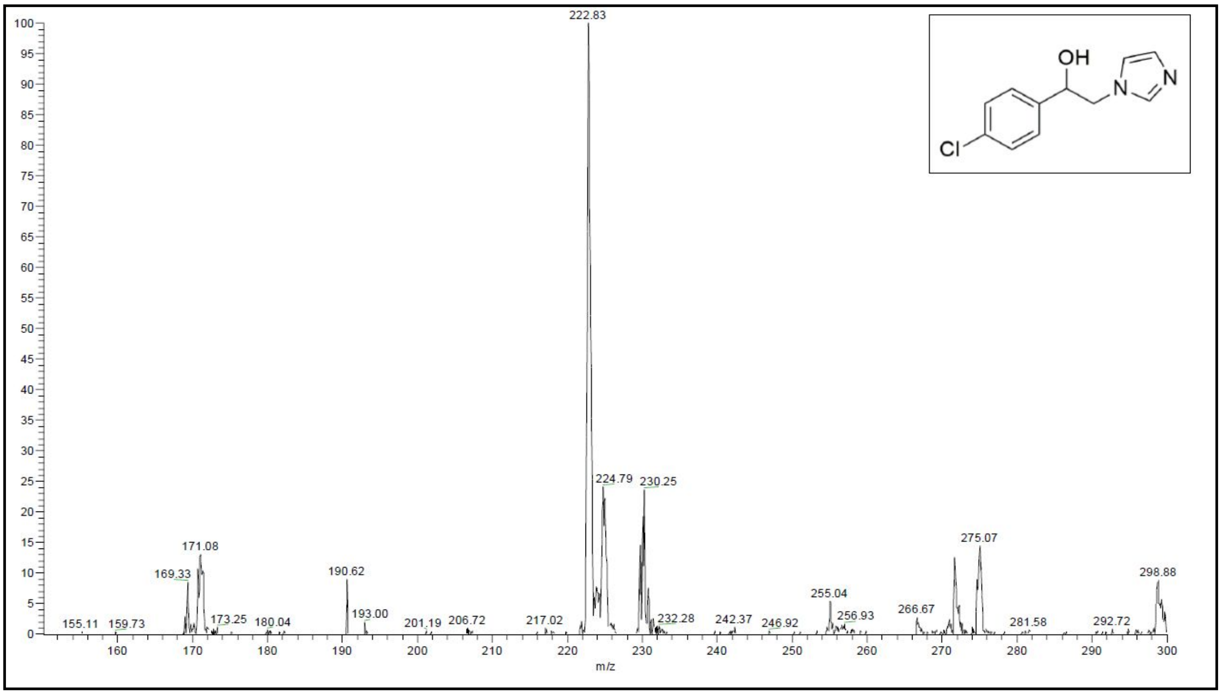

Figure S22. Mass Spectrum **(4b).**

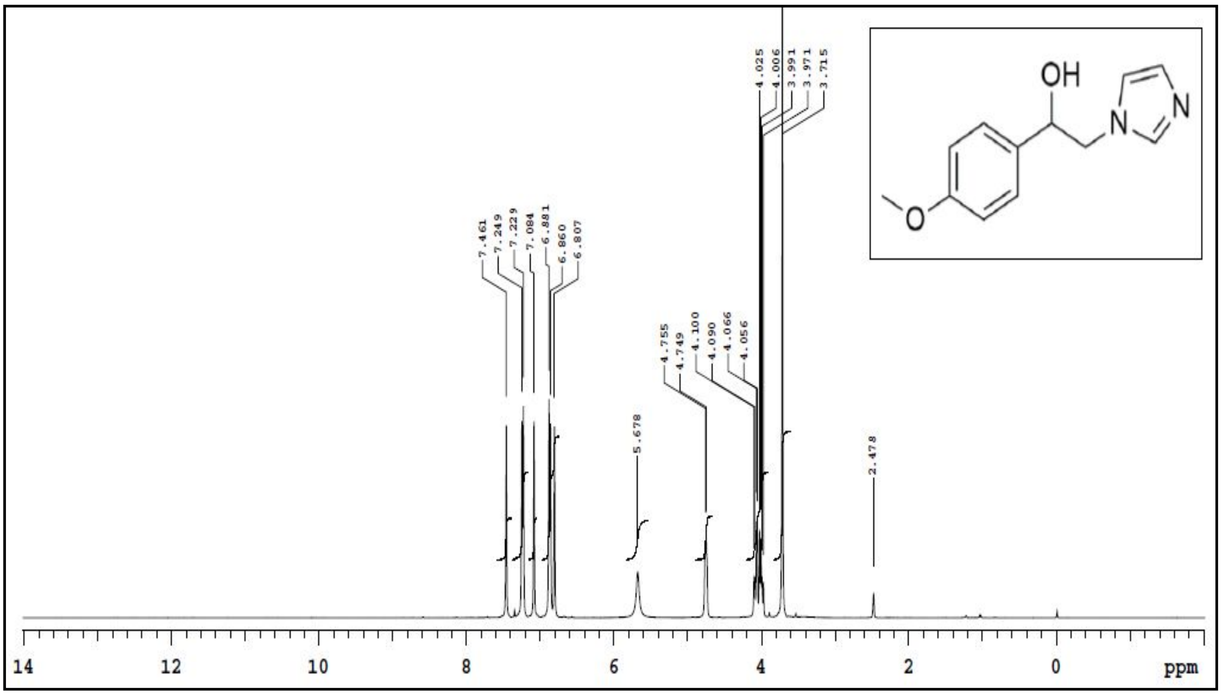

Figure S23. ^{1}H NMR Spectrum (DMSO-d$_6$) **(4c).**

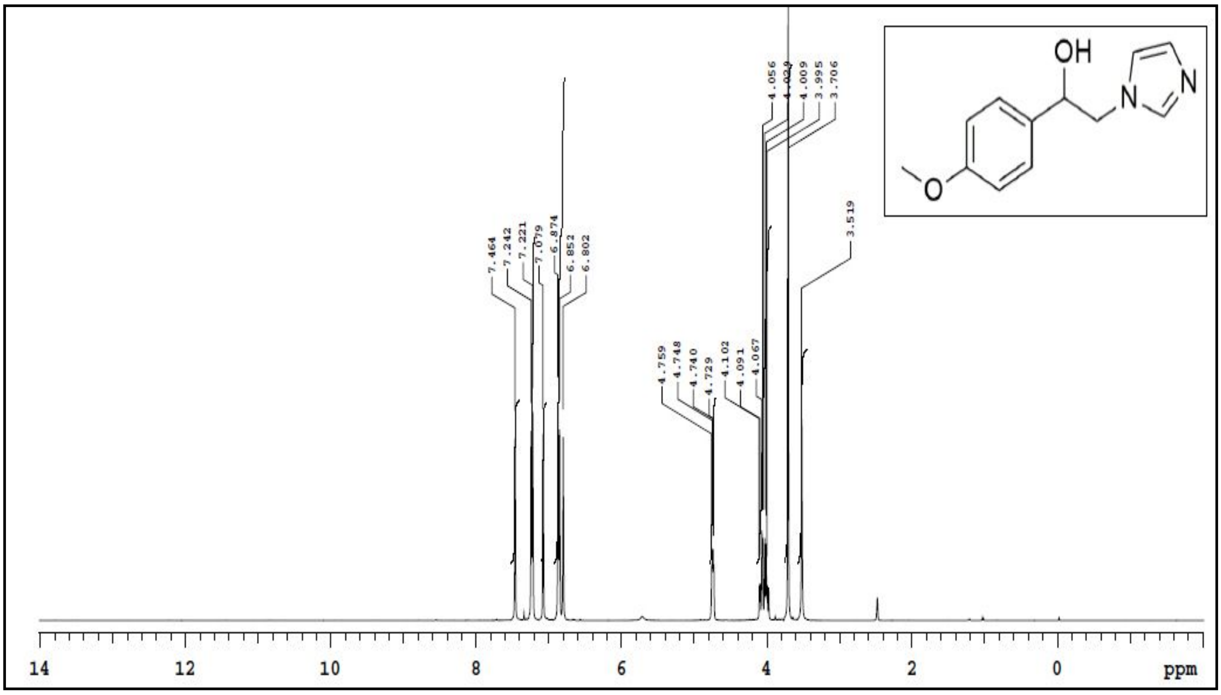

Figure S24. ^{1}H NMR Exchange Spectrum (DMSO-d$_6$) **(4c).**

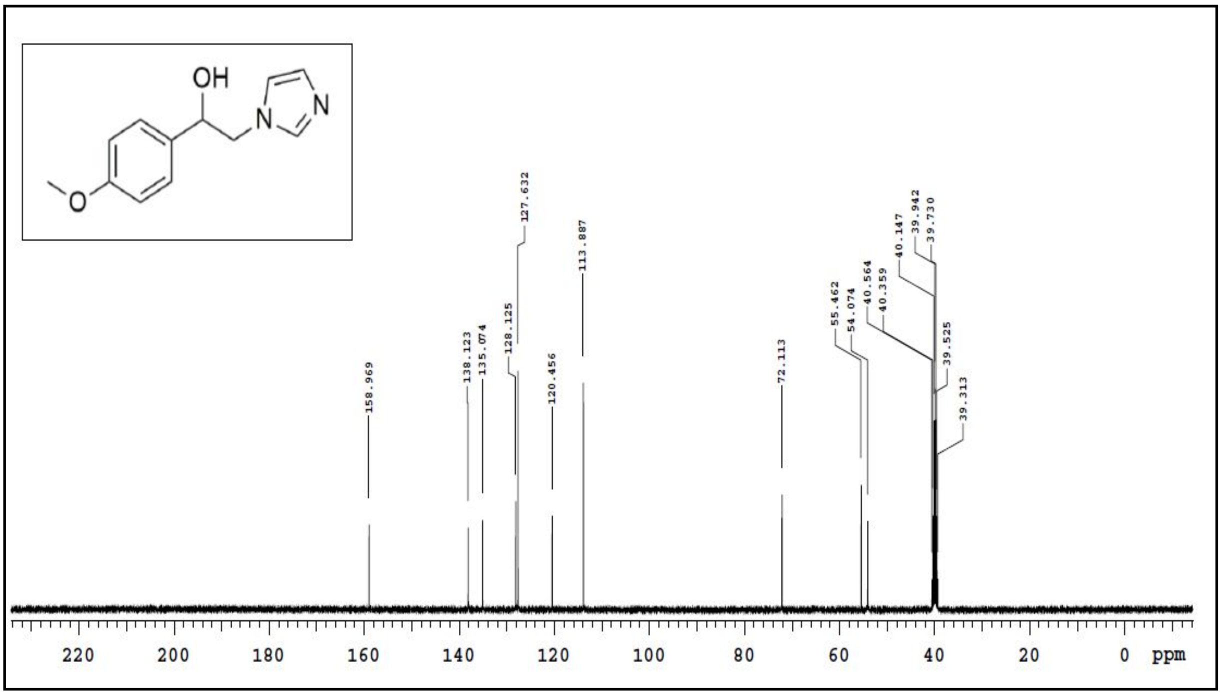

Figure S25. ^{13}C NMR Spectrum (DMSO-d_6) **(4c).**

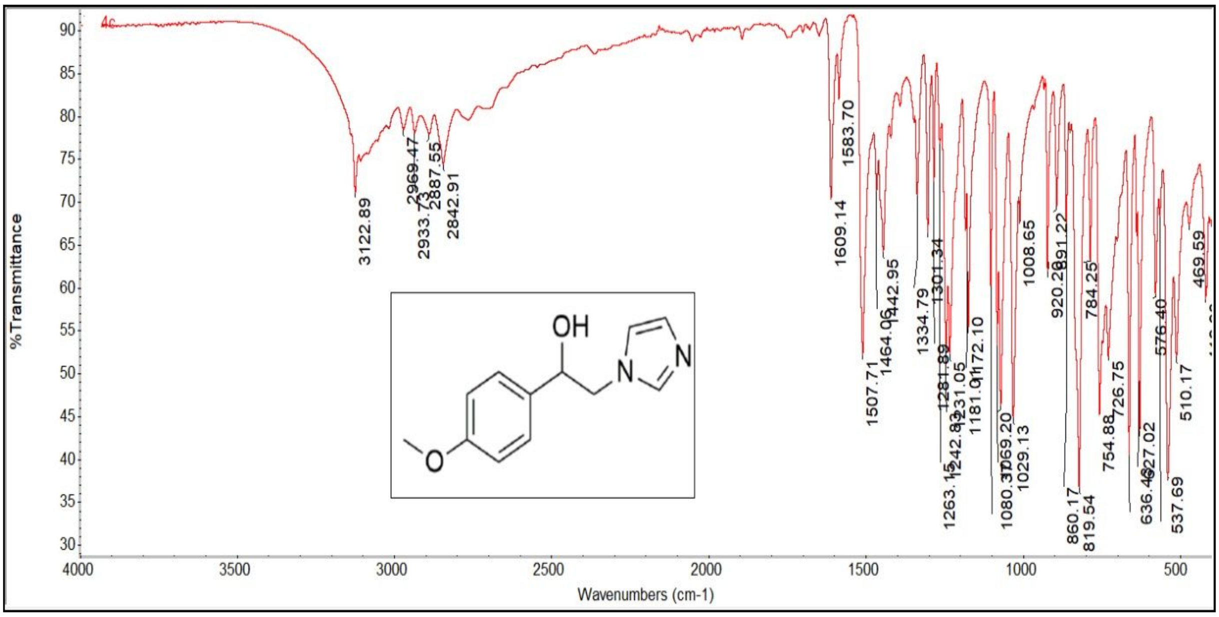

Figure S26. FT-IR Spectrum **(4c).**

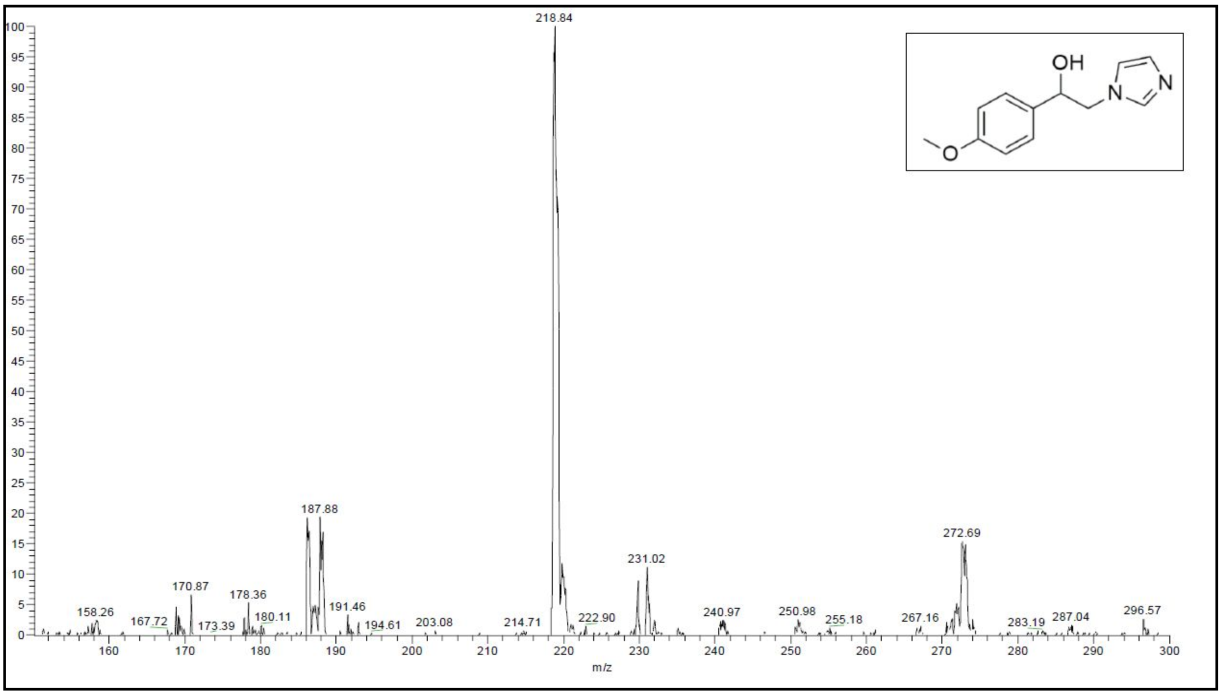

Figure S27. Mass Spectrum **(4c).**

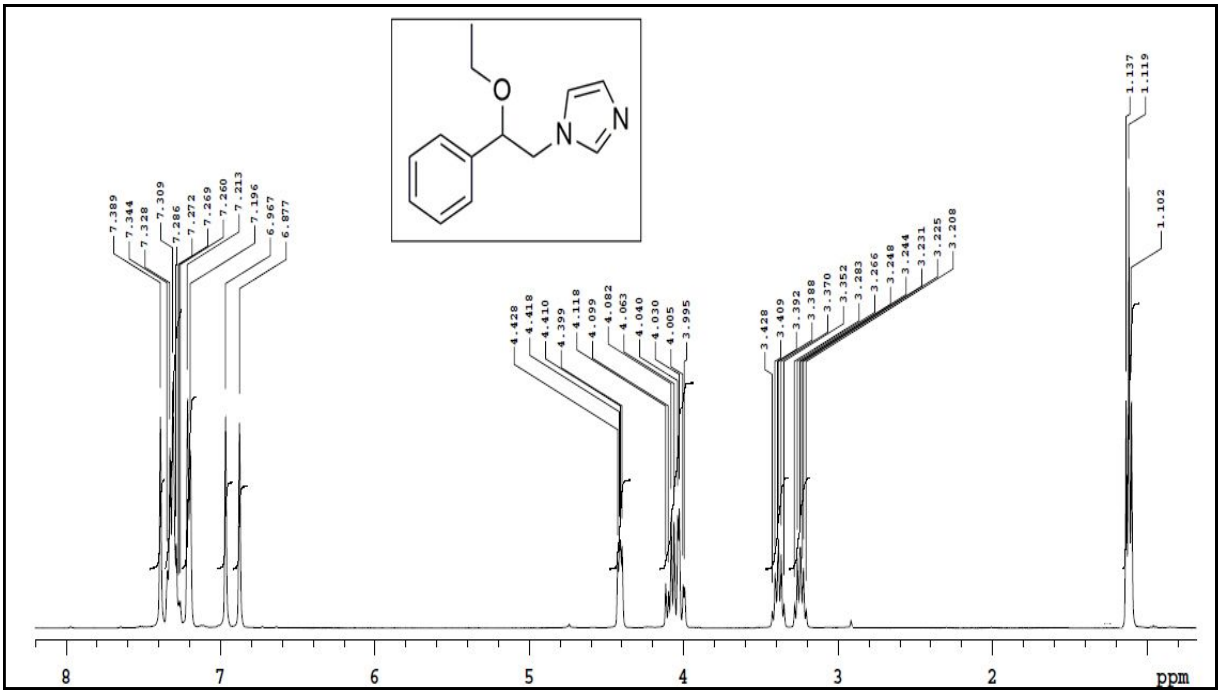

Figure S28. ^{1}H NMR Spectrum ($CDCl_3$) **(7).**

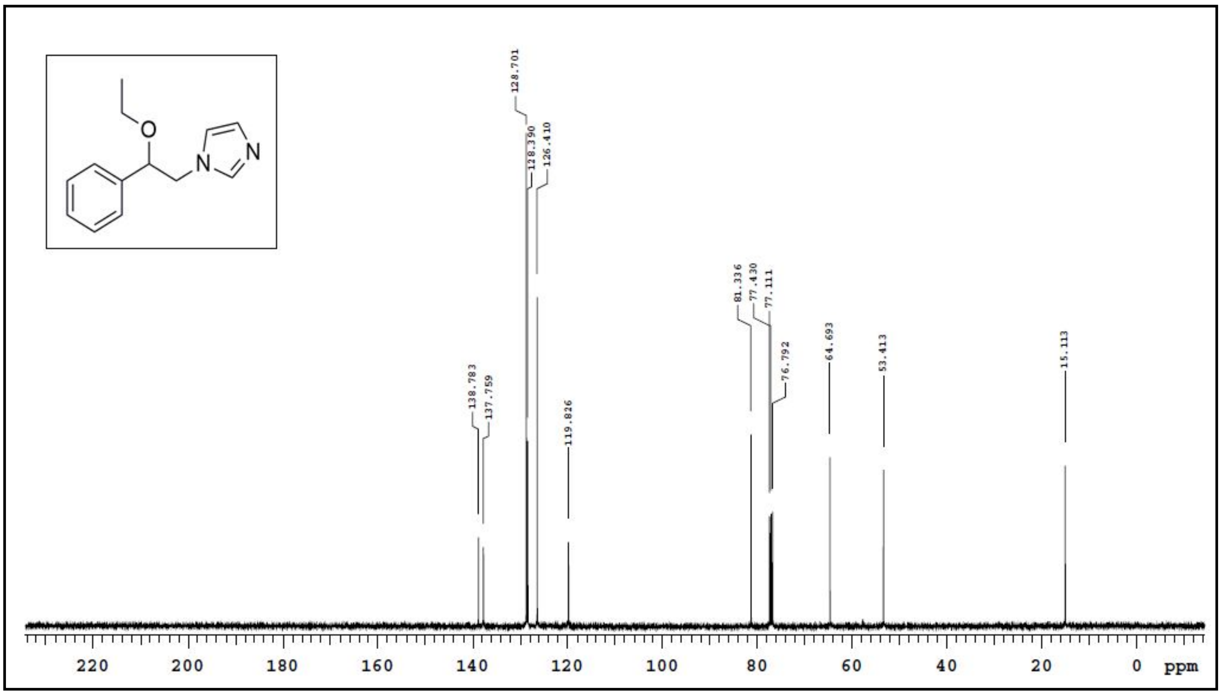

Figure S29. ^{13}C NMR Spectrum ($CDCl_3$) **(7).**

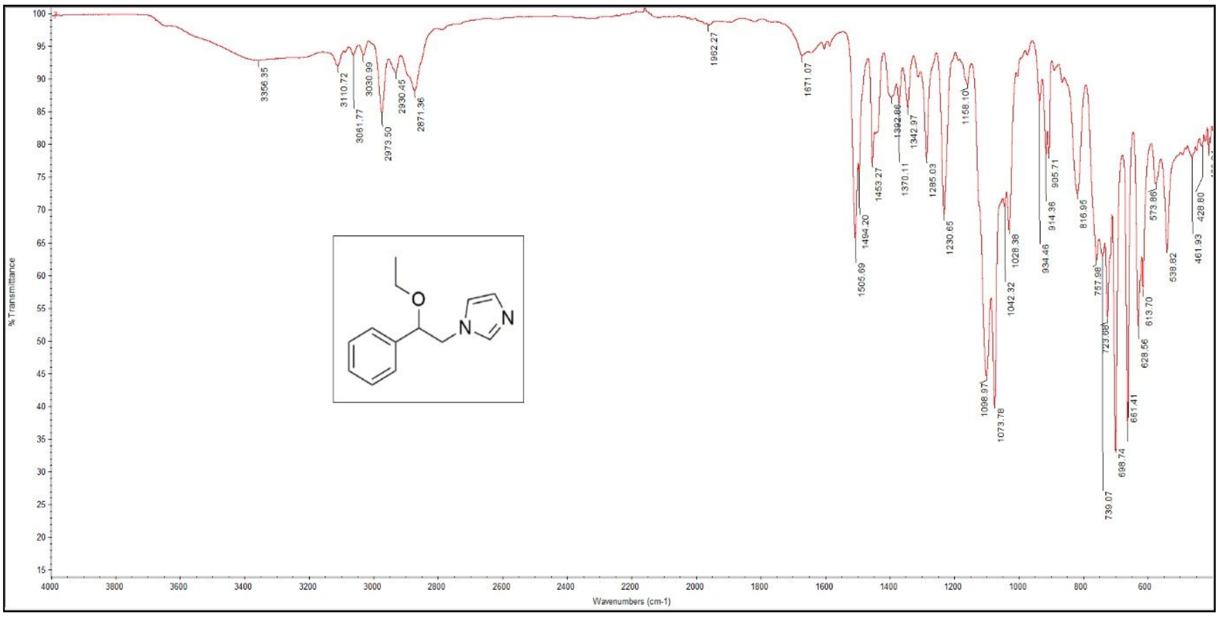

Figure S30. FT-IR Spectrum **(7).**

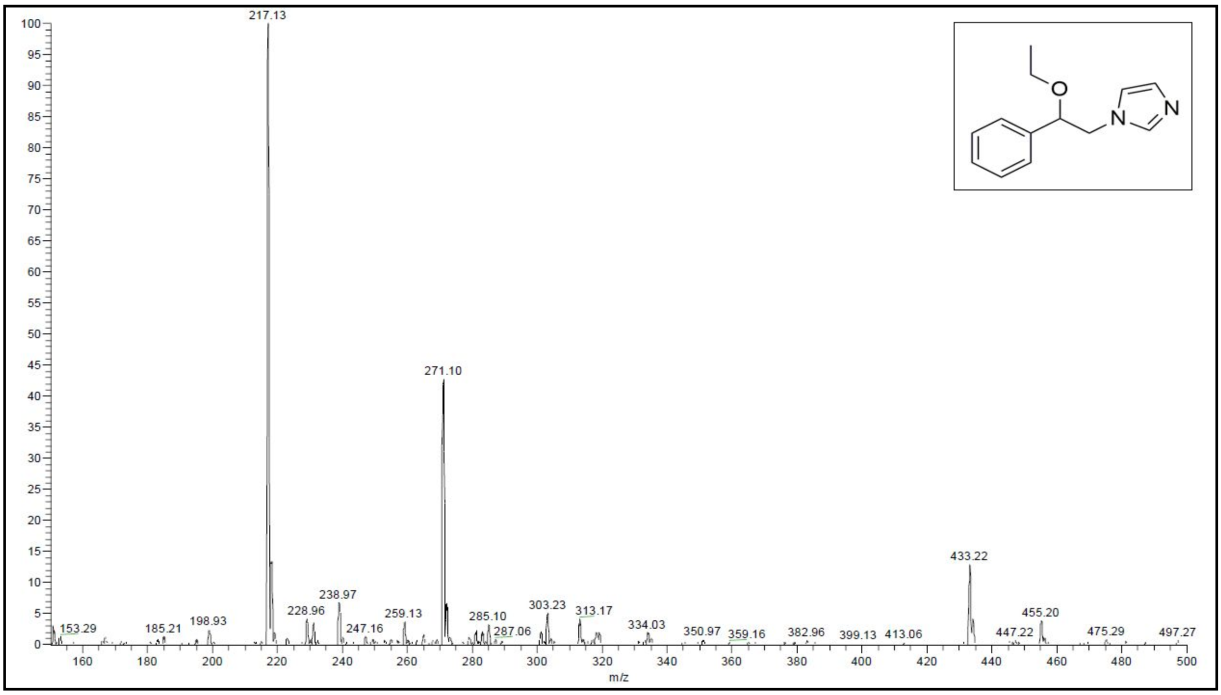

Figure S31. Mass Spectrum **(7).**

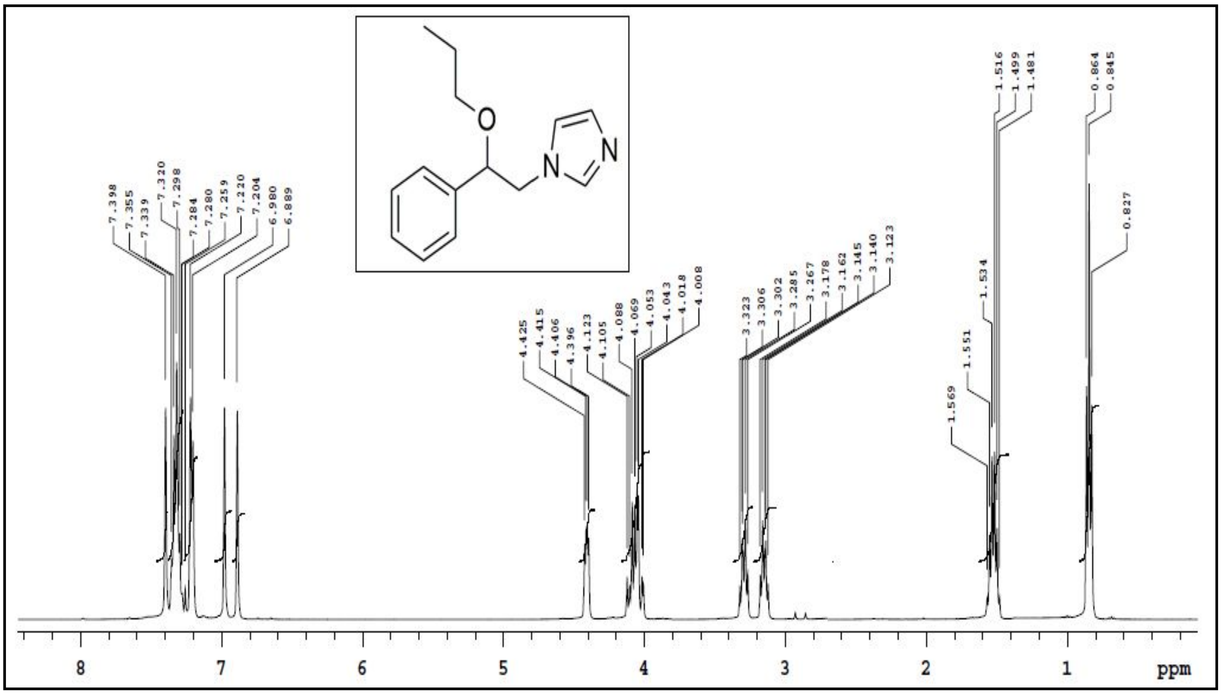

Figure S32. ^{1}H NMR Spectrum ($CDCl_3$) **(8).**

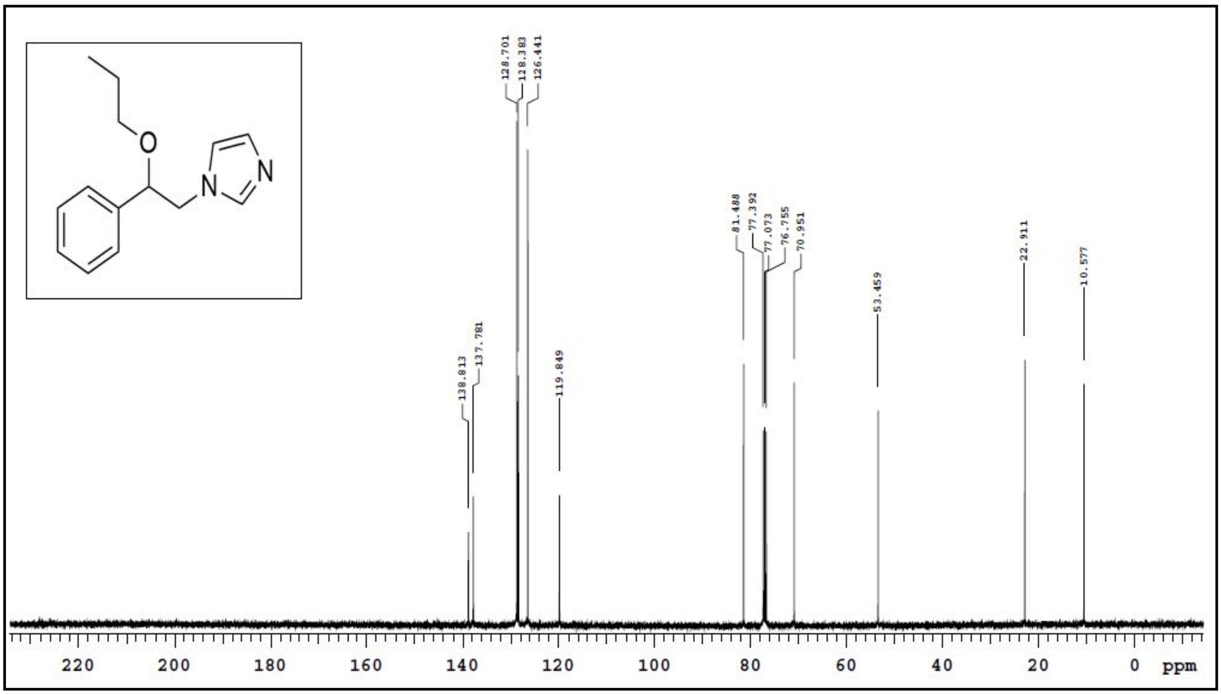

Figure S33. ^{13}C NMR Spectrum ($CDCl_3$) **(8).**

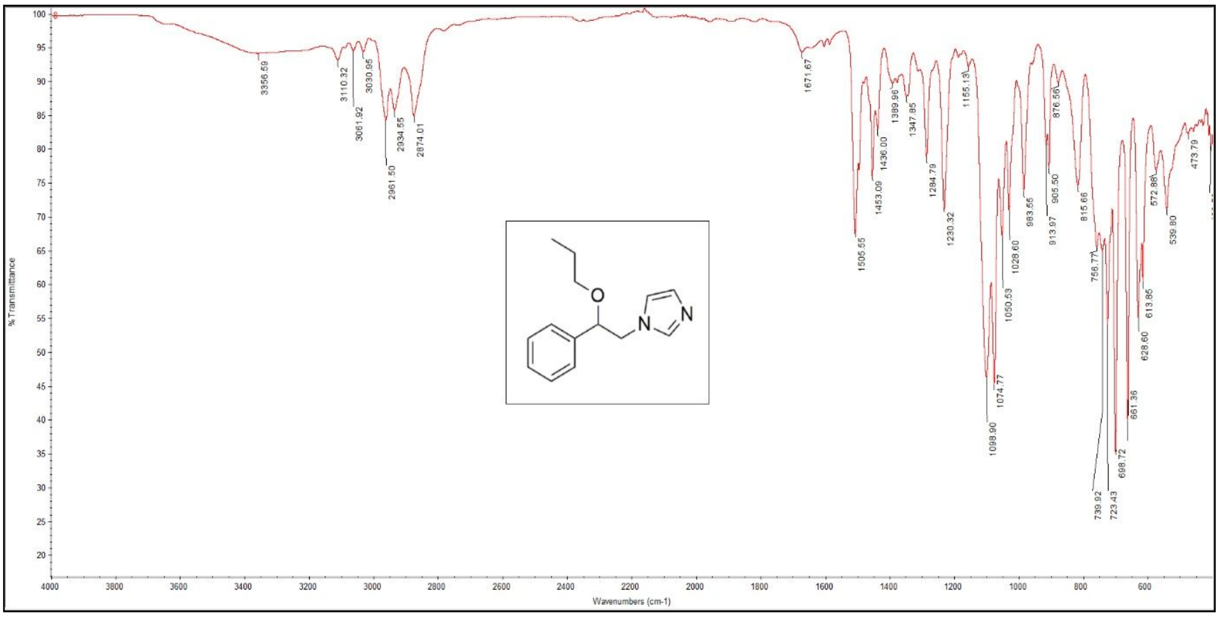

Figure S34. FT-IR Spectrum **(8).**

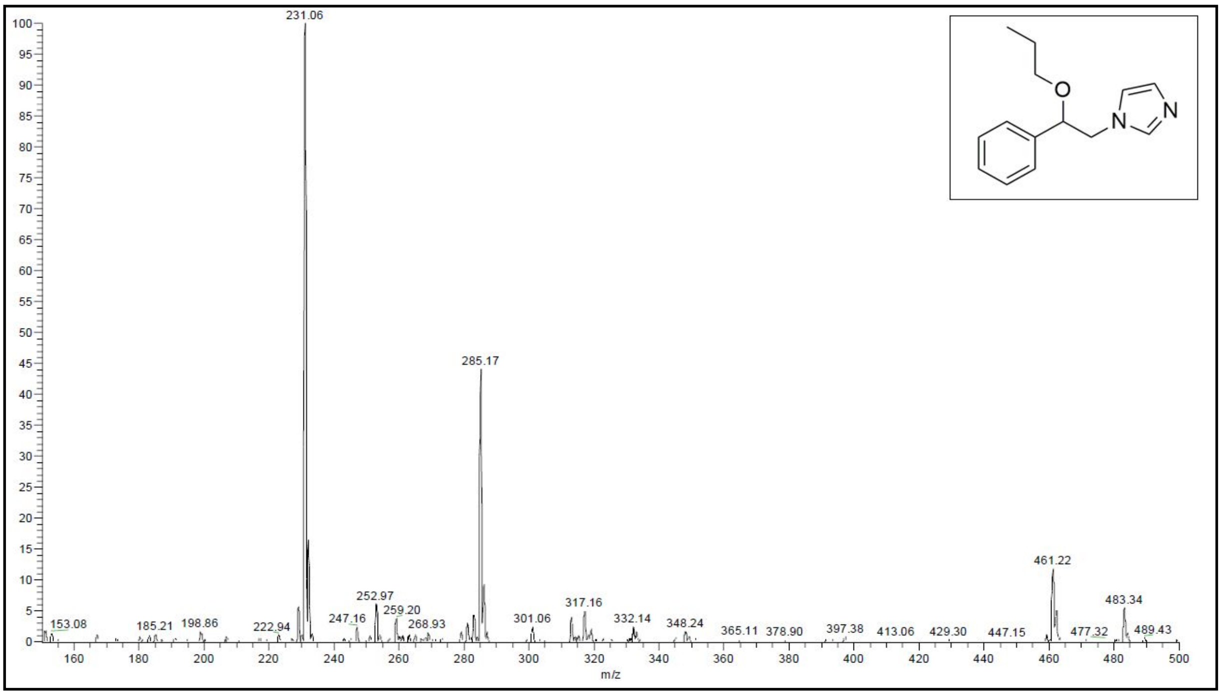

Figure S35. Mass Spectrum **(8).**

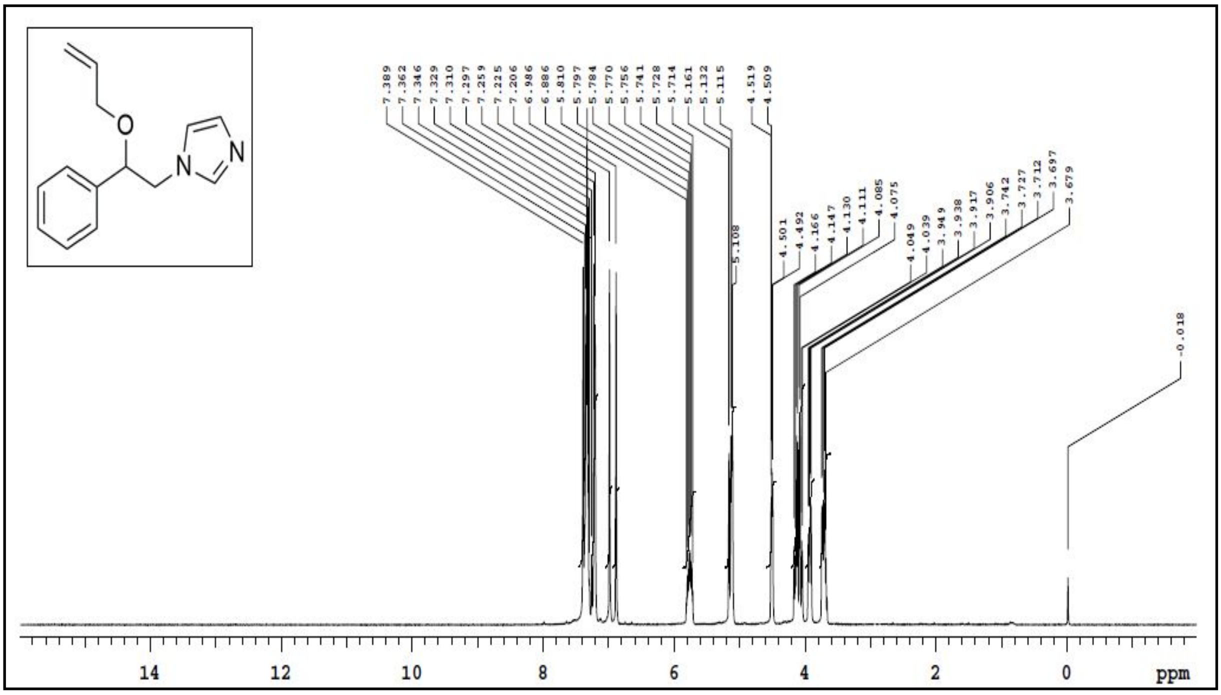

Figure S36. ^{1}H NMR Spectrum ($CDCl_3$) **(9).**

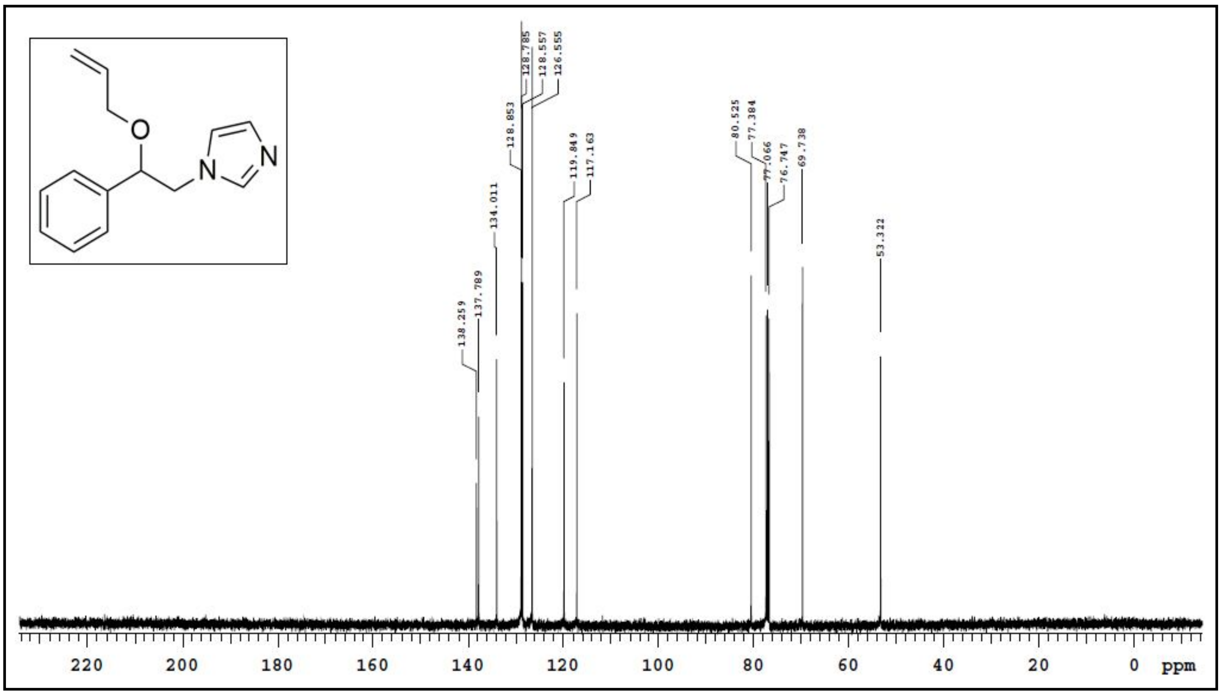

Figure S37. ^{13}C NMR Spectrum ($CDCl_3$) **(9).**

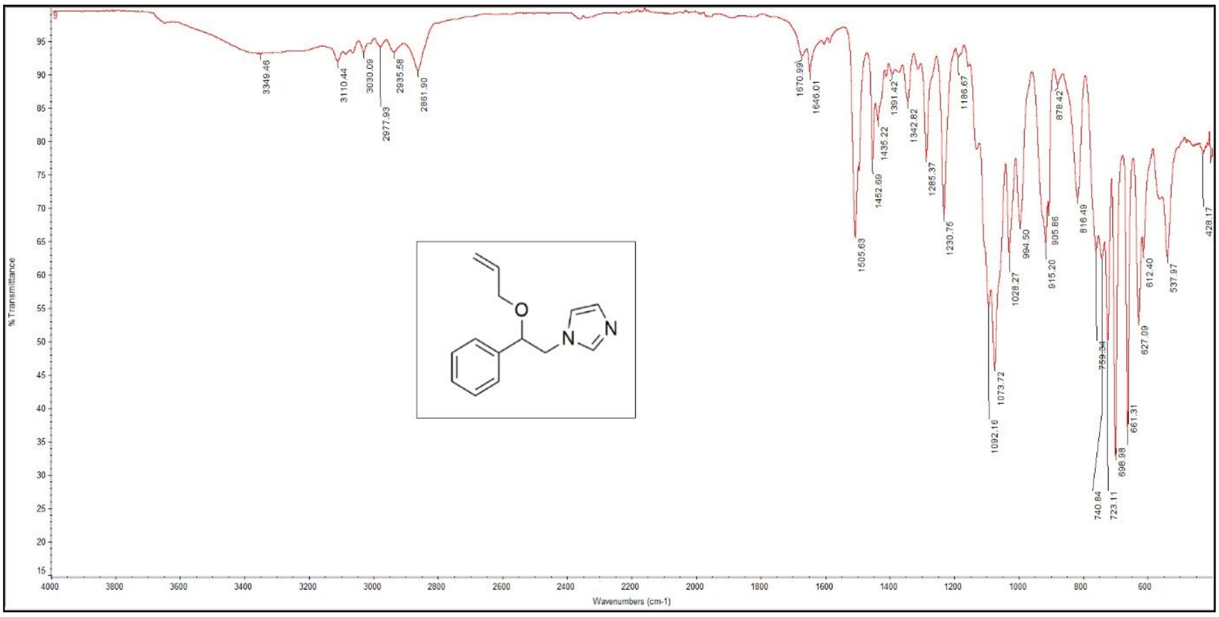

Figure S38. FT-IR Spectrum **(9).**

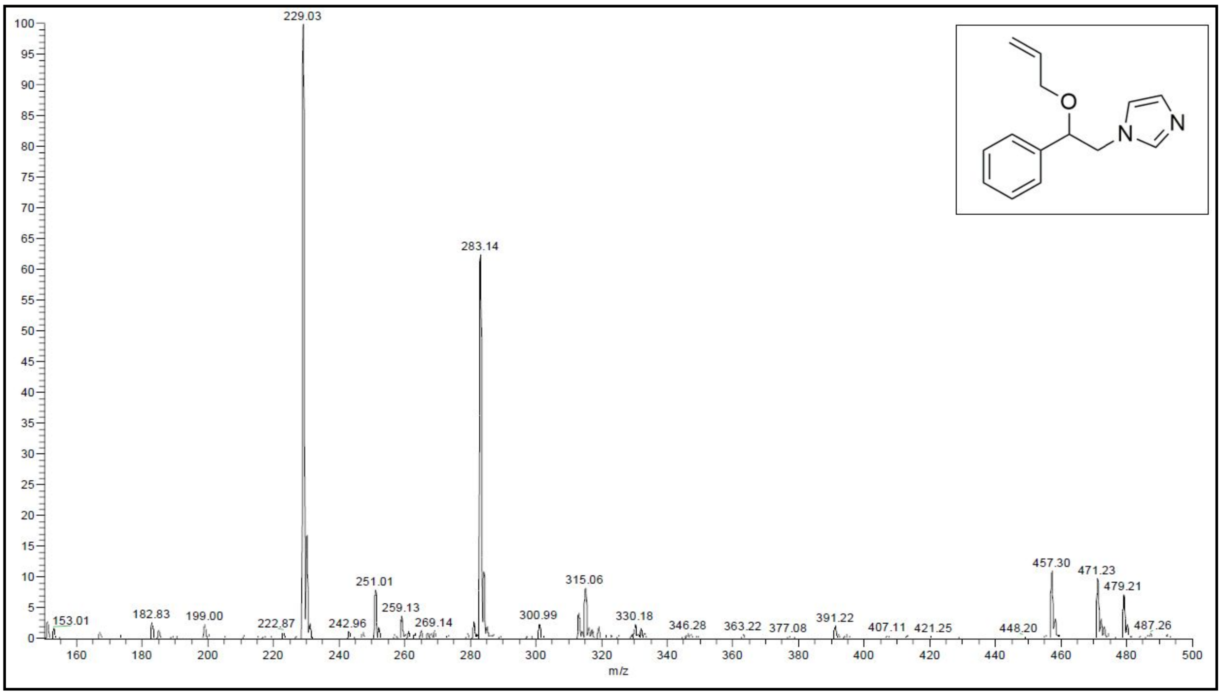

Figure S39. Mass Spectrum **(9).**

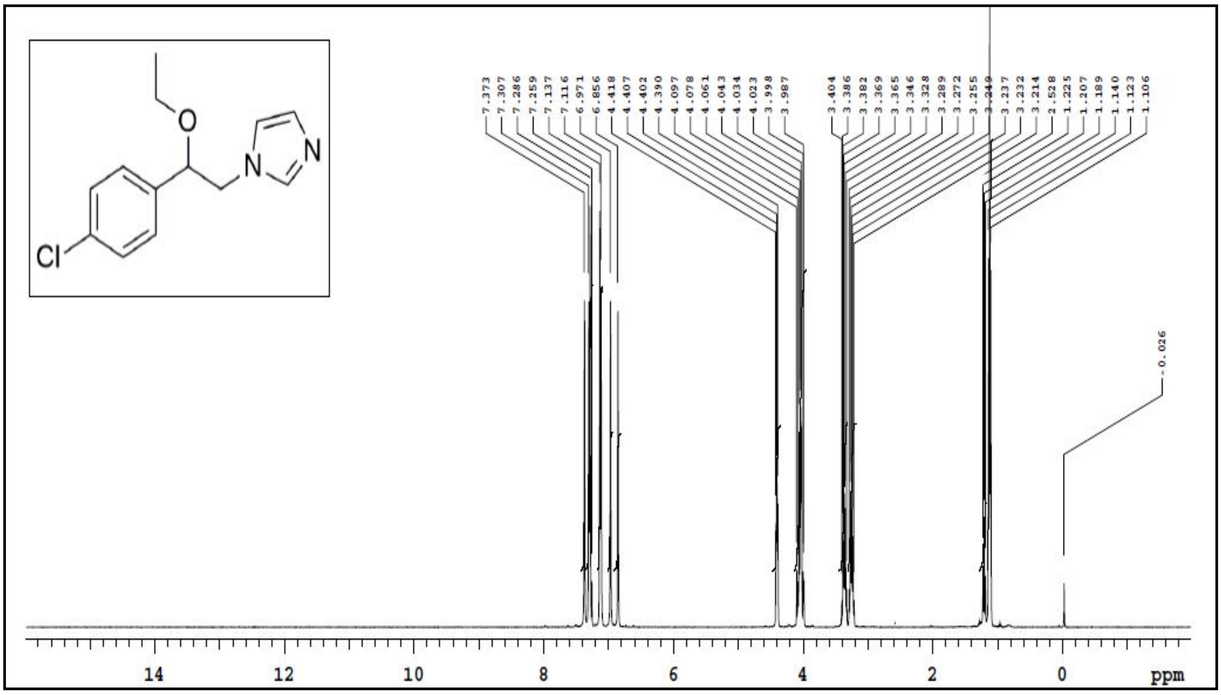

Figure S40. ^{1}H NMR Spectrum ($CDCl_3$) **(10).**

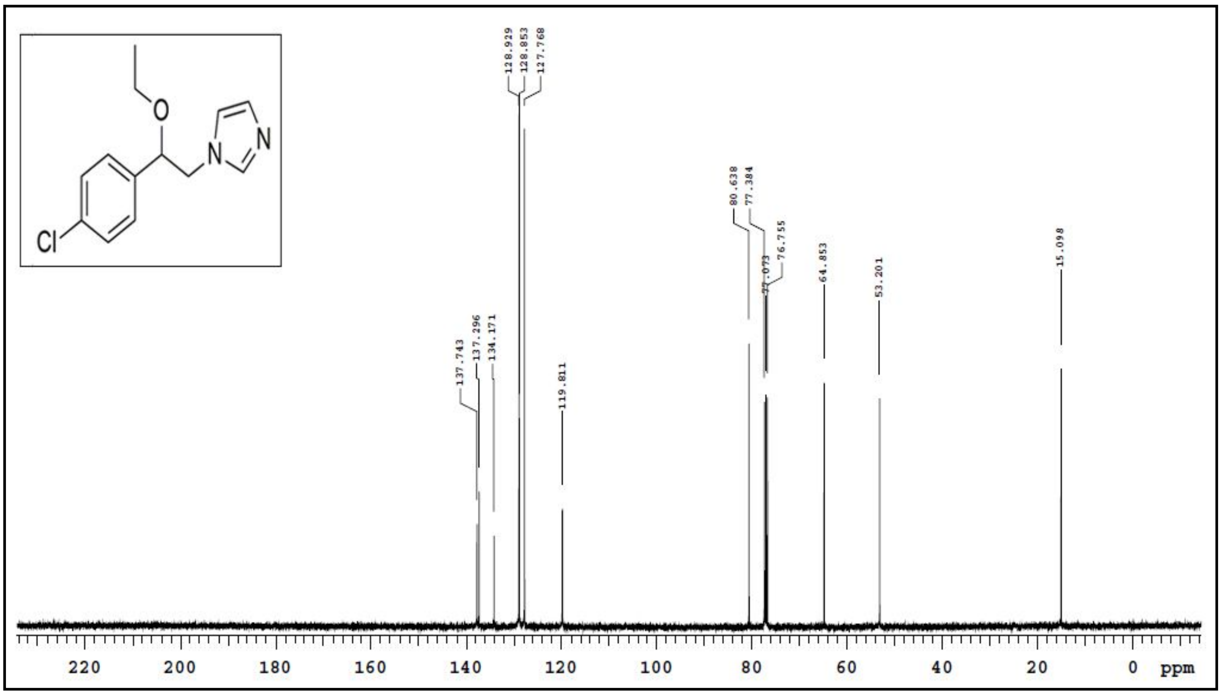

Figure S41. ^{13}C NMR Spectrum ($CDCl_3$) **(10).**

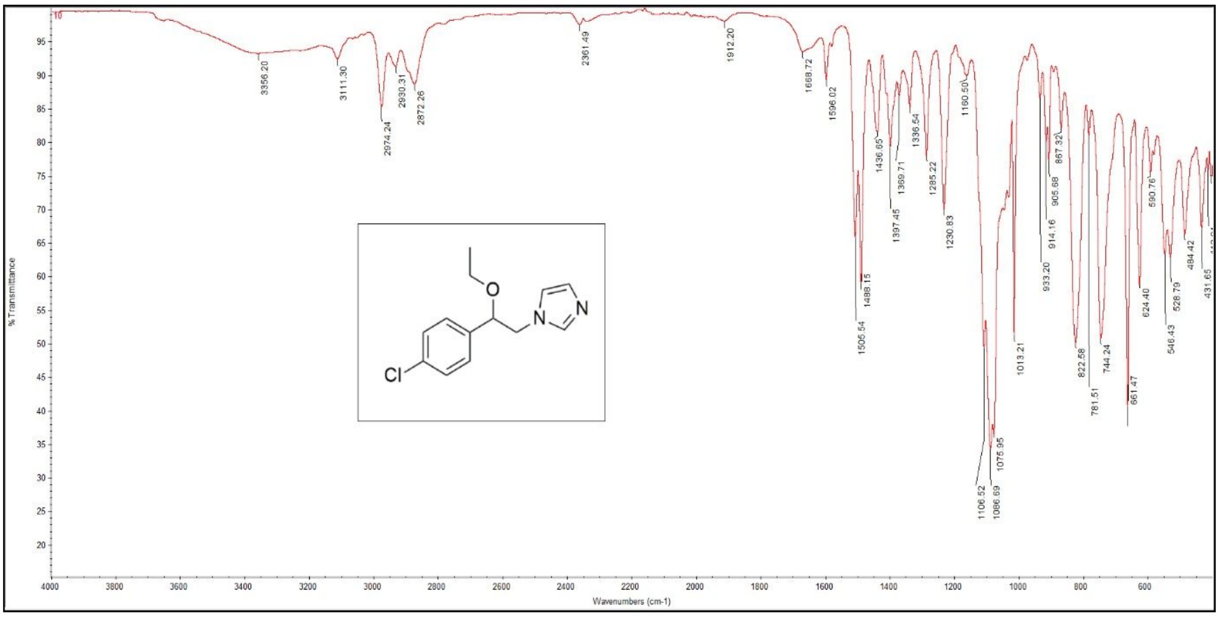

Figure S42. FT-IR Spectrum **(10).**

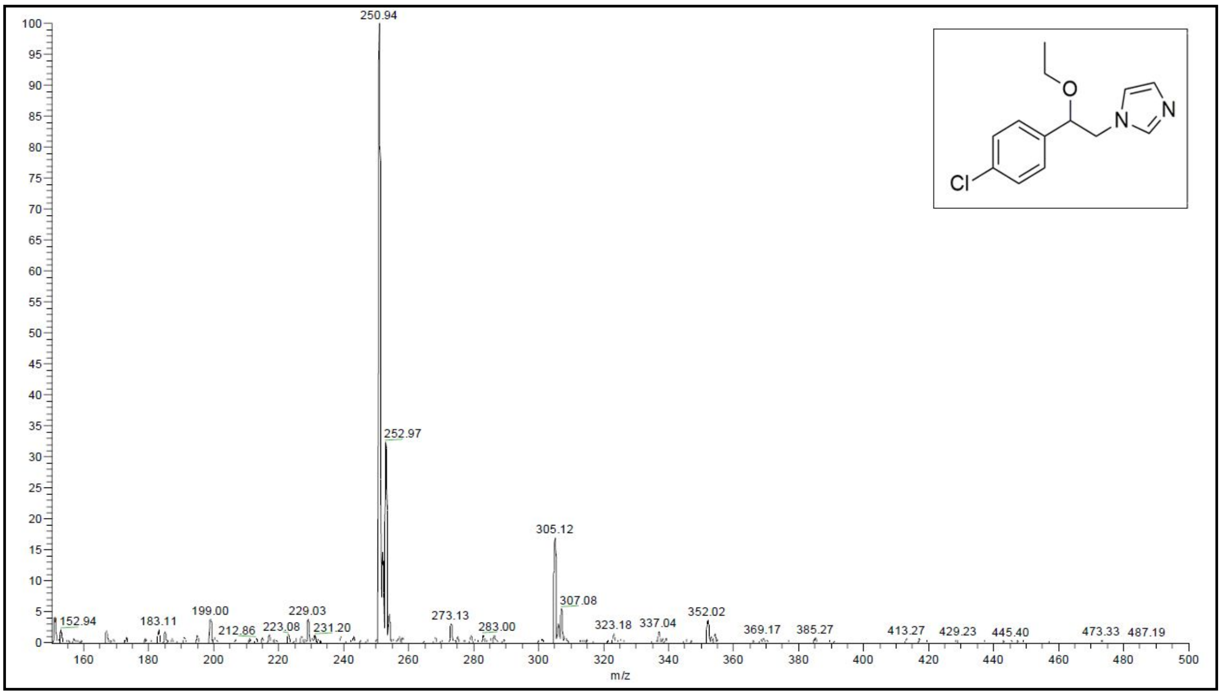

Figure S43. Mass Spectrum **(10).**

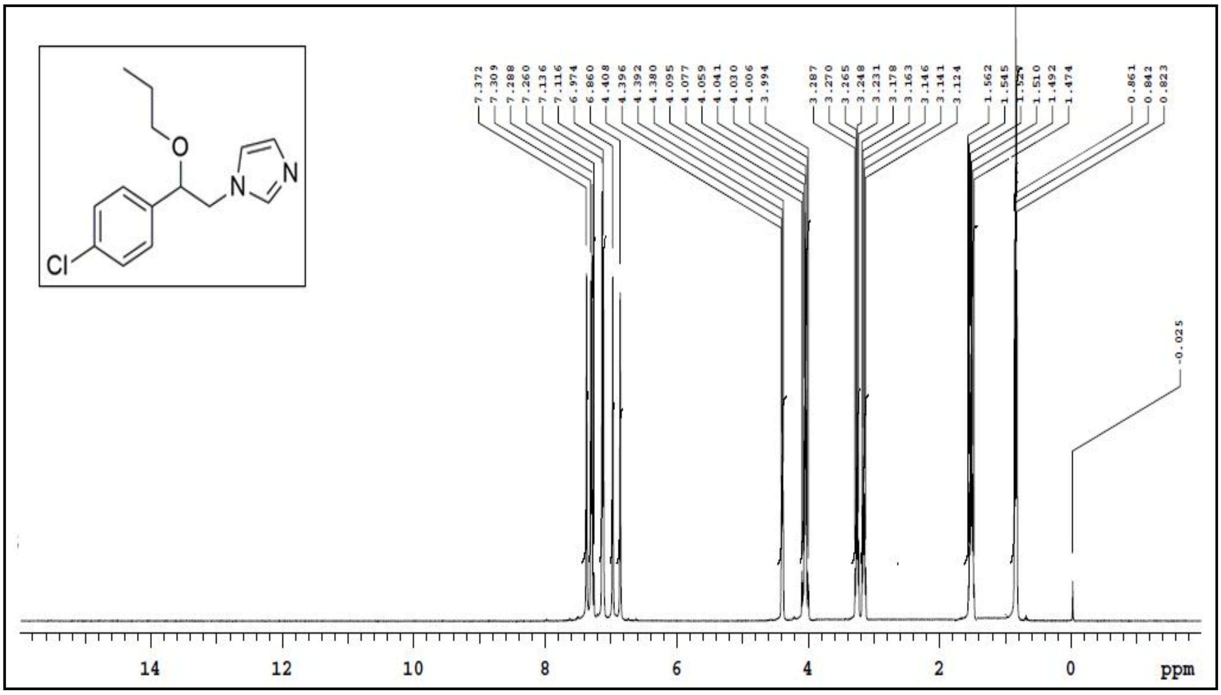

Figure S44. ^{1}H NMR Spectrum ($CDCl_3$) **(11).**

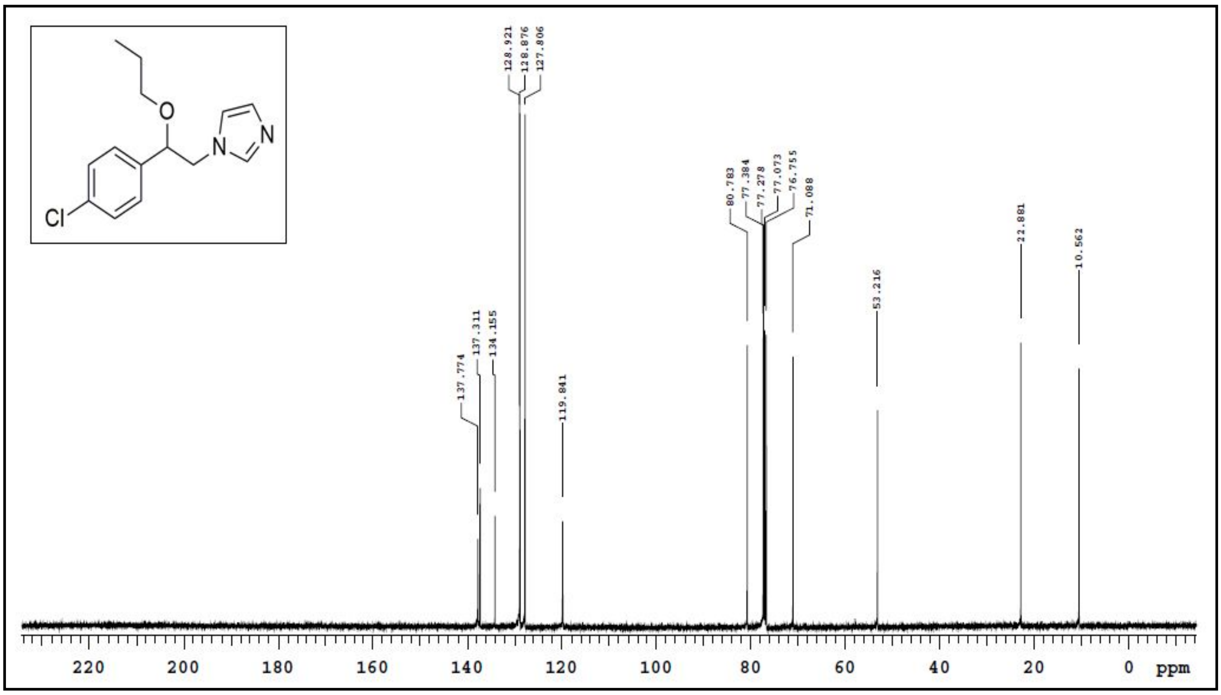

Figure S45. ^{13}C NMR Spectrum ($CDCl_3$) **(11).**

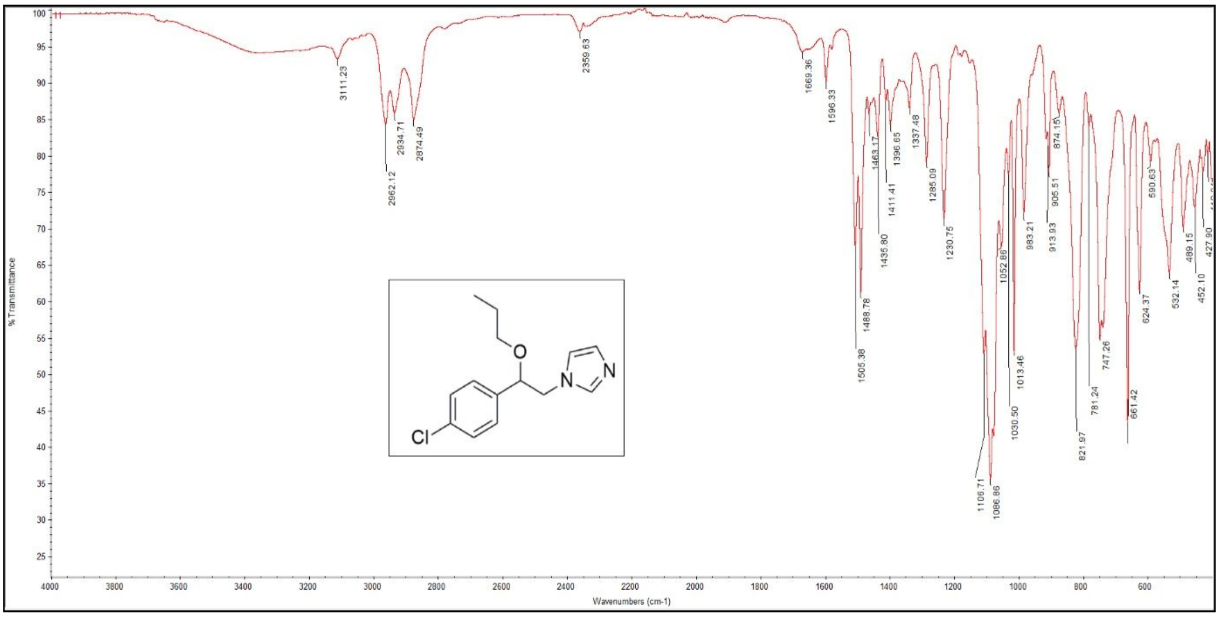

Figure S46. FT-IR Spectrum **(11).**

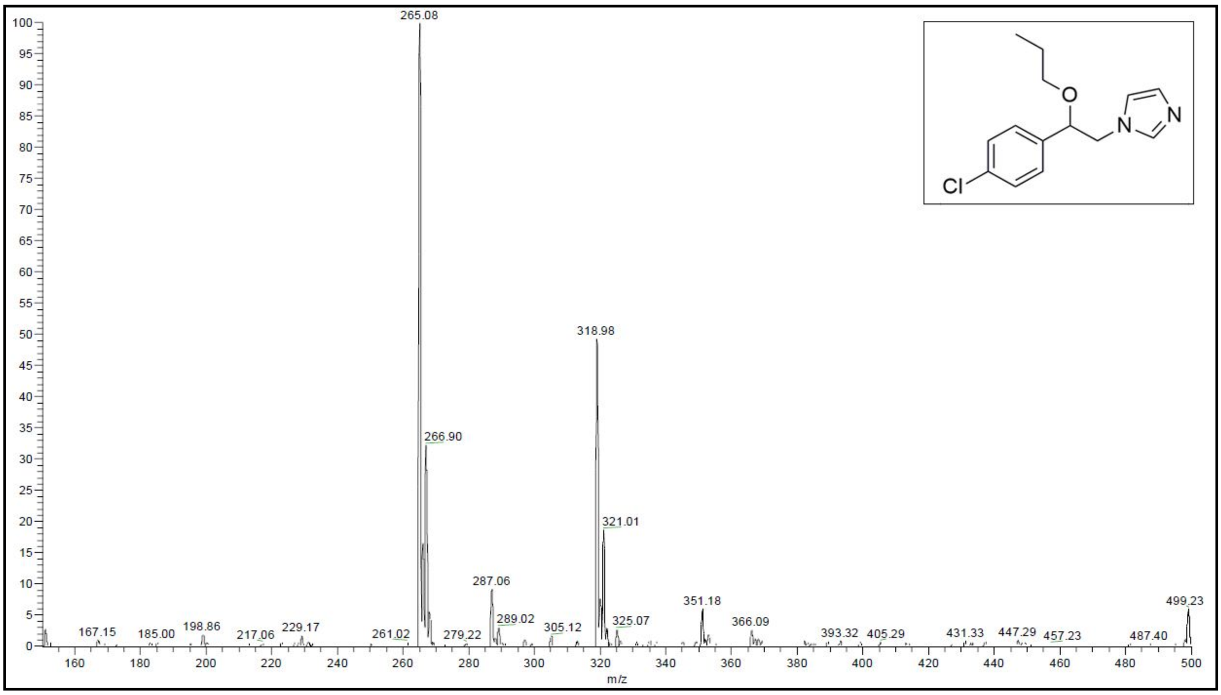

Figure S47. Mass Spectrum **(11).**

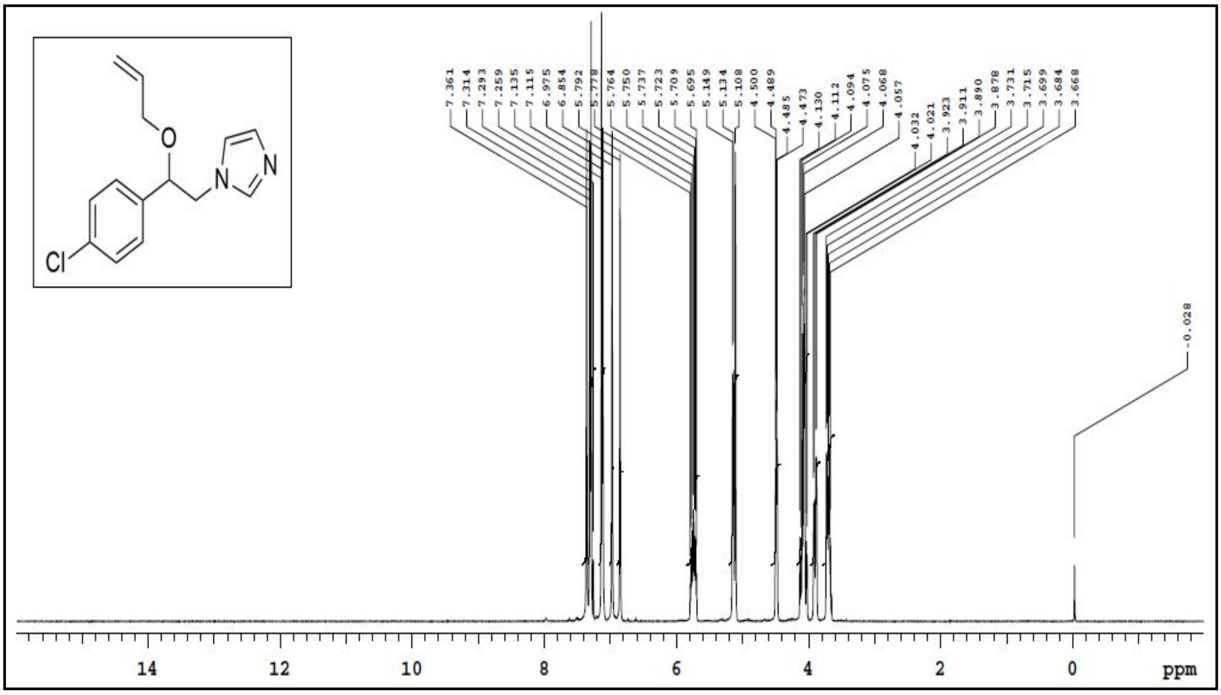

Figure S48. ^{1}H NMR Spectrum ($CDCl_3$) **(12).**

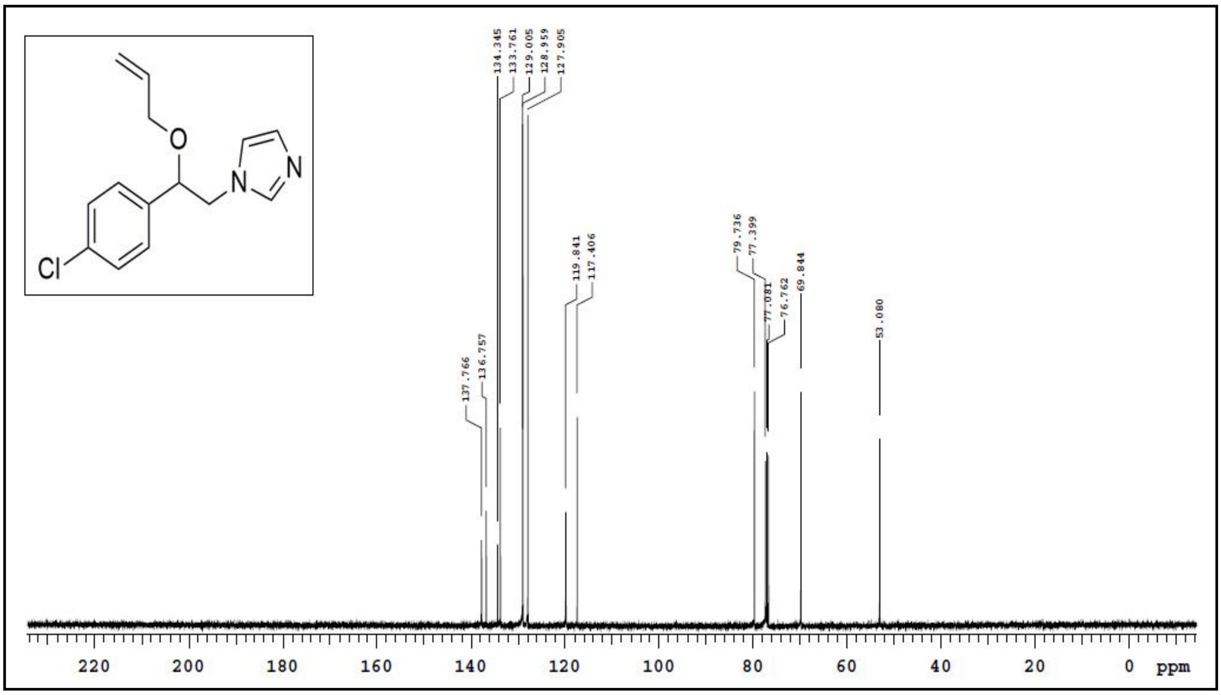

Figure S49. ^{13}C NMR Spectrum ($CDCl_3$) **(12).**

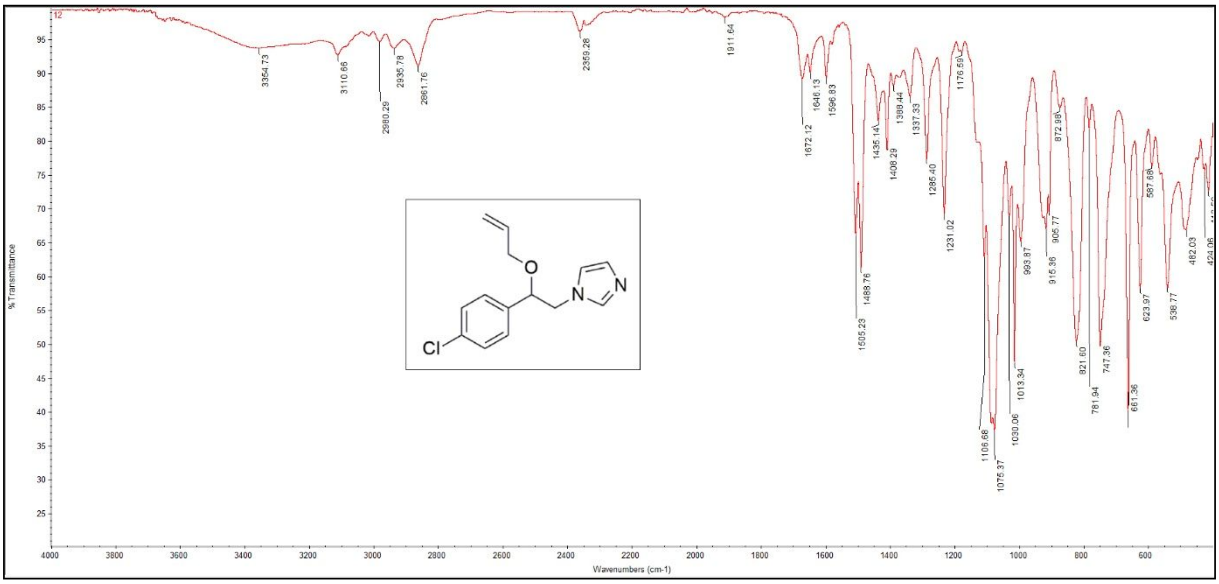

Figure S50. FT-IR Spectrum **(12).**

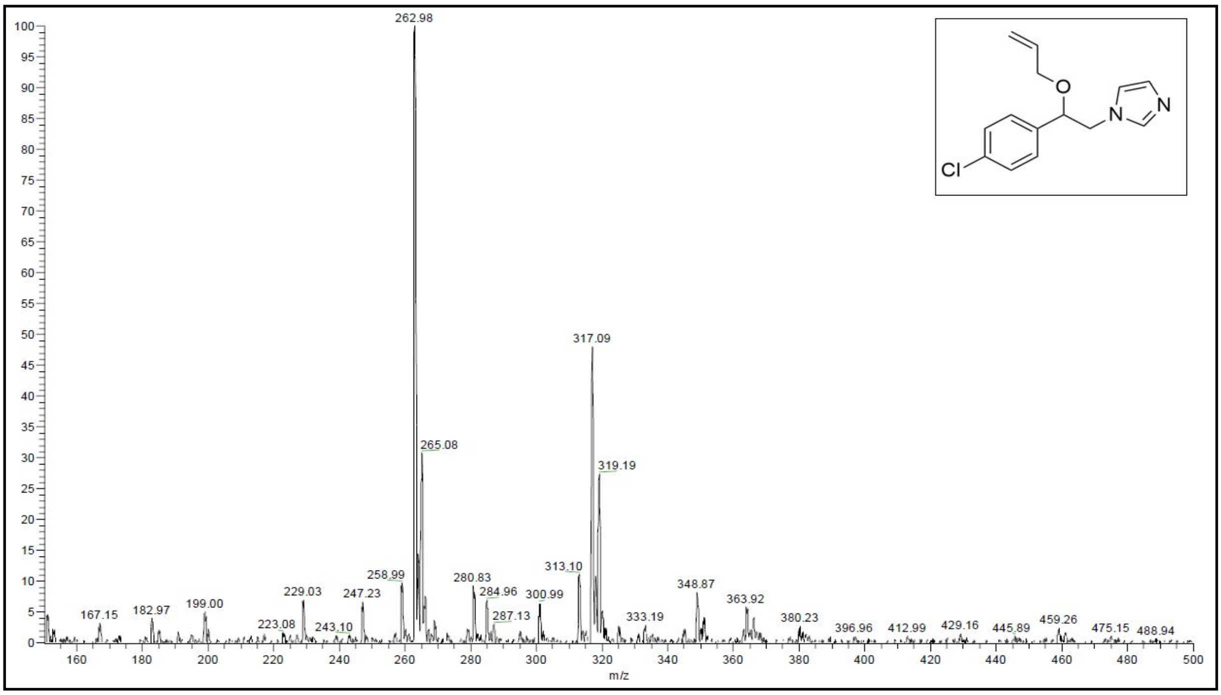

Figure S51. Mass Spectrum **(12).**

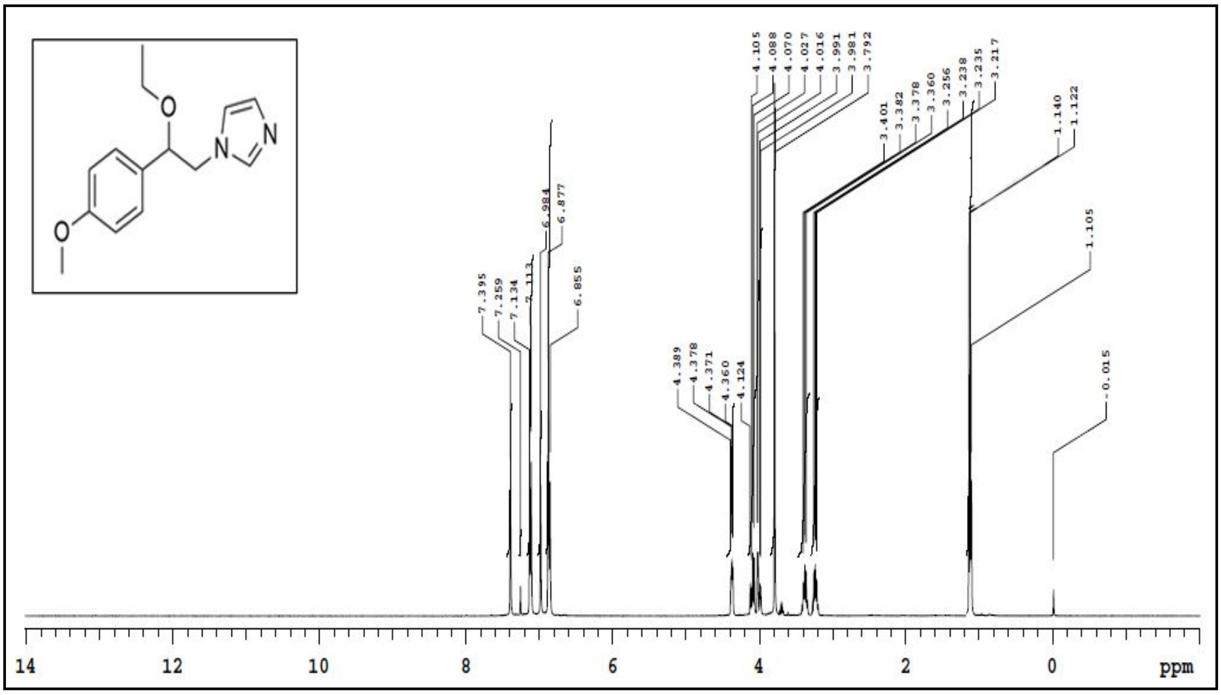

Figure S52. ^{1}H NMR Spectrum ($CDCl_3$) **(13).**

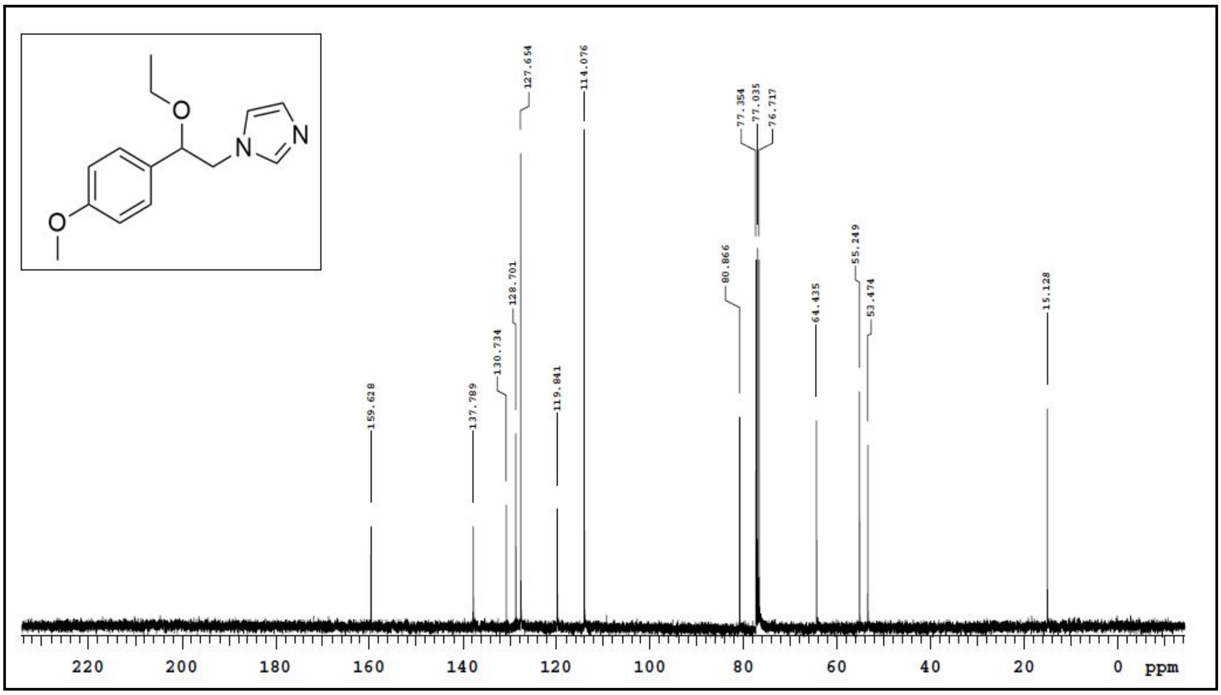

Figure S53. ^{13}C NMR Spectrum ($CDCl_3$) **(13).**

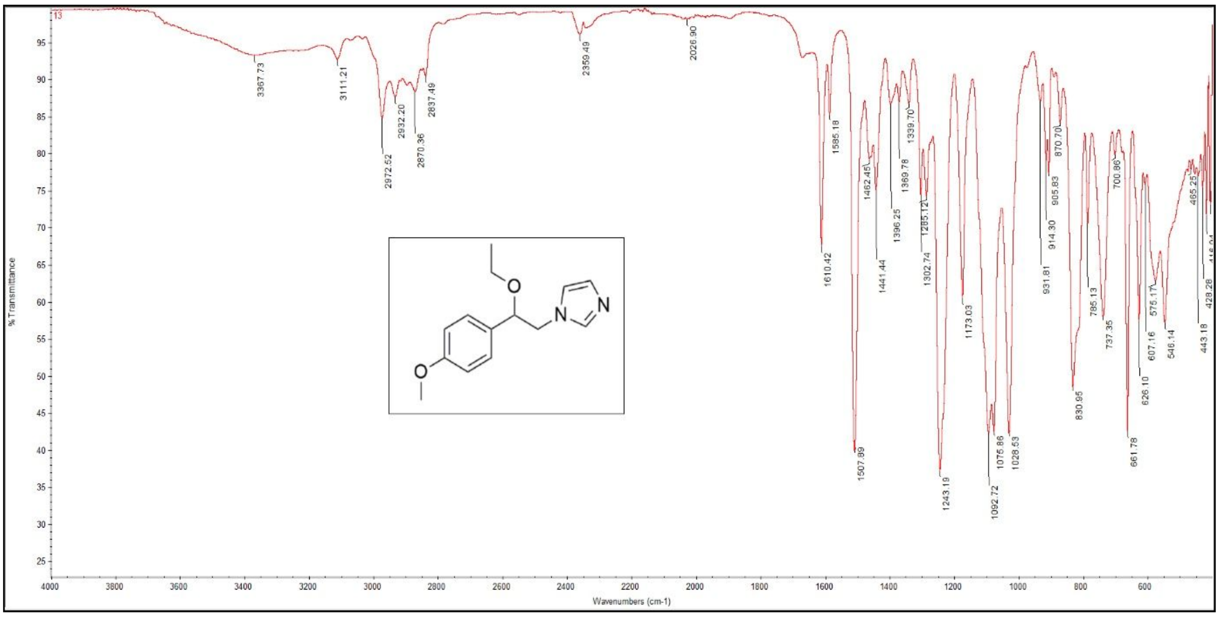

Figure S54. FT-IR Spectrum **(13).**

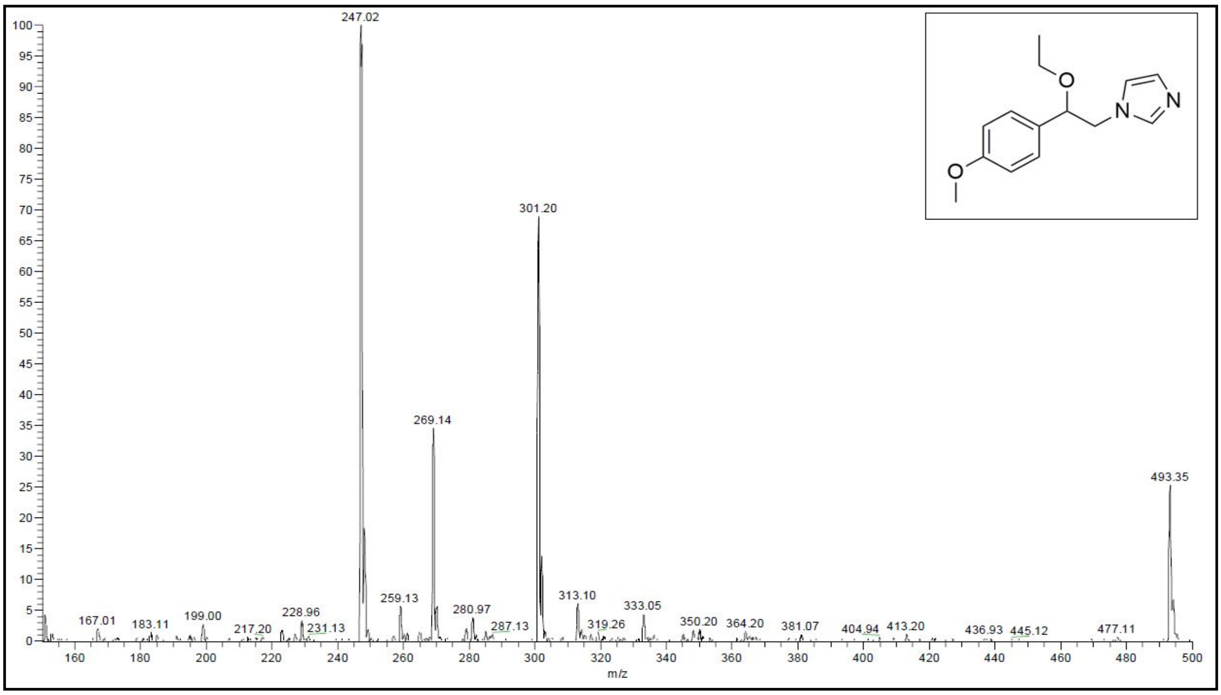

Figure S55. Mass Spectrum **(13).**

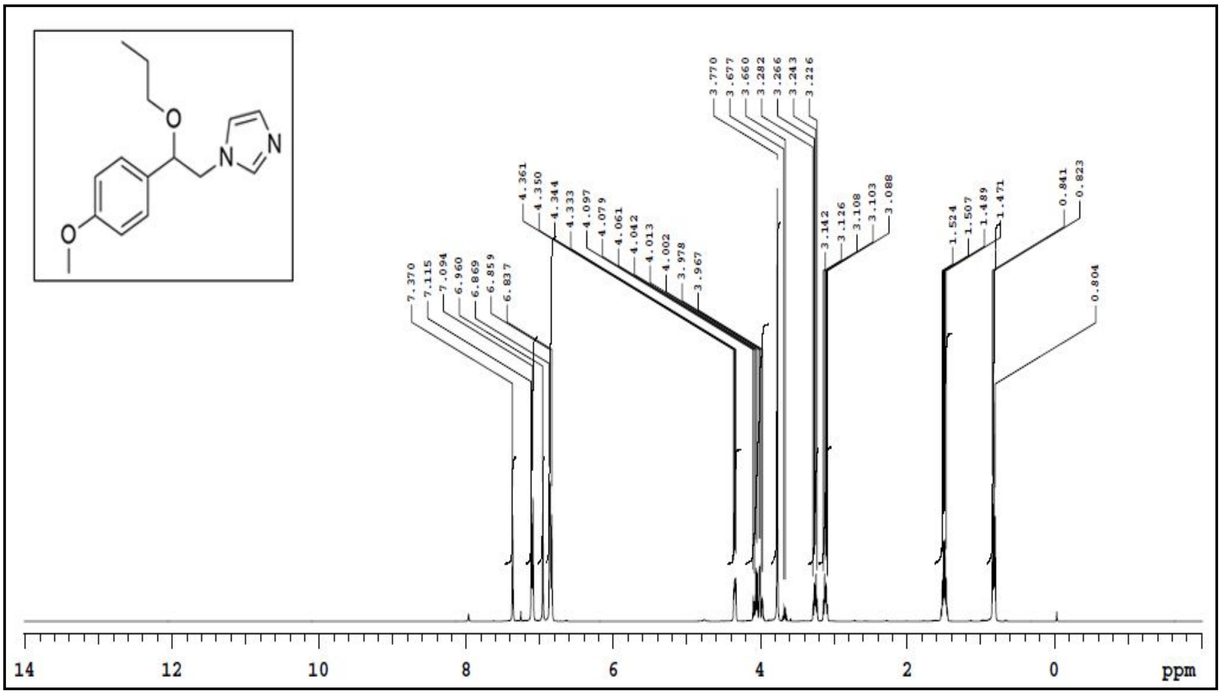

Figure S56. ^{1}H NMR Spectrum ($CDCl_3$) **(14).**

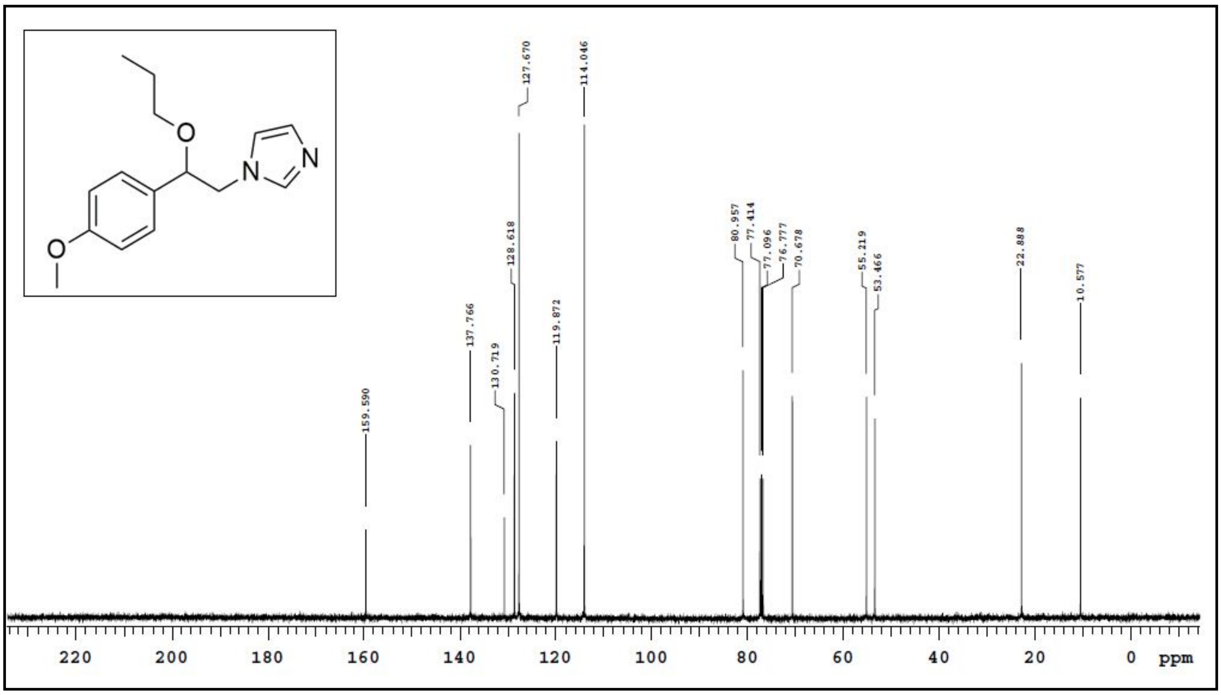

Figure S57. ^{13}C NMR Spectrum ($CDCl_3$) **(14).**

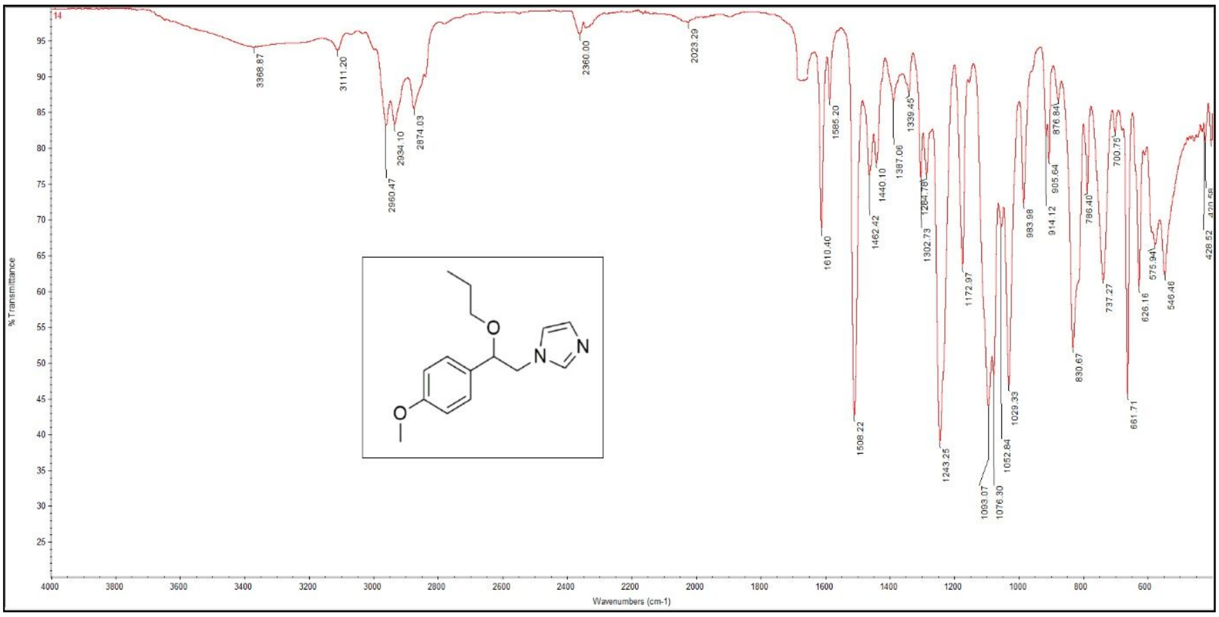

Figure S58. FT-IR Spectrum **(14).**

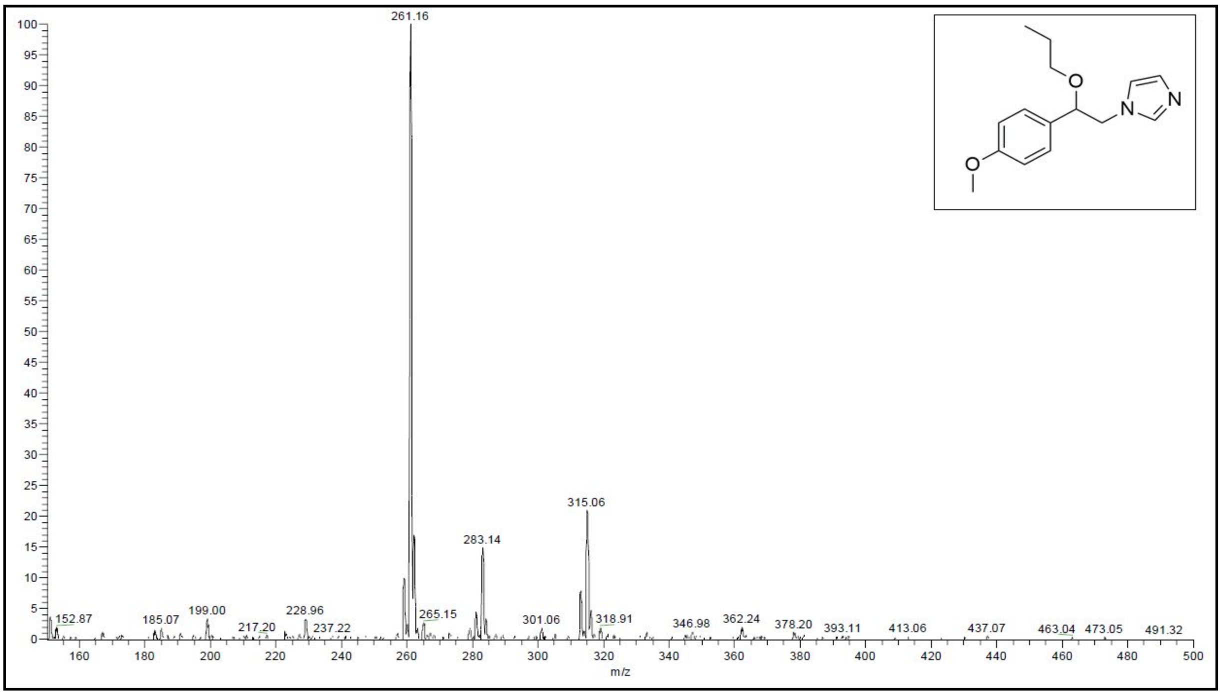

Figure S59. Mass Spectrum **(14).**

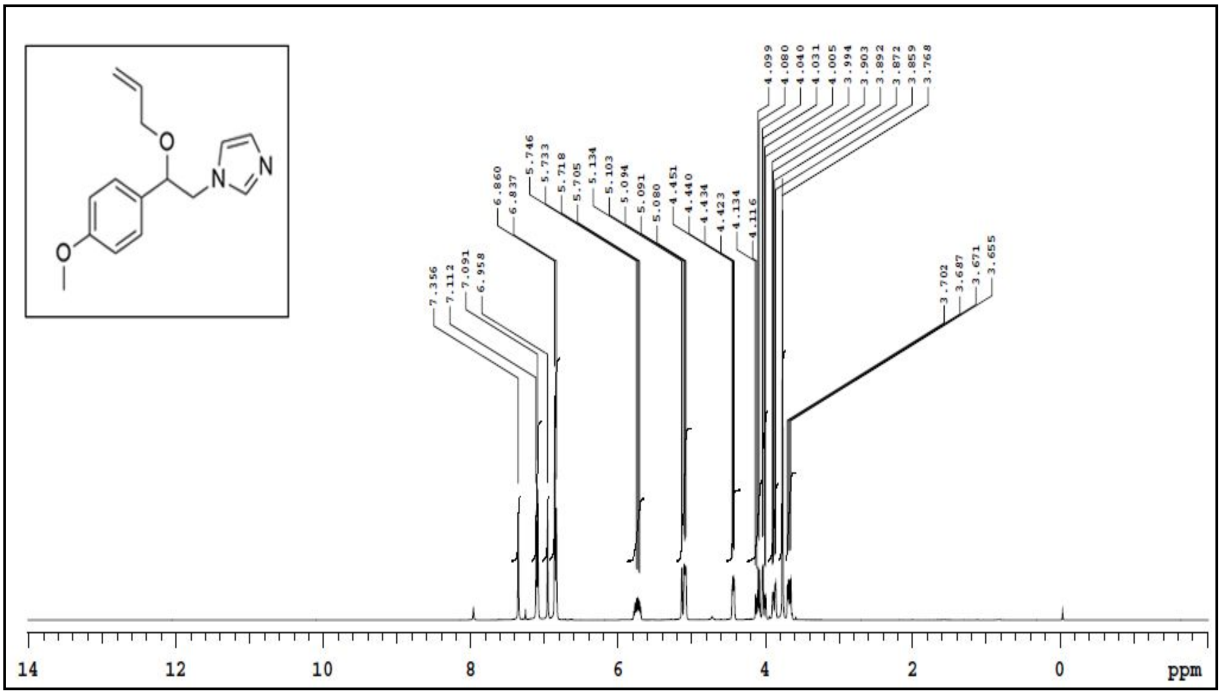

Figure S60. ^{1}H NMR Spectrum ($CDCl_3$) **(15).**

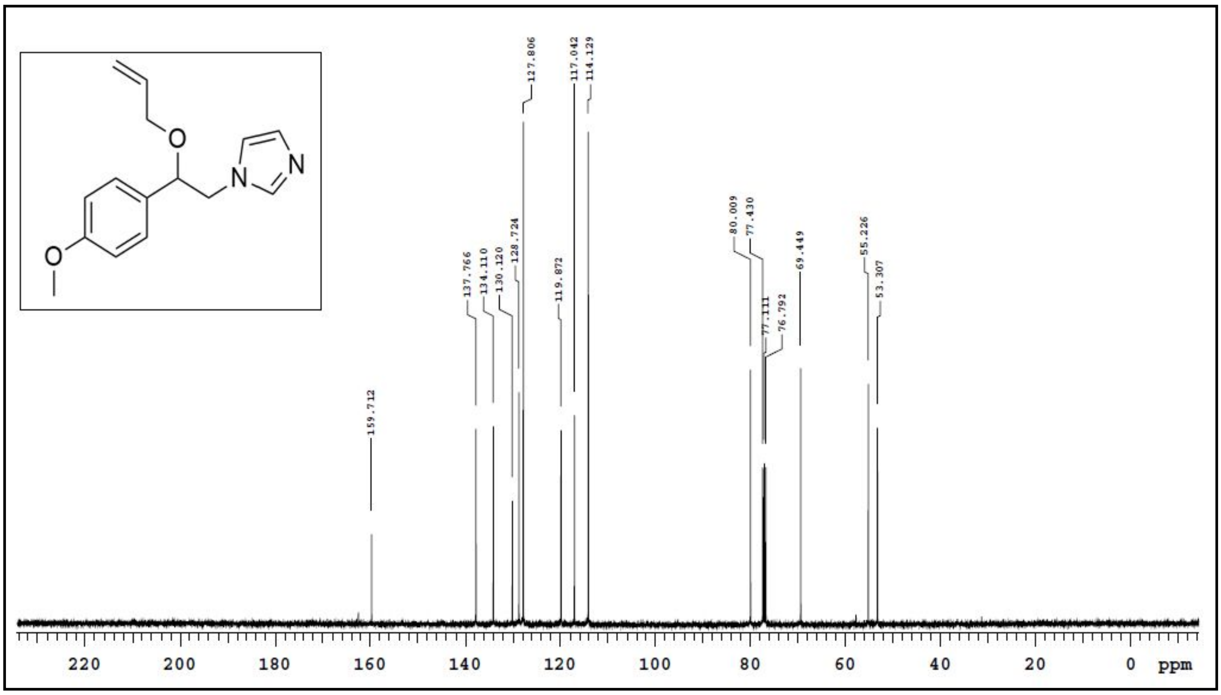

Figure S61. ^{13}C NMR Spectrum ($CDCl_3$) **(15).**

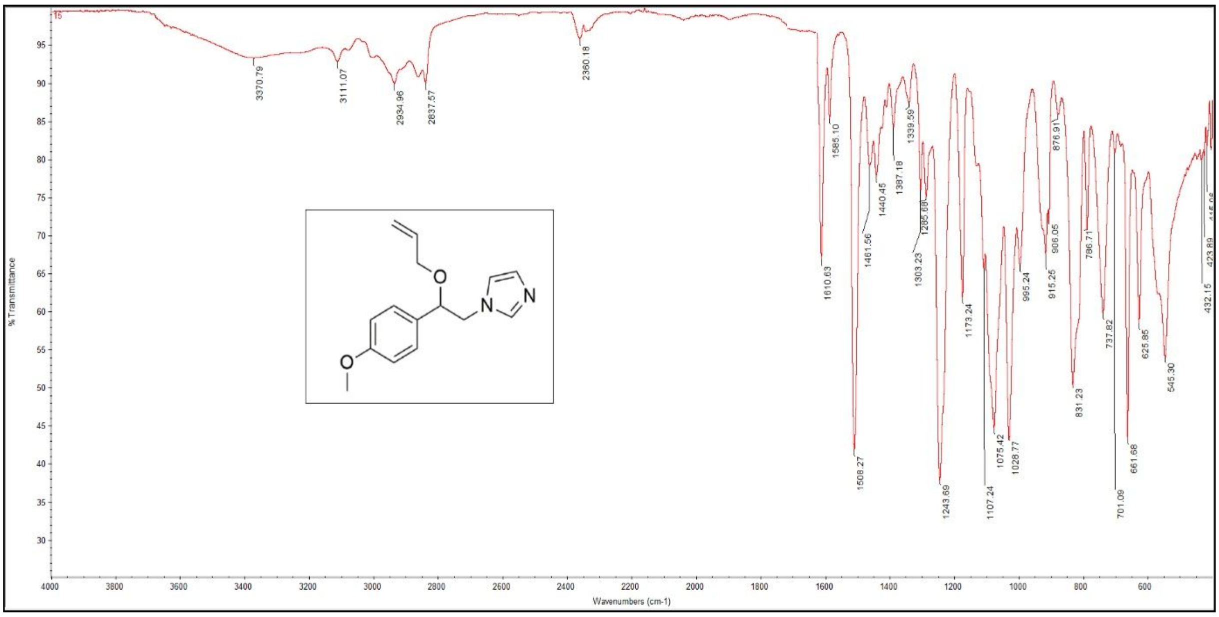

Figure S62. FT-IR Spectrum **(15).**

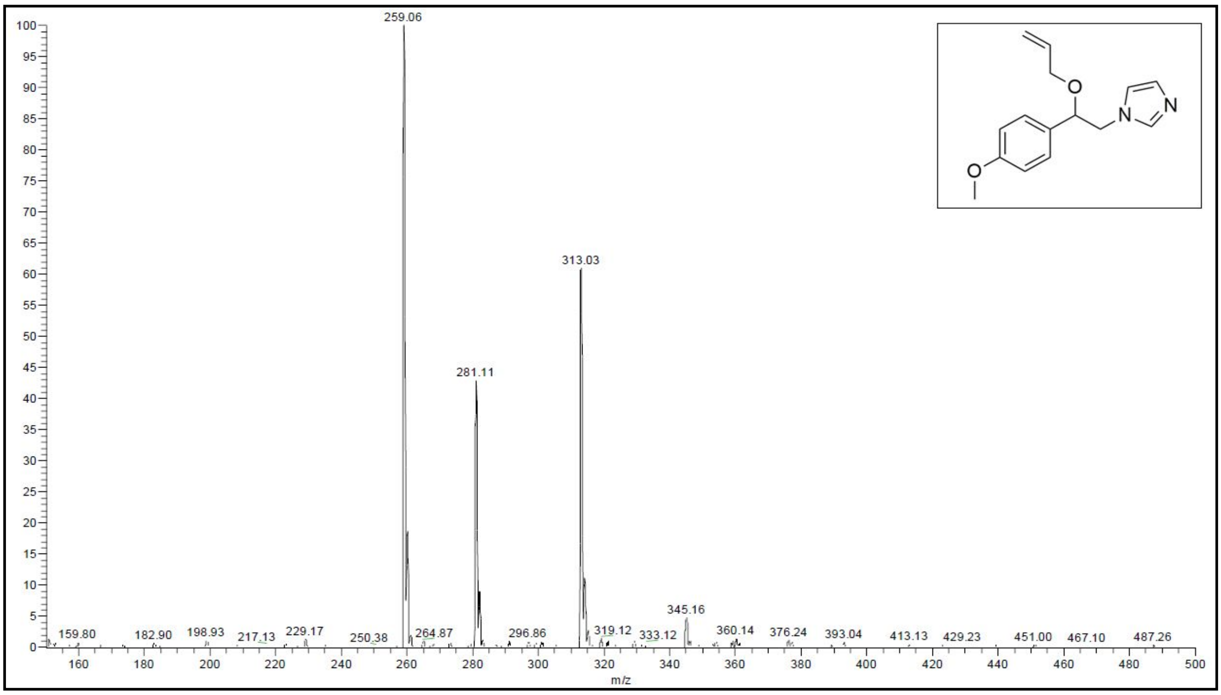

Figure S63. Mass Spectrum **(15).**

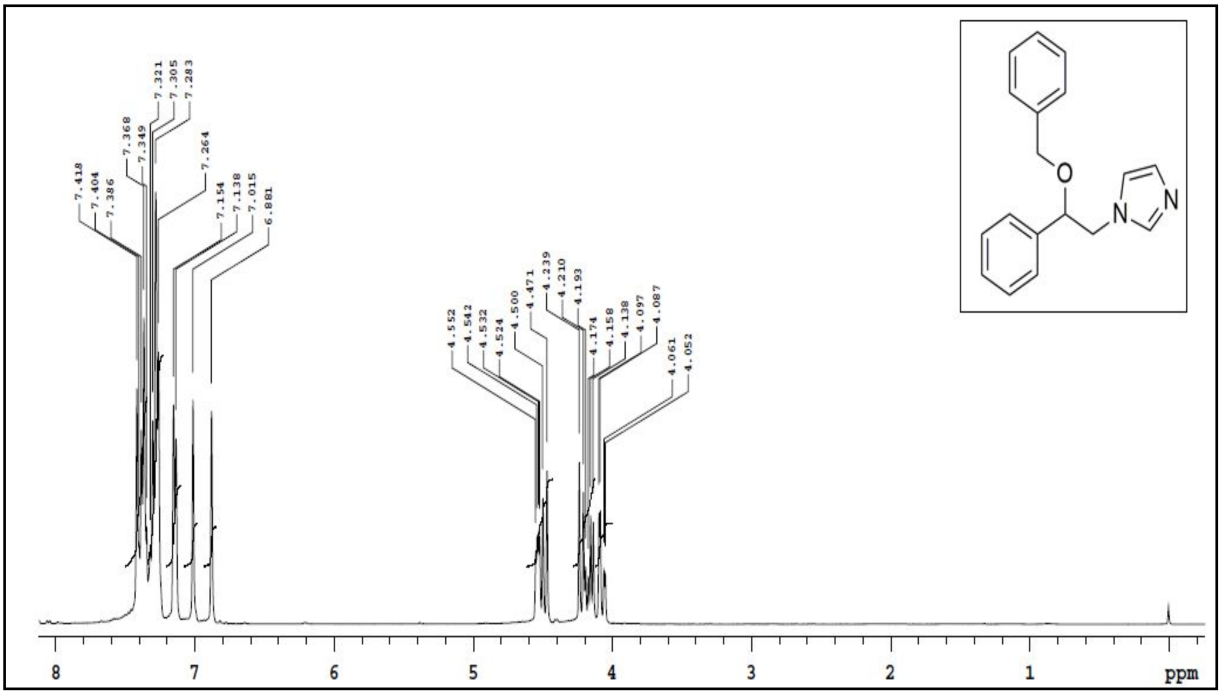

Figure S64. ^{1}H NMR Spectrum ($CDCl_3$) **(16).**

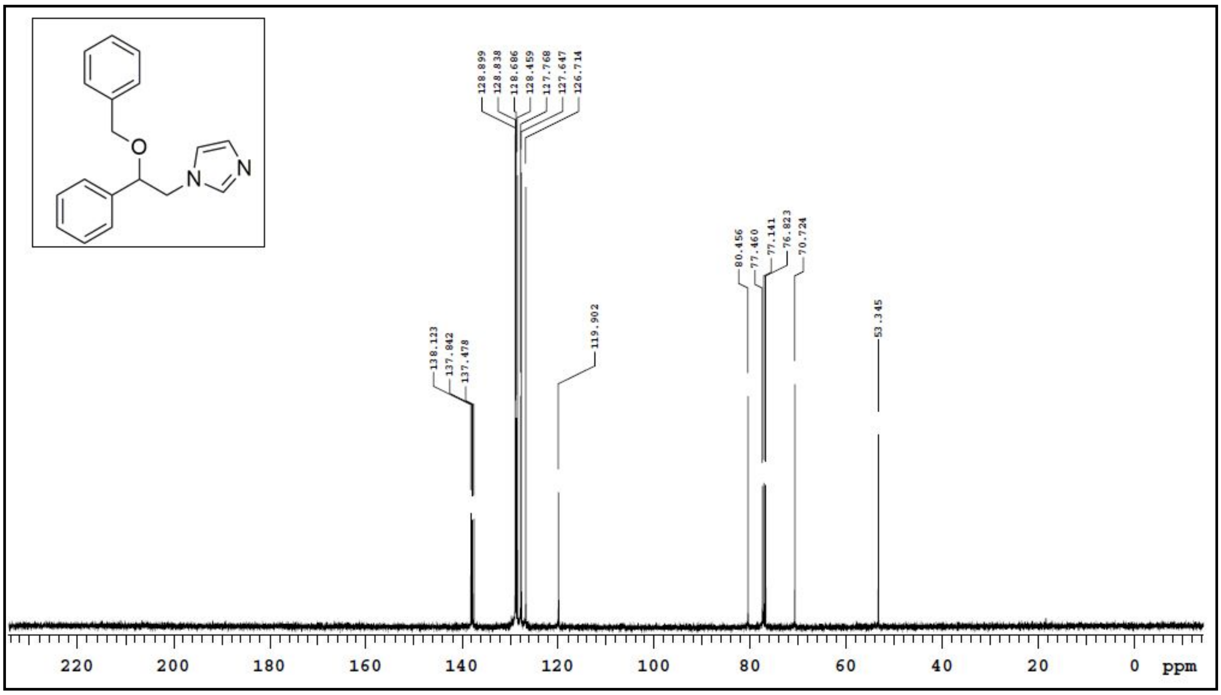

Figure S65. ^{13}C NMR Spectrum ($CDCl_3$) **(16).**

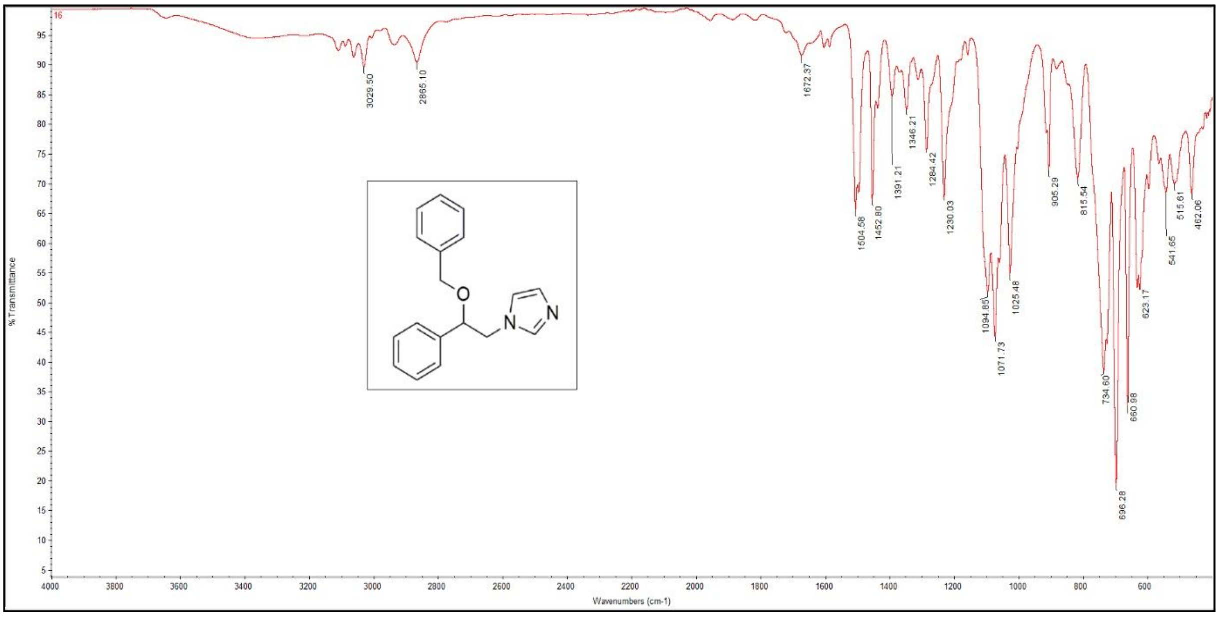

Figure S66. FT-IR Spectrum **(16).**

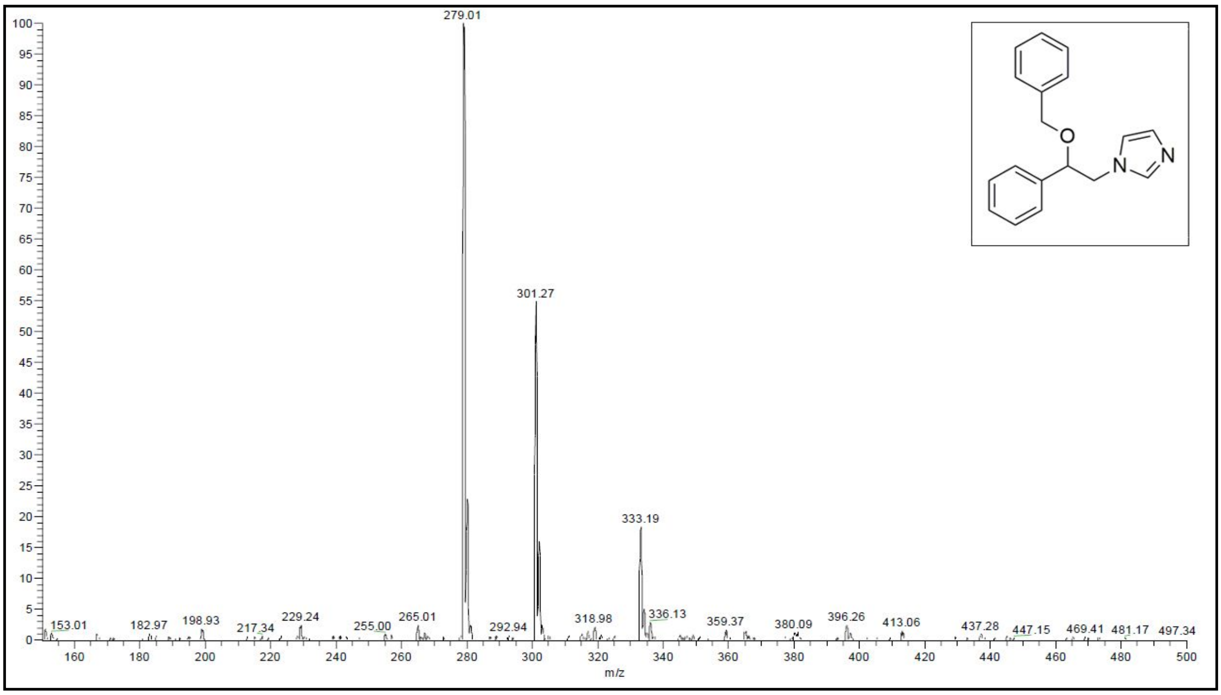

Figure S67. Mass Spectrum **(16).**

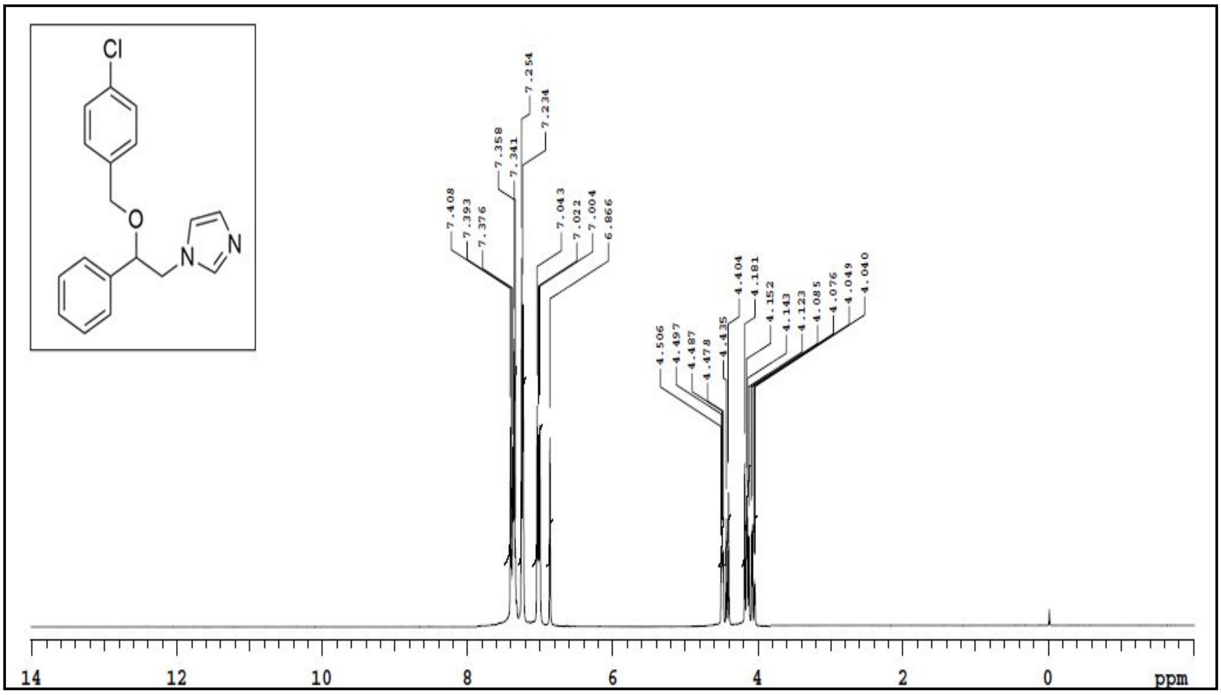

Figure S68. ^{1}H NMR Spectrum ($CDCl_3$) **(17).**

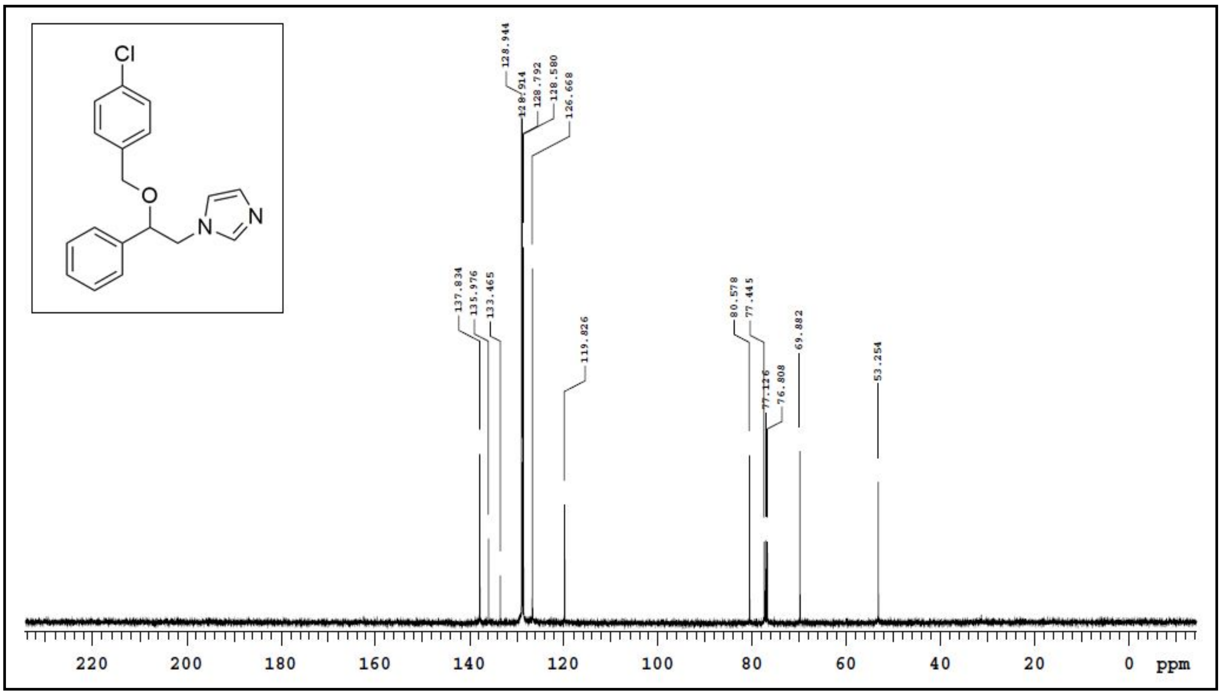

Figure S69. ^{13}C NMR Spectrum ($CDCl_3$) **(17).**

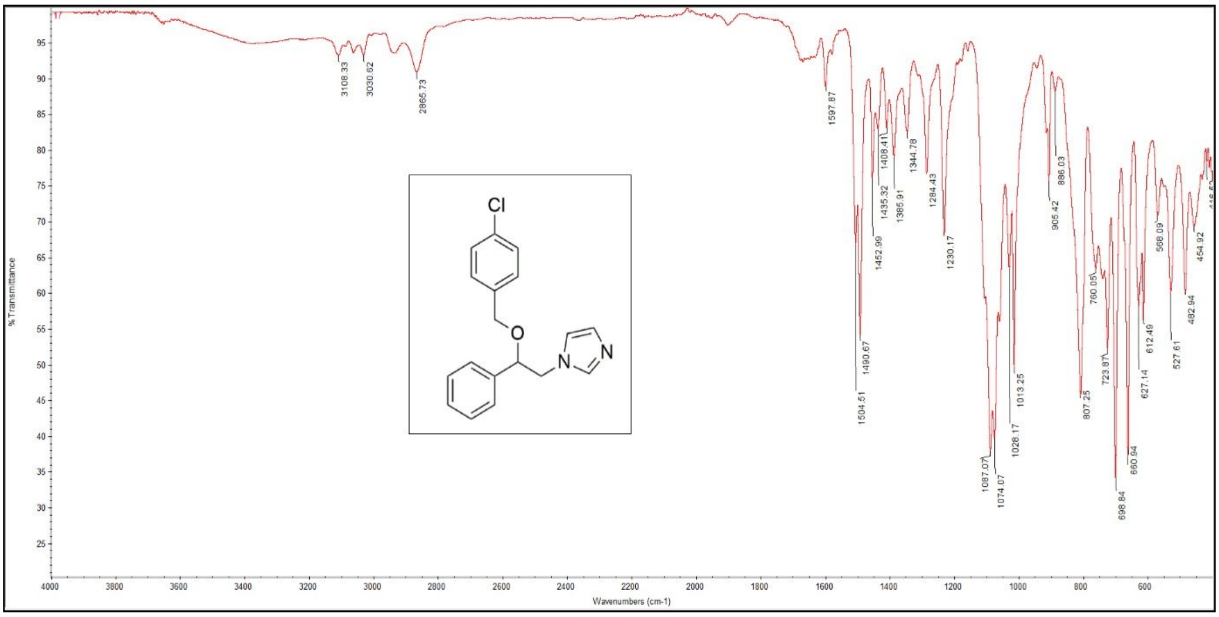

Figure S70. FT-IR Spectrum **(17).**

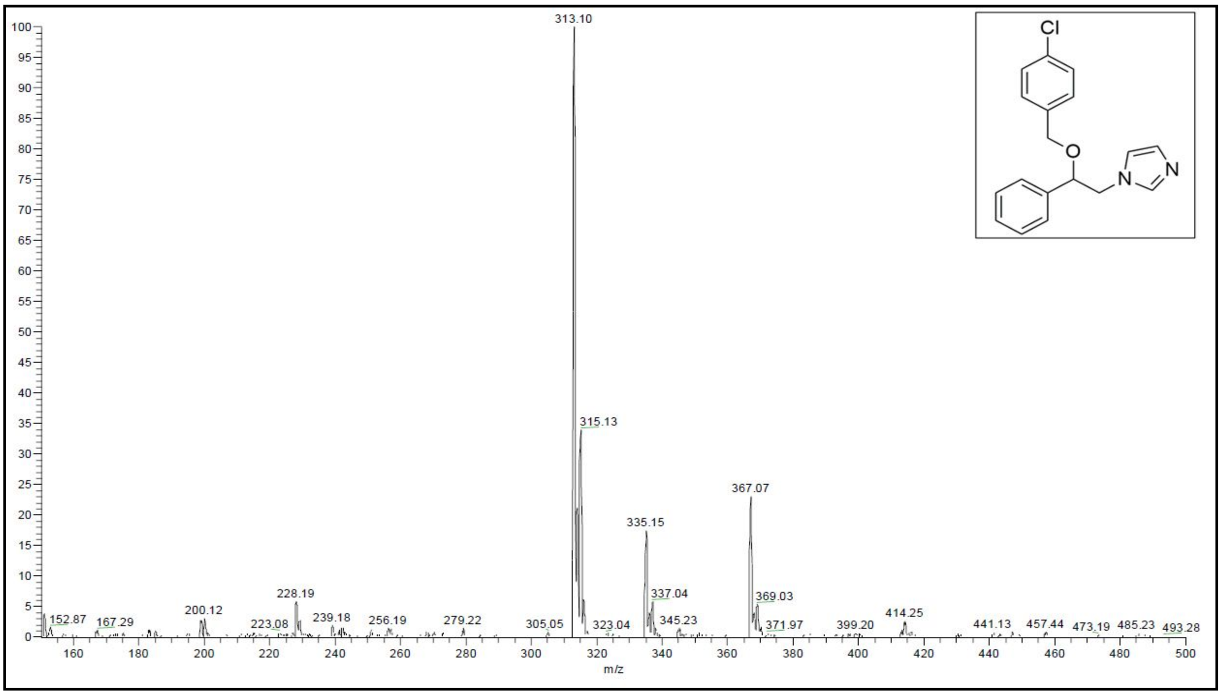

Figure S71. Mass Spectrum **(17).**

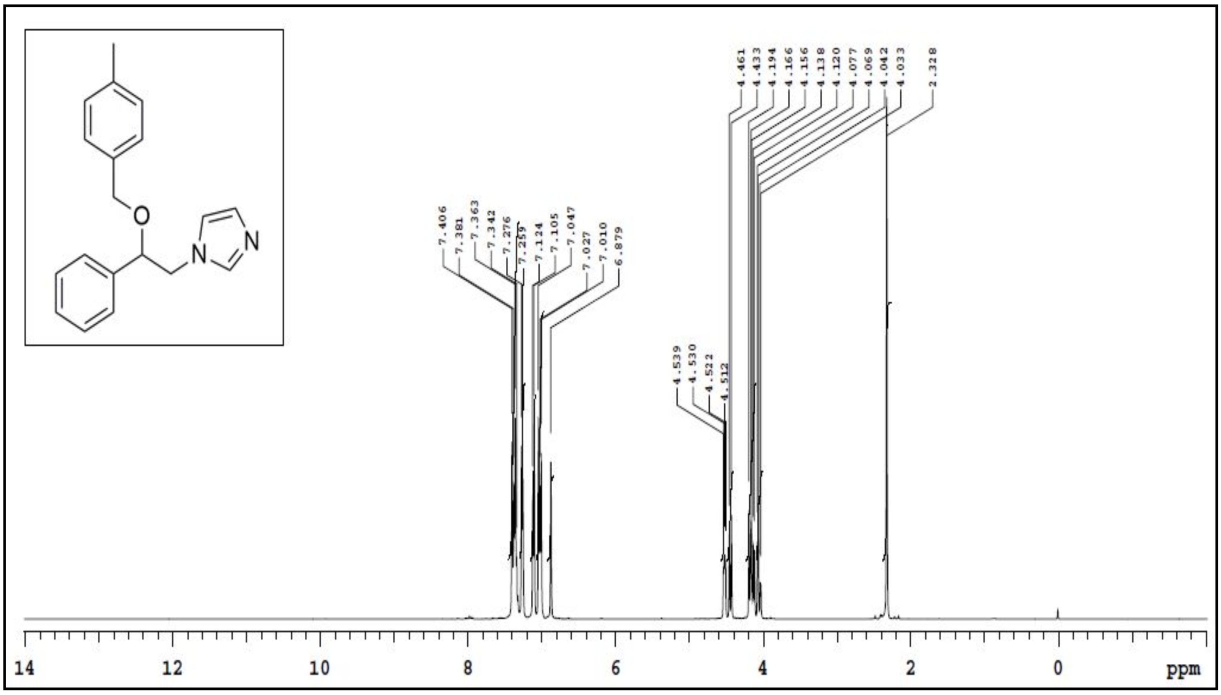

Figure S72. ^{1}H NMR Spectrum ($CDCl_3$) **(18).**

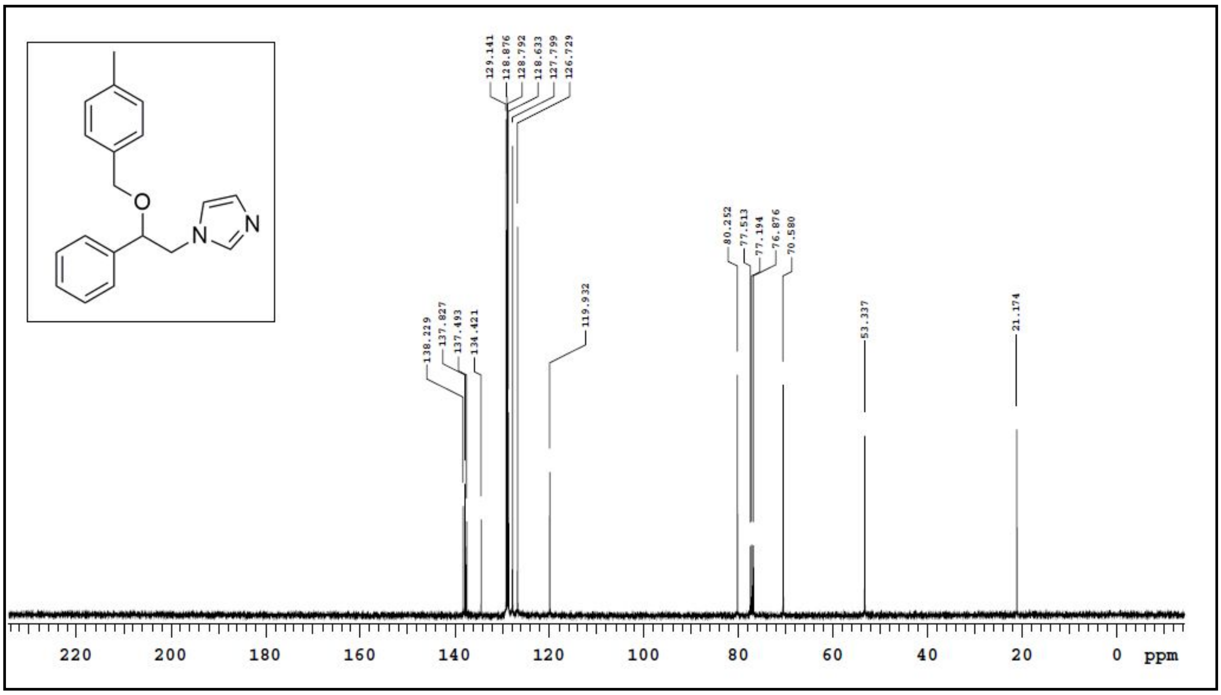

Figure S73. ^{13}C NMR Spectrum ($CDCl_3$) **(18).**

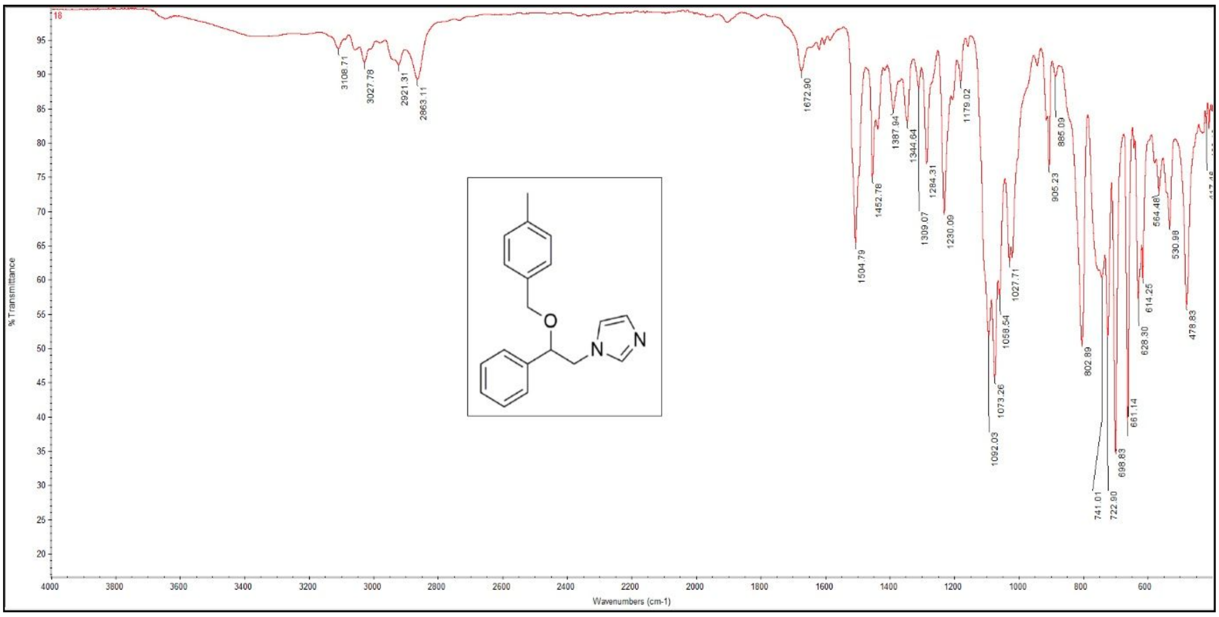

Figure S74. FT-IR Spectrum **(18).**

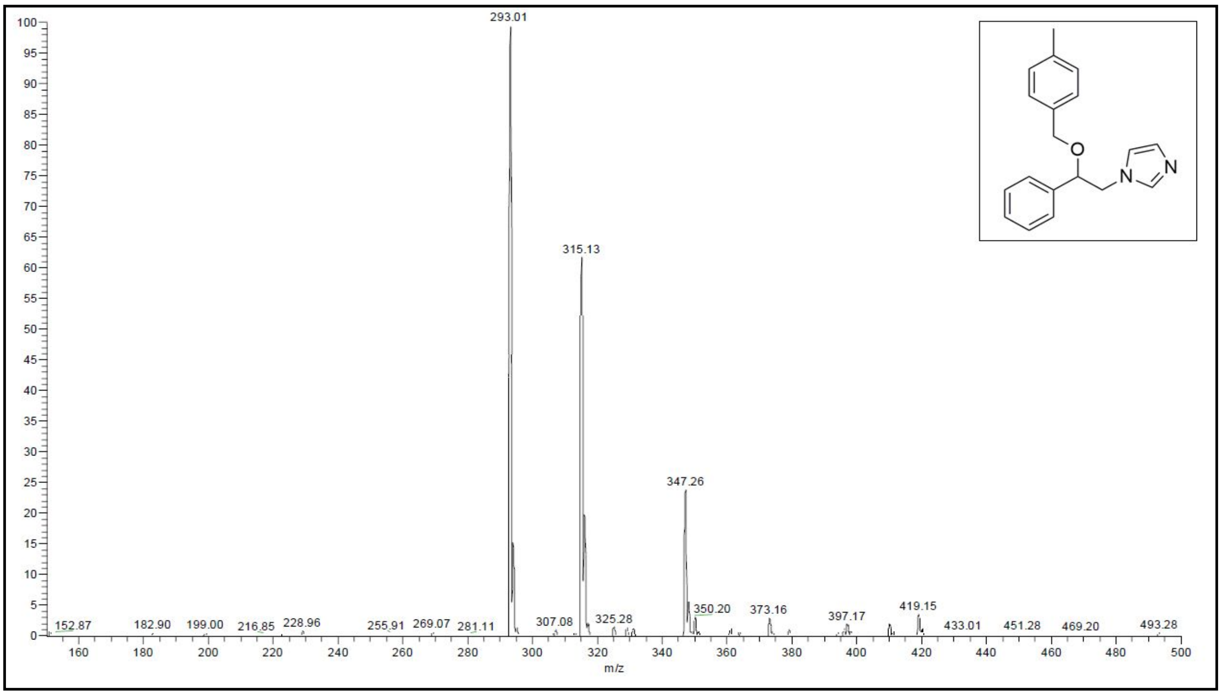

Figure S75. Mass Spectrum **(18).**

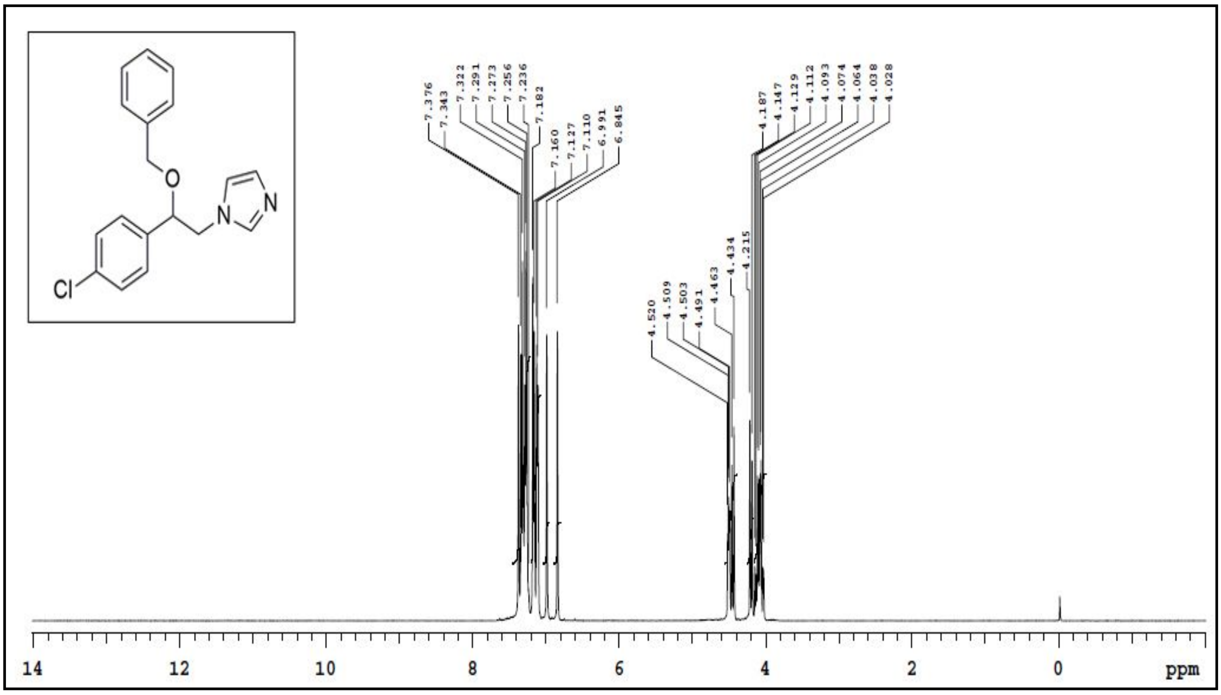

Figure S76. ^{1}H NMR Spectrum ($CDCl_3$) **(19).**

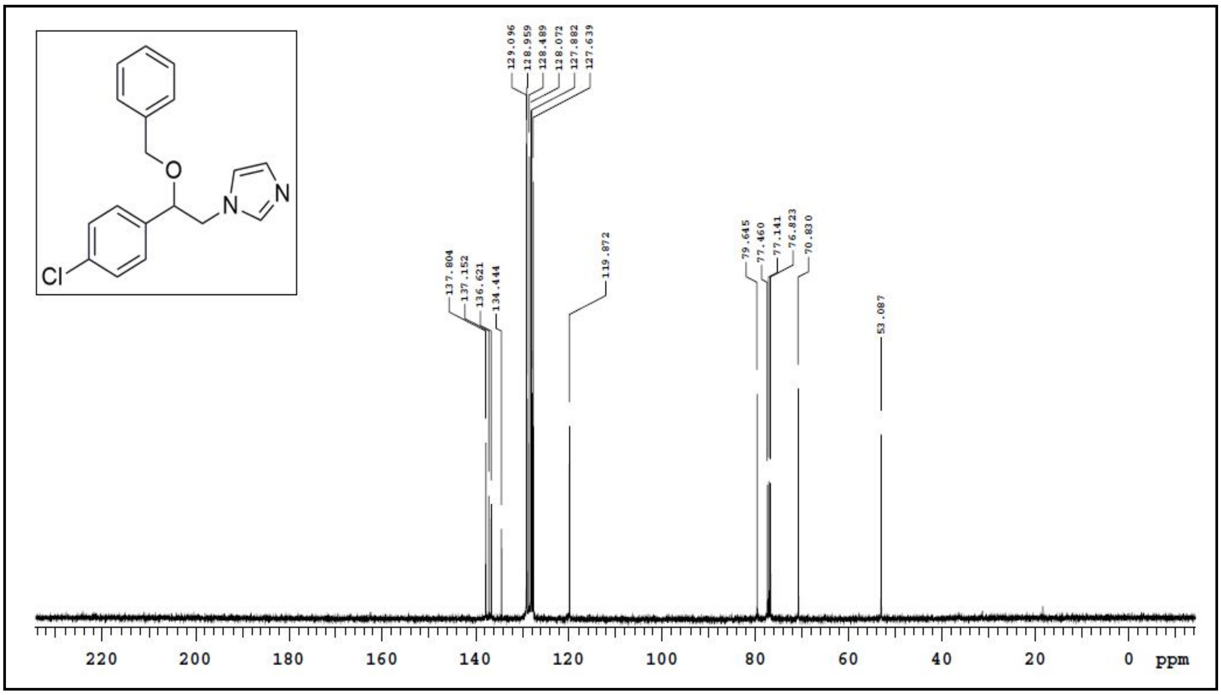

Figure S77. ^{13}C NMR Spectrum ($CDCl_3$) **(19).**

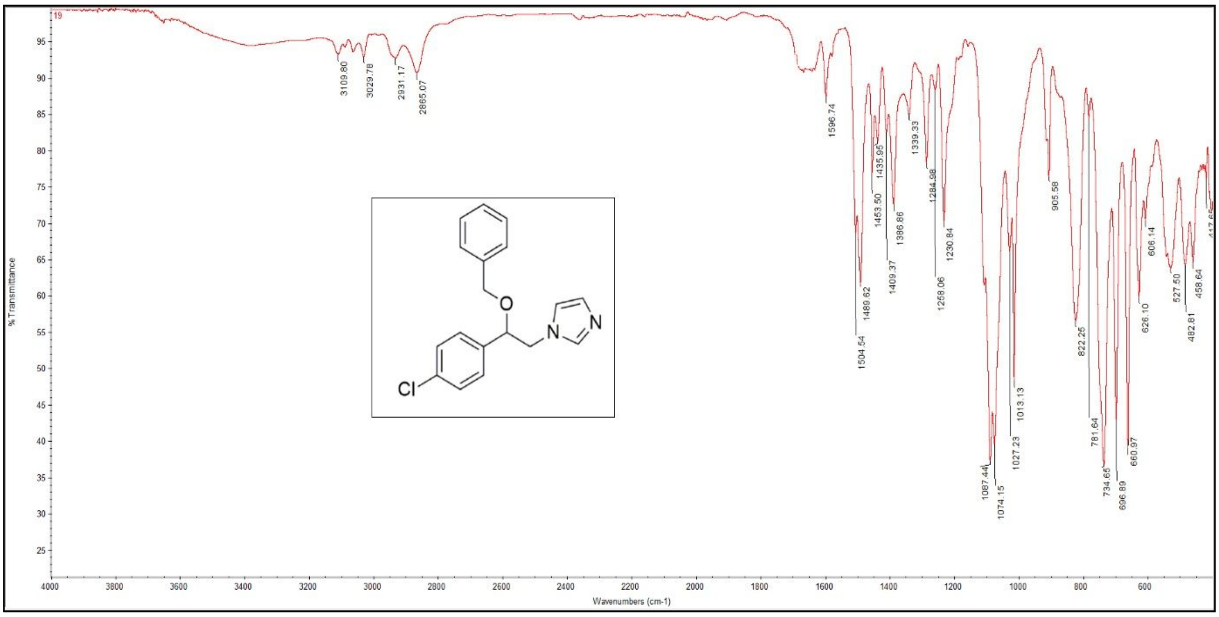

Figure S78. FT-IR Spectrum **(19).**

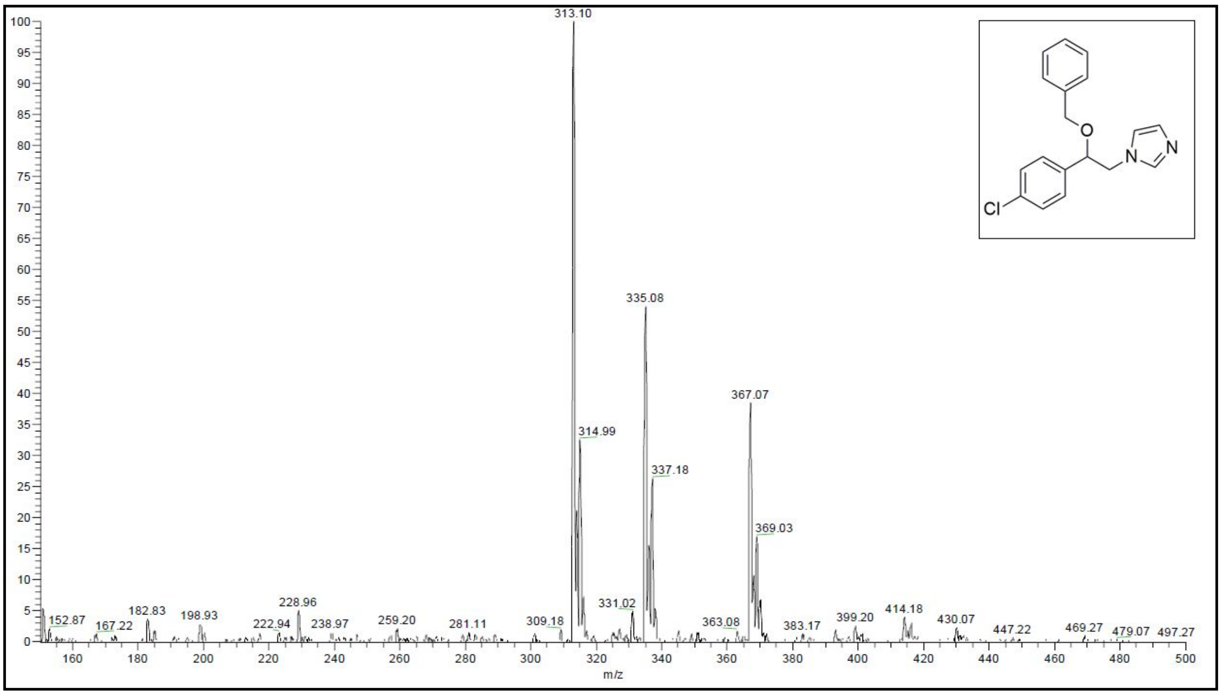

Figure S79. Mass Spectrum **(19).**

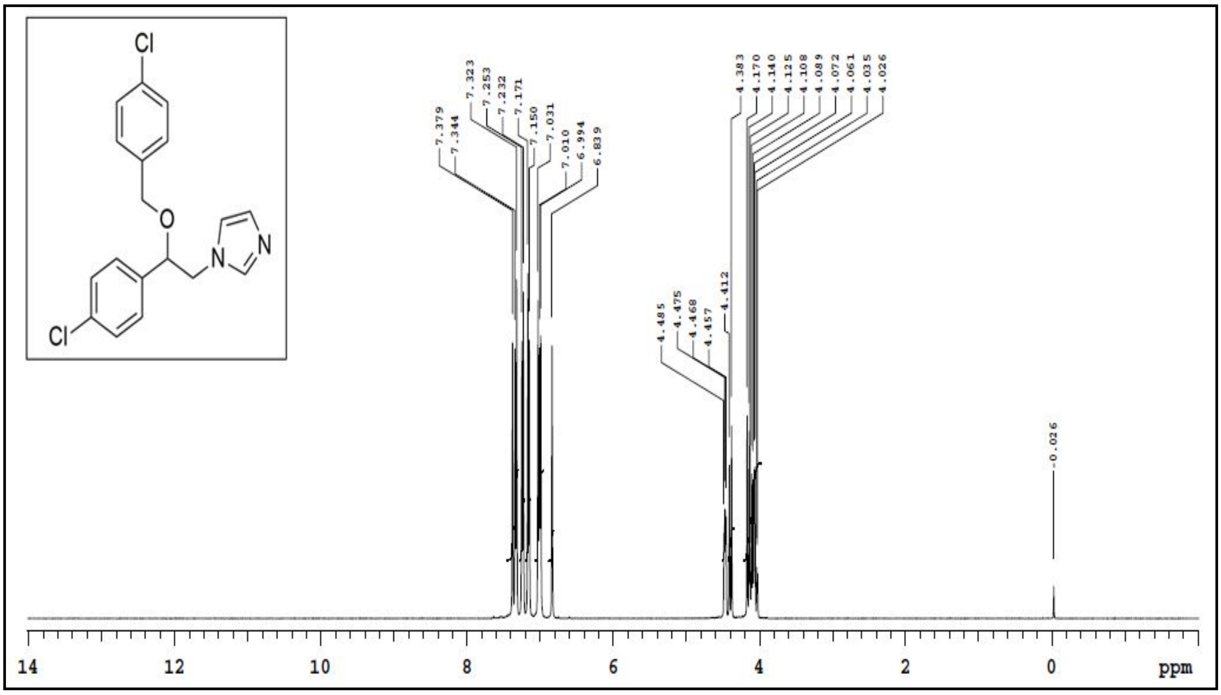

Figure S80. ^{1}H NMR Spectrum ($CDCl_3$) **(20).**

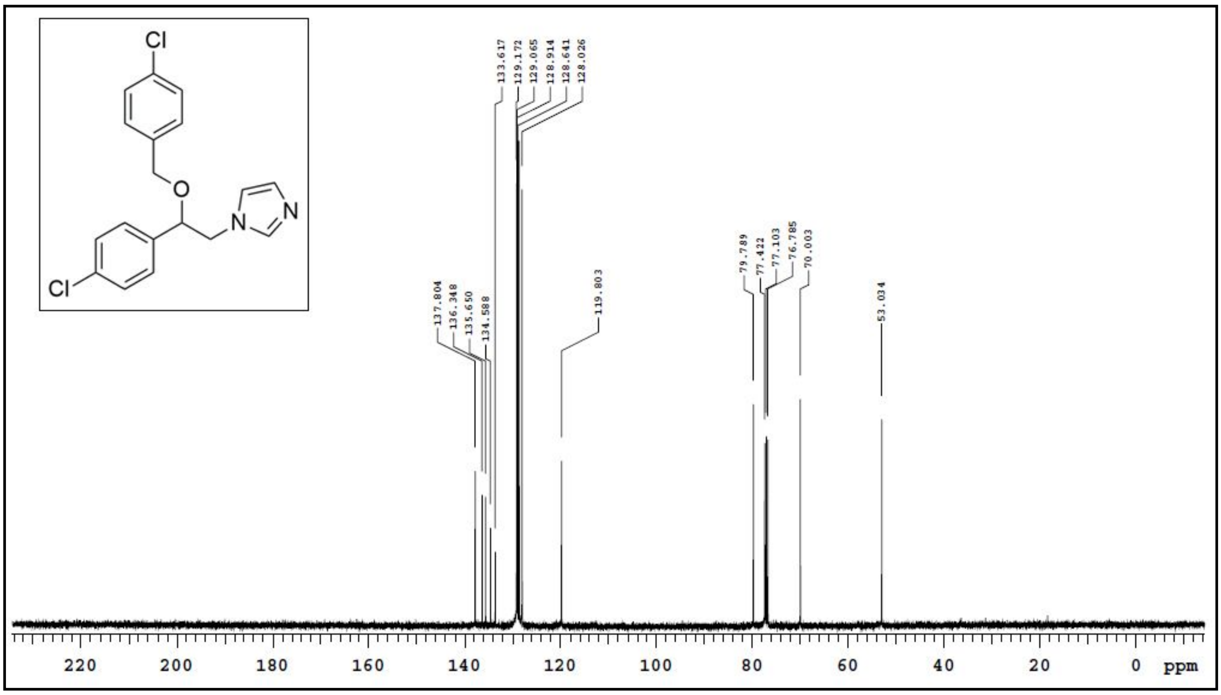

Figure S81. ^{13}C NMR Spectrum ($CDCl_3$) **(20).**

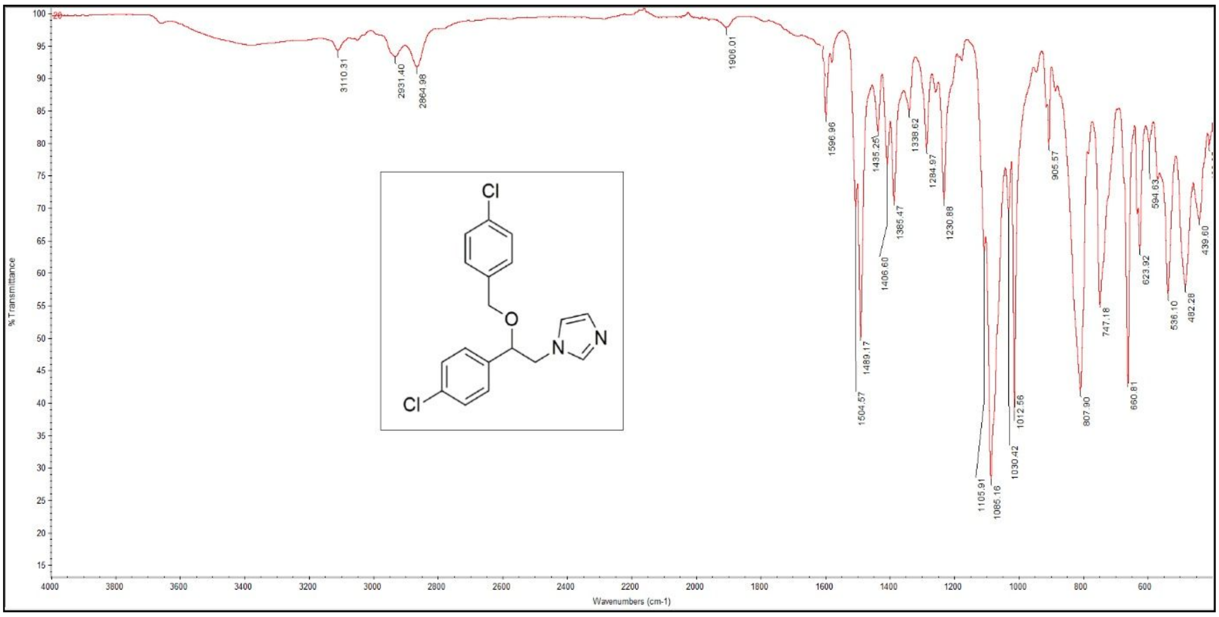

Figure S82. FT-IR Spectrum **(20).**

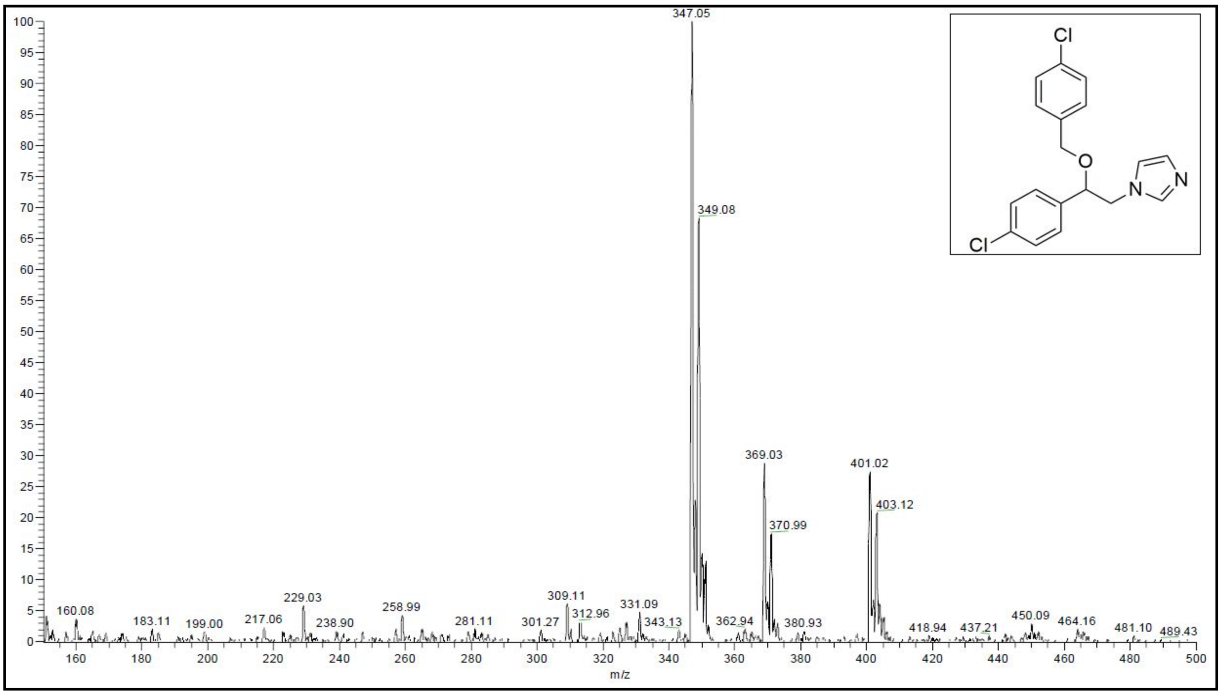

Figure S83. Mass Spectrum **(20).**

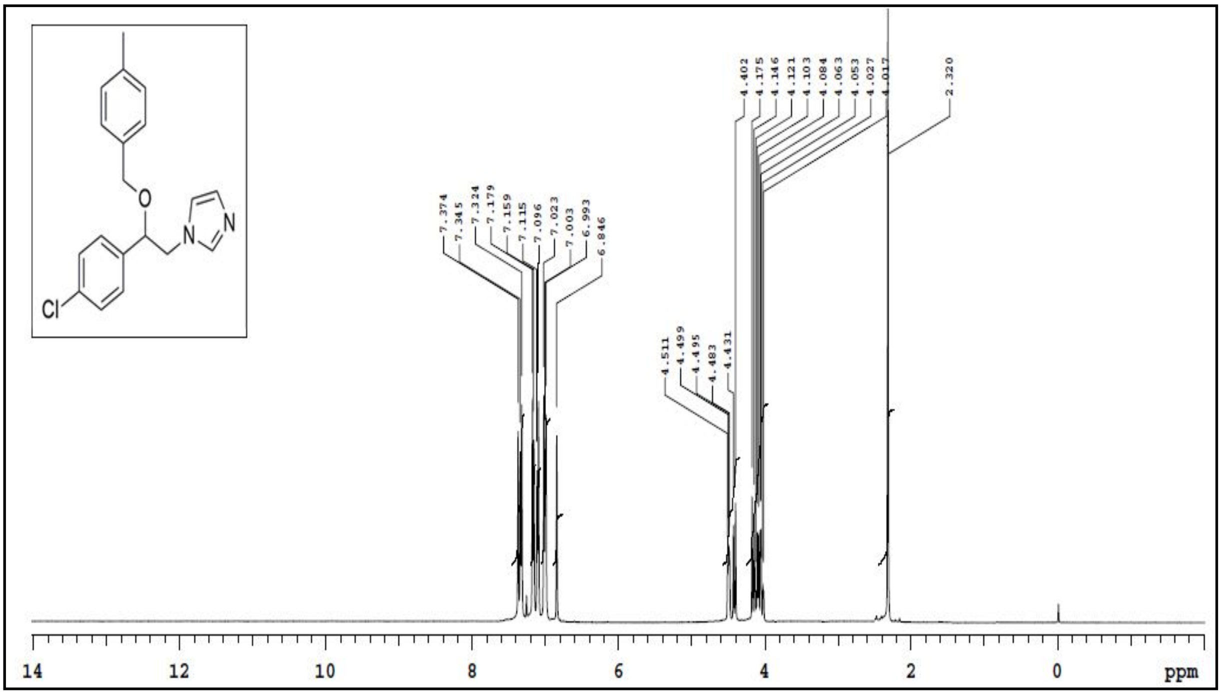

Figure S84. ¹H NMR Spectrum ($CDCl_3$) **(21).**

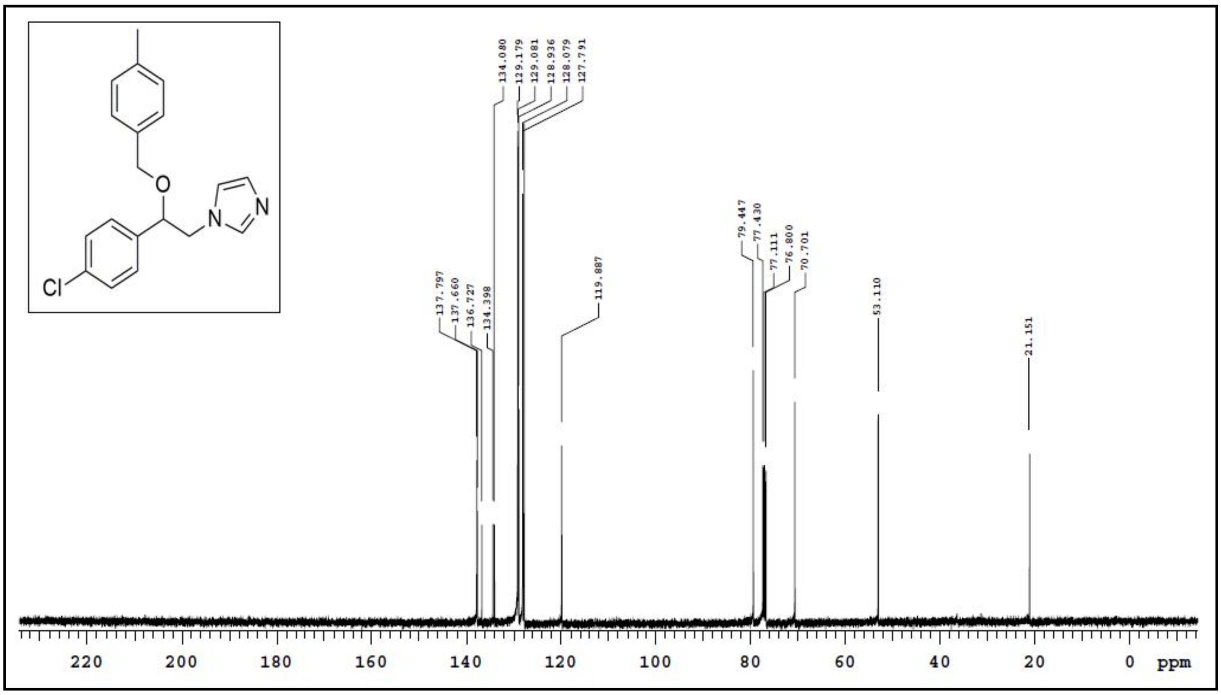

Figure S85. ^{13}C NMR Spectrum ($CDCl_3$) **(21).**

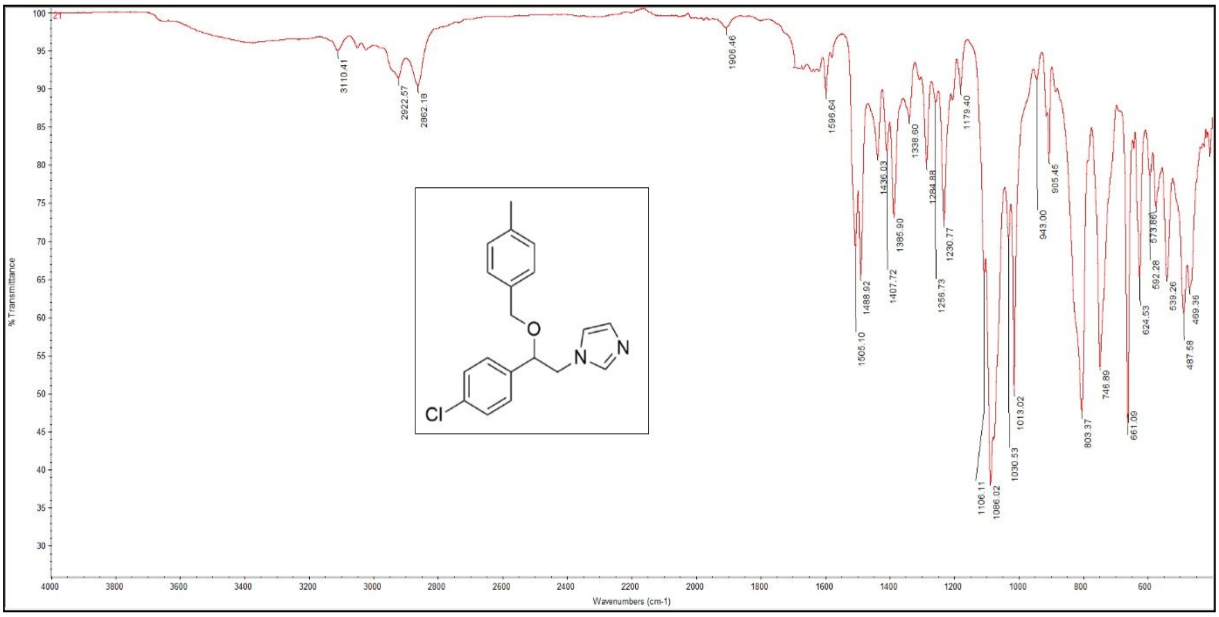

Figure S86. FT-IR Spectrum **(21).**

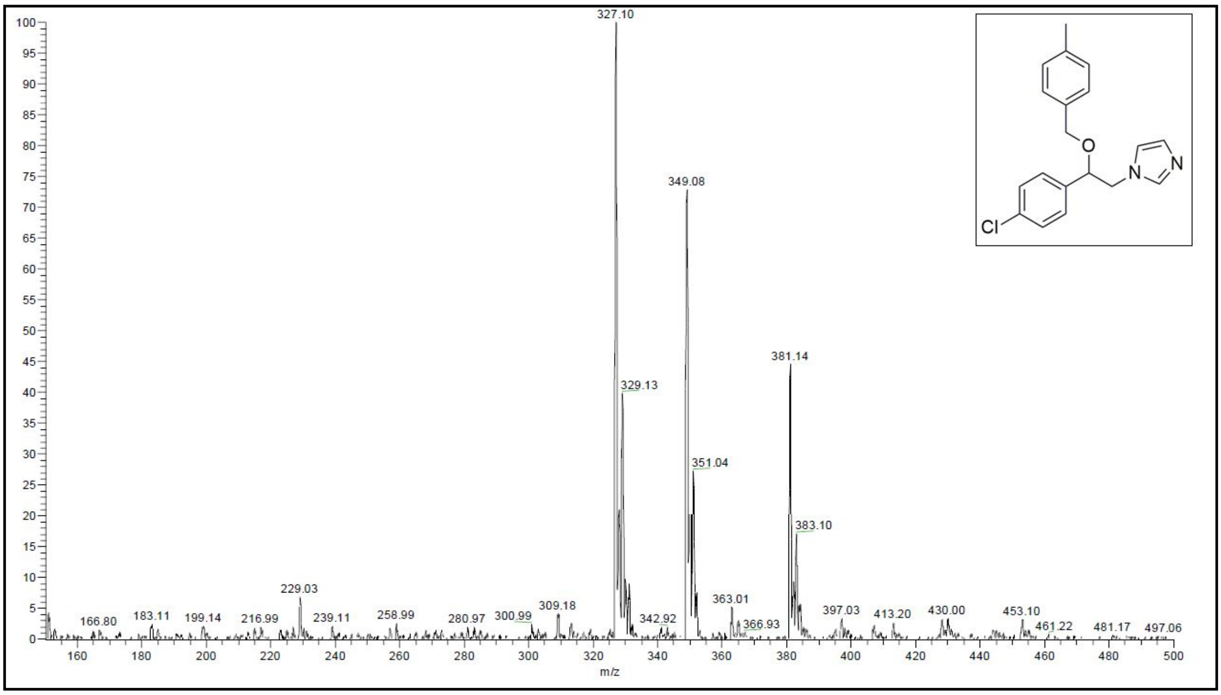

Figure S87. Mass Spectrum **(21).**

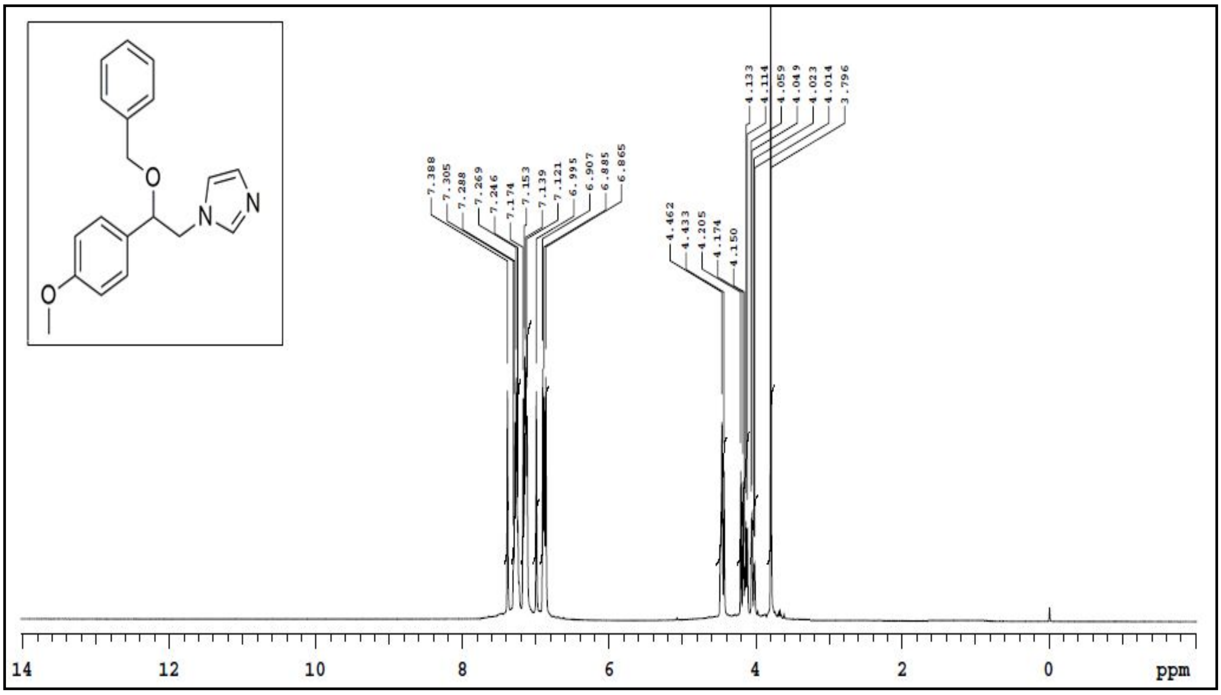

Figure S88. ^{1}H NMR Spectrum ($CDCl_3$) **(22).**

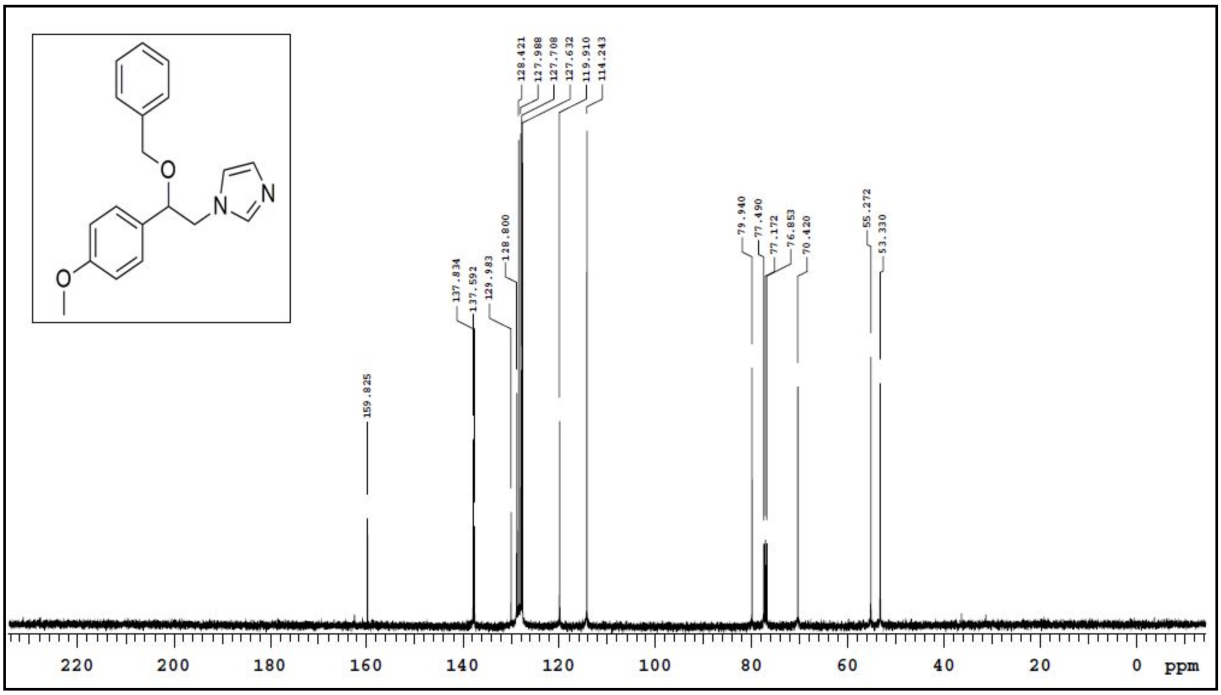

Figure S89. ^{13}C NMR Spectrum ($CDCl_3$) **(22).**

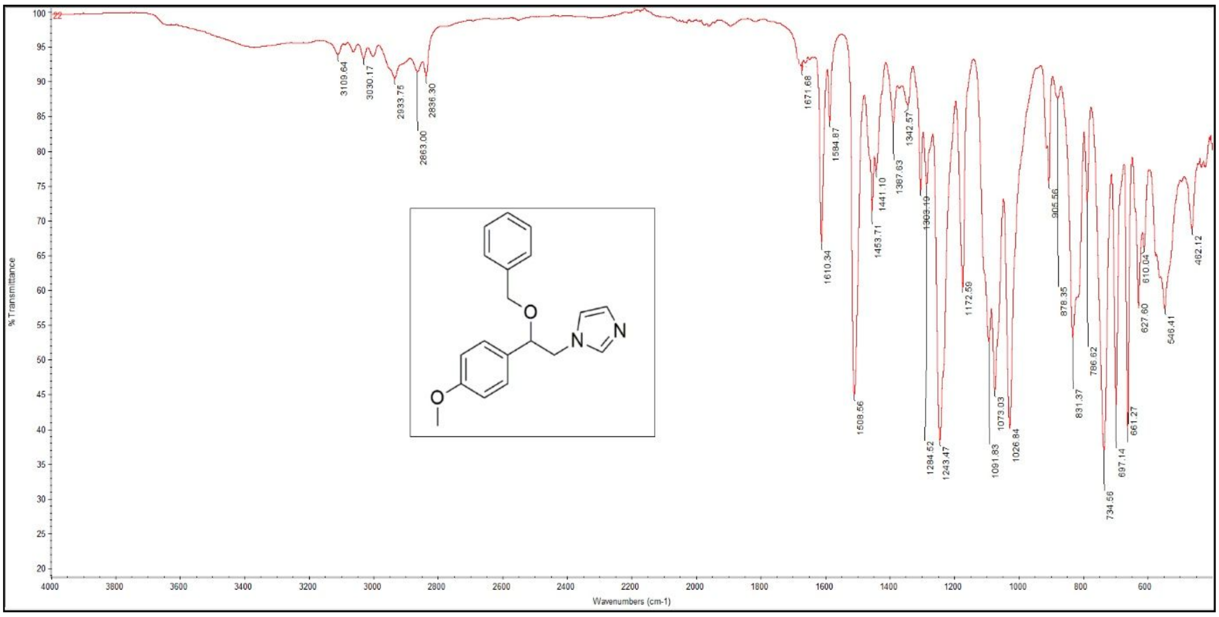

Figure S90. FT-IR Spectrum **(22).**

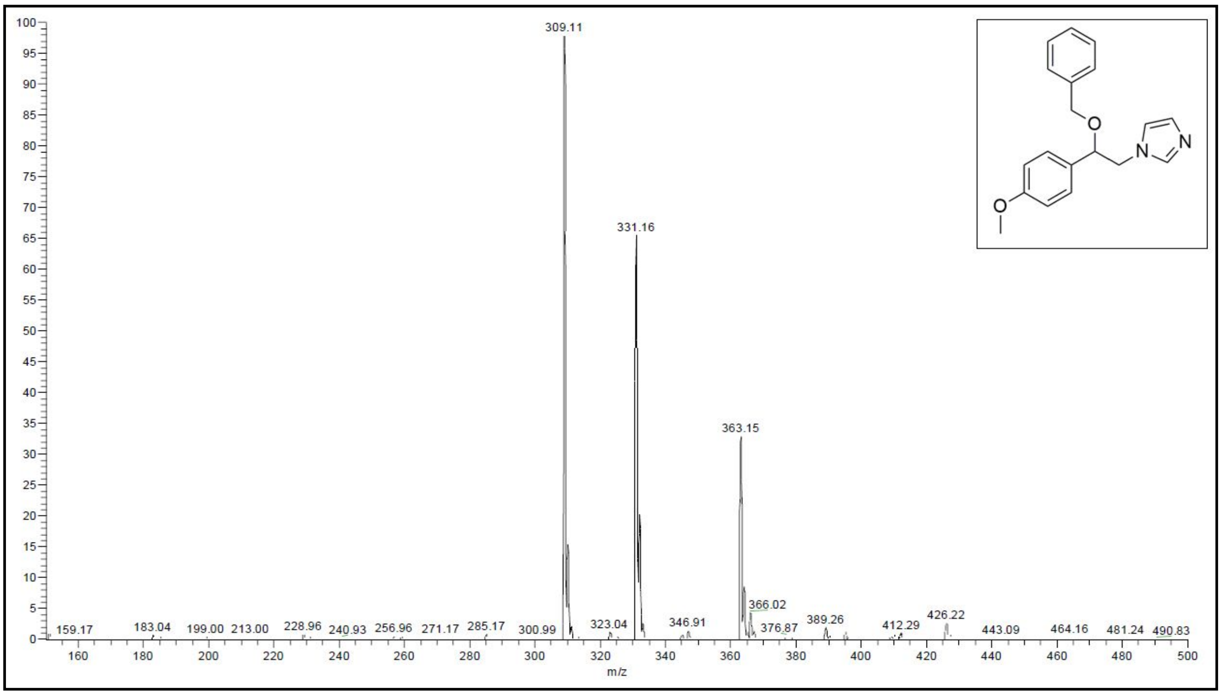

Figure S91. Mass Spectrum **(22).**

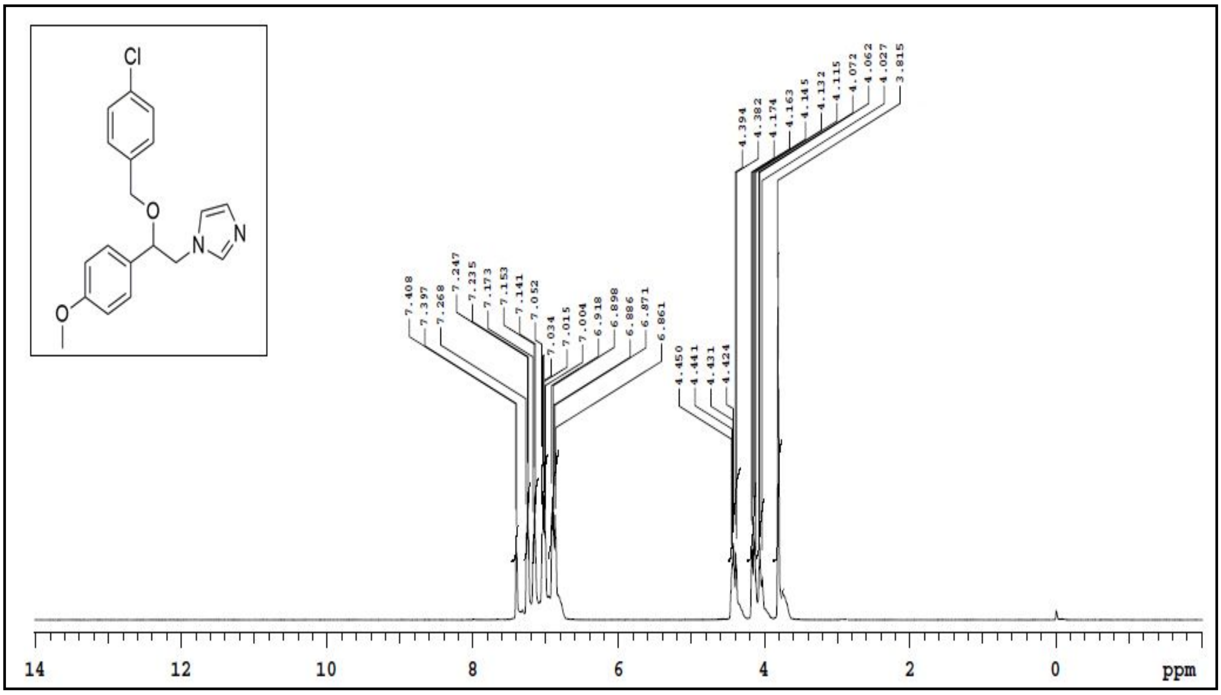

Figure S92. ^{1}H NMR Spectrum ($CDCl_3$) **(23).**

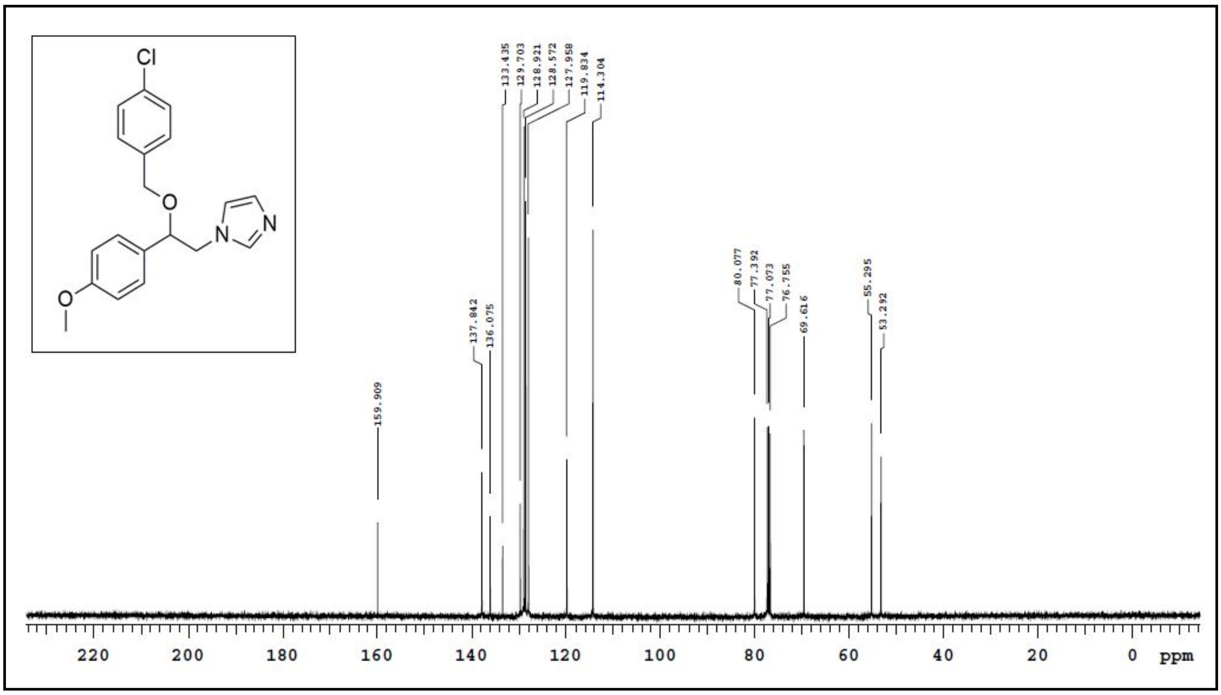

Figure S93. ^{13}C NMR Spectrum ($CDCl_3$) **(23).**

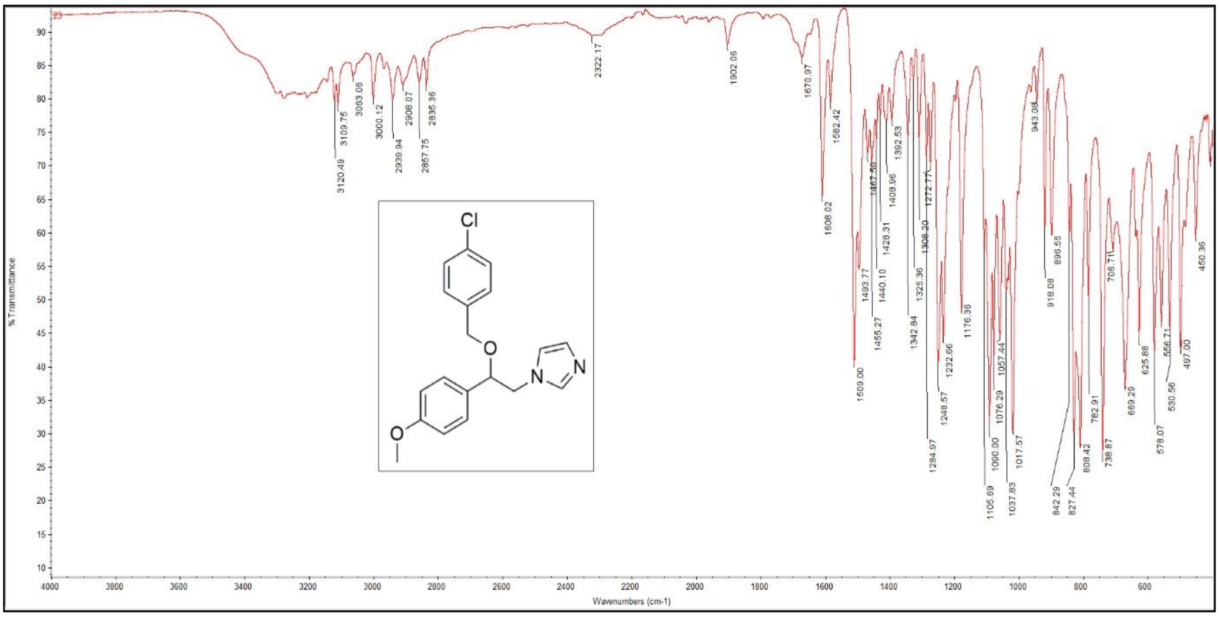

Figure S94. FT-IR Spectrum **(23).**

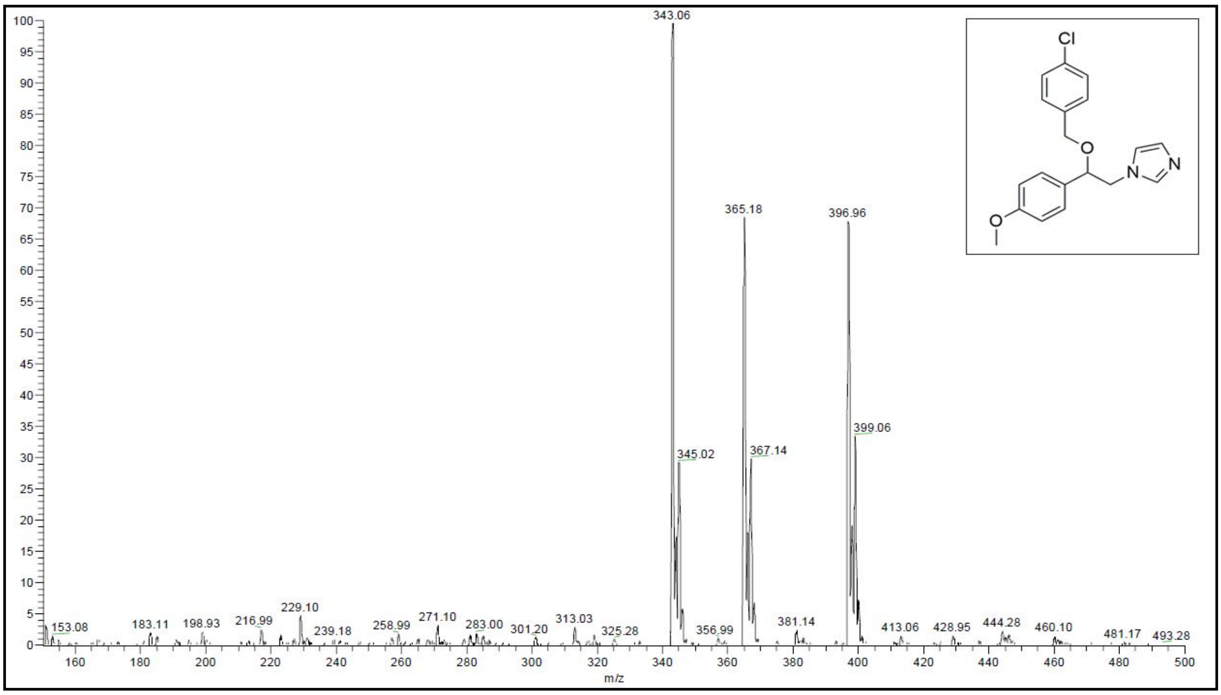

Figure S95. Mass Spectrum **(23).**

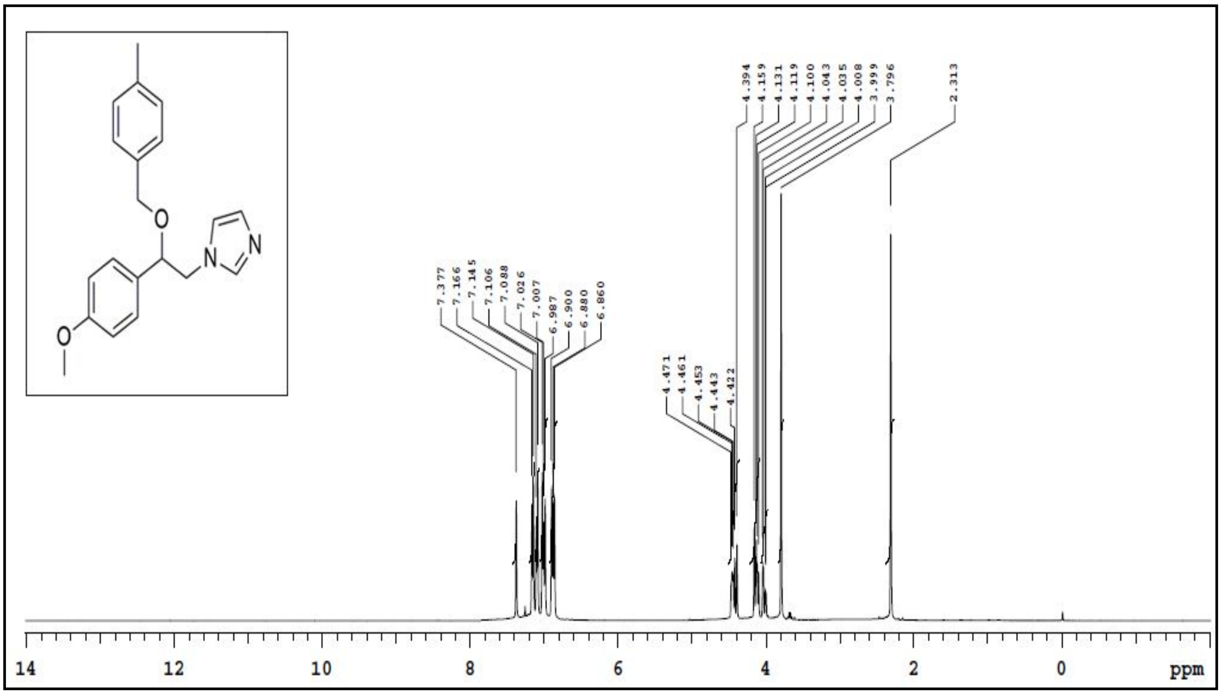

Figure S96. ^{1}H NMR Spectrum ($CDCl_3$) **(24).**

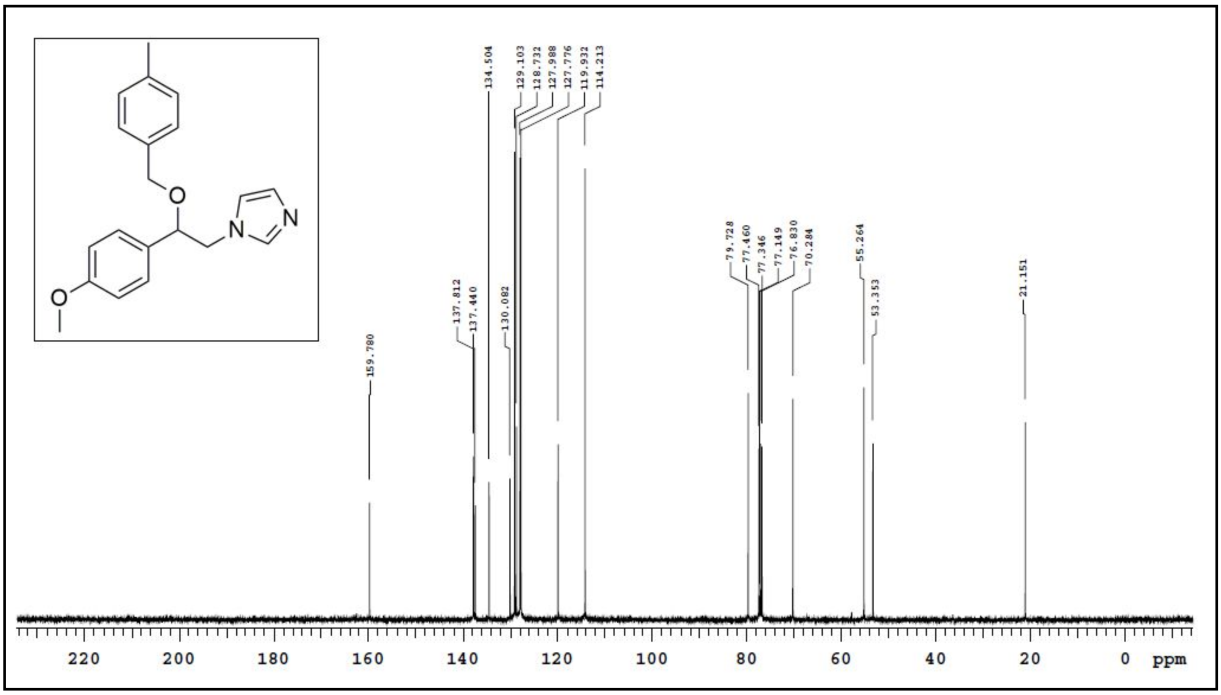

Figure S97. ^{13}C NMR Spectrum ($CDCl_3$) **(24).**

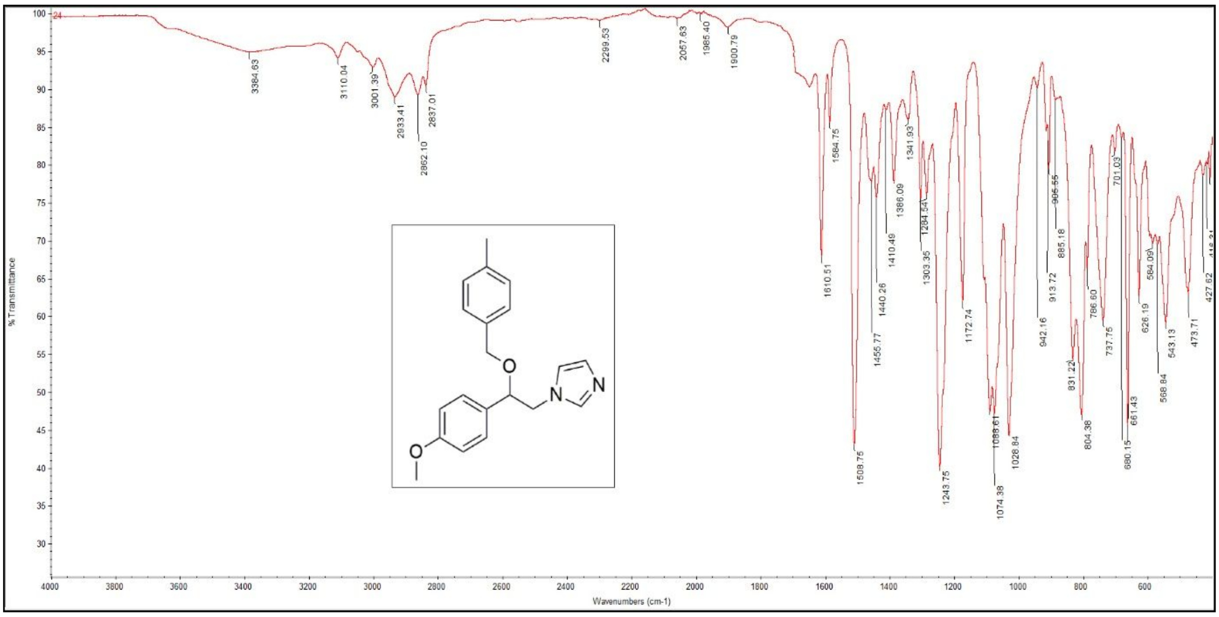

Figure S98. FT-IR Spectrum **(24).**

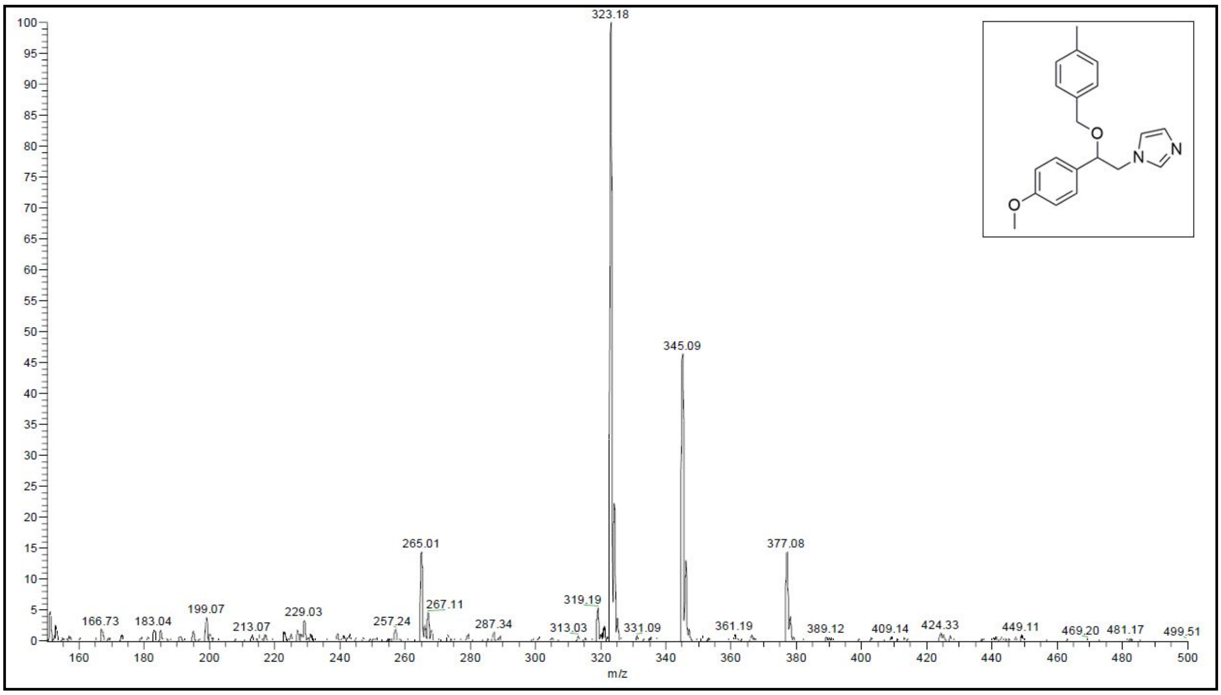

Figure S99. Mass Spectrum **(24).**

2. Geometry-Optimized Structures of the Novel and Known Compounds

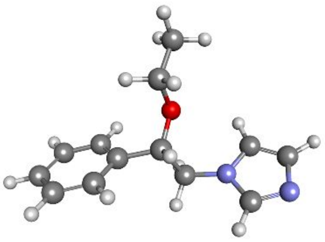
7

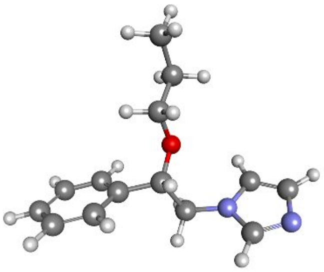
8

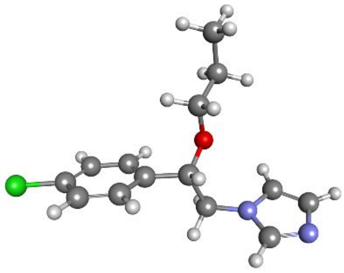
11

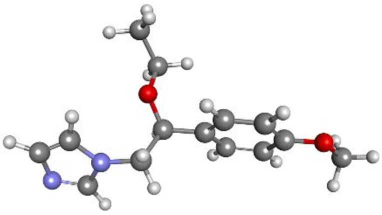
13

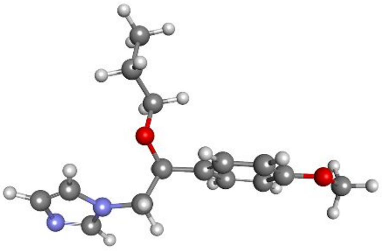
14

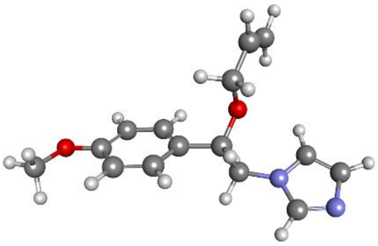
15

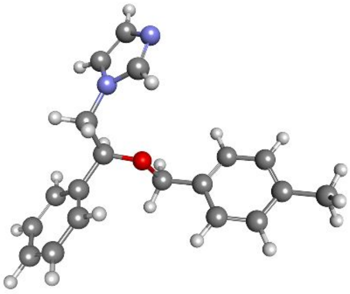
18

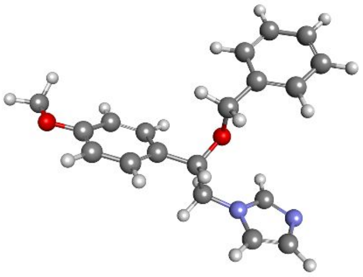
22

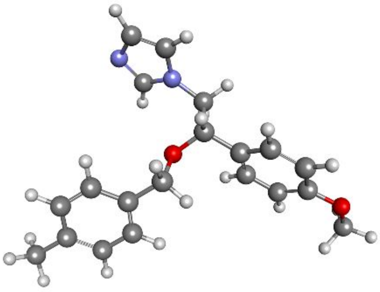
24

Figure S100. Geometry-optimized structures of novel compounds.

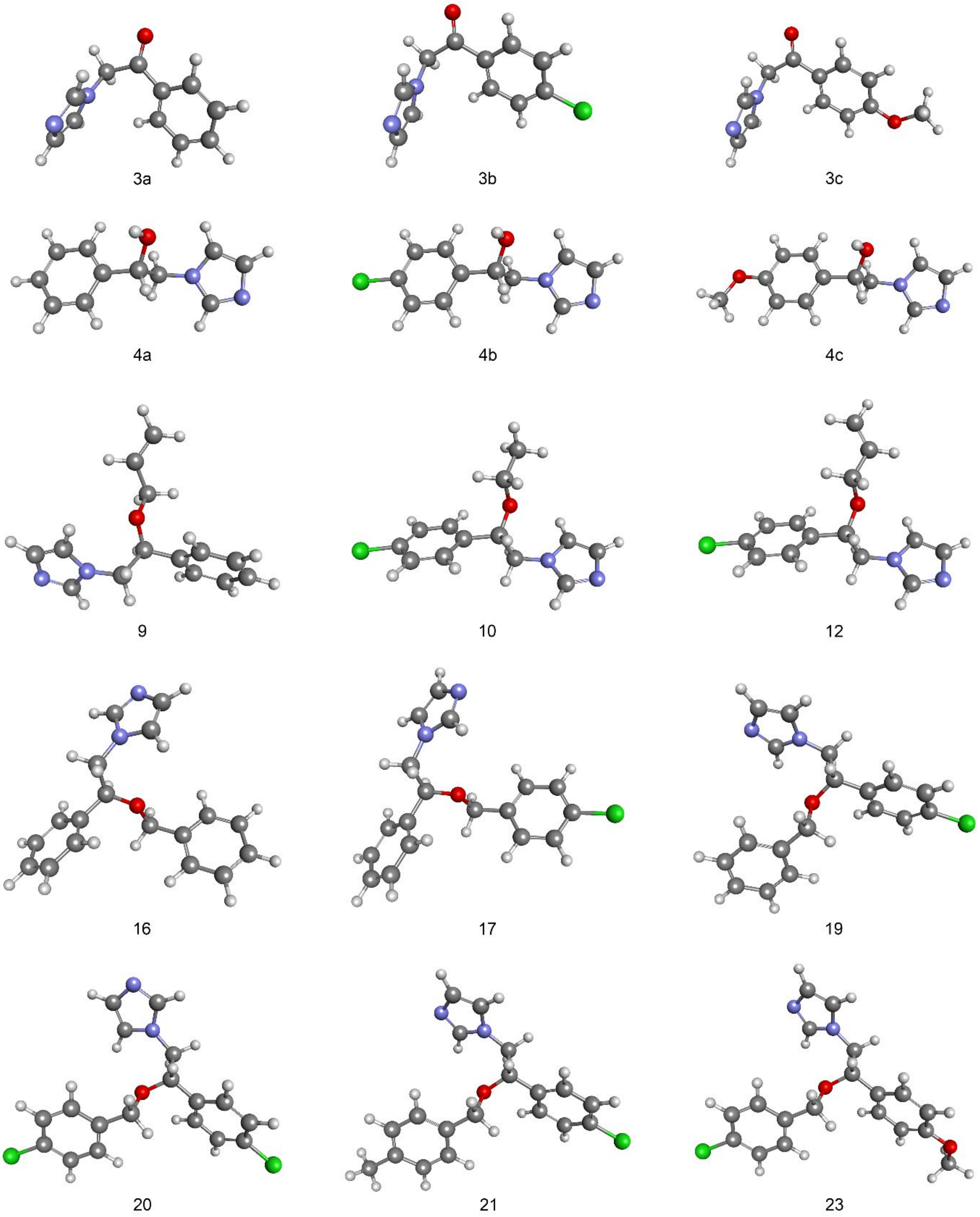

Figure S101. Geometry-optimized structures of the known compounds.

3. Molecular Electrostatic Potential Maps of the Novel and Known Compounds

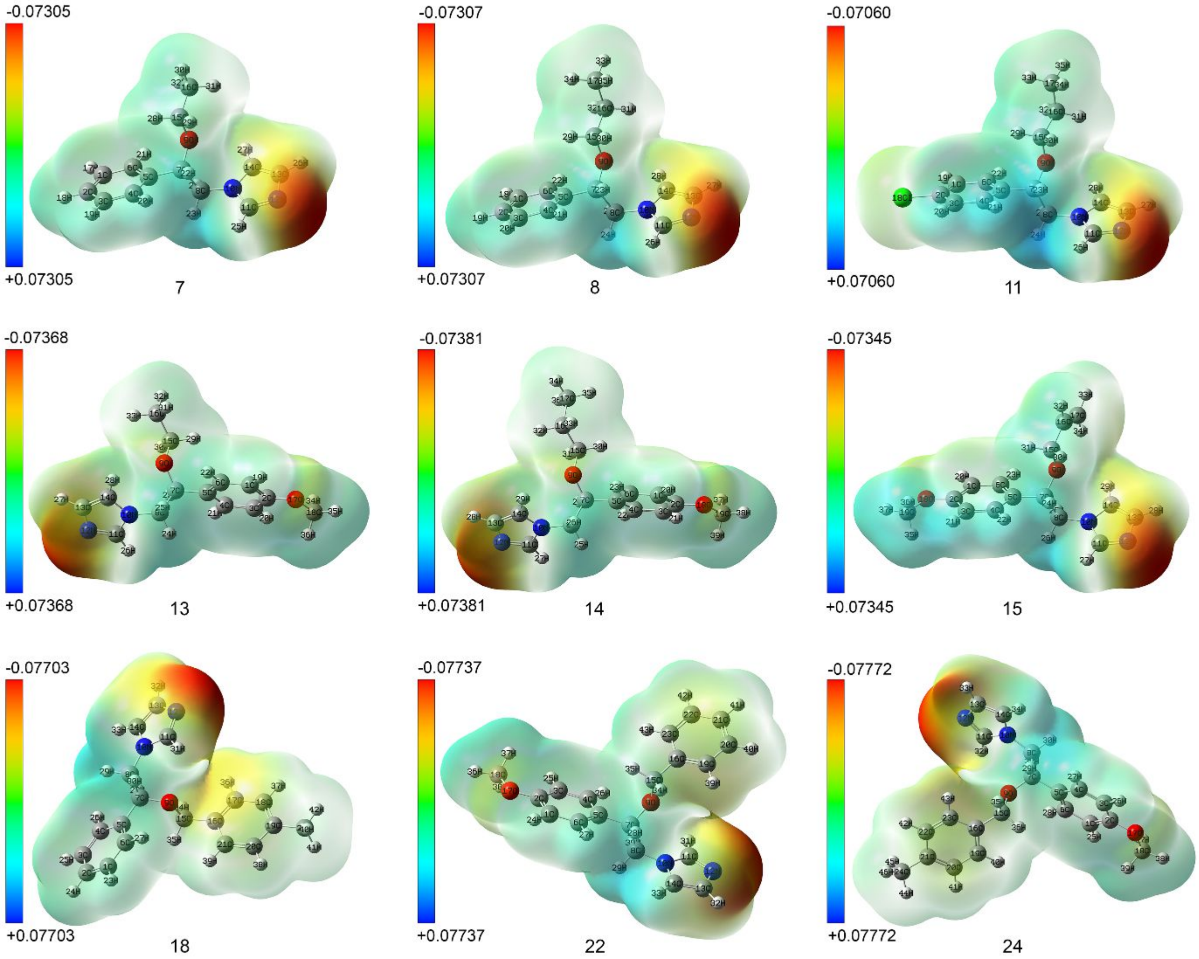

Figure S102. Molecular electrostatic potential maps of the novel compounds.

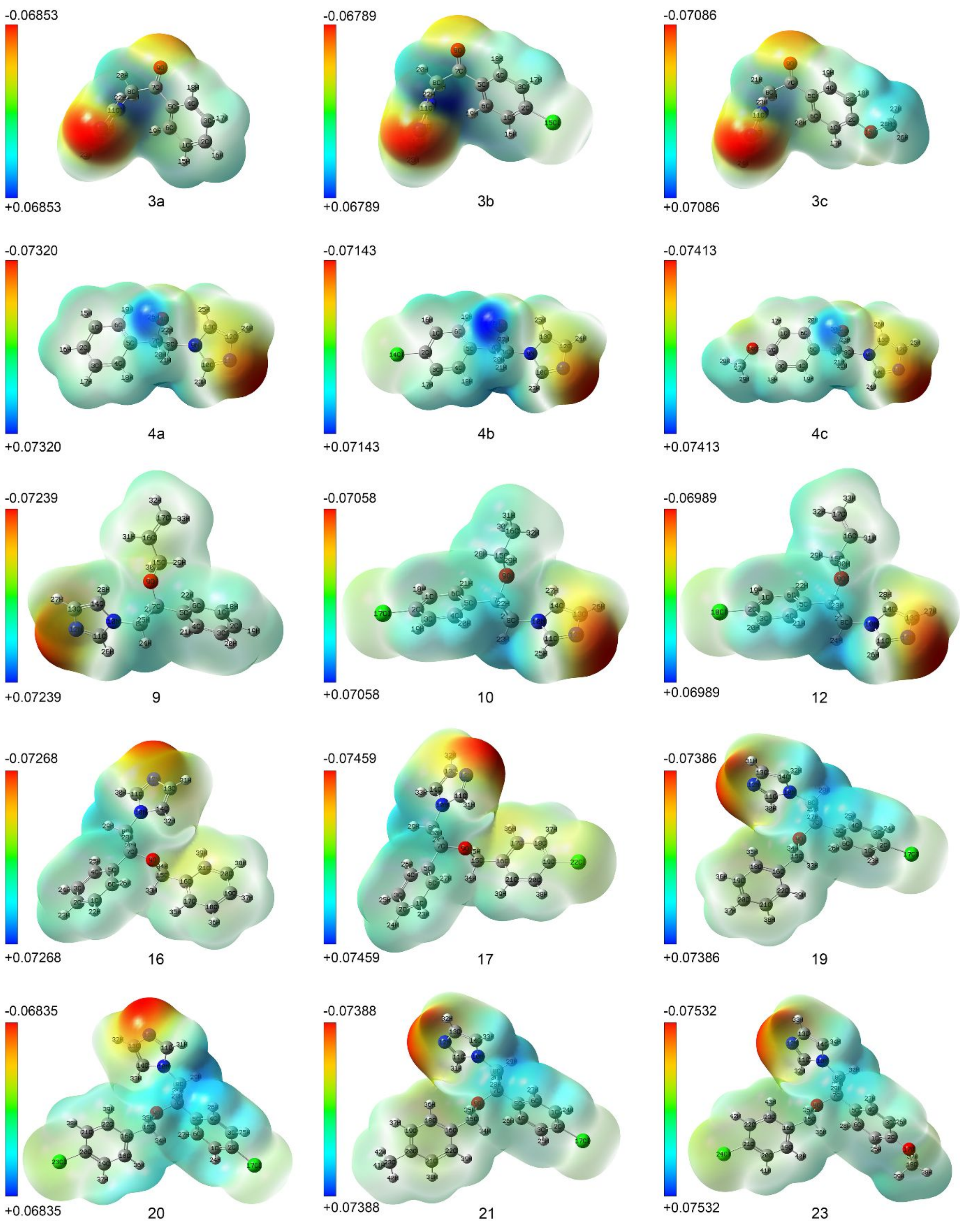

Figure S103. Molecular electrostatic potential maps of the known compounds.

4. Binding Poses and Ligand-Receptor Interactions

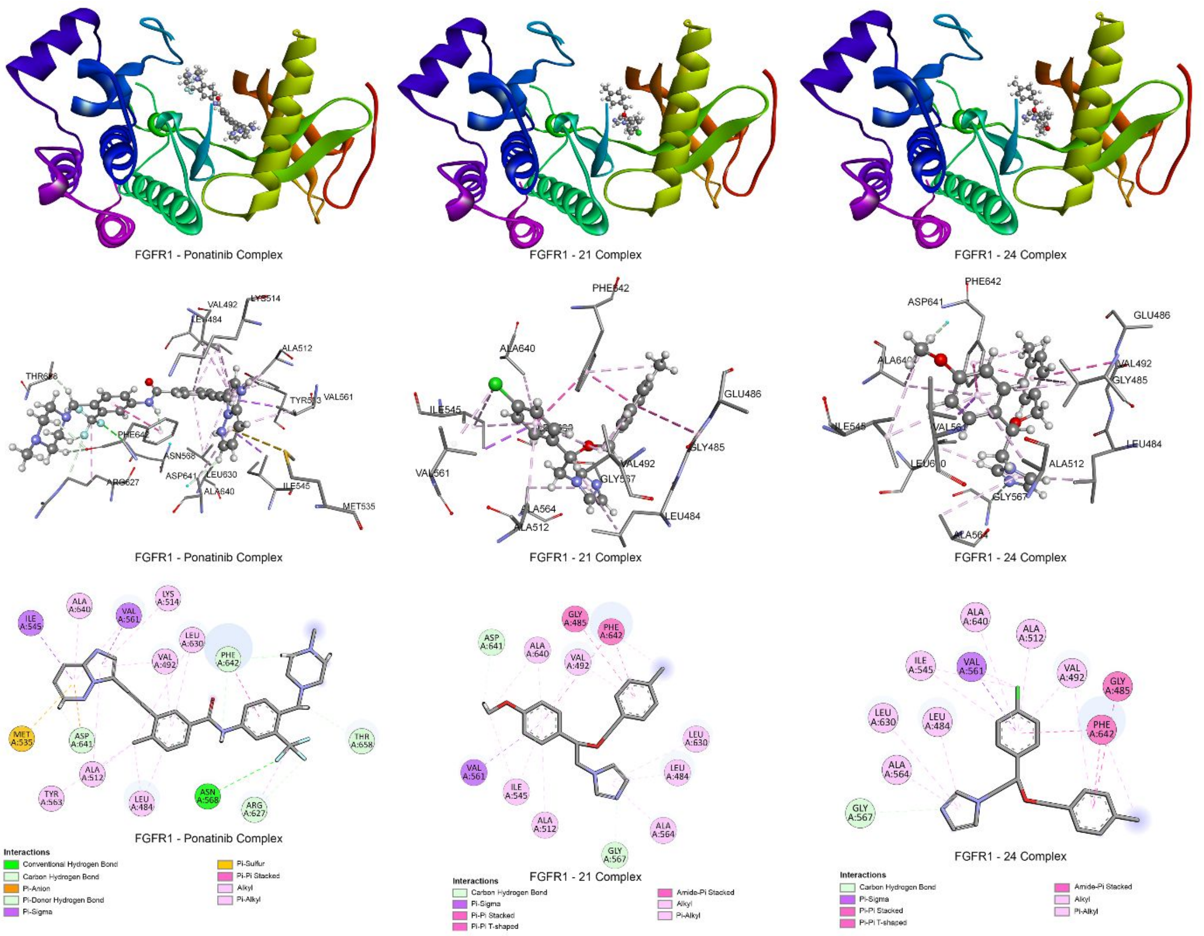

Figure S104. Binding poses and ligand-receptor interactions for FGFR1 – Ponatinib, FGFR1 – 21, and FGFR1 – 24 complexes.

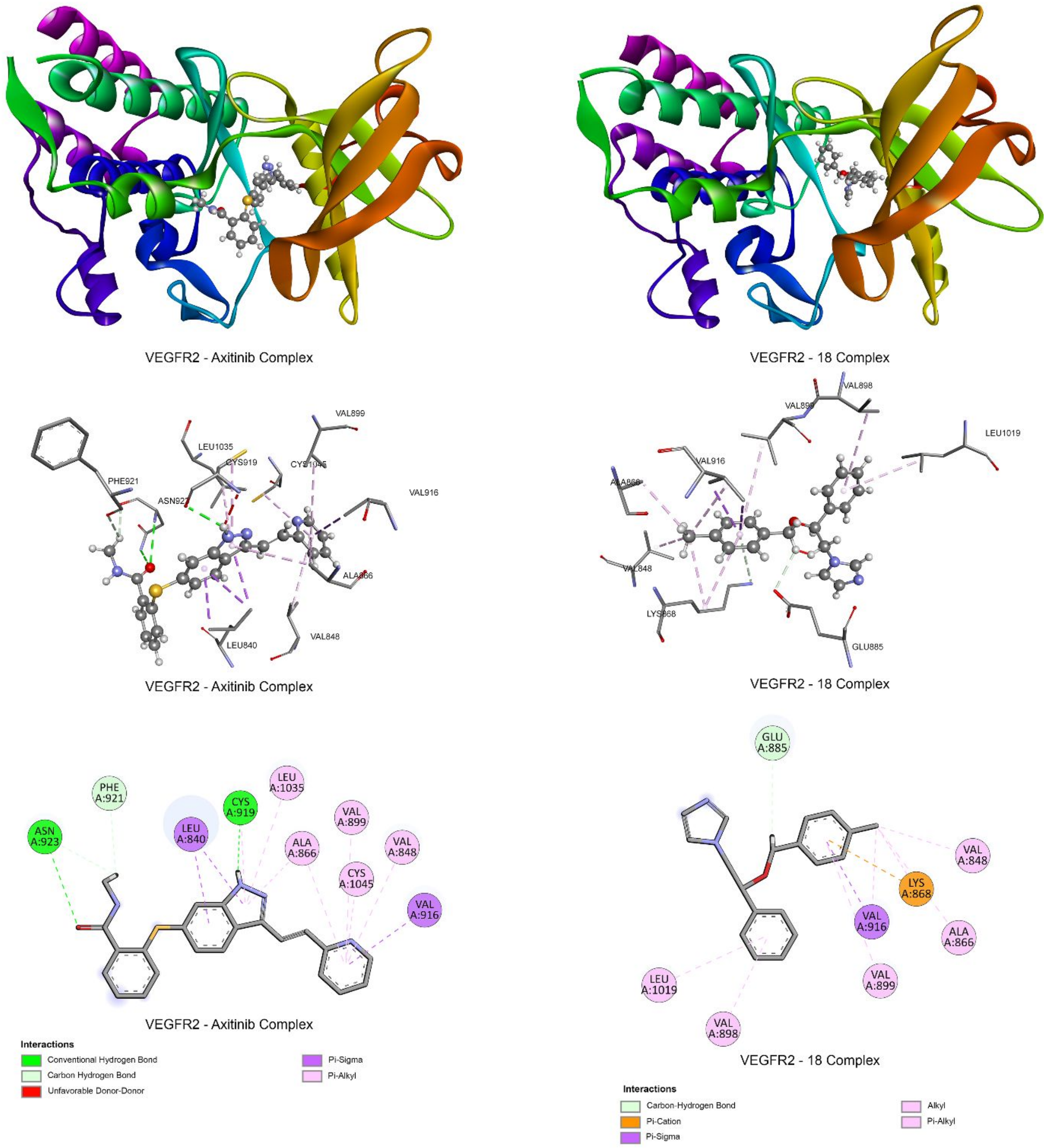

Figure S105. Binding poses and ligand-receptor interactions for VEGFR2 – Axitinib and VEGFR2 – 18 complexes.

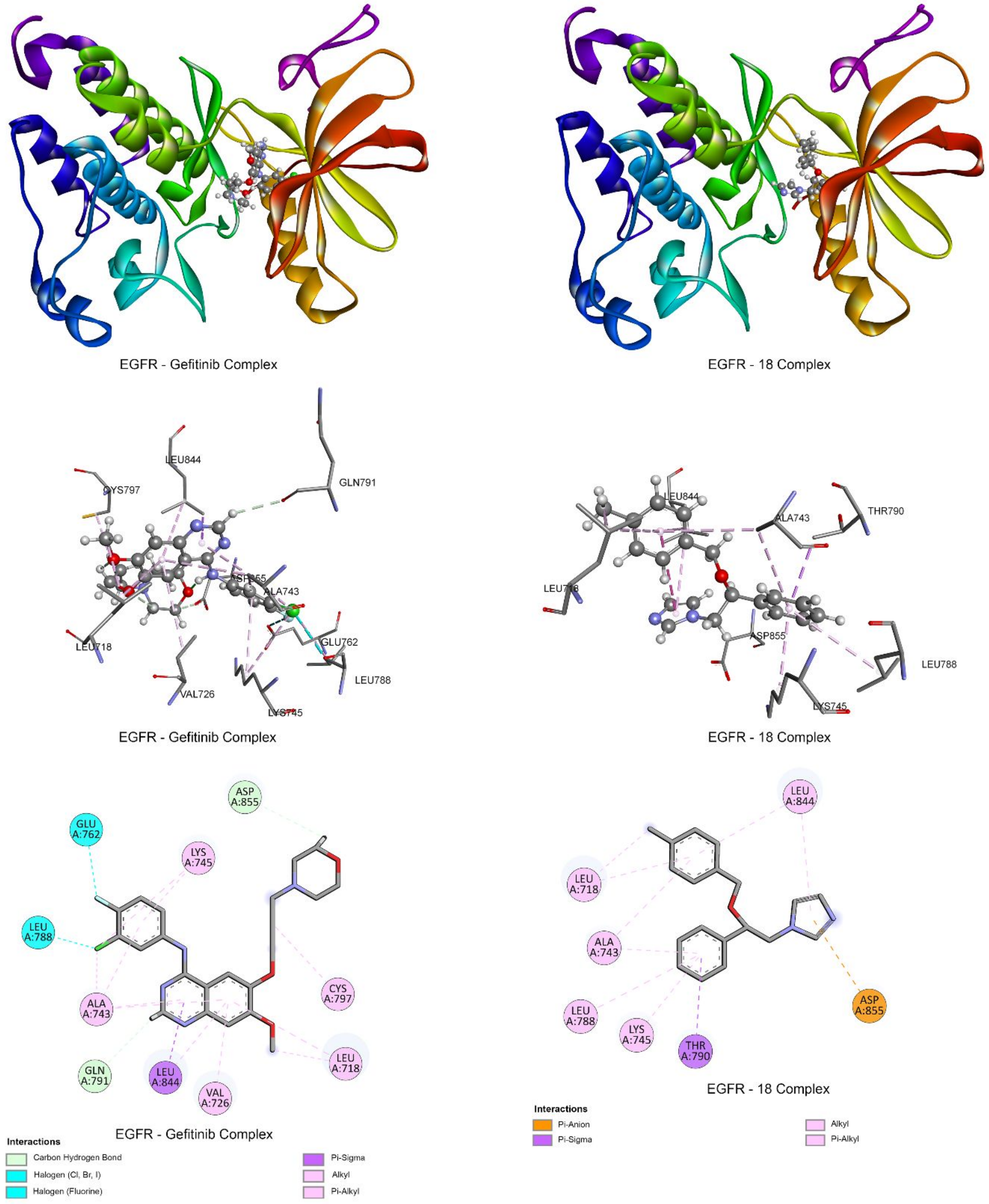

Figure S106. Binding poses and ligand-receptor interactions for EGFR – Gefitinib and EGFR – 18 complexes.

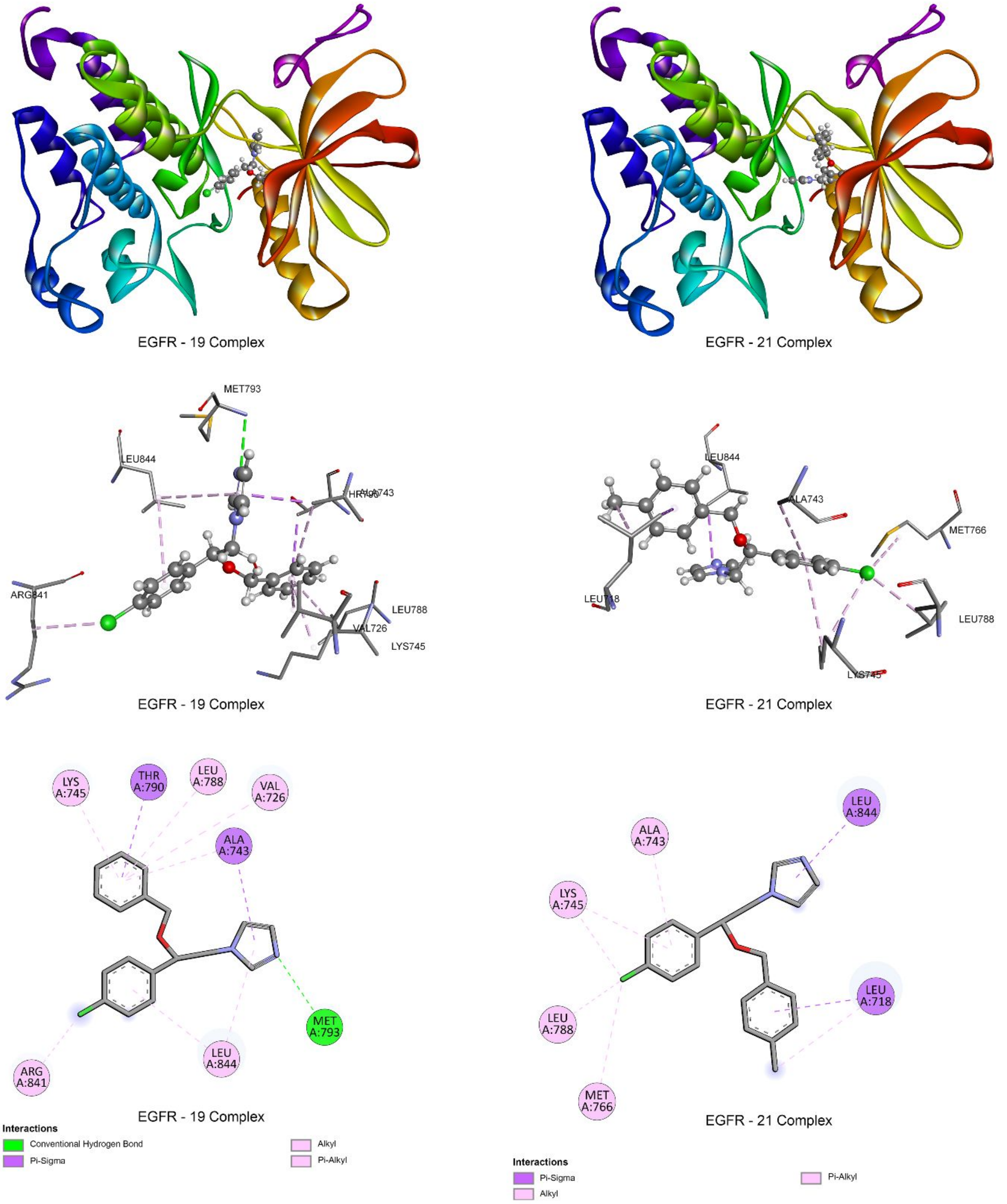

Figure S107. Binding poses and ligand-receptor interactions for EGFR – 19 and EGFR – 21 complexes.

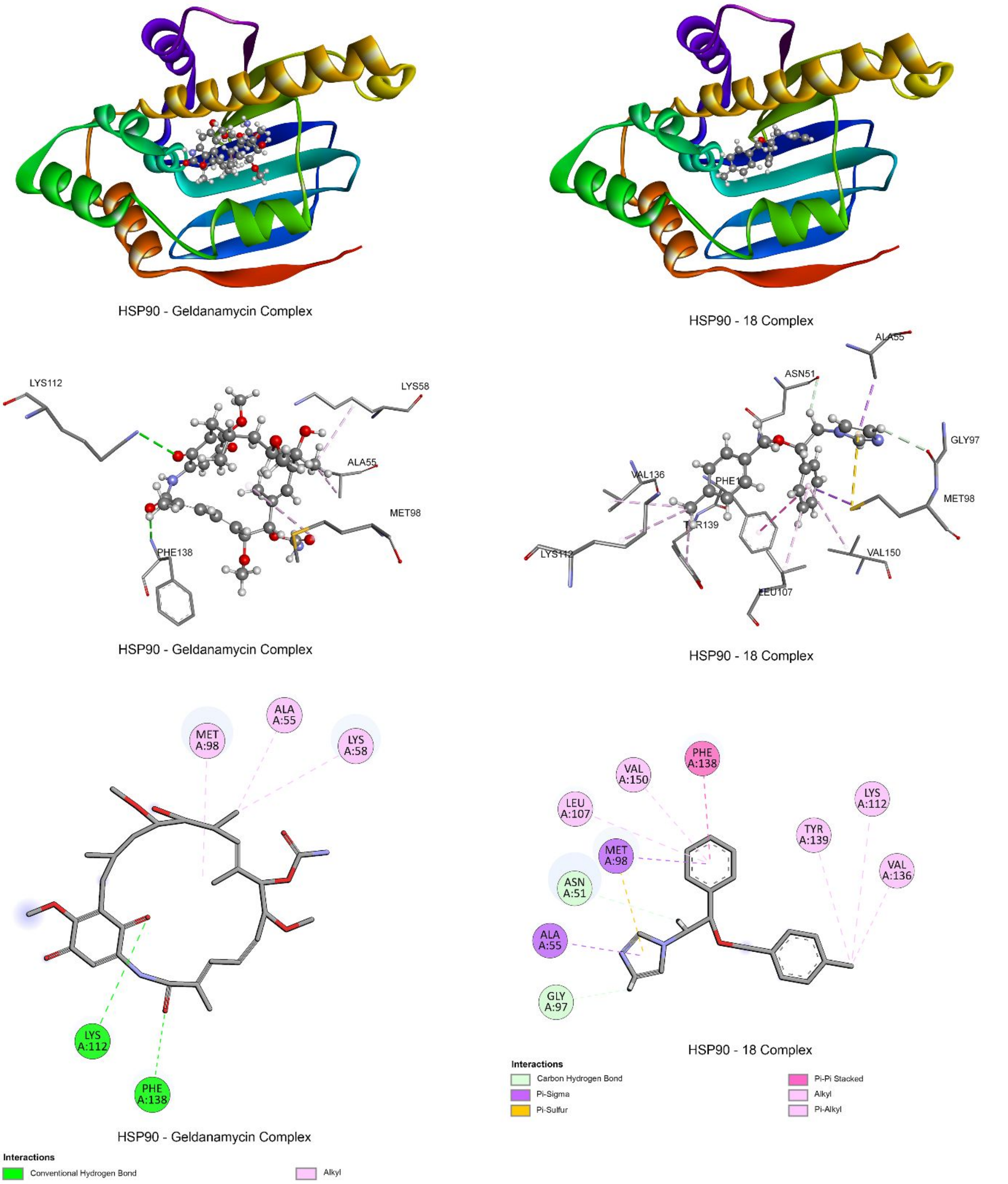

Figure S108. Binding poses and ligand-receptor interactions for HSP90 – Geldanamycin and HSP90 – 18 complexes.

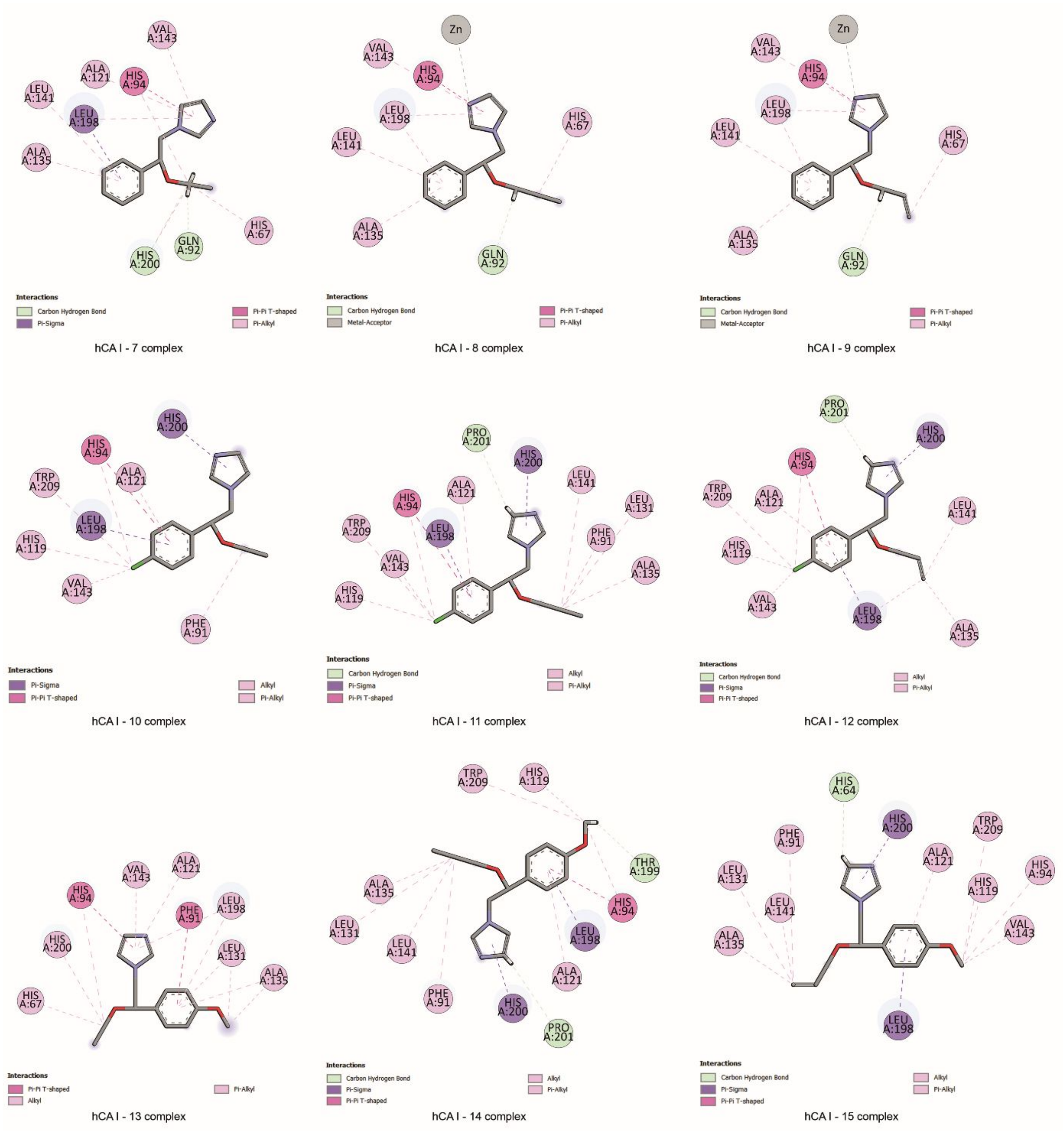

Figure S109. Ligand-receptor interactions belonging to hCA I – alkyl ether complexes.

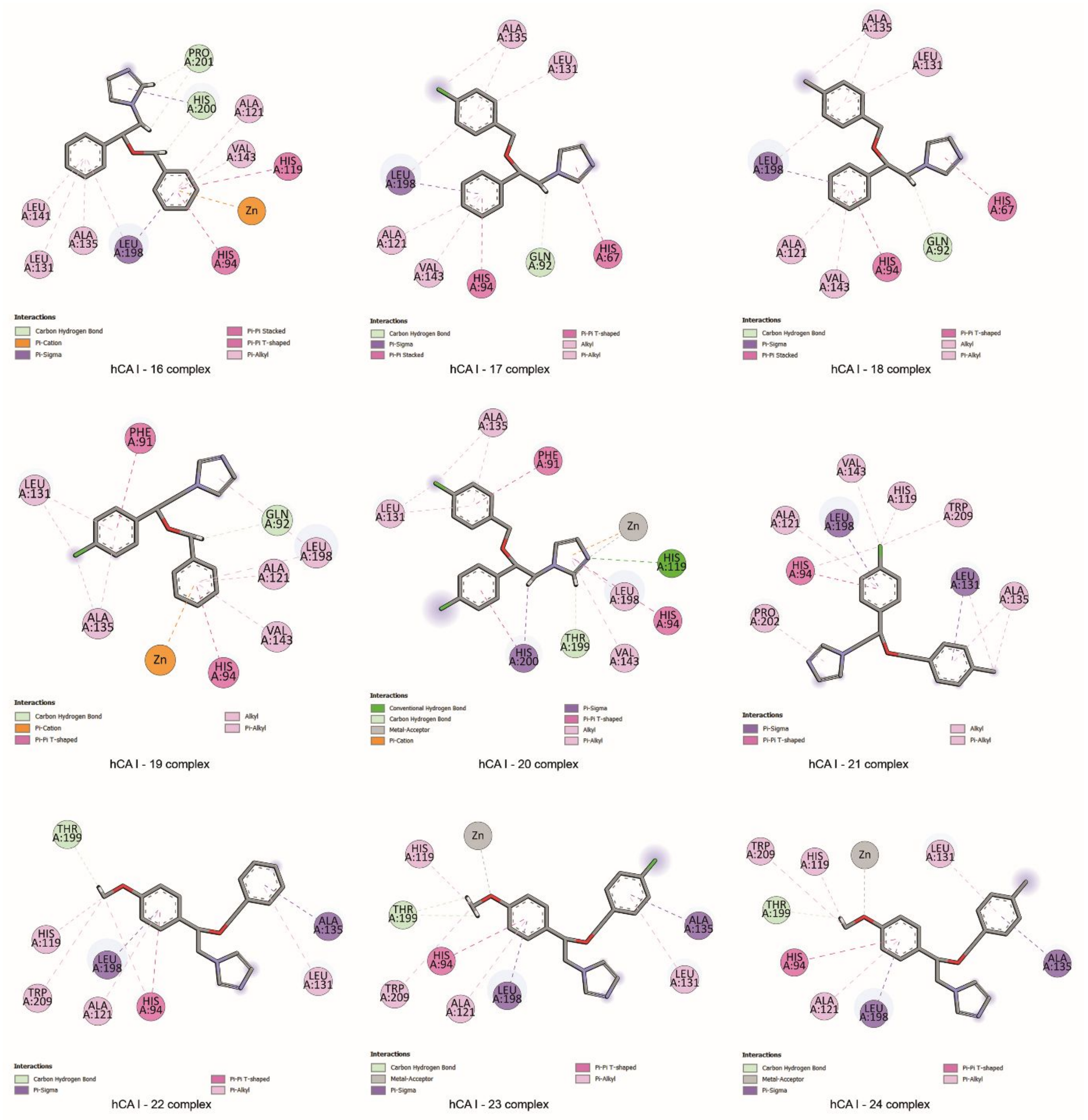

Figure S110. Ligand-receptor interactions belonging to hCA I – aryl ether complexes.

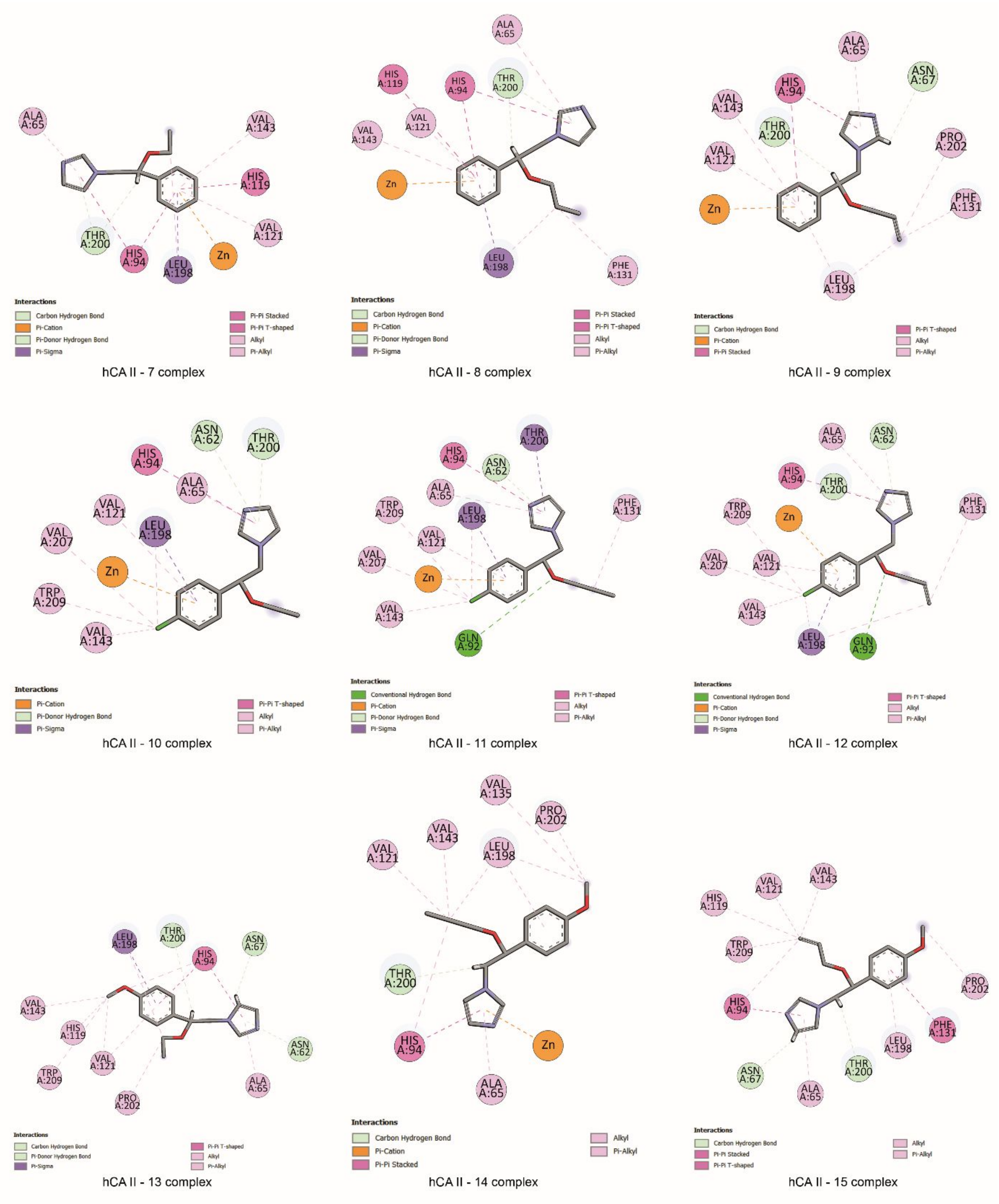

Figure S111. Ligand-receptor interactions belonging to hCA II – alkyl ether complexes.

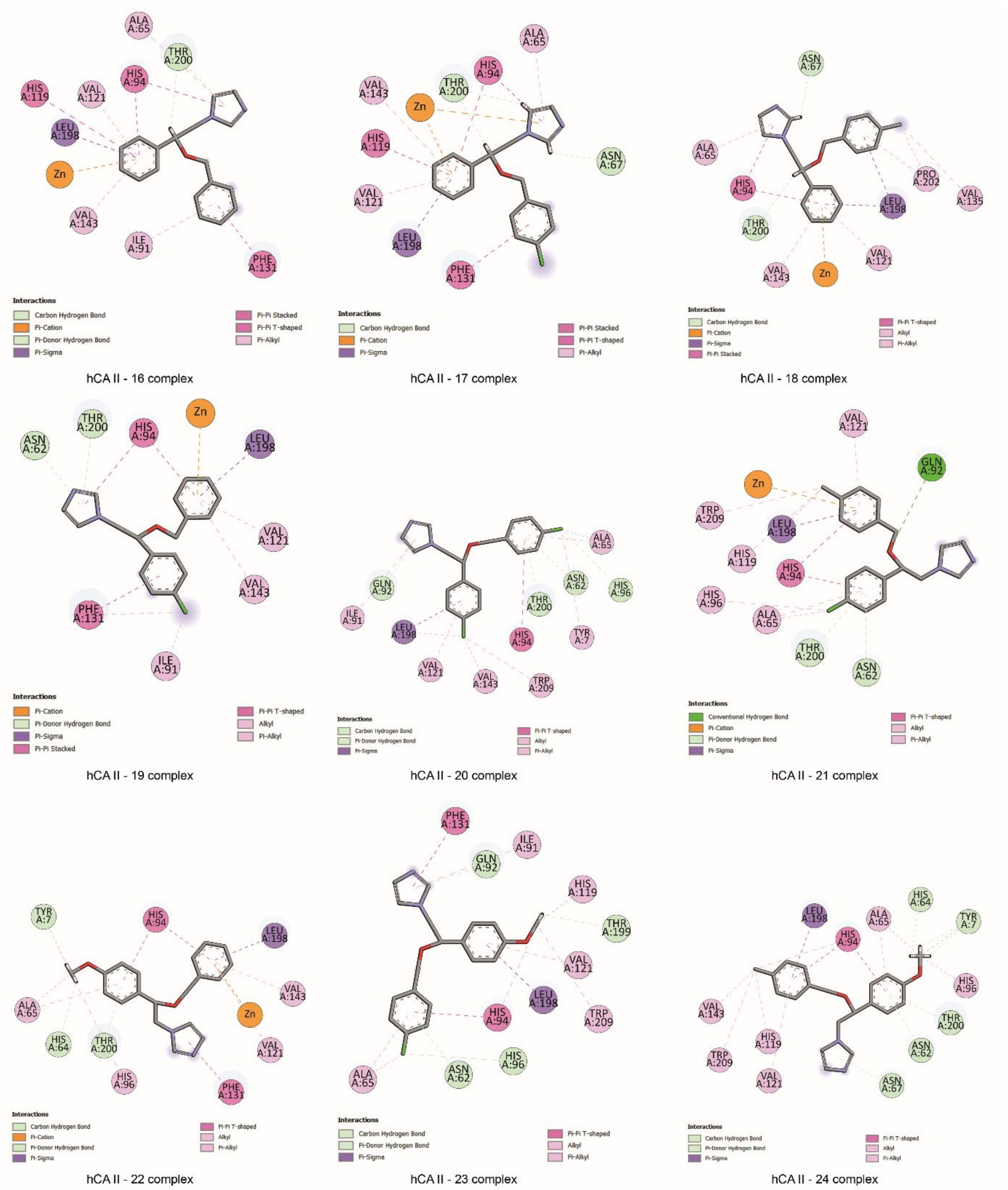

Figure S112. Ligand-receptor interactions belonging to hCA II – aryl ether complexes.

5. Binding Scores

Table S1. Binding scores obtained from molecular docking calculations.

	AutoDock Vina Binding Scores (kcal/mol)			
**Compounds**	**FGFR1**	**VEGFR2**	**EGFR**	**HSP90**
**3a**	-6.9	-7.2	-6.0	-5.8
**3b**	-7.0	-7.5	-6.1	-5.8
**3c**	-7.2	-7.3	-5.9	-5.7
**4a**	-6.6	-6.8	-5.9	-5.8
**4b**	-7.1	-7.1	-5.7	-5.8
**4c**	-7.2	-7.0	-5.8	-5.8
**7**	-6.4	-6.5	-6.1	-5.9
**8**	-6.5	-6.4	-6.1	-5.9
**9**	-6.0	-6.4	-6.2	-6.0
**10**	-7.1	-6.8	-5.9	-5.8
**11**	-6.5	-6.7	-5.9	-5.9
**12**	-6.9	-6.7	-6.0	-5.9
**13**	-6.9	-6.8	-5.9	-5.6
**14**	-6.7	-6.7	-6.0	-5.8
**15**	-6.8	-6.7	-6.0	-5.9
**16**	-7.8	-8.1	-7.3	-7.2
**17**	-7.9	-8.4	-7.3	-7.3
**18**	-8.0	-8.6	-7.4	-7.5
**19**	-7.9	-7.9	-7.4	-7.0
**20**	-8.0	-8.1	-7.3	-7.3
**21**	-8.2	-8.4	-7.4	-7.3
**22**	-7.9	-7.7	-7.1	-6.9
**23**	-7.9	-8.1	-7.1	-7.1
**24**	-8.2	-8.2	-7.1	-7.1
**Ponatinib**	-11.7	-	-	-
**Axitinib**	-	-9.7	-	-
**Gefitinib**	-	-	-8.1	-
**Geldanamycin**	-	-	-	-10.5

6. Drug-likeness and ADME Analyses

Table S2. Drug-likeness and ADME analyses of the investigated novel compounds.

	Compounds								
	**7**	**8**	**11**	**13**	**14**	**15**	**18**	**22**	**24**
**Physicochemical Properties**									
Formula	$C_{13}H_{16}N_2O$	$C_{14}H_{18}N_2O$	$C_{14}H_{17}ClN_2O$	$C_{14}H_{18}N_2O_2$	$C_{15}H_{20}N_2O_2$	$C_{15}H_{18}N_2O_2$	$C_{19}H_{20}N_2O$	$C_{19}H_{20}N_2O_2$	$C_{20}H_{22}N_2O_2$
Molecular Weight (g/mol)	216.28	230.31	264.75	246.3	260.33	258.32	292.37	308.37	322.4
Number of Heavy Atoms	16	17	18	18	19	19	22	23	24
Number of Aromatic Heavy Atoms	11	11	11	11	11	11	17	17	17
Fraction C sp^3	0.31	0.36	0.6	0.36	0.4	0.27	0.21	0.21	0.25
Number of Rotatable Bonds	5	6	6	6	7	7	6	7	7
Number of H-Bond Acceptors	2	2	2	3	3	3	2	3	3
Number of H-Bond Donors	0	0	0	0	0	0	0	0	0
Molar Refractivity	63.48	68.29	73.3	69.97	74.78	74.31	88.13	89.65	94.62
TPSA ($Å^2$)	27.05	27.05	27.05	36.28	36.28	36.28	27.05	36.28	36.28
**Lipophilicity**									
Log $P_{o/w}$ (iLOGP)	2.50	2.66	2.99	2.57	2.96	2.81	3.15	2.84	3.54
Log $P_{o/w}$ (XLOGP)	2.09	2.61	2.85	1.67	2.19	2.54	3.58	2.79	3.16
Log $P_{o/w}$ (WLOGP)	2.31	2.73	3.38	2.35	2.74	2.51	3.67	3.37	3.68
Log $P_{o/w}$ (MLOGP)	1.32	1.59	2.11	1.00	1.26	1.18	2.57	1.99	2.21
Log $P_{o/w}$ (SILICOS-IT)	2.22	2.59	3.22	2.25	2.63	2.62	3.85	3.38	3.90
Consensus Log $P_{o/w}$	2.09	2.43	2.91	1.97	2.35	2.33	3.37	2.87	3.30
**Water Solubility**									
Log S (ESOL)	S	S	S	S	S	S	MS	S	S
Log S	S	S	S	S	S	S	S	S	S
Log S (SILICOS-IT)	S	S	MS	MS	MS	MS	PS	PS	PS
**Pharmacokinetics**									
GI Absorption	High	High	High	High	High	High	High	High	High
BBB Permeant	+	+	+	+	+	+	+	+	+
P-gp substrate	-	-	-	-	-	-	-	+	-
CYP1A2 inhibitor	-	-	+	-	-	-	-	-	+
CYP2C19 inhibitor	-	+	+	+	+	+	+	+	+
CYP2C9 inhibitor	-	-	-	-	-	-	+	+	+
CYP2D6 inhibitor	-	+	+	-	+	-	+	+	+
CYP3A4 inhibitor	-	-	-	-	-	-	+	+	+
Log K_p (skin permeation)	-6.14	-5.85	-5.89	-6.62	-6.33	-6.07	-5.54	-6.20	-6.02

(cm/s)									
**Druglikeness**									
Lipinski	+	+	+	+	+	+	+	+	+
Ghose	+	+	+	+	+	+	+	+	+
Veber	+	+	+	+	+	+	+	+	+
Egan	+	+	+	+	+	+	+	+	+
Muegge	+	+	+	+	+	+	+	+	+
Bioavailability Score	0.55	0.55	0.55	0.55	0.55	0.55	0.55	0.55	0.55
**Medicinal Chemistry**									
PAINS	0	0	0	0	0	0	0	0	0
Brenk	0	0	0	0	0	1	0	0	0
Leadlikeness	No (1)	No (1)	Yes	No (1)	Yes	Yes	No (1)	Yes	Yes
Synthetic Accessibility	2.49	2.67	2.74	2.61	2.78	2.69	2.94	2.92	3.05

TPSA: Topological Polar Surface Area, S: Soluble, MS: Moderately Soluble, PS: Poorly Soluble.

7. Effect of Synthesized Compounds on hCA I and II Isoenzymes Activity

Table S3. Effect of synthesized compounds on hCA I and II isoenzymes activity (+: active, -: not effect).

Compounds	hCA I	hCA II
**3a**	**-**	**-**
**3b**	**-**	**-**
**3c**	**-**	**-**
**4a**	+	+
**4b**	+	+
**4c**	+	+
**7**	+	+
**8**	+	+
**9**	+	+
**10**	+	+
**11**	+	+
**12**	+	+
**13**	+	+
**14**	+	+
**15**	+	+
**16**	+	+
**18**	+	+

8. % Activity - inhibitor concentration graphs of all the compounds

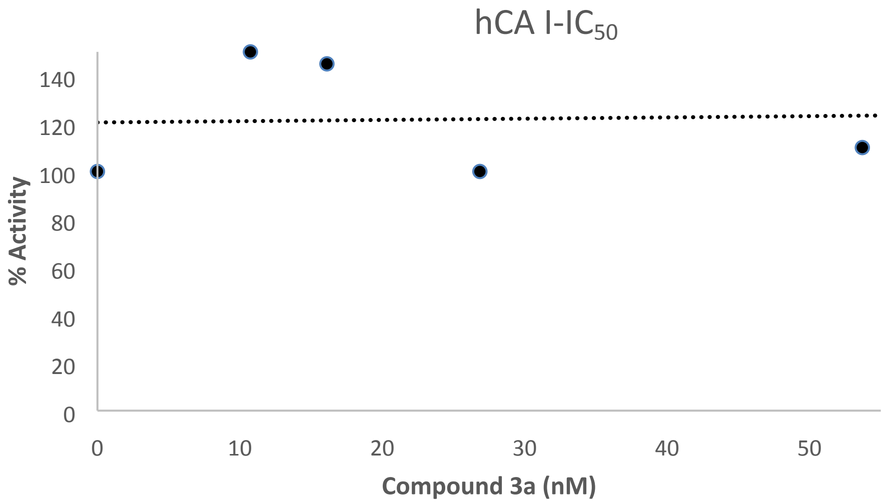

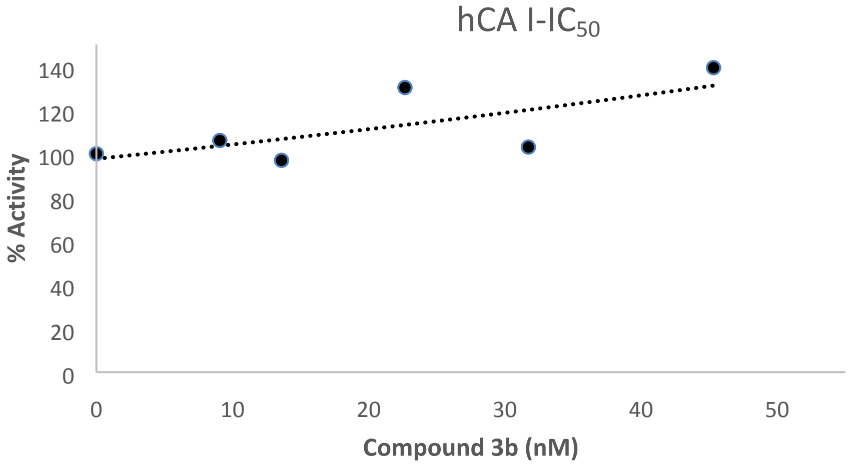

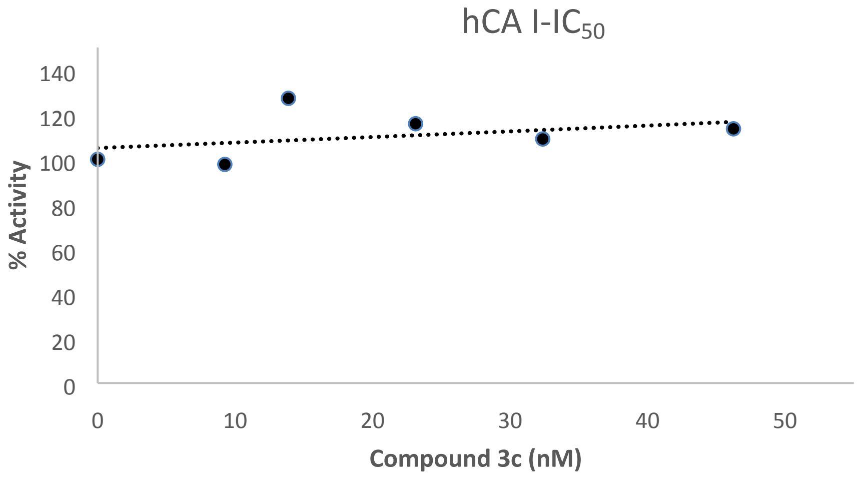

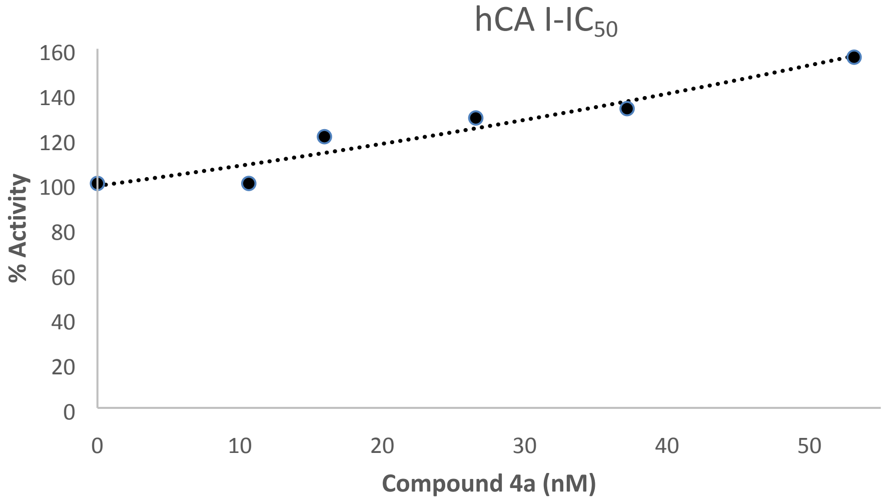
hCA I-IC50
160
140
120
100
80
60
40
20
0
% Activity
0
10
20
30
40
50
Compound 4a (nM)

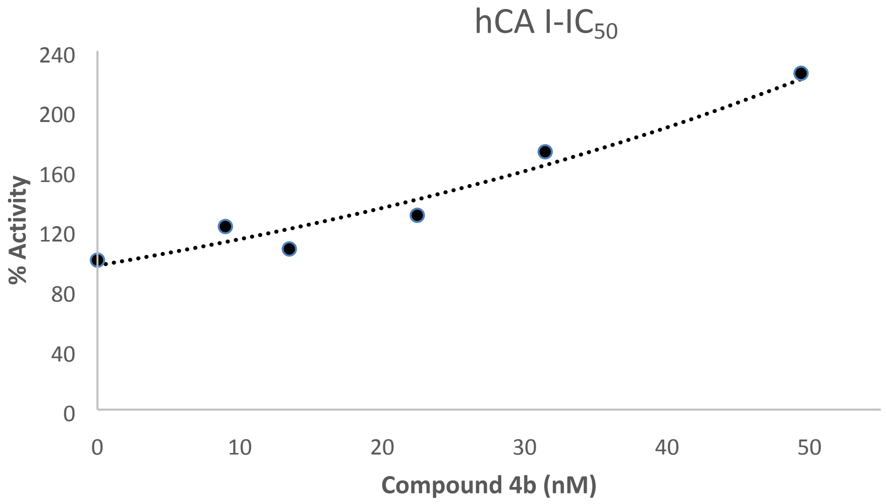
hCA I-IC50
240
200
160
120
80
40
0
% Activity
0
10
20
30
40
50
Compound 4b (nM)

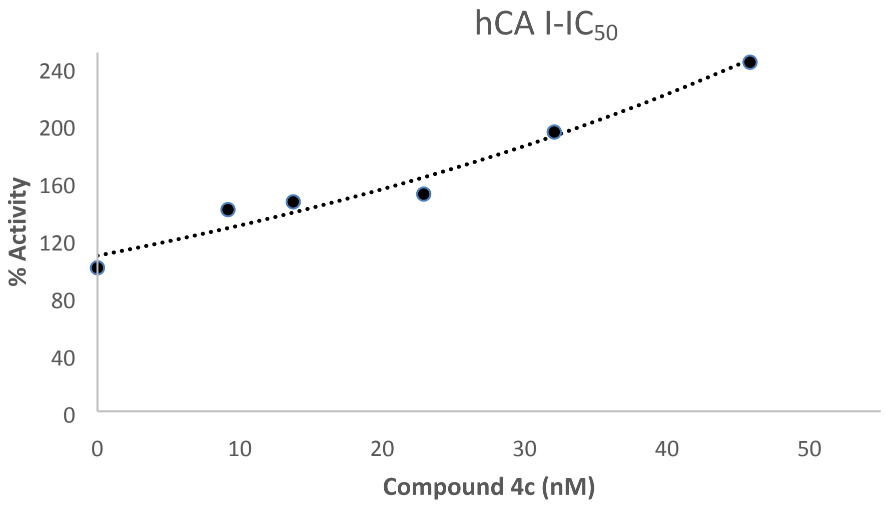
hCA I-IC50
240
200
160
120
80
40
0
% Activity
0
10
20
30
40
50
Compound 4c (nM)

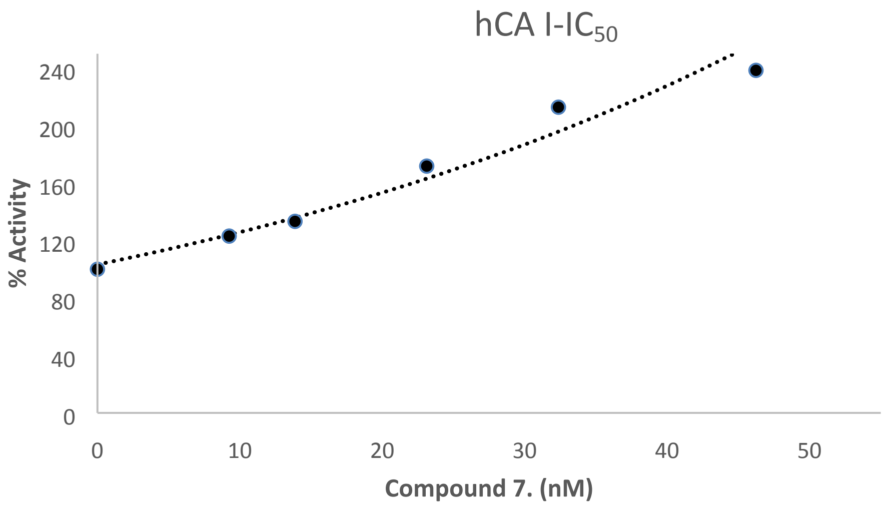

hCA I-IC$_{50}$
240
200
160
120
80
40
0
% Activity
0
10
20
30
40
50
Compound 7. (nM)

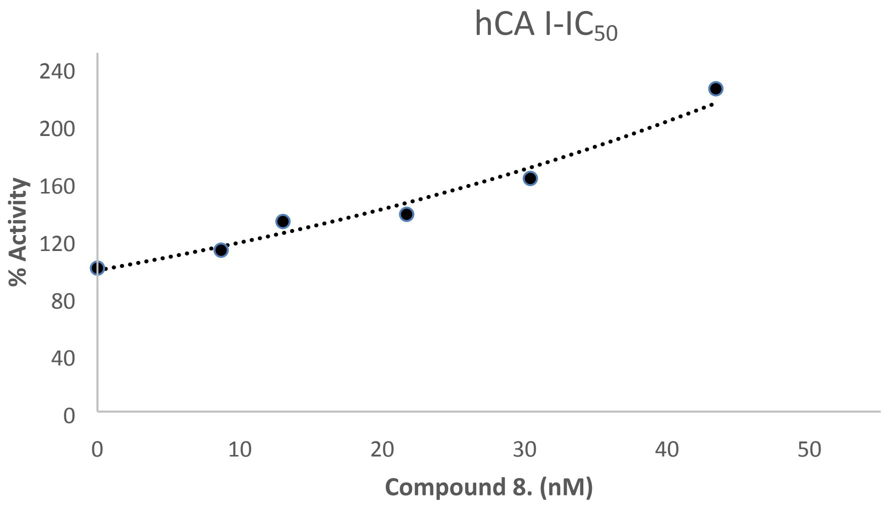

hCA I-IC$_{50}$
240
200
160
120
80
40
0
% Activity
0
10
20
30
40
50
Compound 8. (nM)

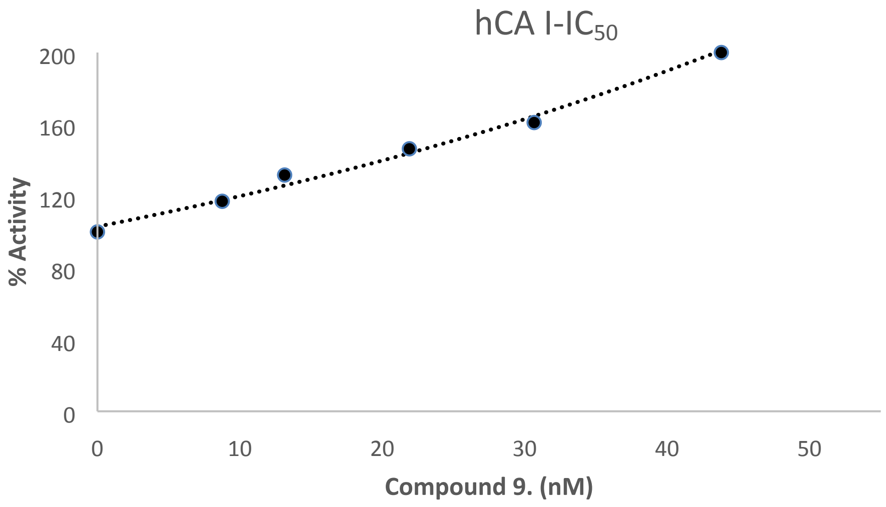

hCA I-IC$_{50}$
200
160
120
80
40
0
% Activity
0
10
20
30
40
50
Compound 9. (nM)

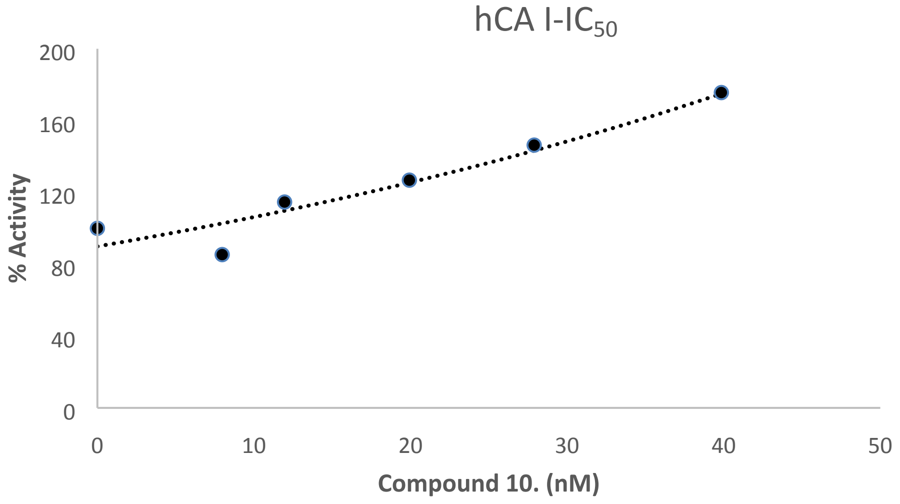
hCA I-IC50
% Activity
200
160
120
80
40
0
0
10
20
30
40
50
Compound 10. (nM)

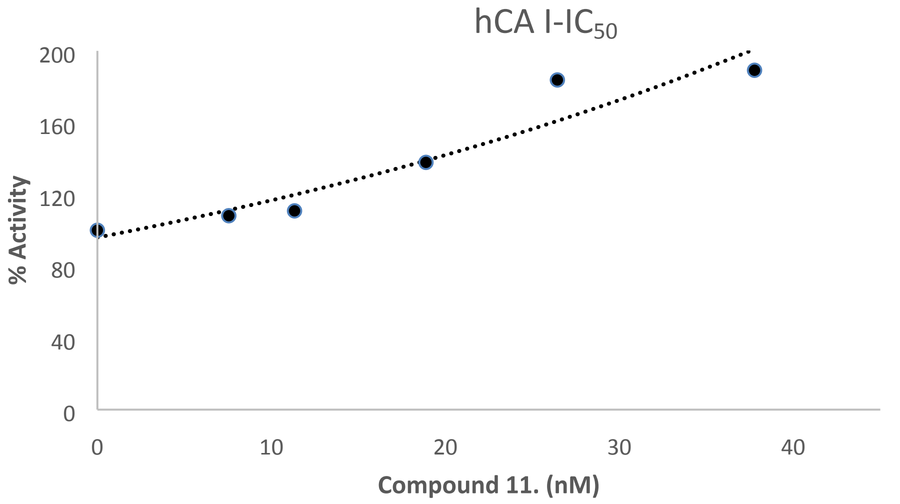
hCA I-IC50
% Activity
200
160
120
80
40
0
0
10
20
30
40
Compound 11. (nM)

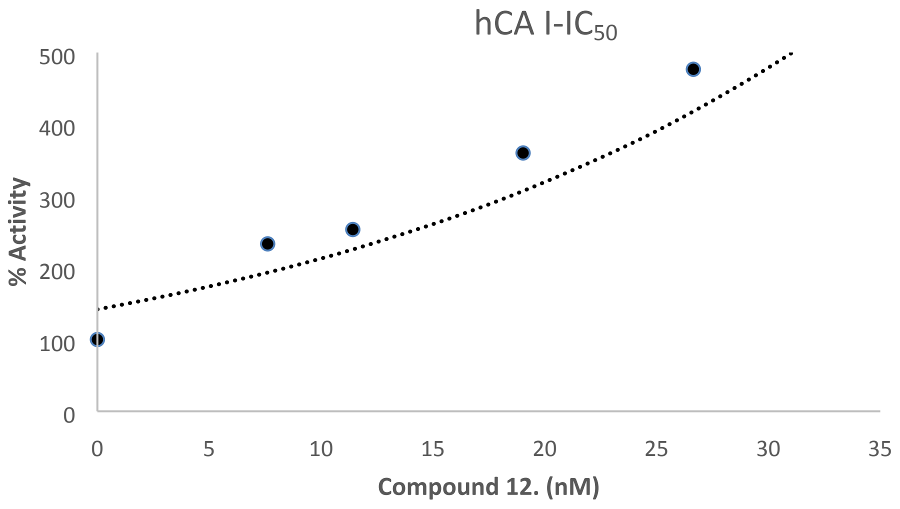
hCA I-IC50
% Activity
500
400
300
200
100
0
0
5
10
15
20
25
30
35
Compound 12. (nM)

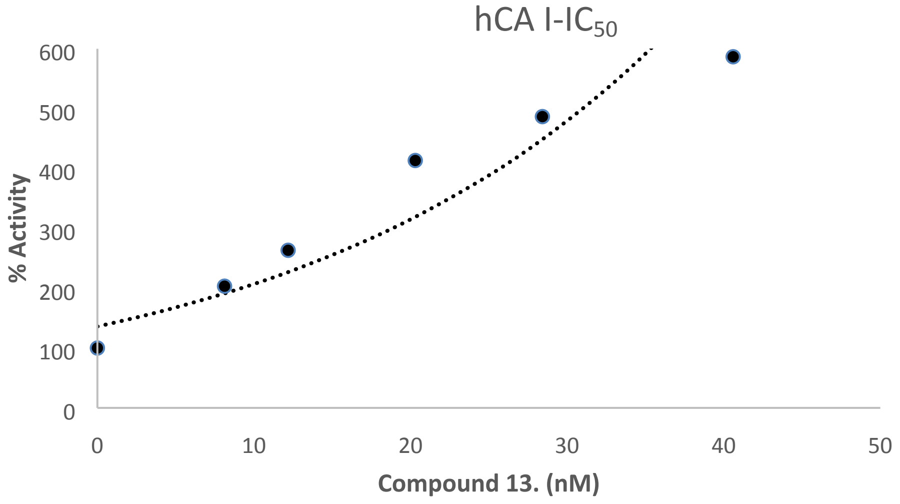
hCA I-IC$_{50}$
% Activity
0
100
200
300
400
500
600
0
10
20
30
40
50
Compound 13. (nM)

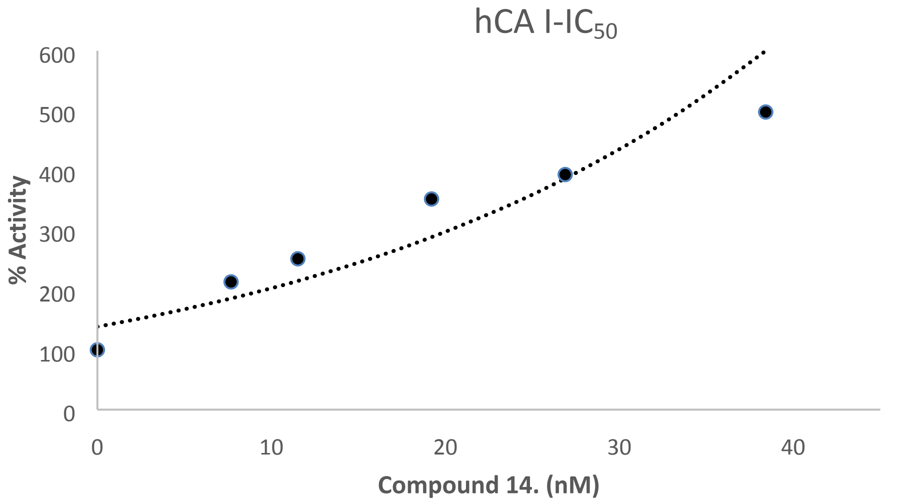
hCA I-IC$_{50}$
% Activity
0
100
200
300
400
500
600
0
10
20
30
40
Compound 14. (nM)

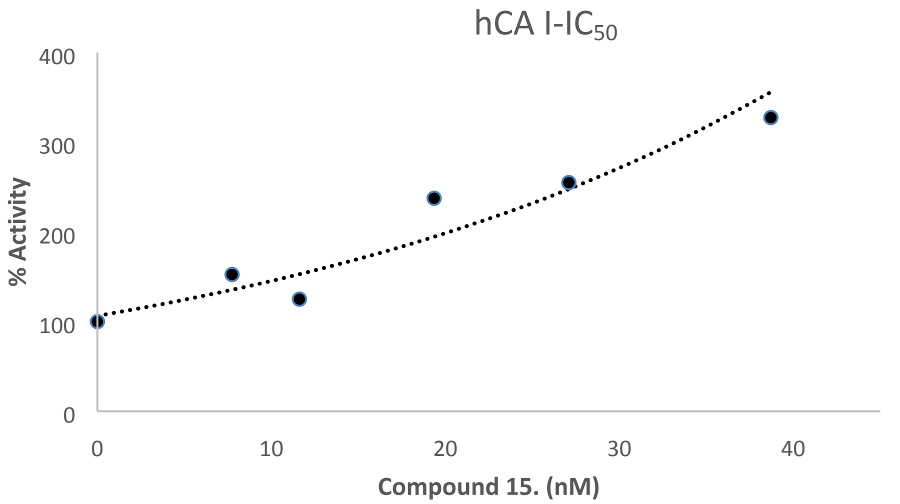
hCA I-IC$_{50}$
% Activity
0
100
200
300
400
0
10
20
30
40
Compound 15. (nM)

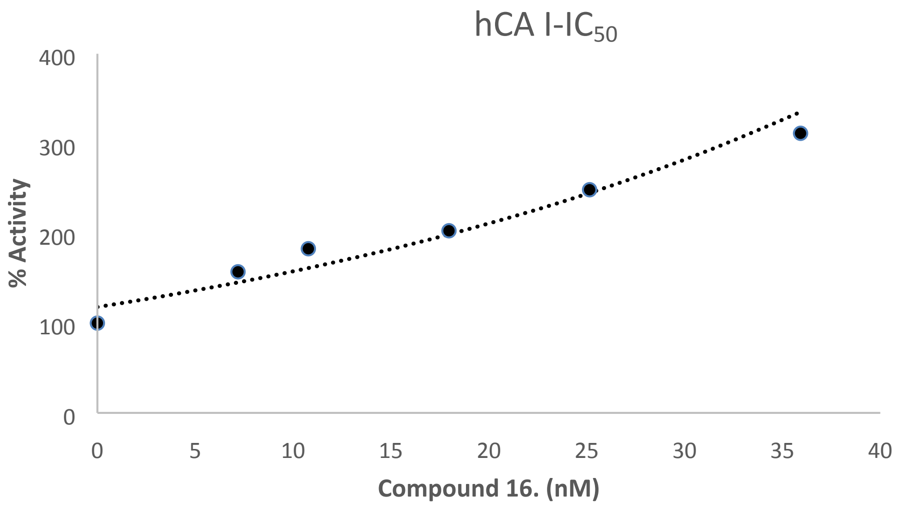
hCA I-IC$_{50}$
% Activity
400
300
200
100
0
0 5 10 15 20 25 30 35 40
Compound 16. (nM)

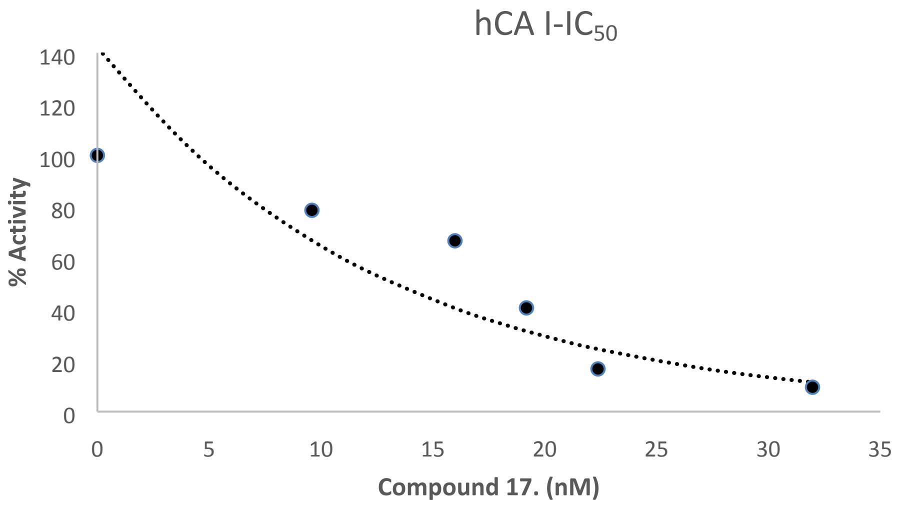
hCA I-IC$_{50}$
% Activity
140
120
100
80
60
40
20
0
0 5 10 15 20 25 30 35
Compound 17. (nM)

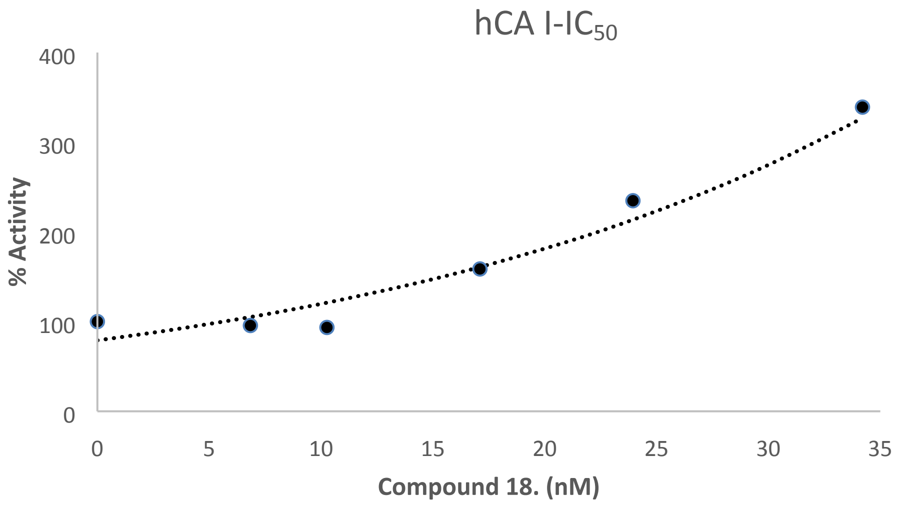
hCA I-IC$_{50}$
% Activity
400
300
200
100
0
0 5 10 15 20 25 30 35
Compound 18. (nM)

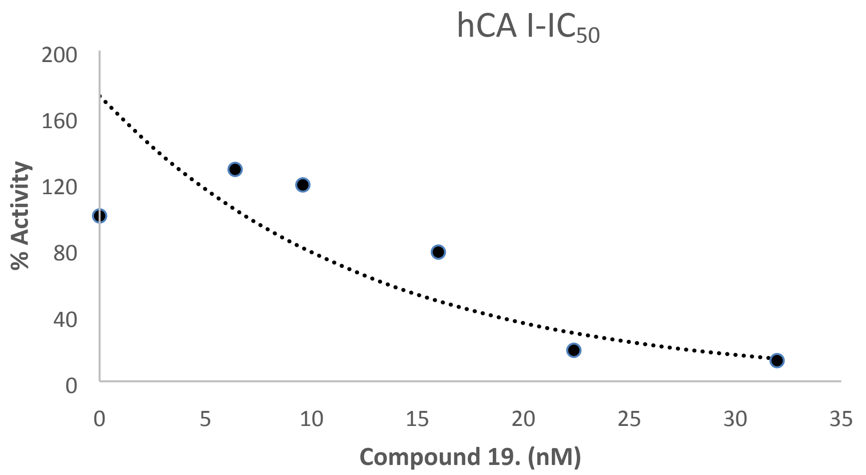
hCA I-IC50
% Activity
200
160
120
80
40
0
0
5
10
15
20
25
30
35
Compound 19. (nM)

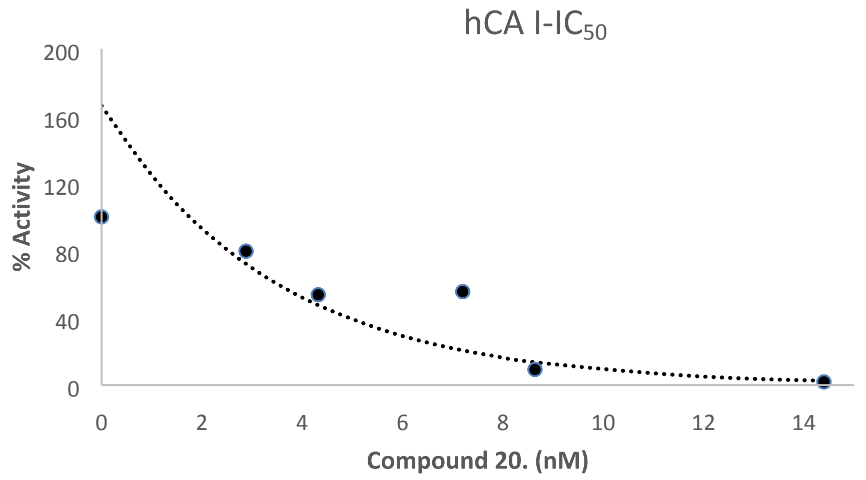
hCA I-IC50
% Activity
200
160
120
80
40
0
0
2
4
6
8
10
12
14
Compound 20. (nM)

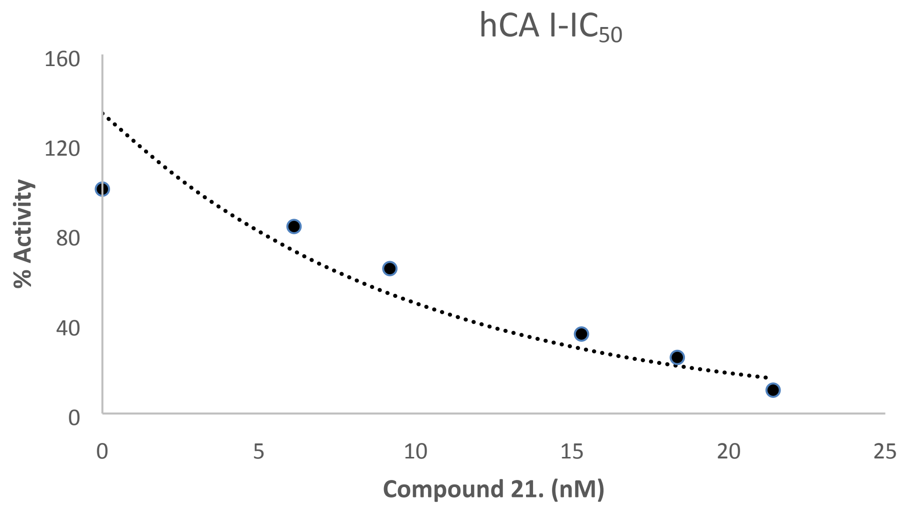
hCA I-IC50
% Activity
160
120
80
40
0
0
5
10
15
20
25
Compound 21. (nM)

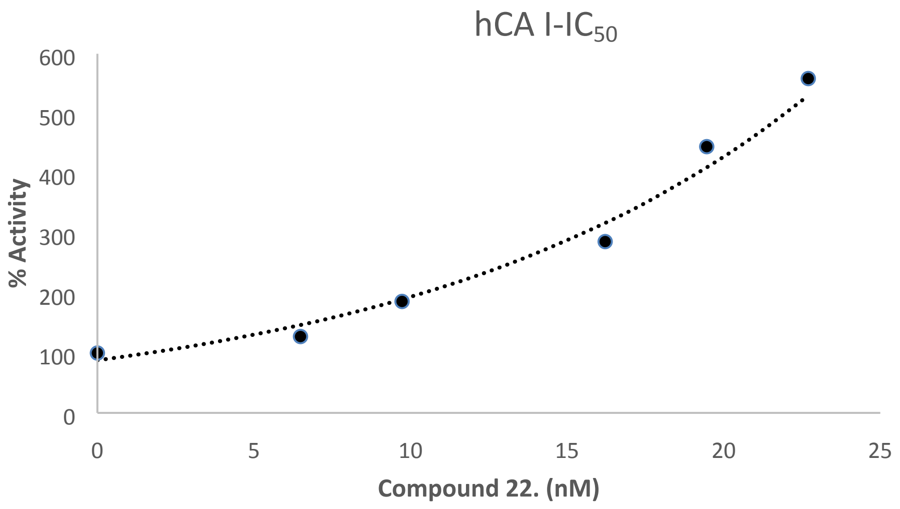
hCA I-IC50
% Activity
600
500
400
300
200
100
0
0
5
10
15
20
25
Compound 22. (nM)

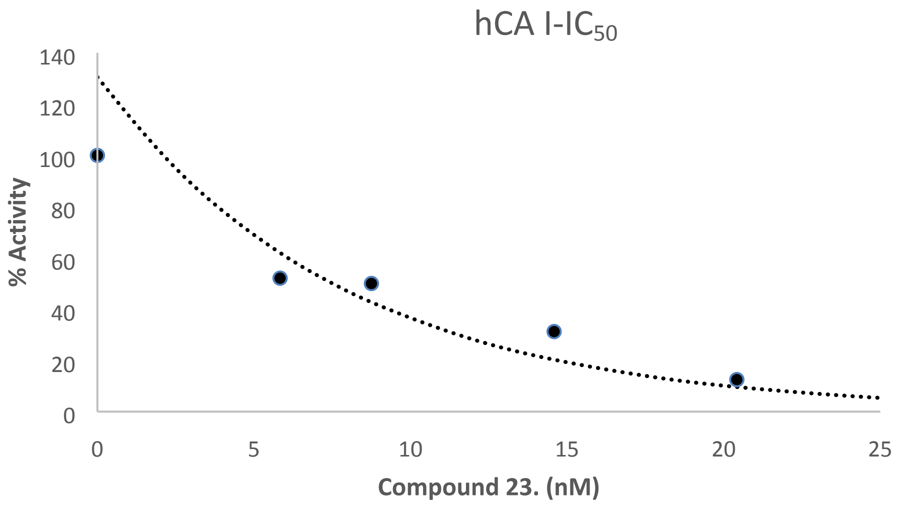
hCA I-IC50
% Activity
140
120
100
80
60
40
20
0
0
5
10
15
20
25
Compound 23. (nM)

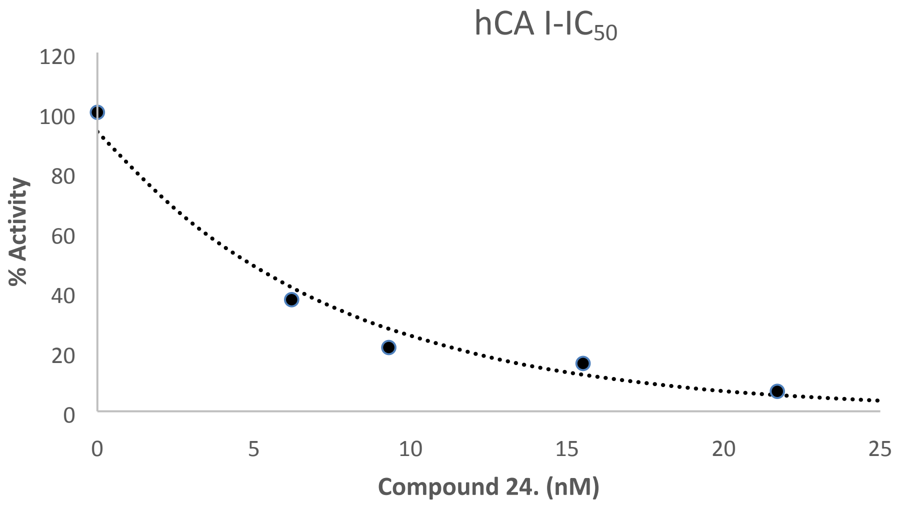
hCA I-IC50
% Activity
120
100
80
60
40
20
0
0
5
10
15
20
25
Compound 24. (nM)

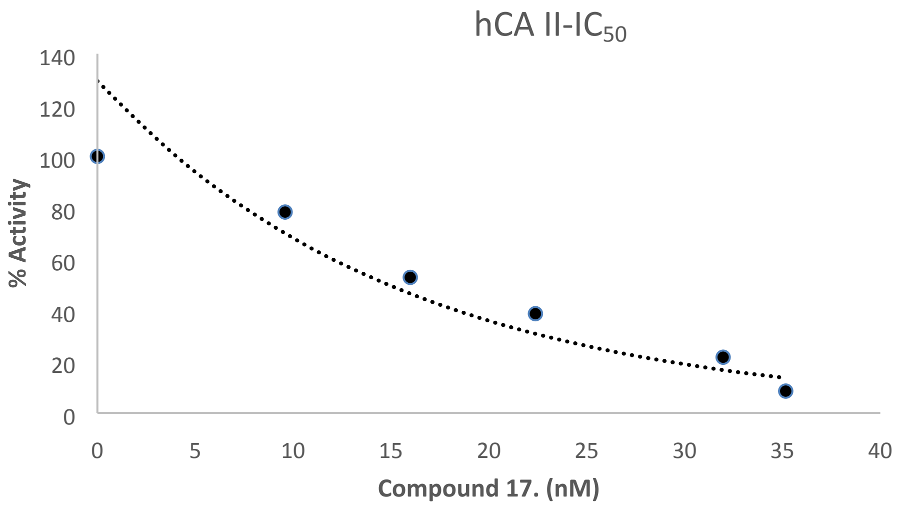
hCA II-IC50
% Activity
0
20
40
60
80
100
120
140
0
5
10
15
20
25
30
35
40
Compound 17. (nM)

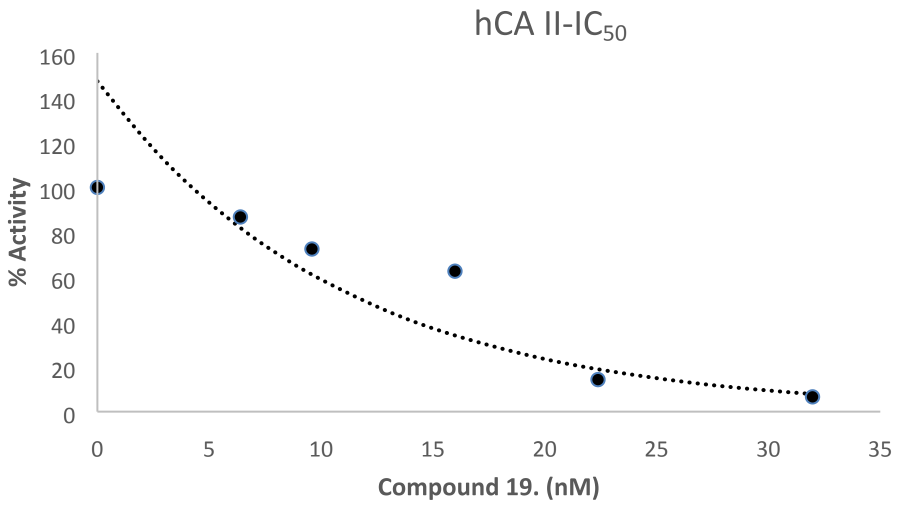
hCA II-IC50
% Activity
0
20
40
60
80
100
120
140
160
0
5
10
15
20
25
30
35
Compound 19. (nM)

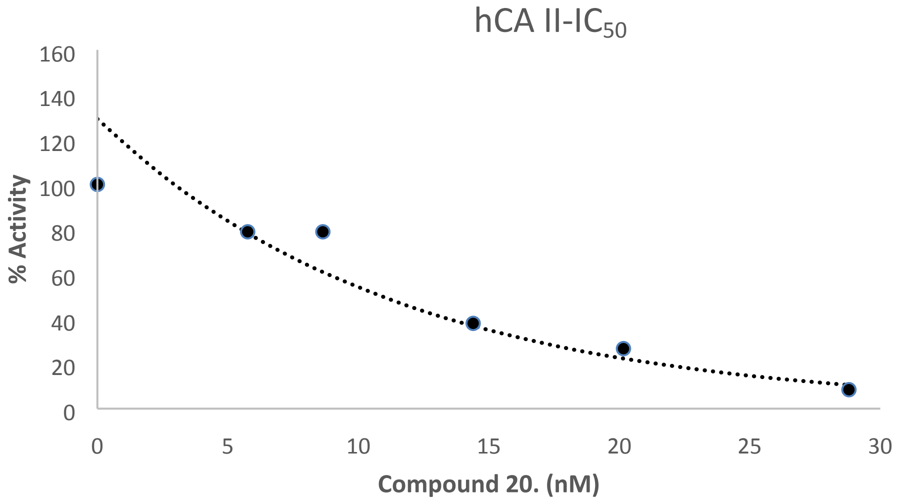
hCA II-IC50
% Activity
0
20
40
60
80
100
120
140
160
0
5
10
15
20
25
30
Compound 20. (nM)

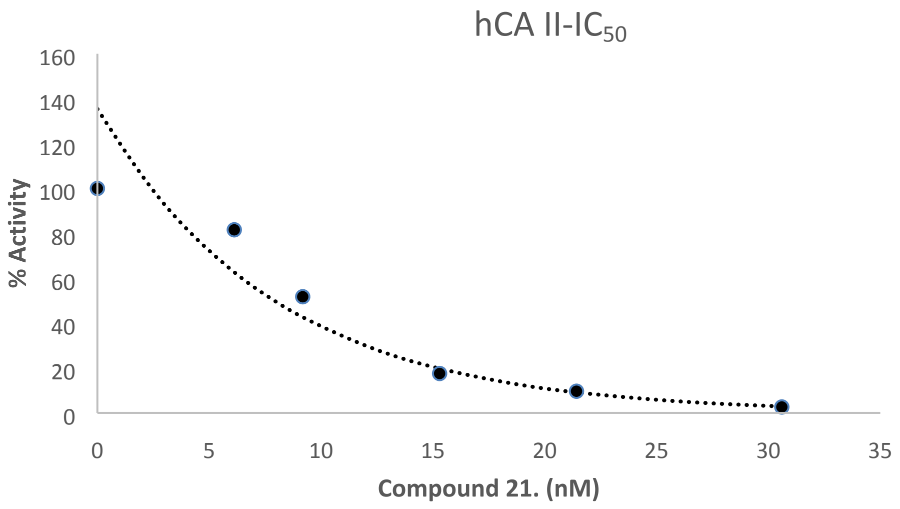

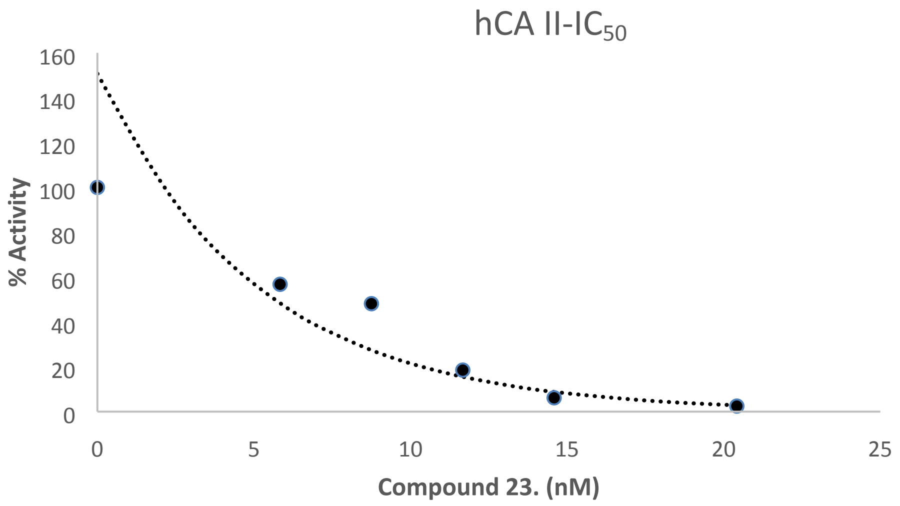

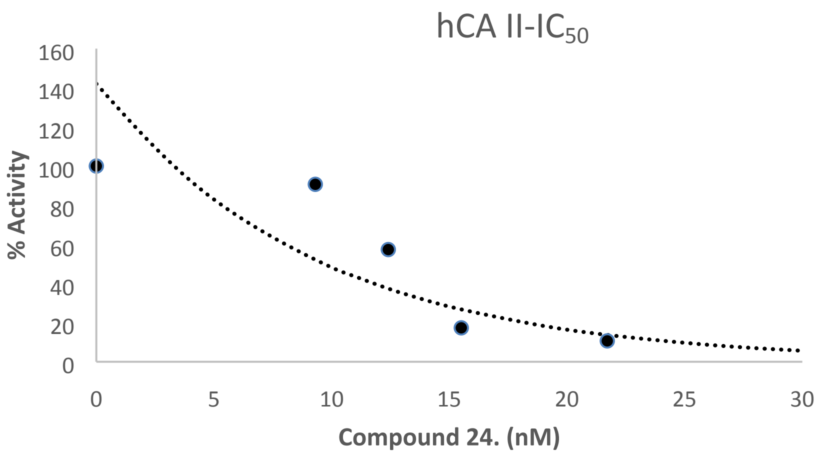

Figure S113. % Activity - inhibitor concentration graphs of all the compounds.
